# Arylthianthrenium Salts for Triplet Energy Transfer Catalysis

**DOI:** 10.1021/jacs.4c11099

**Published:** 2024-10-28

**Authors:** Yuan Cai, Triptesh Kumar Roy, Till J. B. Zähringer, Beatrice Lansbergen, Christoph Kerzig, Tobias Ritter

**Affiliations:** † Max-Planck-Institut für Kohlenforschung, 45470 Mülheim an der Ruhr, Germany; § Institute of Organic Chemistry, RWTH Aachen University, Landoltweg 1, 52074 Aachen, Germany; ‡ Department of Chemistry, Johannes Gutenberg University Mainz, 55128 Mainz, Germany

## Abstract

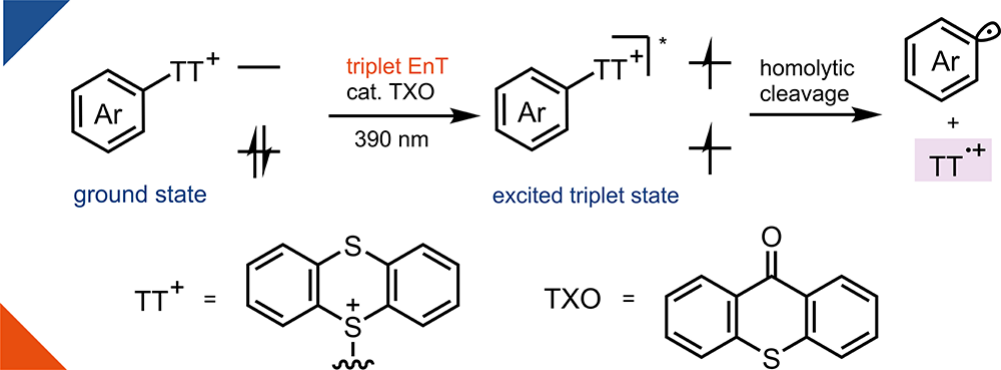

Sigma bond cleavage through electronically excited states allows synthetically useful transformations with two radical species. Direct excitation of simple aryl halides to form both aryl and halogen radicals necessitates UV-C light, so undesired side reactions are often observed and specific equipment is required. Moreover, only aryl halides with extended π systems and comparatively low triplet energy are applicable to synthetically useful energy transfer catalysis. Here we show the conceptual advantages of arylthianthrenium salts (ArTTs) for energy transfer catalysis with high energy efficiency compared to conventional aryl (pseudo)halides and their utility in arylation reactions of ethylene. The fundamental advance is enabled by the low triplet energy of ArTTs that may originate in large part from the electronic interplay between the distinct sulfur atoms in the tricyclic thianthrene scaffold, which is not accessible in either simple (pseudo)halides or other conventional sulfonium salts.

## Introduction

**Figure 1:**
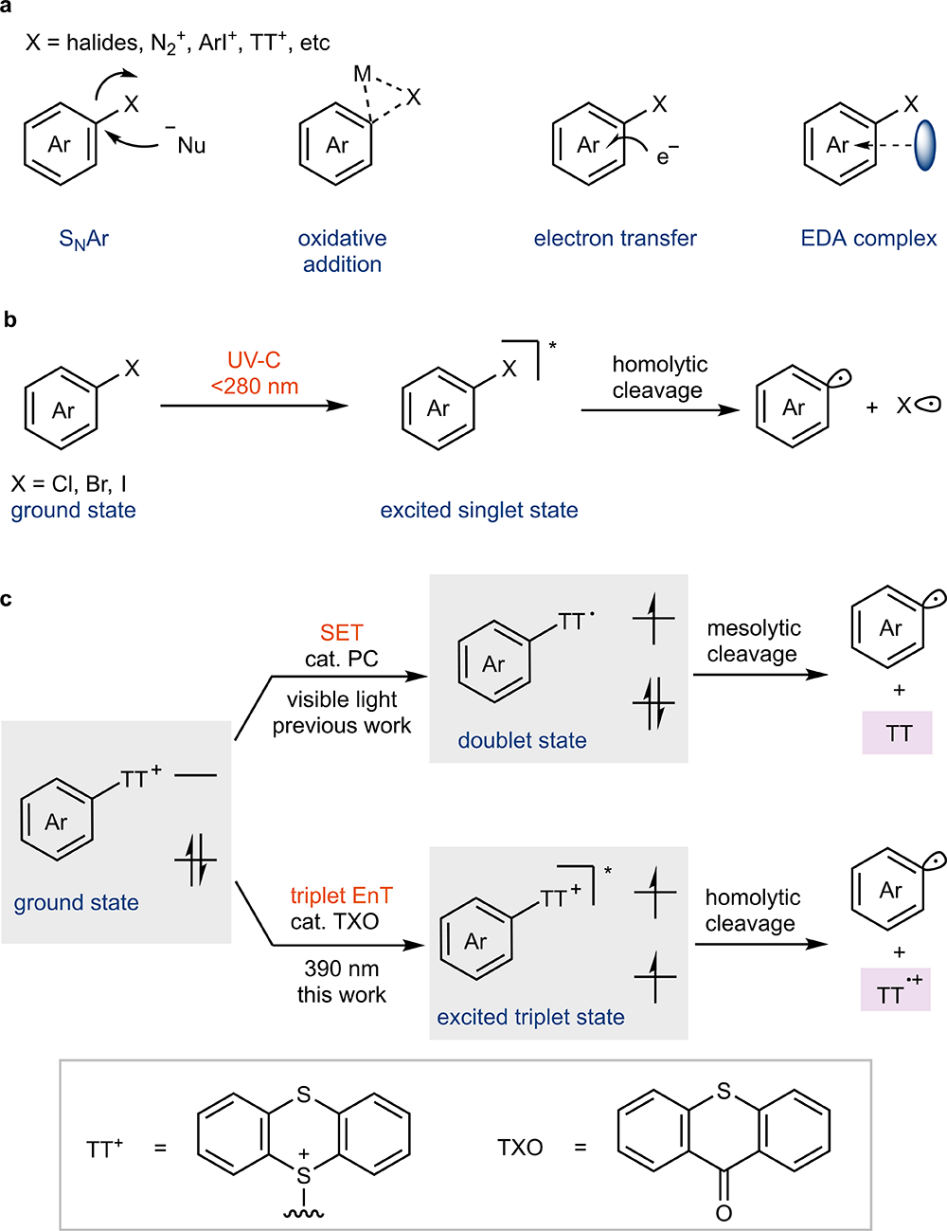
**Reactivity of aryl electrophiles in ground and excited states.** (a) Fundamental reactivity for aryl electrophiles in the ground state. Ar, aryl group; Nu, nucleophile; M, transition metal; X, (pseudo)halide; EDA, electron donor–acceptor. (b) Homolytic cleavage of C–X bonds in aryl halides from excited singlet state obtained under UV-C radiation. UV-C, Ultraviolet C. (c) Mesolytic cleavage of ArTTs through SET and homolytic cleavage of ArTTs through triplet EnT with 390 nm light. SET, single electron transfer; EnT, energy transfer; TXO, thioxanthone.

Aryl (pseudo)halides are versatile electrophiles in organic synthesis owing to the broad reactivity of their polar C–X bond in ground state (Figure 1a).^1,2^ Electronically excited aryl halides can undergo homolysis of the C–X bond to form two synthetically useful radical species, a reactivity that is usually unattainable from the ground state on account of the large bond dissociation energy (BDE) of the C–X bonds, for example around 84 kcal·mol^–1^ for aryl bromides.^3^ Yet, aryl halides typically do not absorb in the visible spectrum, so UV light, often in the UV–C spectrum (<280 nm), is required for their excitation, which delivers over 102 kcal·mol^–1^ of energy, capable of cleaving many organic bonds, including C–C (∼83 kcal·mol^–1^) and C–H bonds (∼100 kcal·mol^–1^) (Figure 1b).^4^ Visible light (380–800 nm) provides photons with less than 75 kcal·mol^–1^ energy, which allows most organic covalent bonds to remain intact. Additionally, visible light is safer, more accessible, and does not require special equipment, such as quartz glassware. However, simple aryl (pseudo)halides must typically be excited through direct excitation to a singlet state with UV-C light. Electronic excitation through triplet energy transfer (EnT) processes from an excited sensitizer are promising but have not been applied to simple aryl halides with visible light.^5−8^ Because triplet energy (*E*_T_) and spin multiplicity are transferred via concurrent electron exchange between an excited photosensitizer donor and the substrate acceptor, the excited high-energy singlet state of the substrate is circumvented. Nevertheless, sensitization of simple aryl halides requires higher triplet energy than is obtainable from photosensitizers with visible light absorption, which generally exhibit triplet energies of less than 66 kcal·mol^–1^.^6−8^ Only special aryl halides with an appropriate electronic structure have a sufficiently low triplet energy for successful excitation, for example, through extended π systems.^9^ Aryl halides with highly conjugated or fused aryl systems, such as biphenyl and naphthyl halides, have low enough triplet energies (60–64 kcal·mol^–1^)^9^ and can be used in energy transfer catalysis with visible light.^10,11^ For simple aryl halides, high energy photons (less than 280 nm) from UV light and quartz glassware must be used.^10^ High-power LEDs with high electricity-to-light conversion efficiencies are only available with wavelengths starting from about 365 nm.^12^ Other synthetically useful, colorless molecules with a triplet energy of less than 66 kcal·mol^–1^ include, for example, styrene-derived substrates,^6−8^ disulfides,^13^ benzophenone-based oxime carbonates,^14^ fused arenes,^15^ α-keto esters,^16^ N–N pyridinium ylides,^17^ and naphthyl ketones.^18^ Aryldiazonium and–iodonium compounds primarily function as electron acceptors rather than energy acceptors as a result of their high reduction potentials that can be rationalized, in part, by their positive charge, which favors capturing an electron instead of accepting energy from an excited photosensitizer.^19^ Although both single electron transfer (SET) and EnT can generate synthetically useful aryl radicals from aryl (pseudo)halides, SET produces negatively charged (pseudo)halides after mesolytic cleavage, whereas EnT generates neutral (pseudo)halogen radicals after homolytic cleavage, and thereby provides a conceptually different reactivity mode that can be exploited for distinct reaction chemistry beyond what is possible with SET chemistry.^20^ So far, a general platform for visible light-induced homolysis of the C–X bond in aryl electrophiles is lacking, both in terms of direct excitation and triplet energy transfer, and synthetically useful transformations based on excited aryl electrophiles still rely on the use of harmful UV light.^4,21^ ArTTs have been shown to participate in various mechanisms, for example, transition metal catalysis,^22^ photoredox catalysis,^23^ direct excitation with 254 nm light,^21^ and charge transfer processes.^24^ Herein, we analyze the electronic structure of arylthianthrenium salts, describe their differences to aryl halides and other aryl-based positively charged pseudohalides, such as aryldiazonium salts, and show that arylthianthrenium salts undergo productive energy transfer to enable chemistry (Figure 1c).^25^

Manipulation of HOMO/LUMO energy states and triplet energy with electron donor and acceptor substituents to obtain bipolar molecules has been widely employed in the development of phosphorescent organic light-emitting diodes (PhOLEDs) and organic photocatalysts.^26^ A priori, triplet energies cannot be derived from HOMO–LUMO gaps, yet, it is acknowledged that triplet energies are correlated with the degree of conjugation in molecules,^6,9^ and so are HOMO–LUMO gaps. Arylthianthrenium salts^22^ possess a positively charged, therefore strongly electron-accepting, sulfur atom, and a neutral, electron-donating sulfur atom, the combination of which may result in a lower HOMO–LUMO gap and in a lower triplet energy. We speculate that the lower triplet energy of ArTTs may be determined by the dipolar structure of the thianthrenium substituent itself, and not primarily influenced by the arene, and should therefore render ArTTs with varying aryls promising candidates for energy transfer catalysis with visible light. Furthermore, cationic ArTTs are less likely to engage in SET with commonly used photosensitizers when compared to other aryl-based cations such as phenyl diazonium (−0.1 V vs SCE) and –iodonium salts (−0.8 V vs SCE) due to their more negative reduction potential (−1.5 V vs SCE).^23^ Energy transfer to arylthianthrenium salts would result in an excited triplet state and the formation of aryl radicals and the persistent thianthrenium radical cation (TT^•+^) upon homolysis, which is distinct from single electron transfer (SET) and electron donor–acceptor (EDA) complex pathways that form neutral thianthrene after mesolytic cleavage. The conceptually different reactivity mode of EnT offers distinct opportunities for reaction chemistry beyond what SET or EDA complex pathways have enabled, and may result in significantly higher quantum yields and thereby energy efficiency. As a proof of concept, we demonstrate the 1,2-arylfunctionalization reactions of ethylene,^27^ a transformation currently inaccessible with other activation modes and other aryl (pseudo)halides with visible light.^28^

## Results and Discussion

### Reaction Development

**Figure 2:**
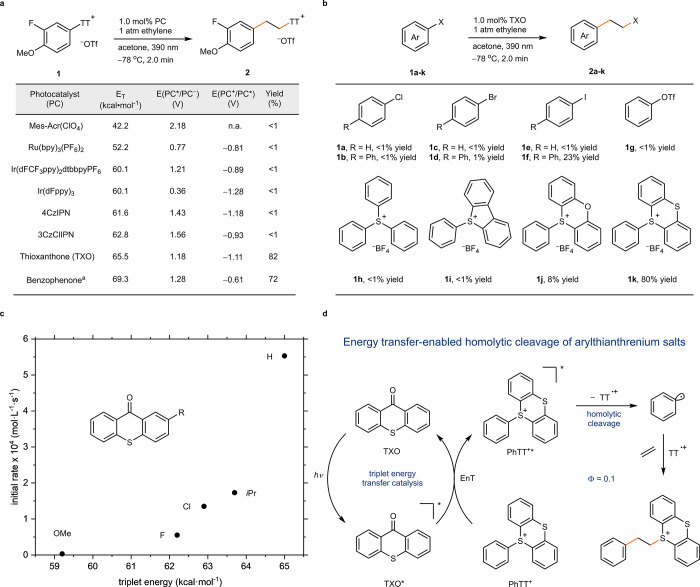
**Development and application of arylthianthrenium salts for triplet energy transfer catalysis.** (a) Evaluation of different photocatalysts in the 1,2-arylfunctionalization reactions of ethylene with arylthianthrenium salt **1**. Triplet energies (*E*_T_) and reduction potentials (*E* vs SCE) were taken from refs (29) and (30). ^a^20 mol % catalyst loading, 20 min. OTf, triflate; Mes-Acr, 9-mesityl-10-methylacridinium ion; bpy, 2,2′-bipyridyl; ppy, 2-phenylpyridyl; 4CzIPN, 2,4,5,6-tetrakis(9*H*-carbazol-9-yl)isophthalonitrile; 3CzClIPN, 2,4,6-tris(9*H*-carbazol-9-yl)-5-chloroisophthalonitrile; n.a., not available. (b) Evaluation of different aryl (pseudo)halides with thioxanthone as photosensitizer. (c) Correlation between reaction rates and triplet energies of different 2-substituted TXO photosensitizers. Triplet energies are calculated values (Table S13). (d) Proposed mechanism.

To probe our hypothesis that ArTTs could act as energy acceptor and undergo homolytic C–S bond cleavage, we exposed arylthianthrenium salt **1** to 390 nm LED irradiation in the presence of various photocatalysts under 1 atm of ethylene gas. Efficiency of the reaction correlated with the triplet energy of the photosensitizer and not with the reduction potentials of the excited state (Figure 2a), which points toward an EnT process as opposed to an SET process. By employing the commonly used thioxanthone (TXO) photosensitizer, with a triplet energy of 65.5 kcal·mol^–1^,^31^ the desired arylethyl thianthrenium salt **2** was formed in 82% yield within 2 min at −78 °C. No reactivity was observed under the optimized conditions in the absence of TXO, even with an extended reaction time of 30 min or with substrates having extended conjugation (Scheme S1), which shows that direct excitation of the arylthianthrenium slats is less efficient than energy transfer from an excited sensitizer. At 25 °C, the solubility of ethylene in acetone is significantly lower than at −78 °C, which leads to the formation of hydrodefunctionalized byproducts via hydrogen abstraction by aryl radicals. Longer reaction time results in lower yield, presumably due to unproductive energy transfer to the product alkyl thianthrenium salt. The catalyst loading can be as low as 1.0 mol %, which is uncommon when utilizing TXO as a photocatalyst because of the occurrence of undesired hydrogen atom abstraction with excited TXO.^32^ The low catalyst loading can be attributed to either the absence of a proper hydridic hydrogen atom in the reaction or efficient energy transfer facilitated by significant overlap between excited TXO and thianthrenium salts (vide infra). Other photosensitizers with triplet energies below 62.8 kcal·mol^–1^ all exhibit less than 2% conversion. Benzophenone, a photosensitizer with a higher triplet energy of 69.3 kcal·mol^–1^,^29^ requires a catalyst loading of 20 mol % and a prolonged reaction time of 20 min to afford a similar yield, likely due to the low absorption coefficient at 390 nm. Phenyl chloride (**1a**), bromide (**1c**), iodide (**1e**), and triflate (**1g**) do not show reactivity under the same conditions (Figure 2b), likely owing to their high triplet energy (∼82 kcal·mol^–1^),^9^ which renders the triplet state energetically inaccessible with visible light sensitization. Although biphenyl halides (**1b**, **1d**, **1f**) have an energetically reachable triplet energy of around 63 kcal·mol^–1^,^9^ they do not participate in an efficient reaction, possibly on account of prohibitively high BDEs of the C–X bonds,^33^ or less efficient energy transfer owing to lower overlap with the excited sensitizer compared to thianthrenium salts (vide infra). Other phenyl sulfonium salts, like triphenylsulfonium (**1h**)^34^ and 5-phenyldibenzothiophenium salts (**1i**),^35^ show negligible reactivity even with a reaction time of 30 min (Scheme S2), possibly as a result of the instability of the diphenyl sulfide– or dibenzothiophene radical cation,^36^ the high triplet energy of **1h**, insufficient overlap, or a combination of these factors. Although the phenoxathiin radical cation is also persistent,^36^ reaction of its corresponding salt (**1j**) is less efficient than with arylthianthreniums.

### Mechanistic Investigations

Diaryl ketones are often employed as triplet photosensitizers rather than photoredox catalysts, primarily owing to their high triplet energy, notable efficiencies in intersystem crossing (typically Φ_ISC_ > 0.90), and long-lived triplet excited states of approximately 50 μs.^29^ Use of several 2-substituted TXOs with different triplet energy and reduction potentials illustrates a positive correlation between triplet state energy and reaction rates, which is consistent with the involvement of an EnT pathway (Figure 2c). The absence of a positive correlation between reaction rates and redox potentials of TXOs in both ground and excited states renders an SET pathway less likely (Figure S8). Furthermore, electronically excited TXO (−1.11 V vs SCE) and benzophenone (−0.61 V vs SCE)^29^ exhibit insufficient reduction potentials for single electron reduction of ArTTs (∼ – 1.5 V vs SCE).^23^ The lack of an observable interaction between ArTTs and TXO in the ground state, as observed from UV–vis studies, renders the formation of an electron donor–acceptor (EDA) complex unlikely (Figure S4).^24^ Triplet quenchers, such as anthracene and cyclooctatetraene, decelerate or even inhibit the reaction, which is also consistent with an EnT pathway (Table S10). A quantum yield value of Φ = 0.1 is below 1 and consistent with a pathway that does not proceed via a chain mechanism. The observed positive but relatively small Hammett ρ value of 1.8 (Figure S10) aligns with the reduction of positive charge on the aromatic ring in the transition state. The bond dissociation energy (BDE) of the exocyclic C–S bond in PhTT^+^ has been computed to be only at 64.2 kcal·mol^–1^, slightly lower than its triplet state energy (65.5 kcal·mol^–1^), which implies the prospect for synthetically useful homolytic cleavage following sensitization.

**Figure 3:**
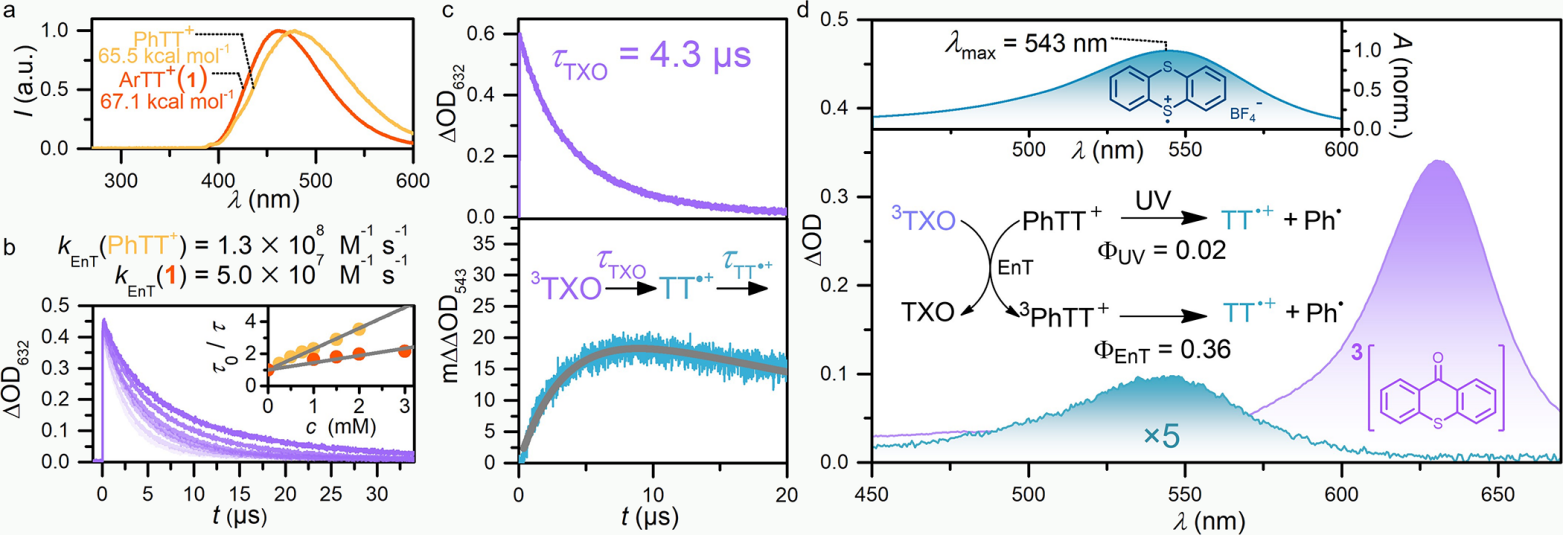
**Mechanistic studies through photophysical analysis.** (a) Phosphorescence measurements of PhTT^+^ and **1** at 77 K. (b) Stern–Volmer analysis of ^3^TXO quenching by PhTT^+^ and **1**. (c) Kinetic traces of ^3^TXO quenching (top) and isolated TT^•+^ formation and decay (bottom). (d) Transient absorption spectra of 50 μM TXO and 1 mM PhTT^+^ after 50 ns (purple) and 50 μs (cyan) and comparison of direct and sensitized bond homolysis. Inset: absorption spectrum of thianthrenium radical cation (as tetrafluoroborate).

To obtain further mechanistic insights, we carried out detailed laser flash photolysis experiments. The 77 K phosphorescence spectra obtained upon pulsed excitation for PhTT^+^ and ArTT^+^ (**1**) give experimental triplet energies (Figure 3a) that are in agreement with computed *E*_T_ values (see below) and comparable to that of ^3^TXO. In line with an almost isoenergetic (PhTT^+^) and a slightly thermodynamically uphill (ArTT^+^, **1**) EnT process, we measured quenching rate constants that are slower than the diffusion limit by about 2 orders of magnitude (Figure 3b). Such a fast energy transfer rate is consistent with the estimated and expected time scales for highly efficient energy transfer reactions and does not indicate a chain reaction mechanism (see Supporting Information page S57). Transient absorption studies revealed that upon ^3^TXO quenching a single intermediate (Figure 3c) with a characteristic spectrum in the visible range is formed, which we could unambiguously assign to TT^•+^ (Figure 3d).^37^ TXO-derived products that would result from a photoinduced SET can be excluded,^38^ substantiating the anticipated EnT process followed by homolytic bond cleavage. In contrast, SET is the primary quenching mechanism with Ir(dFppy)_3_ (Figure S1). Quantitative laser experiments with the excitation wavelength 355 nm allowed us to compare the quantum efficiency of TT^•+^ and aryl radical (that do not absorb in our detection range) generation upon direct UV excitation and sensitization at 20 °C. Based on our results, the EnT approach is inherently more efficient by as much as a factor of 18, highlighting another advantage of the strategy presented herein. These mechanistic studies explain the impressively short reaction times and considerable overall quantum yield observed at lower temperatures. We believe that highly efficient photocatalytic reaction protocols as presented herein are required for the development of photoreactions on a larger scale and industrially scalable photoreactions.

Based on our collective experimental findings, we propose an operative mechanism as shown in Figure 2d, in which electronically excited TXO sensitizes ArTTs through energy transfer to yield triplet-excited ArTTs that undergoes rapid homolysis of the labile C–S bond to produce an aryl radical and persistent TT^•+^. The aryl radical adds to ethylene to form a highly reactive primary homobenzyl radical. At low conversion, when the concentration of the persistent TT radical cation is low, the transient aryl and alkyl radicals may preferentially undergo side reactions, such as hydrodefunctionalization and dimerization, the products of which have been observed under the optimized reaction conditions. As the reaction progresses, the persistent TT^•+^ can accumulate and eventually recombine with the alkyl radical to form the desired product. Because TT^•+^ is persistent, as opposed to radicals derived from other aryl (pseudo)halides, productive radical recombination is not challenged by side reactions resulting from TT^•+^, as could be expected from other radicals, such as hydrogen atom abstraction from chlorine and bromine radicals,^39^ and decomposition of diphenyl sulfide-^34^ or iodobenzene radical cations.^40^ The observation of crossover products in a radical scrambling experiment (Figure S9) using two different salts, Ar^1^TT^+^ and Ar^2^TFT^+^, indicates that radicals escape from the solvent cage prior to the C–S bond formation process.

### Computational Study of Ground and Triplet States of Aryl (Pseudo)halides

**Figure 4:**
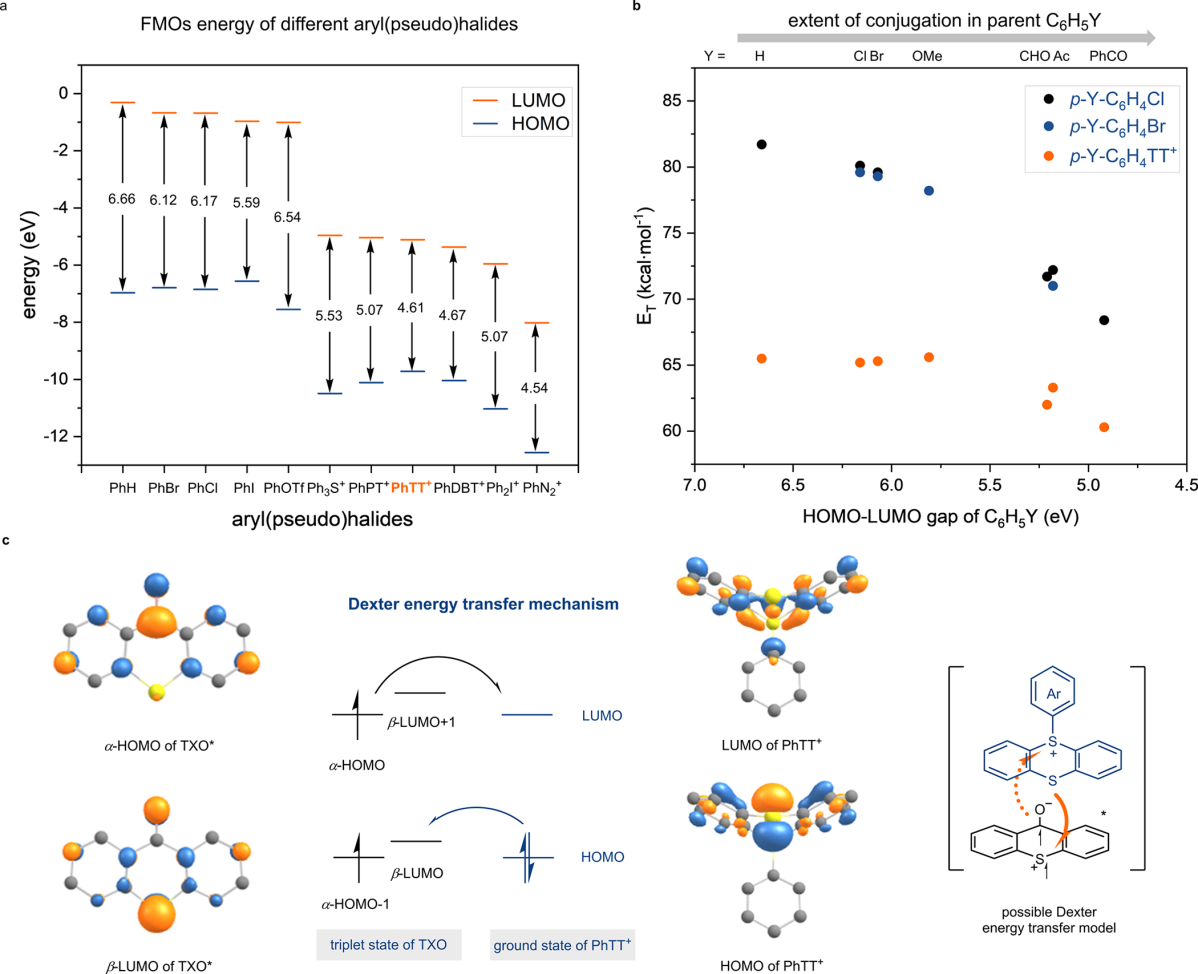
**Computational study of aryl (pseudo)halides and Dexter energy transfer mechanism.** (a) Comparison of frontier molecular orbitals energy of different aryl (pseudo)halides. FMOs, frontier molecular orbitals; Ph, phenyl; OTf, triflate; Ph_3_S^+^, triphenylsulfonium; PhPT^+^, 10-phenylphenoxathiinium; PhDBT^+^, 5-phenyldibenzothiophenium; Ph_2_I^+^, diphenyl iodonium salt; PhN_2_^+^, phenyl diazonium salt. (b) Relationship between triplet energy of aryl (pseudo)halides *p*-Y-C_6_H_4_X and conjugation extent of their parent C_6_H_5_Y. Triplet energies for *p*-Y-C_6_H_4_Cl and *p*-Y-C_6_H_4_Br were taken from ref (9). Ac, acetyl group; *E*_T_, triplet energy; X, (pseudo)halide; Y, substituent. (c) Dexter energy transfer mechanism and possible Dexter energy transfer model.

Density functional theory (DFT) calculations were conducted to elucidate the conceptual difference between ArTTs and other aryl (pseudo)halides in energy transfer chemistry. Although the bent geometry of the thianthrenium framework limits extensive conjugation potential,^22^ the calculation results predict that PhTT^+^ exhibits a narrower HOMO–LUMO gap in comparison to phenyl halides and other structurally similar phenyl sulfonium salts (Figure 4a). As for other positively charged aryl-substituted salts, the LUMO of PhTT^+^ is significantly lower than for neutral aryl halides, yet its HOMO is comparatively high when compared to the other cations, possibly a result of the electron-rich neutral sulfur atom, which leads to a smaller HOMO–LUMO gap. PhTT^+^ is computed to have a triplet energy of 65.5 kcal·mol^–1^ that is in agreement with the experimental value, significantly lower than that of corresponding phenyl halides (∼82 kcal·mol^–1^)^9^ and triphenyl sulfonium salt (∼75 kcal·mol^–1^).^34^ While phenyl diazonium and -iodonium salts also possess low triplet energies of 61.8 kcal·mol^–1^ (Table S14) and 64.3 kcal·mol^–1^, respectively,^40−42^ their lower LUMO energy levels likely result in higher electron affinity and faster electron transfer than for ArTTs.^19^ DFT calculations also indicate that the frontier molecular orbitals of phenyl halides are mainly located within the aromatic π system, while the TT framework contributes significantly to the frontier molecular orbitals of ArTTs (Table S14). Likewise, the triplet state energies of ArTTs appear to be governed by the TT substituent, which remains similar between 60–66 kcal·mol^–1^, largely independent of the aryl substituent (Figure 4b). In contrast, the triplet energy of aryl halides is significantly influenced by substituents, and exhibits a stronger correlation with the HOMO–LUMO gaps of their parent arenes.

The reaction is rapid and reaches full conversion within 2 min at a catalyst loading of just 1.0 mol % and a reaction temperature of −78 °C. We propose that the geometry of the electronic overlap between excited TXO and ArTTs plays an important role: In a Dexter energy transfer process, simultaneous exchange of electrons between excited photosensitizer and substrate result in energy transfer (Figure 4c).^43^ The frontier molecular orbitals (α-HOMO and β-LUMO) of the excited TXO are predominantly situated on the carbon of the carbonyl group and the sulfur atom, respectively, while those of the ground state PhTT^+^ are primarily located on the neutral and positively charged sulfur atoms, respectively. Given the analogous tricyclic fused ring system of TXO and thianthrenium, the spatial alignment of the frontier molecular orbitals of excited TXO and ground state PhTT^+^ coincides. In view of the significant impact of orbital interactions on energy transfer rates,^43^ the interplay between thianthrenium and TXO may result in rapid energy transfer. Such a hypothesis would also provide additional insight into why arylthianthreniums react more favorably, even than those special aryl pseudohalides (Figure 2b) with appropriate triplet energy, and why other well-established photosensitizers such as benzophenone are significantly less efficient. Such fortuitous energy transfer, based on the thianthrenium structure, is yet another example of how thianthrene-chemistry can elicit desired chemical reactivity beyond what is currently possible with conventional (pseudo)halides.

### Substrate Scope

ArTTs with different substituents display comparable triplet energy between 60–65 kcal·mol^–1^, lower than the triplet energy of TXO, which suggests a broad theoretical scope of aryl electrophiles for productive and chemoselective sensitization. As shown in Table 1, a variety of ArTTs bearing ortho-, meta-, and para-substituted aryls, and electron-rich, -neutral, and -poor aryls, as well as mono-, di-, and trisubstituted aryls, are tolerated. In our prior research utilizing aryl halides as energy acceptors, only substrates featuring highly conjugated biphenyl and naphthyl frameworks are effective with visible light, and 280 nm UV-C light is necessary when employing simple aryl halides.^10^ Here, no such limitation was observed and 390 nm light could be used for all examples. Various functional groups, heteroaromatic compounds, and complex molecules are well-tolerated. Given the higher triplet energy of aryl halides compared to ArTTs, halides (**7**, **22**, **25**) remain untouched and can be potentially utilized in subsequent transformations. The large functional group tolerance may also be explained by a chemoselective Dexter energy transfer that is fastest between the TXO photosensitizer and the thianthrenium group based on their structural and electronic similarity, and leave other functional groups, potentially sensitive to energy transfer and hydrogen atom transfer, untouched. The main side products include hydrodefunctionalization, which can be reduced by further decreasing the reaction temperature to −94 °C, thereby increasing the solubility of ethylene, and the Minisci byproduct, which can be minimized by carefully controlling the reaction time or suppressed by adding a bromide nucleophile to capture the alkyl thianthrenium salt (Scheme S5).

**Table 1:**
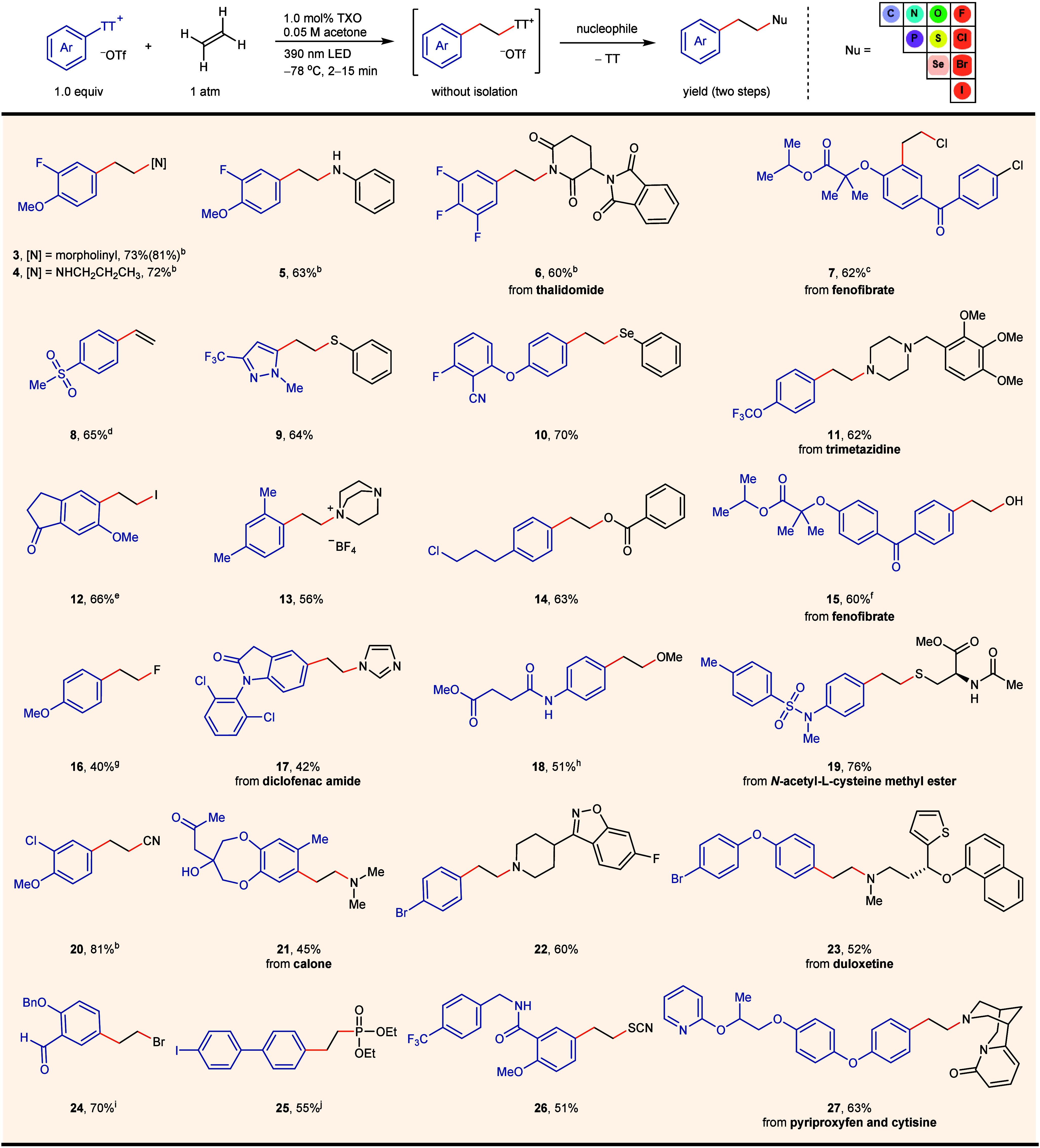
Substrate Scope in Arylation Reactions of Ethylene^a^

a Reaction conditions: arylthianthrenium salt (0.20 mmol), ethylene (1 atm), and TXO in acetone (c = 0.50 mM, 4.0 mL, 2.0 μmol, 1.0 mol %) at −78 °C under 40 W 390 nm LED for 2–15 min. Subsequently, base and nucleophile were added, and the reaction mixture was stirred for 12 h. Refer to the Supporting Information for experimental details for each substrate.

b Tetrabutylammonium bromide (TBAB, 77 mg, 0.24 mmol, 1.2 equiv) was added in the first step and allowed to react at −94 °C for 10 min.

c Tetrabutylammonium chloride (TBACl) was used.

d DBU was used.

e KI was used.

f Water was used at 55 °C.

g Triethylamine trihydrofluoride was used at 55 °C.

h Anhydrous methanol was used as solvent in the second step at 55 °C.

i NaBr was used.

The resulting primary arylethyl thianthrenium salts serve as effective alkyl electrophiles for subsequent transformations.^44,45^ By employing amines as nucleophiles in the same pot, a useful yet otherwise challenging three-component aminoarylation reaction involving aryl electrophiles, alkenes, and amines was achieved (Table 1).^46^ In previous studies with different approaches, aminoarylation of alkenes showed a wide range of alkenes, yet the scope of amines was limited to azide and nitriles.^47−49^ In our study, both the scope of amines and the tolerance toward functional groups are extensive. A wide range of amines, including cyclic and acyclic, primary, secondary, and tertiary, along with aliphatic and aromatic amines, all demonstrate excellent compatibility. A variety of valuable β-arylethylamines are synthesized, which are privileged pharmacophores found in a range of biologically active natural products and pharmaceuticals, particularly in molecules that act on the central nervous system.^47,48^ Oxygen-, sulfur-, and chloride-based nucleophiles allow for the direct synthesis of β-arylethyl ethers, thioethers, and chlorides, all of which are found in various pharmaceuticals and bioactive molecules. Nucleophiles based on carbon, phosphine, other halides, and selenium are all compatible, which demonstrates the versatile ability of neutral thianthrene to function as excellent leaving group. Chloride and bromide can be successfully added at the beginning of the reaction, whereas other nucleophiles such as iodide, aliphatic amine, carboxylate, and thiophenolate cannot, likely a consequence of their competitive reaction with the thianthrenium radical cation (Scheme S4). The wide variety of aromatic components and nucleophiles allows for fragment coupling through a −CH_2_CH_2_– linker (**27**). Nucleophiles with high basicity, such as fluoride, and carbon nucleophiles like cyanide, malonates or acetylides, resulted in low yield (<40%) because of competing elimination reactions, a side reactivity also observed with other nucleophiles except halides (Cl, Br, I), sulfur, and selenium-based ones. The elimination side reaction can be mitigated by converting alkylthianthrenium salts to alkyl bromides in situ before adding the nucleophile, as uncharged alkyl bromides are less prone to elimination. When a non-nucleophilic base, such as DBU was employed, elimination was observed exclusively, and vinylated arene (**8**) was obtained. Various alkenes beyond ethylene, such as α-olefins, styrenes, and Michael acceptors, have been tested successfully. However, the secondary or tertiary alkyl thianthrenium salts formed by the addition of ArTTs to substituted alkenes are not stable toward elimination. To retain synthetic utility, reactions with substituted olefins must be conducted in the presence of bromide to in situ displace the thianthrene leaving group to afford the corresponding alkyl bromides, which can be further functionalized (Table S1).

## Conclusions

We have demonstrated the conceptual advantage of ArTTs to enable visible light-mediated triplet energy transfer catalysis. Our study highlights the pivotal role of the two sulfur atoms in reducing the HOMO–LUMO gap and triplet energy of ArTTs. In view of the small influence of the aryl group, various ArTTs exhibit similar triplet energy. The large structural and electronic overlap between the excited TXO and thianthrenium framework facilitates rapid visible light-driven energy transfer with much higher inherent quantum yields compared to direct UV excitation.^50^ By employing ArTTs as energy acceptors, we have presented synthetically valuable arylethylation reactions of amines, alcohols and other nucleophiles. Further exploration of thianthrenium salts as acceptor for triplet energy transfer catalysis may unveil additional useful reaction chemistry.

## Supporting Information Available

Experimental procedures, spectroscopic data, and NMR spectra of all products (PDF)
