# Supplementary material for: Arylthianthrenium Salts for Triplet Energy Transfer Catalysis
Source: J Am Chem Soc. 2024 Oct 28;146(44):30474–82. doi: 10.1021/jacs.4c11099 (PMC11544621; doi:10.1021/jacs.4c11099)
Supplement: Supplementary file 1 — ja4c11099_si_001.pdf [file ja4c11099_si_001.pdf]

SUPPORTING INFORMATION

Arylthianthrenium Salts for Triplet Energy Transfer Catalysis

Yuan Cai[1], Triptesh Kumar Roy[1,2], Till J. B. Zähringer[3], Beatrice Lansbergen[1], Christoph Kerzig[3,*], and Tobias Ritter[1,2,*]

[1]Max-Planck-Institut für Kohlenforschung, Kaiser-Wilhelm-Platz 1, 45470 Mülheim an der Ruhr, Germany.

[2]Institute of Organic Chemistry, RWTH Aachen University, Landoltweg 1, 52074 Aachen, Germany

[3]Department of Chemistry, Johannes Gutenberg University Mainz, 55128 Mainz, Germany.

*ckerzig@uni-mainz.de

*ritter@kofo.mpg.de.

TABLE OF CONTENTS

TABLE OF CONTENTS 1

MATERIALS AND METHODS 6

EXPERIMENTAL DATA 8

Scope of arylthianthrenium salts and nucleophiles 8

General procedure 8

Arylethylamine **3** 9

Arylethylamine **4** 11

Arylethylamine **5** 11

Thalidomide-derived arylethylamine **6** 13

Fenofibrate-derived arylethyl chloride **7** 14

Vinyl arene **8** 15

Arylethyl thioether **9** 16

Arylethyl selenoether **10** 17

Trimetazidine-derived arylethylamine **11** 18

Arylethyl iodide **12** 19

Arylethylammonium salt **13** 20

Arylethyl benzoate **14** 21

Fenofibrate-derived 2-arylethanol **15** 22

Arylethyl fluoride **16** 23

Diclofenac amide-derived arylethylamine **17** 24

Arylethyl methyl ether **18** 25

Cysteine-derived arylethyl thioether **19** 26

Arylpropionitrile **20** 27

Calone-derived arylethylamine **21** 28

Arylethylamine **22** 29

Duloxetine-derived arylethylamine **23** 30

Arylethyl bromide **24** 31

Arylethyl phosphonate **25** 32

Arylethyl thiocyanate **26** 33

Pyriproxyfen- and cytisine-derived arylethylamine **27** 34

Scope of alkenes 35

Arylethyl bromide **28** 36

Arylethyl bromide **29** 36

Arylethyl bromide **30** 37

Arylethyl bromide **31** 38

Arylethyl bromide **32** 39
Arylethyl bromide **33** 40
Arylethyl bromide **34** 41
Arylethyl bromide **35** 42
Arylethyl bromide **36** 43

Condition optimization 44

Mechanistic studies 54

Transient absorption spectroscopy and related spectroscopic studies 54
Analysis of energy transfer rate and expected timescales 57
UV-vis absorption spectrum of reaction mixtures 58
Cyclic voltammograms 59
Relationship between reaction rates and triplet energy or redox potentials 60
Quantum yield measurement 64
Reaction in the presence of triplet quencher 66
Radical scrambling experiment 67
Hammett plot 68

DFT CALCULATIONS 70

Methods 70

Comparison with reported data 70

Visualization of frontier molecular orbitals and triplet energy of arylpseudohalides 73

HOMO/LUMO energy of arylpseudohalides 77

Visualization of frontier molecular orbitals of TXO 78

Bond dissociation energy of thianthrenium salts 79

Cartesian coordinates of the optimized species 80

SPECTROSCOPIC DATA 115

^{1}H NMR of arylethylamine **3** 115

^{13}C NMR of arylethylamine **3** 116

^{19}F NMR of arylethylamine **3** 117

^{1}H NMR of arylethylamine **4** 118

^{13}C NMR of arylethylamine **4** 119

^{19}F NMR of arylethylamine **4** 120

^{1}H NMR of arylethylamine **5** 121

^{13}C NMR of arylethylamine **5** 122

^{19}F NMR of arylethylamine **5** 123

^{1}H NMR of thalidomide-derived arylethylamine **6** 124

^{13}C NMR of thalidomide-derived arylethylamine **6** 125

^{19}F NMR of thalidomide-derived arylethylamine **6** 126

^{1}H NMR of fenofibrate-derived arylethyl chloride **7** 127

^{13}C NMR of fenofibrate-derived arylethyl chloride **7** 128

^{1}H NMR of vinyl arene **8** 129

^{13}C NMR of vinyl arene **8** 130

^{1}H NMR of arylethyl thioether **9** 131

^{13}C NMR of arylethyl thioether **9** 132

^{19}F NMR of arylethyl thioether **9** 133

^{1}H NMR of arylethyl selenoether **10** 134

^{13}C NMR of arylethyl selenoether **10** 135

^{19}F NMR of arylethyl selenoether **10** 136

^{1}H NMR of trimetazidine-derived arylethylamine **11** 137

^{13}C NMR of trimetazidine-derived arylethylamine **11** 138

^{19}F NMR of trimetazidine-derived arylethylamine **11** 139

^{1}H NMR of arylethyl iodide **12** 140

^{13}C NMR of arylethyl iodide **12** 141

^{1}H NMR of arylethylammonium salt **13** 142

^{13}C NMR of arylethylammonium salt **13** 143

^{19}F NMR of arylethylammonium salt **13** 144

^{1}H NMR of arylethyl benzoate **14** 145

^{13}C NMR of arylethyl benzoate **14** 146

^{1}H NMR of fenofibrate-derived 2-arylethanol **15** 147

^{13}C NMR of fenofibrate-derived 2-arylethanol **15** 148

^{1}H NMR of arylethyl fluoride **16** 149

^{13}C NMR of arylethyl fluoride **16** 150

^{19}F NMR of arylethyl fluoride **16** 151

^{1}H NMR of diclofenac amide-derived arylethylamine **17** 152

^{13}C NMR of diclofenac amide-derived arylethylamine **17** 153

^{1}H NMR of arylethyl methyl ether **18** 154

^{13}C NMR of arylethyl methyl ether **18** 155

^{1}H NMR of cysteine-derived arylethyl thioether **19** 156

^{13}C NMR of cysteine-derived arylethyl thioether **19** 157

^{1}H NMR of arylpropionitrile **20** 158

^{13}C NMR of arylpropionitrile **20** 159

^{1}H NMR of calone-derived arylethylamine **21** 160

^{13}C NMR of calone-derived arylethylamine **21** 161

^{1}H NMR of arylethylamine **22** 162

^{13}C NMR of arylethylamine **22** 163

^{19}F NMR of arylethylamine **22** 164

^{1}H NMR of duloxetine-derived arylethylamine **23** 165

^{13}C NMR of duloxetine-derived arylethylamine **23** 166

^{1}H NMR of arylethyl bromide **24** 167

^{13}C NMR of arylethyl bromide **24** 168

^{1}H NMR of arylethyl phosphonate **25** 169

^{13}C NMR of arylethyl phosphonate **25** 170

^{31}P NMR of arylethyl phosphonate **25** 171

^{1}H NMR of arylethyl thiocyanate **26** 172

^{13}C NMR of arylethyl thiocyanate **26** 173

^{19}F NMR of arylethyl thiocyanate **26** 174

^{1}H NMR of pyriproxyfen- and cytisine-derived arylethylamine **27** 175

^{13}C NMR of pyriproxyfen- and cytisine-derived arylethylamine **27** 176

^{1}H NMR of arylethyl bromide **28** 177

^{13}C NMR of arylethyl bromide **28** 178

^{1}H NMR of arylethyl bromide **29** 179

^{13}C NMR of arylethyl bromide **29**......180

^{1}H NMR of arylethyl bromide **30**......181

^{13}C NMR of arylethyl bromide **30**......182

^{1}H NMR of arylethyl bromide **31**......183

^{13}C NMR of arylethyl bromide **31**......184

^{1}H NMR of arylethyl bromide **32**......185

^{13}C NMR of arylethyl bromide **32**......186

^{31}P NMR of arylethyl bromide **32**......187

^{1}H NMR of arylethyl bromide **33**......188

^{13}C NMR of arylethyl bromide **33**......189

^{1}H NMR of arylethyl bromide **34**......190

^{13}C NMR of arylethyl bromide **34**......191

^{1}H NMR of arylethyl bromide **35**......192

^{13}C NMR of arylethyl bromide **35**......193

^{1}H NMR of arylethyl bromide **36**......194

^{13}C NMR of arylethyl bromide **36**......195

REFERENCES......196

MATERIALS AND METHODS

All air- and moisture-insensitive reactions were carried out under ambient atmosphere and monitored by thin-layer chromatography (TLC). Concentration under reduced pressure was performed by rotary evaporation at 25–40 °C at an appropriate pressure. Purified compounds were further dried under high vacuum (0.010–0.005 mbar). Yields refer to purified and spectroscopically pure compounds. All air- and moisture-sensitive manipulations were performed using oven-dried glassware (120 °C for a minimum of 12 hours) and standard Schlenk techniques under an atmosphere of argon.

Solvents

Anhydrous MeCN was obtained from *Phoenix Solvent Drying Systems*. Anhydrous acetone was dried by storage over MgSO4. All deuterated solvents were purchased from *Euriso-Top*.

Chromatography

Thin layer chromatography (TLC) was performed using EMD TLC plates pre-coated with 250 μm thickness silica gel 60 F254 plates and visualized by fluorescence quenching under 254 nm UV light or permanganate stain. Flash chromatography was performed using silica gel (40–63 μm particle size) purchased from Geduran®.

Spectroscopy and Instruments

NMR spectra were recorded on a *Bruker Ascend* TM 500 spectrometer operating at 500 MHz, 471 MHz and 126 MHz, for ^{1}H, ^{19}F, and ^{13}C acquisitions, respectively. Chemical shifts are reported in ppm with the solvent residual peak as the internal standard. For ^{1}H NMR: $CDCl_3$, 7.26; CD_3CN, 1.96; For ^{13}C NMR: $CDCl_3$, 77.16; CD_3CN, 1.32.[1] ^{19}F NMR spectra were referenced using a unified chemical shift scale based on the ^{1}H resonance of tetramethylsilane (1% v/v solution in the respective solvent). Data is reported as follows: s = singlet, d = doublet, t = triplet, q = quartet, quin = quintet, sext = sextet, sept = septet, m = multiplet, bs = broad singlet; coupling constants in Hz. The LP980KS setup from Edinburgh Instruments equipped with an Nd:YAG laser from Quantel (Q-Smart 450) was employed for transient absorption and time-resolved emission spectroscopy. The frequency-tripled output with a wavelength of 355 nm served as the excitation source. The laser pulse duration was ~5 ns and the pulse frequency was 10 Hz. The pulse energy used for transient absorption and emission studies was 10-30 mJ. Detection of transient absorption spectra occurred on an iCCD camera from Andor. Kinetic traces were recorded using a photomultiplier tube. The transient absorption experiments were performed at 293 K. If not stated otherwise the TA spectra were integrated over 100 ns. 77 K emission measurements were carried out with the LP980KS setup. The standard cuvette holder was replaced by a dewar that holds a 4 mm glass tube surrounded by liquid nitrogen. A 1:1 mixture of EtOH and MeOH was used as the solvent. The phosphorescence spectra in Fig. 3b (main paper) were integrated over 10 ms.

Triplet-triplet energy transfer rate constants

Triplet-triplet energy transfer rate constants using TXO as a sensitizer were determined through Stern-Volmer analyses. Unless otherwise specified, low laser intensities were employed to avoid self-quenching effects of TXO. The lifetimes were obtained by fitting the transient absorption data sets with a monoexponential decay function.

Starting materials

All substrates were used as received from commercial suppliers. Arylthianthrenium salts[2] and 2-substituted thioxanthones[3] were prepared according to published procedures. Chemicals were purchased from *Sigma-Aldrich*, *TCI*, or *Alfa Aesar*.

EXPERIMENTAL DATA

Scope of arylthianthrenium salts and nucleophiles

General procedure

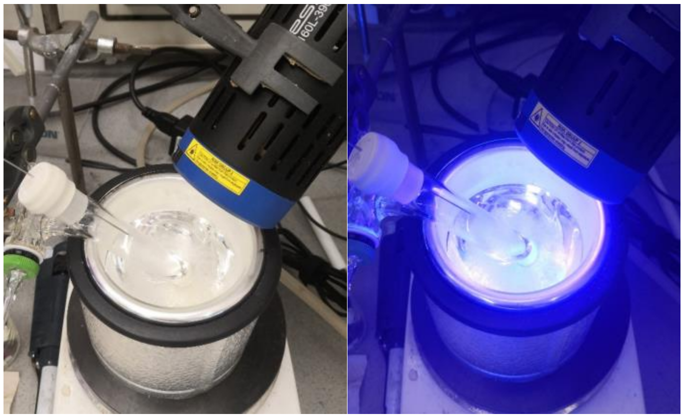

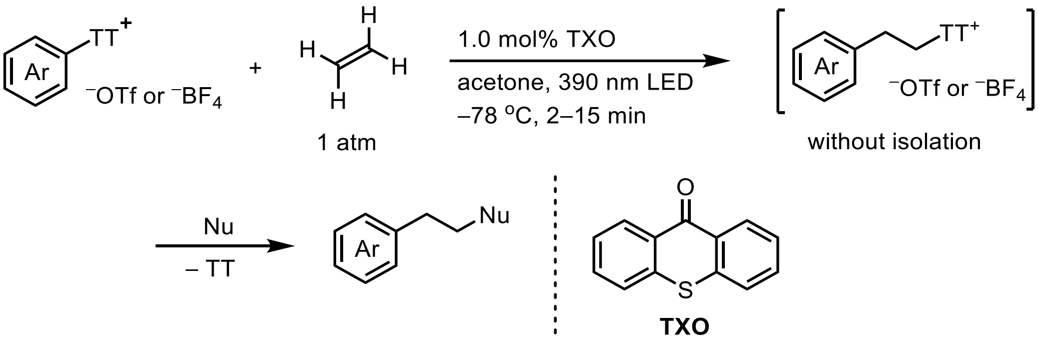

To a 5-mL Schlenk tube containing a Teflon-coated magnetic stirring bar were added arylthianthrenium salt (0.200 mmol, 1.00 equiv) and thioxanthone (TXO) in acetone (c = 0.50 mM, 4.0 mL, 2.0 μmol, 1.0 mol%). The tube was sealed with a rubber septum and immersed in a dry ice/acetone bath at –78°C. A balloon filled with ethylene gas was connected to a long needle, which was inserted to the tube through the rubber septum. Roughly 100 ml of ethylene gas was bubbled through the solution over a time period of approximately 5 minutes, and excess gas was released through the side arm, all while avoiding exposure to light. The valve of the side arm was closed, and the needle was withdrawn from the solution but remained in the headspace of the vessel. The reaction mixture was then subjected to irradiation by one Kessil PR160-390 nm LED with 100% intensity (40 W, ~6 cm away) for a period of ranging from 2 to 15 minutes, all while maintaining the bath temperature at –78°C. The progress of the reaction was monitored at 2, 5, 10, and 15 min by ^{1}H NMR spectroscopy. The complete conversion of arylthianthrenium salt was confirmed by comparing the ^{1}H NMR spectrum of the crude reaction mixture with that of pure arylthianthrenium salt, with particular attention to the disappearance of the characteristic doublet peak at around 8.6 ppm, typically occurring within 5 minutes. Then, nucleophile (0.24 mmol, 1.2 equiv) and base (0.40 mmol, 2.0 equiv) were added. The resulting mixture was stirred for 12 h without exposure to light. During this period, the reaction temperature was gradually allowed to rise to 23 ºC. In certain cases, heating and solvent changes were required as part of the process.

Subsequently, silica gel (5–10 mL) was added, and the reaction mixture was concentrated to dryness under reduced pressure. The resulting residue was purified by chromatography on silica gel to afford the desired product.

Notes:

1. Exercise caution when handling the high-pressure ethylene gas cylinder during balloon filling.
2. The purity of ArTTs is crucial for achieving high yield, as colored impurities may quench the excited photosensitizer.
3. Ensure a clear dry ice/acetone bath, as a turbid solution can diminish irradiation efficiency.
4. A clear reaction solution is also crucial for irradiation efficiency. Reactivity comparisons between **1** with $^{-}$OTf and $^{-}BF_4$ counterions didn't show obvious difference, given their comparable solubility in acetone at –78°C. However, during substrate scope investigations, it was observed that $^{-}BF_4$ counterions occasionally led to lower solubility compared to $^{-}$OTf, resulting in reduced yields. In such cases, extending the reaction time, diluting the solution, or employing acetonitrile as a solvent or cosolvent may enhance the yields. Further details on these observations have been outlined in the detailed procedure for each substrate.
5. Eliminate HCl when using amine/HCl salts as starting materials, as chloride can act as a competitive nucleophile.
6. To enhance light exposure, it is recommended to incline the tube slightly, ensuring a more effective irradiation by maximizing the surface area exposed to light.

Arylethylamine 3

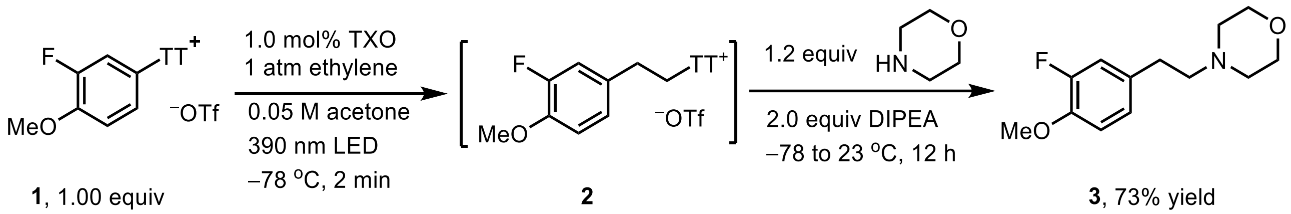

1, 1.00 equiv **2** **3**, 73% yield

To a 5-mL Schlenk tube containing a Teflon-coated magnetic stirring bar were added arylthianthrenium salt **1** (98.0 mg, 0.200 mmol, 1.00 equiv) and thioxanthone in acetone (c = 0.50 mM, 4.0 mL, 2.0 μmol, 1.0 mol%). The tube was sealed with a rubber septum and immersed in a dry ice/acetone bath at –78°C. A balloon filled with ethylene gas was connected to a long needle, which was inserted to the tube through the rubber septum. Roughly 100 ml of ethylene gas was bubbled through the solution over a time period of approximately 5 minutes, and excess gas was released through the side arm, all while avoiding exposure to light. The valve of the side arm was closed, and the needle was withdrawn from the solution but remained in the headspace of the vessel. The reaction mixture was then subjected to irradiation by one Kessil PR160-390 nm LED with 100% intensity (40 W, ~6 cm away) for 2 minutes, all while maintaining the bath temperature at –78°C. Then, morpholine (21 μL, 21 mg, 0.24 mmol, 1.2 equiv) and DIPEA (70 μL, 52 mg, 0.40 mmol, 2.0 equiv) were added. The resulting mixture was stirred for 12 h without exposure to light. During this period, the reaction

temperature was gradually allowed to rise to 23 ºC. Subsequently, silica gel (5–10 mL) was added, and the reaction mixture was concentrated to dryness under reduced pressure. The resulting residue was purified by chromatography on silica gel (EtOAc/Et_3N = 100:1) to afford **3** as a colorless liquid in 73% yield (34.9 mg).

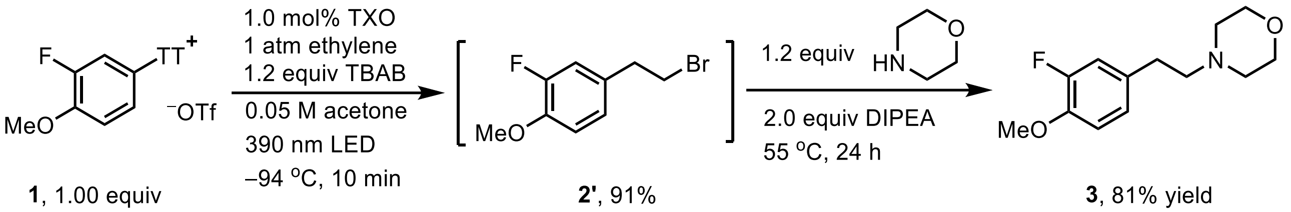

1, 1.00 equiv **2'**, 91% **3**, 81% yield

To a 5-mL Schlenk tube containing a Teflon-coated magnetic stirring bar were added arylthianthrenium salt **1** (98.0 mg, 0.200 mmol, 1.00 equiv), tetrabutylammonium bormide (TBAB, 77 mg, 0.24 mmol, 1.2 equiv), and thioxanthone in acetone (c = 0.50 mM, 4.0 mL, 2.0 μmol, 1.0 mol%). The tube was sealed with a rubber septum and immersed in a liquid nitrogen/acetone bath at –94°C. A balloon filled with ethylene gas was connected to a long needle, which was inserted to the tube through the rubber septum. Roughly 100 ml of ethylene gas was bubbled through the solution over a time period of approximately 5 minutes, and excess gas was released through the side arm, all while avoiding exposure to light. The valve of the side arm was closed, and the needle was withdrawn from the solution but remained in the headspace of the vessel. The reaction mixture was then subjected to irradiation by one Kessil PR160-390 nm LED with 100% intensity (40 W, ~6 cm away) for 10 minutes, all while maintaining the bath temperature at –94°C. Then, morpholine (21 μL, 21 mg, 0.24 mmol, 1.2 equiv) and DIPEA (70 μL, 52 mg, 0.40 mmol, 2.0 equiv) were added. The resulting mixture was stirred at 55 ºC for 24 h without exposure to light. Subsequently, silica gel (5–10 mL) was added, and the reaction mixture was concentrated to dryness under reduced pressure. The resulting residue was purified by chromatography on silica gel (EtOAc/Et_3N = 100:1) to afford **3** as a colorless liquid in 81% yield (38.7 mg).

R*f* = 0.20 (EtOAc/Et_3N = 100:1).

NMR Spectroscopy:

^{1}H NMR (500 MHz, $CDCl_3$, 23 ºC, δ): 6.96 (dd, *J* = 12.3, 1.8 Hz, 1H), 6.93 – 6.86 (m, 2H), 3.87 (s, 3H), 3.75 (t, *J* = 4.6 Hz, 4H), 2.74 (dd, *J* = 9.8, 6.3 Hz, 2H), 2.60 – 2.54 (m, 2H), 2.52 (t, *J* = 4.5 Hz, 4H).

^{13}C NMR (125 MHz, $CDCl_3$, 23 ºC, δ): 152.2 (d, *J* = 245.5 Hz), 145.9 (d, *J* = 10.7 Hz), 133.2 (d, *J* = 6.0 Hz), 124.1 (d, *J* = 3.5 Hz), 116.3 (d, *J* = 18.0 Hz), 113.5 (d, *J* = 2.3 Hz), 67.0, 60.6, 56.3, 53.7, 32.3.

^{19}F NMR (471 MHz, $CDCl_3$, 23 ºC, δ): –135.55 (dd, *J* = 12.2, 7.7 Hz).

HRMS-ESIpos (m/z) calc'd for $C_{13}H_{19}FNO_2$ $[M+H]^+$, 240.1394; found, 240.1394; deviation: –0.1 ppm.

Arylethylamine 4

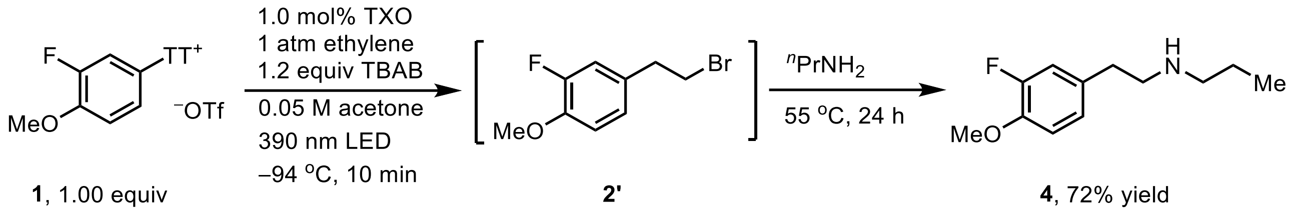

To a 5-mL Schlenk tube containing a Teflon-coated magnetic stirring bar were added arylthianthrenium salt **1** (98.0 mg, 0.200 mmol, 1.00 equiv), tetrabutylammonium bormide (TBAB, 77 mg, 0.24 mmol, 1.2 equiv), and thioxanthone in acetone (c = 0.50 mM, 4.0 mL, 2.0 μmol, 1.0 mol%). The tube was sealed with a rubber septum and immersed in a liquid nitrogen/acetone bath at –94°C. A balloon filled with ethylene gas was connected to a long needle, which was inserted to the tube through the rubber septum. Roughly 100 ml of ethylene gas was bubbled through the solution over a time period of approximately 5 minutes, and excess gas was released through the side arm, all while avoiding exposure to light. The valve of the side arm was closed, and the needle was withdrawn from the solution but remained in the headspace of the vessel. The reaction mixture was then subjected to irradiation by one Kessil PR160-390 nm LED with 100% intensity (40 W, ~6 cm away) for 10 minutes, all while maintaining the bath temperature at –94°C. Then, propylamine (0.5 mL) was added and the resulting mixture was stirred at 55 °C for 24 h without exposure to light. Subsequently, silica gel (5–10 mL) was added, and the reaction mixture was concentrated to dryness under reduced pressure. The resulting residue was purified by chromatography on silica gel (EtOAc/Et_3N = 100:1) to afford **4** as a colorless liquid in 72% yield (30.4 mg).

R_f = 0.20 (EtOAc/Et_3N = 100:1).

NMR Spectroscopy:

^{1}H NMR (500 MHz, $CDCl_3$, 23 °C, δ): 7.03 – 6.84 (m, 3H), 3.88 (s, 3H), 2.85 (td, *J* = 7.1, 1.0 Hz, 2H), 2.75 (t, *J* = 7.3 Hz, 2H), 2.66 – 2.53 (m, 2H), 1.63 – 1.39 (m, 2H), 0.91 (t, *J* = 7.4 Hz, 3H).

^{13}C NMR (125 MHz, $CDCl_3$, 23 °C, δ): 152.3 (d, *J* = 245.4 Hz), 145.9 (d, *J* = 10.7 Hz), 133.3, 124.2 (d, *J* = 3.4 Hz), 116.3 (d, *J* = 17.9 Hz), 113.5 (d, *J* = 2.2 Hz), 56.4, 51.8, 51.0, 35.5, 23.2, 11.8.

^{19}F NMR (471 MHz, $CDCl_3$, 23 °C, δ): –135.55.

HRMS-ESIpos (m/z) calc'd for $C_{12}H_{19}FNO$ $[M+H]^+$, 212.1445 found, 212.1448 deviation: –1.3 ppm.

Arylethylamine 5

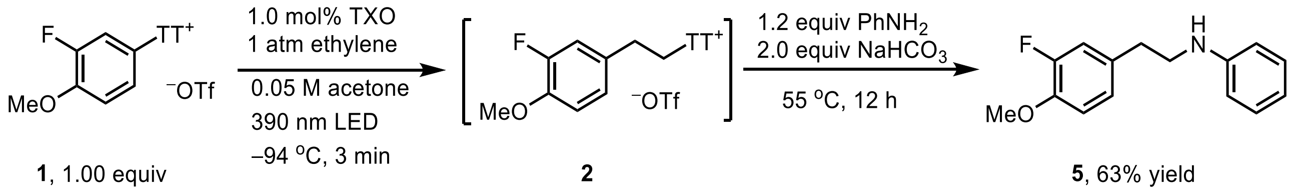

To a 5-mL Schlenk tube containing a Teflon-coated magnetic stirring bar were added arylthianthrenium salt **1** (98.0 mg, 0.200 mmol, 1.00 equiv) and thioxanthone in acetone (c = 0.50 mM, 4.0 mL, 2.0 μmol, 1.0 mol%). The tube was sealed with a rubber septum and immersed in a liquid nitrogen/acetone bath at –94°C. A balloon filled with ethylene gas was connected to a long needle, which was inserted to the tube through the rubber septum. Roughly 100 ml of ethylene gas was bubbled through the solution over a time period of approximately 5 minutes, and excess gas was released through the side arm, all while avoiding exposure to light. The valve of the side arm was closed, and the needle was withdrawn from the solution but remained in the headspace of the vessel. The reaction mixture was then subjected to irradiation by one Kessil PR160-390 nm LED with 100% intensity (40 W, ~6 cm away) for 3 minutes, all while maintaining the bath temperature at –94°C. Then, aniline (23 μL, 23 mg, 0.24 mmol, 1.2 equiv) and $NaHCO_3$ (34 mg, 0.40 mmol, 2.0 equiv) were added. The resulting mixture was stirred at 55 ºC for 12 h without exposure to light. Subsequently, silica gel (5–10 mL) was added, and the reaction mixture was concentrated to dryness under reduced pressure. The resulting residue was purified by chromatography on silica gel (hexanes/EtOAc = 10:1) to afford **5** as a colorless liquid in 63% yield (30.9 mg).

R_f = 0.22 (hexanes/EtOAc = 10:1).

NMR Spectroscopy:

^{1}H NMR (500 MHz, $CDCl_3$, 23 ºC, δ): 7.21 (dd, *J* = 8.6, 7.3 Hz, 2H), 7.02 – 6.89 (m, 4H), 6.75 (t, *J* = 7.3 Hz, 1H), 6.64 (d, *J* = 7.5 Hz, 1H), 3.91 (s, 4H), 3.40 (t, *J* = 7.0 Hz, 2H), 2.88 (t, *J* = 7.0 Hz, 2H).

^{13}C NMR (125 MHz, $CDCl_3$, 23 ºC, δ): 152.4 (d, *J* = 246.0 Hz), 147.9, 146.2 (d, *J* = 10.7 Hz), 132.4 (d, *J* = 6.0 Hz), 129.3, 124.3 (d, *J* = 3.4 Hz), 117.6, 116.4 (d, *J* = 17.9 Hz), 113.6 (d, *J* = 2.3 Hz), 113.0, 56.4, 45.0, 34.6.

^{19}F NMR (471 MHz, $CDCl_3$, 23 ºC, δ): –135.16 (dd, *J* = 12.1, 6.7 Hz).

HRMS-EI (m/z) calc'd for $C_{15}H_{16}FNO$ $[M]^+$, 245.1210; found, 245.1211; deviation: –0.3 ppm.

Thalidomide-derived arylethylamine 6

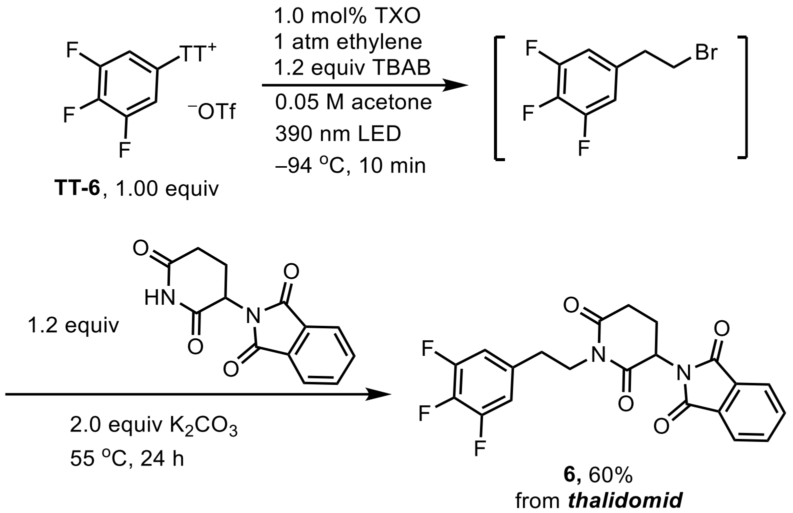

To a 5-mL Schlenk tube containing a Teflon-coated magnetic stirring bar were added **TT-6** (99.2 mg, 0.200 mmol, 1.00 equiv), tetrabutylammonium bormide (TBAB, 77 mg, 0.24 mmol, 1.2 equiv), and thioxanthone in acetone (c = 0.50 mM, 4.0 mL, 2.0 μmol, 1.0 mol%). The tube was sealed with a rubber septum and immersed in a liquid nitrogen/acetone bath at –94°C. A balloon filled with ethylene gas was connected to a long needle, which was inserted to the tube through the rubber septum. Roughly 100 ml of ethylene gas was bubbled through the solution over a time period of approximately 5 minutes, and excess gas was released through the side arm, all while avoiding exposure to light. The valve of the side arm was closed, and the needle was withdrawn from the solution but remained in the headspace of the vessel. The reaction mixture was then subjected to irradiation by one Kessil PR160-390 nm LED with 100% intensity (40 W, ~6 cm away) for 10 minutes, all while maintaining the bath temperature at –94°C. Then, thalidomide (62 mg, 0.24 mmol, 1.2 equiv) and K_2CO_3 (55 mg, 0.40 mmol, 2.0 equiv) were added. The resulting mixture was stirred at 55 ºC for 24 h without exposure to light. Subsequently, silica gel (5–10 mL) was added, and the reaction mixture was concentrated to dryness under reduced pressure. The resulting residue was purified by chromatography on silica gel (hexanes/EtOAc = 3:1) to afford **6** as a colorless liquid in 60% yield (49.9 mg).

R*f* = 0.18 (hexanes/EtOAc = 3:1).

NMR Spectroscopy:

^{1}H NMR (500 MHz, $CDCl_3$, 23 ºC, δ): 7.91 (dd, *J* = 5.5, 3.1 Hz, 2H), 7.80 (dd, *J* = 5.5, 3.1 Hz, 2H), 6.91 (dd, *J* = 8.1, 6.4 Hz, 2H), 5.13 – 4.94 (m, 1H), 4.10 – 4.01 (m, 1H), 4.02 – 3.92 (m, 1H), 2.96 (dd, *J* = 13.0, 2.8 Hz, 1H), 2.85 – 2.68 (m, 4H), 2.25 – 2.08 (m, 1H).

^{13}C NMR (125 MHz, $CDCl_3$, 23 ºC, δ): 170.6, 168.5, 167.4, 151.1 (ddd, *J* = 249.7, 9.8, 3.8 Hz), 138.6 (dt, *J* = 249.8, 15.4 Hz), 134.5, 131.7, 123.8, 113.0 (dd, *J* = 16.0, 4.9 Hz), 50.1, 41.1, 33.2, 31.8, 22.0.

^{19}F NMR (471 MHz, $CDCl_3$, 23 ºC, δ): –134.78 (dd, *J* = 20.7, 8.3 Hz, 2F), –163.55 (tt, *J* = 20.7, 6.5 Hz, 1F).

HRMS-ESIpos (m/z) calc'd for $C_{21}H_{15}F_3N_2O_4$ [M+H]$^+$, 417.1057; found, 417.1058; deviation: –0.4 ppm.

Fenofibrate-derived arylethyl chloride 7

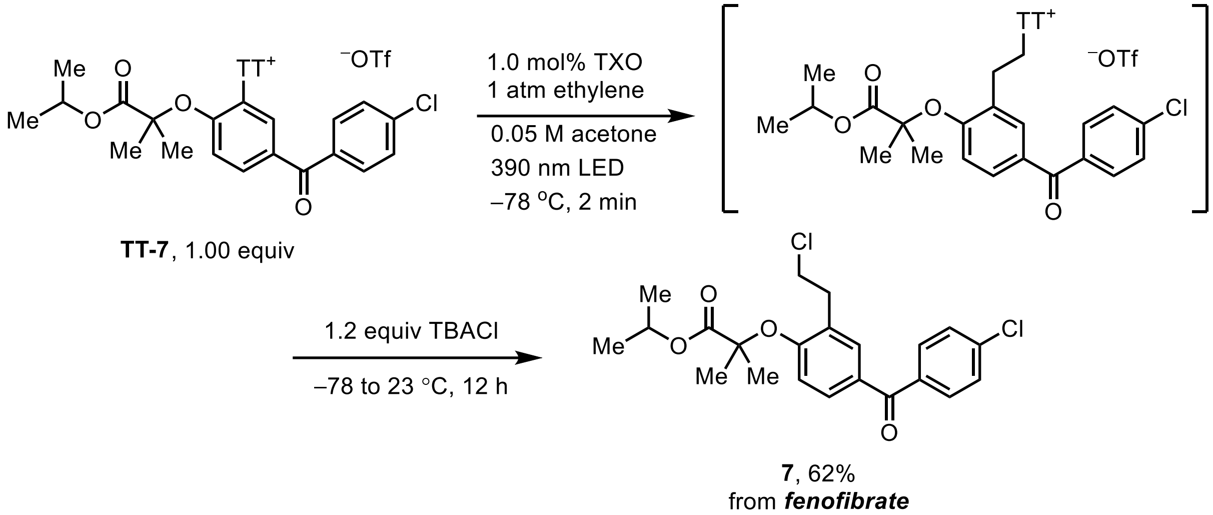

To a 5-mL Schlenk tube containing a Teflon-coated magnetic stirring bar were added **TT-7** (145 mg, 0.200 mmol, 1.00 equiv) and thioxanthone in acetone (c = 0.50 mM, 4.0 mL, 2.0 μmol, 1.0 mol%). The tube was sealed with a rubber septum and immersed in a dry ice/acetone bath at –78°C. A balloon filled with ethylene gas was connected to a long needle, which was inserted to the tube through the rubber septum. Roughly 100 ml of ethylene gas was bubbled through the solution over a time period of approximately 5 minutes, and excess gas was released through the side arm, all while avoiding exposure to light. The valve of the side arm was closed, and the needle was withdrawn from the solution but remained in the headspace of the vessel. The reaction mixture was then subjected to irradiation by one Kessil PR160-390 nm LED with 100% intensity (40 W, ~6 cm away) for 2 minutes, all while maintaining the bath temperature at –78°C. Then, tetrabutylammonium chloride (TBACl, 67 mg, 0.24 mmol, 1.2 equiv) was added. The resulting mixture was stirred for 12 h without exposure to light. During this period, the reaction temperature was gradually allowed to rise to 23 ºC. Subsequently, silica gel (5–10 mL) was added, and the reaction mixture was concentrated to dryness under reduced pressure. The resulting residue was purified by chromatography on silica gel (hexanes/EtOAc = 10:1) first and further purified by HPLC [YMC Pack Triart C18 (30×150 mm: 5 μm), 77.5:22.5 MeOH/H_2O (v/v), flow rate = 42.5 mL/min, 35 ºC] to afford **7** as a colorless liquid in 62% yield (52.4 mg).

R*f* = 0.28 (hexanes/EtOAc = 10:1).

NMR Spectroscopy:

^{1}H NMR (500 MHz, $CDCl_3$, 23 ºC, δ): 7.72 (d, *J* = 8.5 Hz, 2H), 7.70 (d, *J* = 2.2 Hz, 1H), 7.63 (dd, *J* = 8.6, 2.3 Hz, 1H), 7.47 (d, *J* = 8.5 Hz, 2H), 6.71 (d, *J* = 8.6 Hz, 1H), 5.07 (hept, *J* = 6.3 Hz, 1H), 3.79 (t, *J* = 7.1 Hz, 2H), 3.15 (t, *J* = 7.1 Hz, 2H), 1.72 (s, 6H), 1.18 (d, *J* = 6.2 Hz, 6H).

^{13}C NMR (125 MHz, $CDCl_3$, 23 ºC, δ): 194.2, 172.8, 157.9, 138.4, 136.4, 133.5, 131.2, 130.5, 129.8,

128.5, 128.0, 113.8, 79.6, 69.4, 43.4, 34.5, 25.6, 21.5.

HRMS-ESIpos (m/z) calc'd for $C_{22}H_{24}O_4Cl_2Na$ $[M+Na]^+$, 445.0944; found, 445.0941; deviation: +0.7 ppm.

Vinyl arene 8

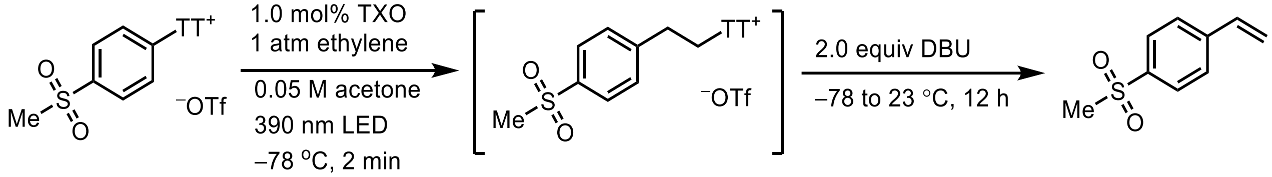

TT-8, 1.00 equiv   **8,** 65%

To a 5-mL Schlenk tube containing a Teflon-coated magnetic stirring bar were added **TT-8** (104 mg, 0.200 mmol, 1.00 equiv) and thioxanthone in acetone (c = 0.50 mM, 4.0 mL, 2.0 μmol, 1.0 mol%). The tube was sealed with a rubber septum and immersed in a dry ice/acetone bath at –78°C. A balloon filled with ethylene gas was connected to a long needle, which was inserted to the tube through the rubber septum. Roughly 100 ml of ethylene gas was bubbled through the solution over a time period of approximately 5 minutes, and excess gas was released through the side arm, all while avoiding exposure to light. The valve of the side arm was closed, and the needle was withdrawn from the solution but remained in the headspace of the vessel. The reaction mixture was then subjected to irradiation by one Kessil PR160-390 nm LED with 100% intensity (40 W, ~6 cm away) for 2 minutes, all while maintaining the bath temperature at –78°C. Then, 1,8-diazabicyclo[5.4.]undec-7-ene (DBU, 60 μL, 61 mg, 0.40 mmol, 2.0 equiv) was added. The resulting mixture was stirred for 12 h without exposure to light. During this period, the reaction temperature was gradually allowed to rise to 23 ºC. Subsequently, silica gel (5–10 mL) was added, and the reaction mixture was concentrated to dryness under reduced pressure. The resulting residue was purified by chromatography on silica gel (hexanes/EtOAc = 20:1) to afford **8** as a colorless liquid in 65% yield (23.5 mg).

R*f* = 0.30 (hexanes/EtOAc = 20:1).

NMR Spectroscopy:

^{1}**H NMR** (500 MHz, $CDCl_3$, 23 ºC, δ): 7.91 (d, *J* = 8.4 Hz, 2H), 7.60 (d, *J* = 8.5 Hz, 2H), 6.79 (dd, *J* = 17.6, 10.9 Hz, 1H), 5.93 (d, *J* = 17.6 Hz, 1H), 5.49 (d, *J* = 10.9 Hz, 1H), 3.07 (s, 3H).

^{13}**C NMR** (125 MHz, $CDCl_3$, 23 ºC, δ): 142.9, 139.3, 135.2, 127.7, 127.0, 118.0, 44.6.

HRMS-EI (m/z) calc'd for $C_9H_{10}SO_2$ $[M]^+$, 182.0396; found, 182.0399; deviation: –1.6 ppm.

Arylethyl thioether 9

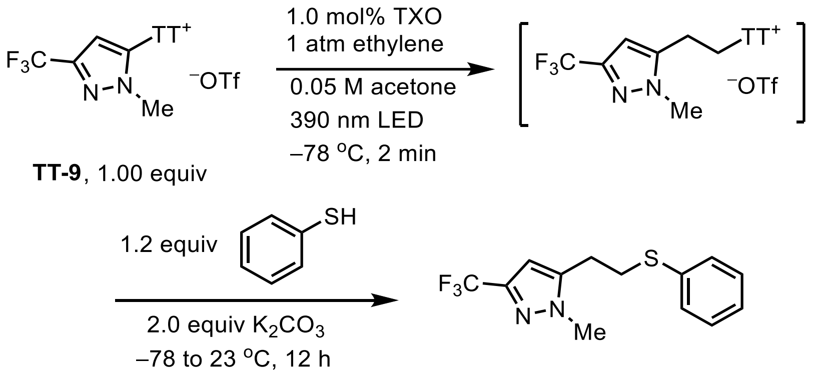

To a 5-mL Schlenk tube containing a Teflon-coated magnetic stirring bar were added **TT-9** (103 mg, 0.200 mmol, 1.00 equiv) and thioxanthone in acetone (c = 0.50 mM, 4.0 mL, 2.0 μmol, 1.0 mol%). The tube was sealed with a rubber septum and immersed in a dry ice/acetone bath at –78°C. A balloon filled with ethylene gas was connected to a long needle, which was inserted to the tube through the rubber septum. Roughly 100 ml of ethylene gas was bubbled through the solution over a time period of approximately 5 minutes, and excess gas was released through the side arm, all while avoiding exposure to light. The valve of the side arm was closed, and the needle was withdrawn from the solution but remained in the headspace of the vessel. The reaction mixture was then subjected to irradiation by one Kessil PR160-390 nm LED with 100% intensity (40 W, ~6 cm away) for 2 minutes, all while maintaining the bath temperature at –78°C. Then, thiophenol (24 μL, 26 mg, 0.24 mmol, 1.2 equiv) and K_2CO_3 (55 mg, 0.40 mmol, 2.0 equiv) were added. The resulting mixture was stirred for 12 h without exposure to light. During this period, the reaction temperature was gradually allowed to rise to 23 ºC. Subsequently, silica gel (5–10 mL) was added, and the reaction mixture was concentrated to dryness under reduced pressure. The resulting residue was purified by chromatography on silica gel (hexanes/EtOAc = 5:1) to afford **9** as a colorless liquid in 64% yield (36.5 mg).

R_f = 0.30 (hexanes/EtOAc = 5:1).

NMR Spectroscopy:

^{1}H NMR (500 MHz, $CDCl_3$, 23 ºC, δ): 7.42 – 7.36 (m, 2H), 7.37 – 7.31 (m, 2H), 7.30 – 7.23 (m, 1H), 6.37 (s, 1H), 3.79 (s, 3H), 3.29 – 3.11 (m, 2H), 2.95 (t, *J* = 7.5 Hz, 2H).

^{13}C NMR (125 MHz, $CDCl_3$, 23 ºC, δ): 142.4, 141.3 (q, *J* = 38.1 Hz), 134.9, 130.1, 129.2, 126.9, 121.3 (q, *J* = 268.3 Hz), 103.2 (q, *J* = 2.3 Hz), 36.8, 32.6, 25.8.

^{19}F NMR (471 MHz, $CDCl_3$, 23 ºC, δ): –62.07.

HRMS-ESIpos (m/z) calc'd for $C_{13}H_{13}F_3N_2NaS$ $[M+Na]^+$, 309.0644; found, 309.0643; deviation: +0.2 ppm.

Arylethyl selenoether 10

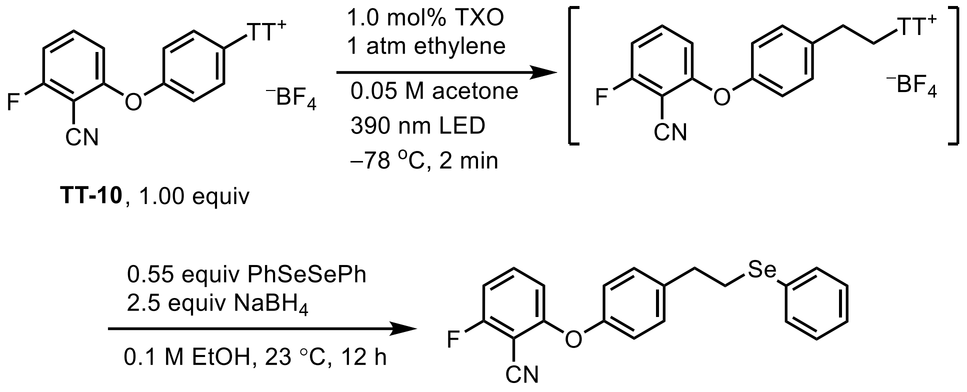

TT-10, 1.00 equiv

10, 70%

To a 5-mL Schlenk tube containing a Teflon-coated magnetic stirring bar were added **TT-10** (103 mg, 0.200 mmol, 1.00 equiv) and thioxanthone in acetone (c = 0.50 mM, 4.0 mL, 2.0 μmol, 1.0 mol%). The tube was sealed with a rubber septum and immersed in a dry ice/acetone bath at –78°C. A balloon filled with ethylene gas was connected to a long needle, which was inserted to the tube through the rubber septum. Roughly 100 ml of ethylene gas was bubbled through the solution over a time period of approximately 5 minutes, and excess gas was released through the side arm, all while avoiding exposure to light. The valve of the side arm was closed, and the needle was withdrawn from the solution but remained in the headspace of the vessel. The reaction mixture was then subjected to irradiation by one Kessil PR160-390 nm LED with 100% intensity (40 W, ~6 cm away) for 2 minutes, all while maintaining the bath temperature at –78°C. Then, the solvent was removed and the crude alkyl thianthrenium salt was directly used for the next step. To another 5-mL Schlenk tube containing a Teflon-coated magnetic stirring bar were added diphenyl diselenide (34 mg, 0.11 mmol, 0.55 equiv) and EtOH (2 mL, c = 0.1 M). Sodium borohydride (19 mg, 0.5 mmol, 2.5 equiv) was added in three portions at 0 °C. The reaction was warmed to room temperature and stirred for 1 h. The resulting mixture was added to the crude alkyl thianthrenium salt and stirred for 12 h. The resulting solution was diluted with ethyl acetate (10 mL), washed by water (10 mL) and brine (10 mL), dried over Na_2SO_4, and concentrated under reduced pressure. The resulting residue was purified by chromatography on silica gel (hexanes/EtOAc = 10:1) to afford **10** as a colorless liquid in 70% yield (55.3 mg).

R*f* = 0.14 (hexanes/EtOAc = 10:1).

NMR Spectroscopy:

^{1}H NMR (500 MHz, $CDCl_3$, 23 °C, δ): 7.53 (dd, *J* = 7.6, 1.9 Hz, 2H), 7.43 (td, *J* = 8.5, 6.4 Hz, 1H), 7.34 – 7.26 (m, 3H), 7.28 – 7.22 (m, 2H), 7.05 (d, *J* = 8.5 Hz, 2H), 6.89 (t, *J* = 8.3 Hz, 1H), 6.60 (d, *J* = 8.6 Hz, 1H), 3.26 – 3.14 (m, 2H), 3.04 (t, *J* = 7.8 Hz, 2H).

^{13}C NMR (125 MHz, $CDCl_3$, 23 °C, δ): 164.1 (d, *J* = 259.4 Hz), 161.2 (d, *J* = 4.1 Hz), 152.9, 138.5, 134.8 (d, *J* = 10.1 Hz), 132.7, 130.2, 130.0, 129.2, 127.0, 120.5, 111.6 (d, *J* = 3.2 Hz), 111.2, 109.5 (d, *J* = 19.3 Hz), 93.5 (d, *J* = 18.2 Hz), 35.9, 28.7.

^{19}F NMR (471 MHz, $CDCl_3$, 23 ºC, δ): –104.68 – –104.96 (m).

HRMS-EI (m/z) calc'd for $C_{21}H_{16}FNOSe$ $[M]^+$, 397.0376; found, 397.0381; deviation: –1.3 ppm.

Trimetazidine-derived arylethylamine 11

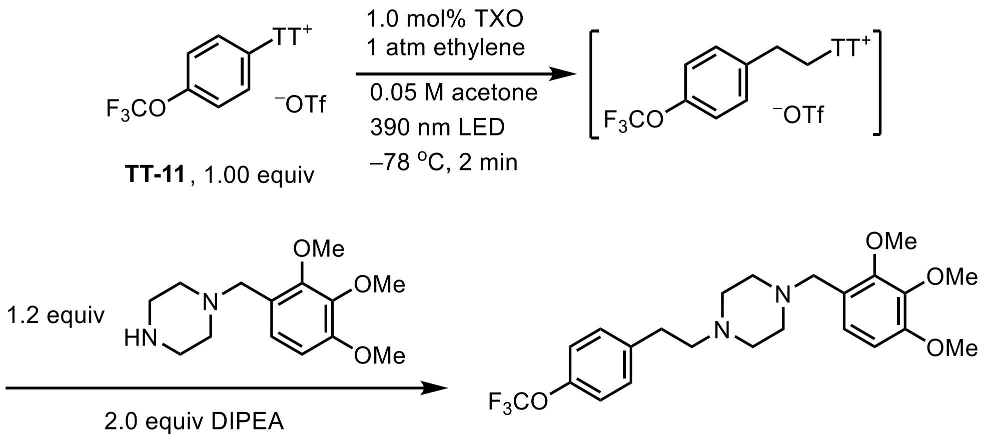

11, 62% yield
from ***trimetazidine***

To a 5-mL Schlenk tube containing a Teflon-coated magnetic stirring bar were added **TT-11** (105 mg, 0.200 mmol, 1.00 equiv) and thioxanthone in acetone (c = 0.50 mM, 4.0 mL, 2.0 μmol, 1.0 mol%). The tube was sealed with a rubber septum and immersed in a dry ice/acetone bath at –78°C. A balloon filled with ethylene gas was connected to a long needle, which was inserted to the tube through the rubber septum. Roughly 100 ml of ethylene gas was bubbled through the solution over a time period of approximately 5 minutes, and excess gas was released through the side arm, all while avoiding exposure to light. The valve of the side arm was closed, and the needle was withdrawn from the solution but remained in the headspace of the vessel. The reaction mixture was then subjected to irradiation by one Kessil PR160-390 nm LED with 100% intensity (40 W, ~6 cm away) for 2 minutes, all while maintaining the bath temperature at –78°C. Then, trimetazidine (64 mg, 0.24 mmol, 1.2 equiv) and DIPEA (70 μL, 52 mg, 0.40 mmol, 2.0 equiv) were added. The resulting mixture was stirred for 12 h without exposure to light. During this period, the reaction temperature was gradually allowed to rise to 23 ºC. Subsequently, silica gel (5–10 mL) was added, and the reaction mixture was concentrated to dryness under reduced pressure. The resulting residue was purified by chromatography on silica gel (EtOAc/Et_3N = 100:1) to afford **11** as a colorless liquid in 62% yield (56.4 mg).

R*f* = 0.30 (EtOAc/Et_3N = 100:1).

NMR Spectroscopy:

^{1}H NMR (500 MHz, $CDCl_3$, 23 ºC, δ): 7.13 (d, *J* = 8.6 Hz, 2H), 7.07 – 7.00 (m, 2H), 6.91 (d, *J* = 8.5 Hz, 1H), 6.55 (d, *J* = 8.5 Hz, 1H), 3.81 (s, 3H), 3.79 (s, 3H), 3.77 (s, 3H), 3.43 (s, 2H), 2.77 – 2.68 (m, 2H), 2.56 – 2.49 (m, 2H), 2.48 (s, 8H).

^{13}C NMR (125 MHz, $CDCl_3$, 23 ºC, δ): 152.9, 152.7, 147.5, 142.3, 139.2, 129.9, 125.2, 123.9, 120.9, 120.5 (d, *J* = 256.4 Hz), 106.9, 61.2, 60.8, 60.3, 56.5, 56.0, 53.3, 52.8, 33.0.

^{19}F NMR (471 MHz, $CDCl_3$, 23 ºC, δ): –57.94.

HRMS-ESIpos (m/z) calc'd for $C_{23}H_{30}F_3N_2O_4$ $[M+H]^+$, 455.2152; found, 455.2154; deviation: –0.5 ppm.

Arylethyl iodide 12

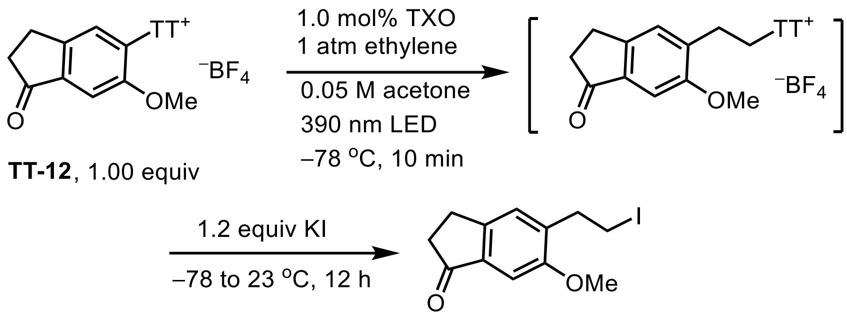

TT-12, 1.00 equiv

12, 66%

To a 5-mL Schlenk tube containing a Teflon-coated magnetic stirring bar were added **TT-12** (92.4 mg, 0.200 mmol, 1.00 equiv) and thioxanthone in acetone (c = 0.50 mM, 4.0 mL, 2.0 µmol, 1.0 mol%). The tube was sealed with a rubber septum and immersed in a dry ice/acetone bath at –78°C. A balloon filled with ethylene gas was connected to a long needle, which was inserted to the tube through the rubber septum. Roughly 100 ml of ethylene gas was bubbled through the solution over a time period of approximately 5 minutes, and excess gas was released through the side arm, all while avoiding exposure to light. The valve of the side arm was closed, and the needle was withdrawn from the solution but remained in the headspace of the vessel. The reaction mixture was then subjected to irradiation by one Kessil PR160-390 nm LED with 100% intensity (40 W, ~6 cm away) for 10 minutes, all while maintaining the bath temperature at –78°C. Then, potassium iodide (40 mg, 0.24 mmol, 1.2 equiv) was added. The resulting mixture was stirred for 12 h without exposure to light. During this period, the reaction temperature was gradually allowed to rise to 23 ºC. Subsequently, silica gel (5–10 mL) was added, and the reaction mixture was concentrated to dryness under reduced pressure. The resulting residue was purified by chromatography on silica gel (hexanes/EtOAc = 10:1) first and further purified by HPLC (YMC Triart C18 (30×150 mm: 5 µm), 60:40 to 90:10 MeCN/H_2O (v/v), flow rate = 20.0 mL/min, 35 ºC) to afford **12** as a colorless solid in 66% yield (41.6 mg).

R_f = 0.20 (hexanes/EtOAc = 10:1).

NMR Spectroscopy:

^{1}H NMR (500 MHz, $CDCl_3$, 23 ºC, δ): 7.55 (s, 1H), 6.90 (s, 1H), 3.94 (s, 3H), 3.37 (t, *J* = 7.6 Hz, 2H), 3.21 (t, *J* = 7.6 Hz, 2H), 3.14 – 3.05 (m, 2H), 2.74 – 2.62 (m, 2H).

^{13}C NMR (125 MHz, $CDCl_3$, 23 ºC, δ): 205.3, 163.1, 157.3, 130.0, 129.4, 125.2, 107.3, 55.8, 36.4, 35.2, 25.9, 4.3.

HRMS-ESIpos (m/z) calc'd for $C_{12}H_{13}INaO2$ $[M+Na]^+$, 338.9852; found, 338.9854; deviation: –0.4 ppm.

Arylethylammonium salt 13

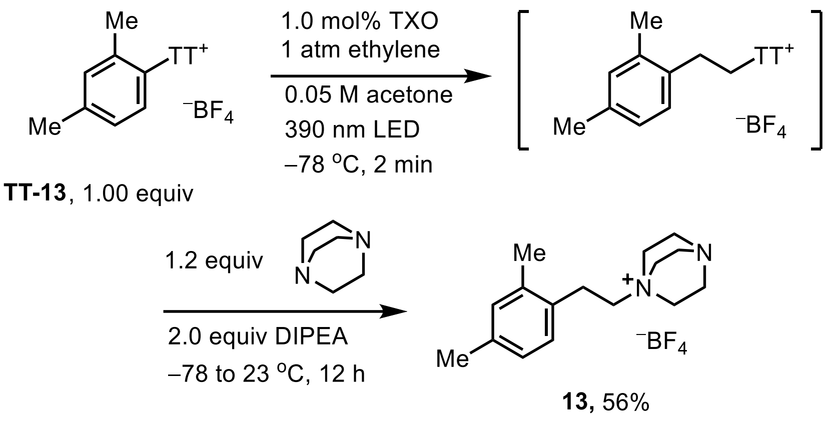

To a 5-mL Schlenk tube containing a Teflon-coated magnetic stirring bar were added **TT-13** (81.4 mg, 0.200 mmol, 1.00 equiv) and thioxanthone in acetone (c = 0.50 mM, 4.0 mL, 2.0 µmol, 1.0 mol%). The tube was sealed with a rubber septum and immersed in a dry ice/acetone bath at –78°C. A balloon filled with ethylene gas was connected to a long needle, which was inserted to the tube through the rubber septum. Roughly 100 ml of ethylene gas was bubbled through the solution over a time period of approximately 5 minutes, and excess gas was released through the side arm, all while avoiding exposure to light. The valve of the side arm was closed, and the needle was withdrawn from the solution but remained in the headspace of the vessel. The reaction mixture was then subjected to irradiation by one Kessil PR160-390 nm LED with 100% intensity (40 W, ~6 cm away) for 2 minutes, all while maintaining the bath temperature at –78°C. Then, 1,4-diazabicyclo(2.2.2)octane (DABCO, 27 mg, 0.24 mmol, 1.2 equiv) and DIPEA (70 µL, 52 mg, 0.40 mmol, 2.0 equiv) were added. The resulting mixture was stirred for 12 h without exposure to light. During this period, the reaction temperature was gradually allowed to rise to 23 ºC. Subsequently, silica gel (5–10 mL) was added, and the reaction mixture was concentrated to dryness under reduced pressure. The resulting residue was purified by chromatography on silica gel (DCM/MeOH = 10:1) to afford **13** as a colorless solid in 56% yield (37.1 mg).

R*f* = 0.26 (DCM/MeOH = 10:1).

NMR Spectroscopy:

^{1}H NMR (500 MHz, CD_3CN, 23 ºC, δ): 7.12 (d, *J* = 7.7 Hz, 1H), 7.07 (s, 1H), 7.03 (d, *J* = 7.6 Hz, 1H), 3.34 (t, *J* = 7.5 Hz, 6H), 3.30 – 3.22 (m, 2H), 3.17 (t, *J* = 7.5 Hz, 8H), 3.06 – 2.97 (m, 2H), 2.32 (s, 3H), 2.30 (s, 3H).

^{13}C NMR (125 MHz, CD_3CN, 23 ºC, δ): 137.2, 136.3, 131.3, 130.8, 129.5, 127.1, 63.8, 52.2, 44.8, 24.7, 20.0, 18.2.

^{19}F NMR (471 MHz, CD_3CN, 23 ºC, δ): –152.41, –152.46.

HRMS-ESIpos (m/z) calc'd for $C_{16}H_{25}N_2$ $[M–BF_4]^+$, 245.2012; found, 245.2013; deviation: –0.4 ppm.

Arylethyl benzoate 14

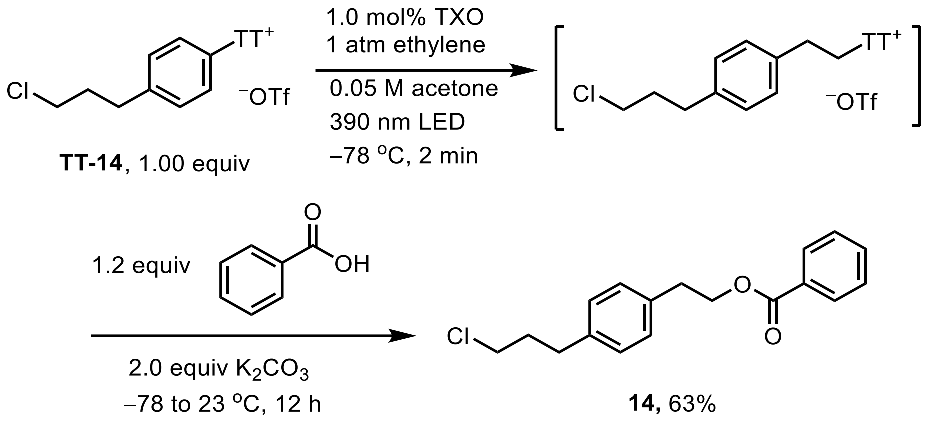

To a 5-mL Schlenk tube containing a Teflon-coated magnetic stirring bar were added **TT-14** (107 mg, 0.200 mmol, 1.00 equiv) and thioxanthone in acetone (c = 0.50 mM, 4.0 mL, 2.0 μmol, 1.0 mol%). The tube was sealed with a rubber septum and immersed in a dry ice/acetone bath at –78°C. A balloon filled with ethylene gas was connected to a long needle, which was inserted to the tube through the rubber septum. Roughly 100 ml of ethylene gas was bubbled through the solution over a time period of approximately 5 minutes, and excess gas was released through the side arm, all while avoiding exposure to light. The valve of the side arm was closed, and the needle was withdrawn from the solution but remained in the headspace of the vessel. The reaction mixture was then subjected to irradiation by one Kessil PR160-390 nm LED with 100% intensity (40 W, ~6 cm away) for 2 minutes, all while maintaining the bath temperature at –78°C. Then, benzoic acid (29 mg, 0.24 mmol, 1.2 equiv) and K_2CO_3 (55 mg, 0.40 mmol, 2.0 equiv) were added. The resulting mixture was stirred for 12 h without exposure to light. During this period, the reaction temperature was gradually allowed to rise to 23 ºC. Subsequently, silica gel (5–10 mL) was added, and the reaction mixture was concentrated to dryness under reduced pressure. The resulting residue was purified by chromatography on silica gel (hexanes/EtOAc = 20:1) to afford **14** as a colorless liquid in 63% yield (38.1 mg).

R*f* = 0.27 (hexanes/EtOAc = 20:1).

NMR Spectroscopy:

^{1}H NMR (500 MHz, $CDCl_3$, 23 ºC, δ): 8.08 – 8.03 (m, 2H), 7.63 – 7.55 (m, 1H), 7.47 (t, *J* = 7.8 Hz, 2H), 7.25 (d, *J* = 8.1 Hz, 2H), 7.19 (d, *J* = 8.0 Hz, 2H), 4.55 (t, *J* = 7.0 Hz, 2H), 3.55 (t, *J* = 6.5 Hz, 2H), 3.09 (t, *J* = 7.0 Hz, 2H), 2.79 (t, *J* = 7.4 Hz, 2H), 2.18 – 2.03 (m, 2H).

^{13}C NMR (125 MHz, $CDCl_3$, 23 ºC, δ): 166.5, 139.0, 135.7, 132.9, 130.3, 129.6, 129.1, 128.7, 128.4, 65.5, 44.2, 34.8, 34.0, 32.4.

HRMS-ESIpos (m/z) calc'd for $C_{18}H_{19}ClNaO_2$ [M+Na]$^+$, 325.0966; found, 325.0966; deviation: +0.0 ppm.

Fenofibrate-derived 2-arylethanol 15

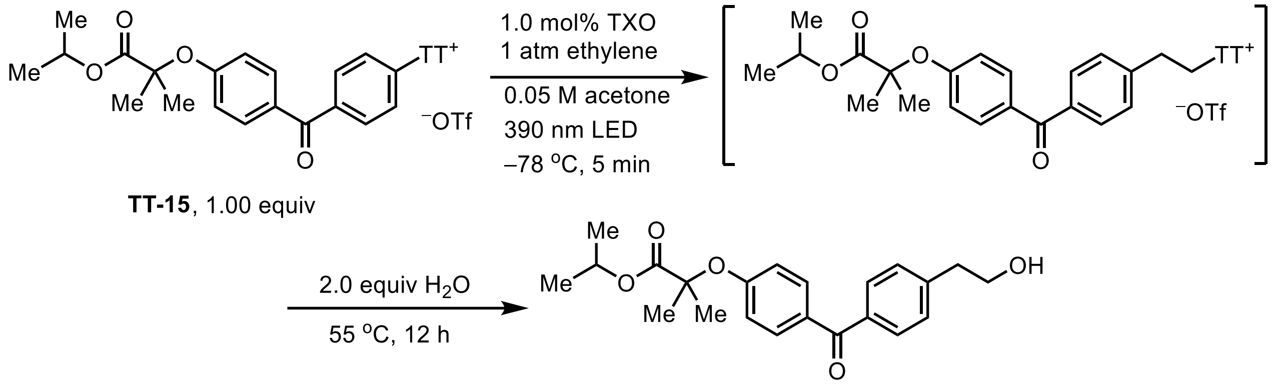

15, 60%
from ***fenofibrate***

To a 5-mL Schlenk tube containing a Teflon-coated magnetic stirring bar were added arylthianthrenium salt **1** (138 mg, 0.200 mmol, 1.00 equiv) and thioxanthone in acetone (c = 0.50 mM, 4.0 mL, 2.0 μmol, 1.0 mol%). The tube was sealed with a rubber septum and immersed in a dry ice/acetone bath at –78°C. A balloon filled with ethylene gas was connected to a long needle, which was inserted to the tube through the rubber septum. Roughly 100 ml of ethylene gas was bubbled through the solution over a time period of approximately 5 minutes, and excess gas was released through the side arm, all while avoiding exposure to light. The valve of the side arm was closed, and the needle was withdrawn from the solution but remained in the headspace of the vessel. The reaction mixture was then subjected to irradiation by one Kessil PR160-390 nm LED with 100% intensity (40 W, ~6 cm away) for 5 minutes, all while maintaining the bath temperature at –78°C. Then, water (7.2 μL, 7.2 mg, 0.40 mmol, 2.0 equiv) was added. The resulting mixture was stirred at 55 ºC for 12 h without exposure to light. Subsequently, silica gel (5–10 mL) was added, and the reaction mixture was concentrated to dryness under reduced pressure. The resulting residue was purified by chromatography on silica gel (hexanes/EtOAc = 2:1) to afford **15** as a colorless liquid in 60% yield (44.3 mg).

R*f* = 0.20 (hexanes/EtOAc = 2:1).

NMR Spectroscopy:

^{1}H NMR (500 MHz, $CDCl_3$, 23 ºC, δ): 7.77 (d, *J* = 8.9 Hz, 2H), 7.73 (d, *J* = 8.2 Hz, 2H), 7.35 (d, *J* = 8.1 Hz, 2H), 6.88 (d, *J* = 8.8 Hz, 2H), 5.10 (hept, *J* = 6.3 Hz, 1H), 3.93 (t, *J* = 6.6 Hz, 2H), 2.97 (t, *J* = 6.5 Hz, 2H), 1.22 (d, *J* = 6.3 Hz, 6H).

^{13}C NMR (125 MHz, $CDCl_3$, 23 ºC, δ): 195.3, 173.2, 159.5, 143.3, 136.4, 132.0, 130.7, 130.1, 128.9, 117.2, 79.4, 69.3, 63.3, 39.2, 25.4, 21.5.

HRMS-ESIpos (m/z) calc'd for $C_{22}H_{26}NaO_5$ $[M+Na]^+$, 393.1672; found, 393.1674; deviation: –0.5 ppm.

Arylethyl fluoride 16

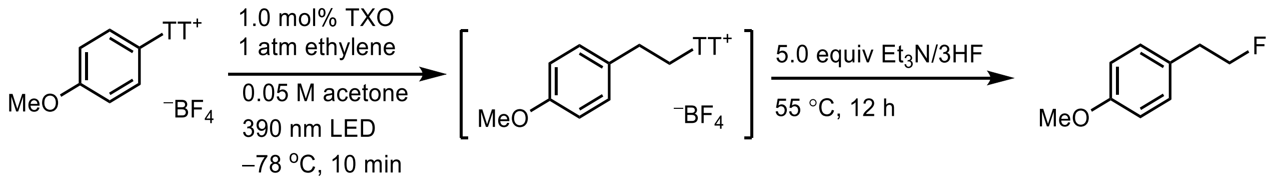

TT-16, 1.00 equiv

16, 40%

To a 5-mL Schlenk tube containing a Teflon-coated magnetic stirring bar were added **TT-16** (81.8 mg, 0.200 mmol, 1.00 equiv) and thioxanthone in acetone (c = 0.50 mM, 4.0 mL, 2.0 μmol, 1.0 mol%). The tube was sealed with a rubber septum and immersed in a dry ice/acetone bath at –78°C. A balloon filled with ethylene gas was connected to a long needle, which was inserted to the tube through the rubber septum. Roughly 100 ml of ethylene gas was bubbled through the solution over a time period of approximately 5 minutes, and excess gas was released through the side arm, all while avoiding exposure to light. The valve of the side arm was closed, and the needle was withdrawn from the solution but remained in the headspace of the vessel. The reaction mixture was then subjected to irradiation by one Kessil PR160-390 nm LED with 100% intensity (40 W, ~6 cm away) for 10 minutes, all while maintaining the bath temperature at –78°C. Then, triethylamine trihydrofluoride (163 μL, 161 mg, 1.0 mmol, 5.0 equiv) was added. The resulting mixture was stirred at 55 ºC for 12 h without exposure to light. Subsequently, silica gel (5–10 mL) was added, and the reaction mixture was concentrated to dryness under reduced pressure. The resulting residue was purified by chromatography on silica gel (hexanes/EtOAc = 20:1) to afford **16** as a colorless liquid in 40% yield (12.2 mg).

$\mathbf{R}_{\boldsymbol{f}}$ = 0.26 (hexanes/EtOAc = 20:1).

NMR Spectroscopy:

^{1}**H NMR** (500 MHz, $CDCl_3$, 23 ºC, δ): 7.18 (d, *J* = 8.6 Hz, 2H), 6.89 (d, *J* = 8.6 Hz, 2H), 4.62 (dt, *J* = 47.1, 6.7 Hz, 2H), 3.82 (s, 3H), 2.99 (dt, *J* = 22.8, 6.7 Hz, 2H).

^{13}**C NMR** (125 MHz, $CDCl_3$, 23 ºC, δ): 158.4, 129.9, 129.1 (d, *J* = 7.0 Hz), 114.0, 84.3 (d, *J* = 168.7 Hz), 55.3, 36.1 (d, *J* = 20.3 Hz).

^{19}**F NMR** (471 MHz, $CDCl_3$, 23 ºC, δ): –214.91 – –215.32 (m).

HRMS-EI (m/z) calc'd for $C_9H_{11}FO$ $[M]^+$, 154.0788; found, 154.0790; deviation: –0.8 ppm.

Diclofenac amide-derived arylethylamine 17

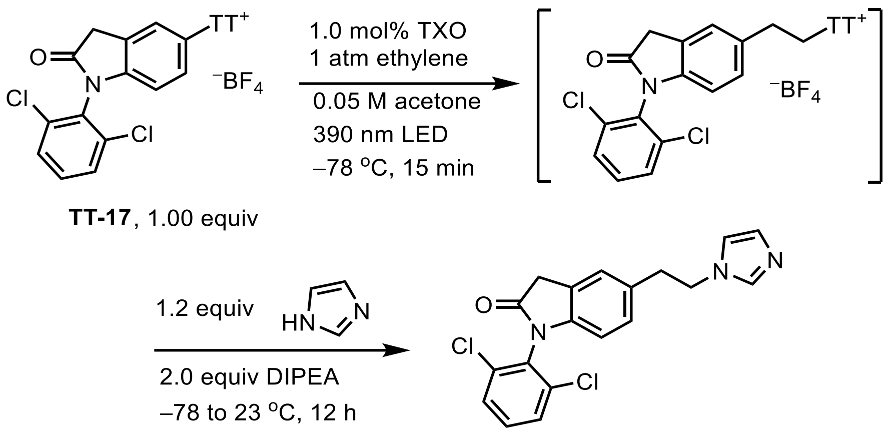

TT-17, 1.00 equiv

17, 42%
from ***diclofenac amide***

To a 5-mL Schlenk tube containing a Teflon-coated magnetic stirring bar were added **TT-17** (116 mg, 0.200 mmol, 1.00 equiv) and thioxanthone in acetone (c = 0.50 mM, 4.0 mL, 2.0 µmol, 1.0 mol%). The tube was sealed with a rubber septum and immersed in a dry ice/acetone bath at –78°C. A balloon filled with ethylene gas was connected to a long needle, which was inserted to the tube through the rubber septum. Roughly 100 ml of ethylene gas was bubbled through the solution over a time period of approximately 5 minutes, and excess gas was released through the side arm, all while avoiding exposure to light. The valve of the side arm was closed, and the needle was withdrawn from the solution but remained in the headspace of the vessel. The reaction mixture was then subjected to irradiation by one Kessil PR160-390 nm LED with 100% intensity (40 W, ~6 cm away) for 15 minutes, all while maintaining the bath temperature at –78°C. Then, imidazole (16 mg, 0.24 mmol, 1.2 equiv) and DIPEA (70 µL, 52 mg, 0.40 mmol, 2.0 equiv) were added. The resulting mixture was stirred for 12 h without exposure to light. During this period, the reaction temperature was gradually allowed to rise to 23 ºC. Subsequently, silica gel (5–10 mL) was added, and the reaction mixture was concentrated to dryness under reduced pressure. The resulting residue was purified by chromatography on silica gel (EtOAc/MeOH = 20:1) first and further purified by HPLC (Eclipse Plus C18 (21.2×150 mm: 5 µm), 60:40 to 85:15 MeOH/NH_4HCO_3 (aq.) (v/v), flow rate = 20.0 mL/min, 35 ºC) to afford **17** as a colorless liquid in 42% yield (31.3 mg).

R*f* = 0.35 (EtOAc/MeOH = 20:1).

NMR Spectroscopy:

^{1}H NMR (500 MHz, $CDCl_3$, 23 ºC, δ): 7.52 (d, *J* = 8.1 Hz, 2H), 7.46 – 7.31 (m, 2H), 7.07 (d, *J* = 8.6 Hz, 2H), 6.89 (s, 1H), 6.90 (d, *J* = 9.0 Hz, 1H), 6.34 (d, *J* = 8.0 Hz, 1H), 4.19 (t, *J* = 7.1 Hz, 2H), 3.75 (s, 2H), 3.06 (t, *J* = 7.1 Hz, 2H).

^{13}C NMR (125 MHz, $CDCl_3$, 23 ºC, δ): 173.4, 142.3, 137.1, 135.4, 132.2, 130.8, 130.4, 129.3, 129.0, 128.1, 125.2, 124.8, 118.8, 109.3, 48.7, 37.5, 35.7.

HRMS-ESIpos (m/z) calc'd for $C_{19}H_{16}Cl_2N_3O$ $[M+H]^+$, 372.0665; found, 372.0664; deviation: +0.2 ppm.

Arylethyl methyl ether 18

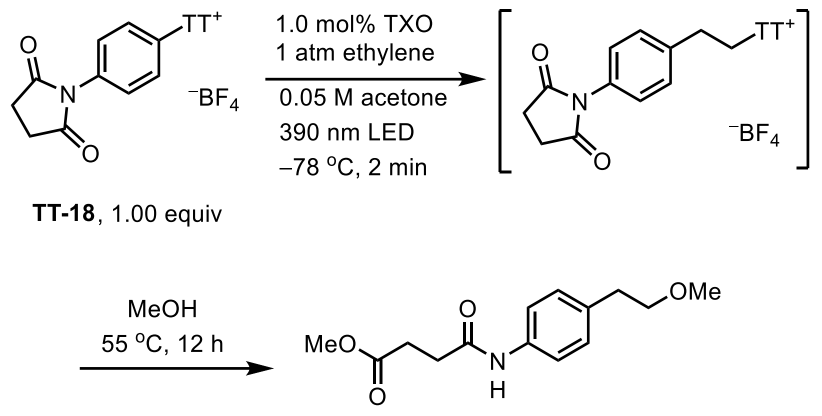

TT-18, 1.00 equiv

18, 51%

To a 5-mL Schlenk tube containing a Teflon-coated magnetic stirring bar were added **TT-18** (95.2 mg, 0.200 mmol, 1.00 equiv), thioxanthone in acetone (c = 0.50 mM, 4.0 mL, 2.0 μmol, 1.0 mol%), and acetonitrile (2 mL). The tube was sealed with a rubber septum and immersed in a dry ice/acetone bath at –78°C. A balloon filled with ethylene gas was connected to a long needle, which was inserted to the tube through the rubber septum. Roughly 100 ml of ethylene gas was bubbled through the solution over a time period of approximately 5 minutes, and excess gas was released through the side arm, all while avoiding exposure to light. The valve of the side arm was closed, and the needle was withdrawn from the solution but remained in the headspace of the vessel. The reaction mixture was then subjected to irradiation by one Kessil PR160-390 nm LED with 100% intensity (40 W, ~6 cm away) for 2 minutes, all while maintaining the bath temperature at –78°C. Then, the solvent was removed and anhydrous methanol (1 mL) was added. The resulting mixture was stirred at 55 ºC for 12 h without exposure to light. Subsequently, the reaction mixture was cooled to room temperature and diluted with ethyl acetate (10 mL). Silica gel (5–10 mL) was added, and the reaction mixture was concentrated to dryness under reduced pressure. The resulting residue was purified by chromatography on silica gel (hexanes/EtOAc = 1:1) to afford **18** as a colorless liquid in 51% yield (27.0 mg).

R_f = 0.23 (hexanes/EtOAc = 1:1).

NMR Spectroscopy:

^{1}H NMR (500 MHz, $CDCl_3$, 23 ºC, δ): 7.58 (s, 1H), 7.44 (d, *J* = 8.1 Hz, 2H), 7.18 (d, *J* = 8.1 Hz, 2H), 3.73 (s, 3H), 3.59 (t, *J* = 7.1 Hz, 2H), 3.36 (s, 3H), 2.86 (t, *J* = 7.0 Hz, 2H), 2.77 (t, *J* = 6.6 Hz, 2H), 2.67 (t, *J* = 6.6 Hz, 2H).

^{13}C NMR (125 MHz, $CDCl_3$, 23 ºC, δ): 173.6, 169.6, 136.0, 135.0, 129.3, 119.9, 73.6, 58.7, 52.0, 35.6, 32.1, 29.3.

HRMS-EI (m/z) calc'd for $C_{14}H_{19}NO_4$ $[M]^+$, 265.1309; found, 265.1311; deviation: –1.1 ppm.

Cysteine-derived arylethyl thioether 19

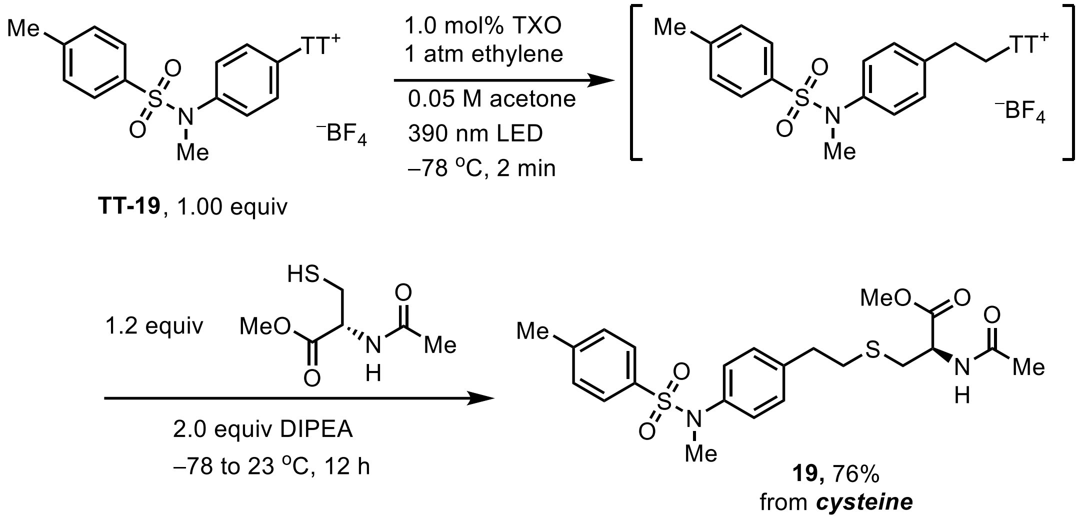

To a 5-mL Schlenk tube containing a Teflon-coated magnetic stirring bar were added **TT-19** (97.2 mg, 0.200 mmol, 1.00 equiv) and thioxanthone in acetone (c = 0.50 mM, 4.0 mL, 2.0 µmol, 1.0 mol%). The tube was sealed with a rubber septum and immersed in a dry ice/acetone bath at –78°C. A balloon filled with ethylene gas was connected to a long needle, which was inserted to the tube through the rubber septum. Roughly 100 ml of ethylene gas was bubbled through the solution over a time period of approximately 5 minutes, and excess gas was released through the side arm, all while avoiding exposure to light. The valve of the side arm was closed, and the needle was withdrawn from the solution but remained in the headspace of the vessel. The reaction mixture was then subjected to irradiation by one Kessil PR160-390 nm LED with 100% intensity (40 W, ~6 cm away) for 2 minutes, all while maintaining the bath temperature at –78°C. Then, *N*-acetyl-L-cysteine methyl ester (45 mg, 0.24 mmol, 1.2 equiv) and DIPEA (70 µL, 52 mg, 0.40 mmol, 2.0 equiv) were added. The resulting mixture was stirred for 12 h without exposure to light. During this period, the reaction temperature was gradually allowed to rise to 23 ºC. Subsequently, silica gel (5–10 mL) was added, and the reaction mixture was concentrated to dryness under reduced pressure. The resulting residue was purified by chromatography on silica gel (hexanes/EtOAc = 1:3) to afford **19** as a colorless liquid in 76% yield (70.2 mg).

R*f* = 0.16 (hexanes/EtOAc = 1:3).

NMR Spectroscopy:

^{1}H NMR (500 MHz, $CDCl_3$, 23 ºC, δ): 7.42 (d, *J* = 7.9 Hz, 2H), 7.25 (d, *J* = 8.0 Hz, 2H), 7.11 (d, *J* = 8.1 Hz, 2H), 7.01 (d, *J* = 8.3 Hz, 2H), 6.42 (d, *J* = 7.6 Hz, 1H), 4.81 (dt, *J* = 7.6, 5.2 Hz, 1H), 3.76 (s, 3H), 3.13 (s, 3H), 3.02 (dd, *J* = 13.8, 5.2 Hz, 1H), 2.95 (dd, *J* = 13.9, 5.4 Hz, 1H), 2.84 (dd, *J* = 9.0, 6.3 Hz, 2H), 2.76 (dd, *J* = 8.1, 5.6 Hz, 2H), 2.42 (s, 3H), 2.04 (s, 3H).

^{13}C NMR (125 MHz, $CDCl_3$, 23 ºC, δ): 171.3, 169.9, 143.6, 139.9, 139.2, 133.5, 129.4, 128.9, 127.9, 126.7, 52.7, 51.9, 38.1, 35.6, 34.2, 33.8, 23.1, 21.6.

HRMS-ESIpos (m/z) calc'd for $C_{22}H_{28}N_2NaO_5S_2$ $[M+Na]^+$, 487.1332; found, 487.1335; deviation: –0.7 ppm.

Arylpropionitrile 20

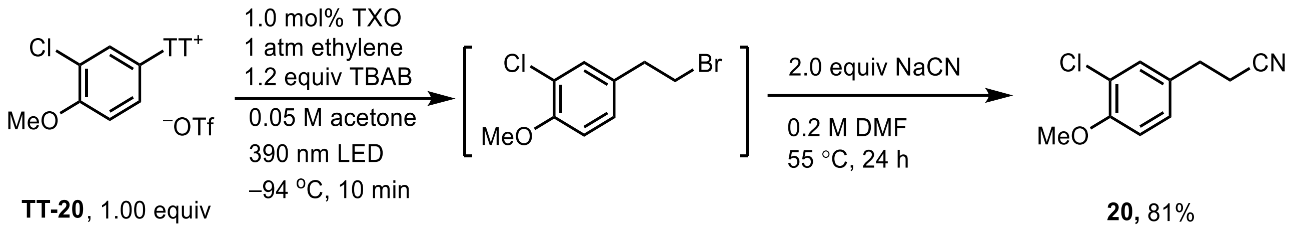

TT-20, 1.00 equiv **20,** 81%

To a 5-mL Schlenk tube containing a Teflon-coated magnetic stirring bar were added **TT-20** (101 mg, 0.200 mmol, 1.00 equiv), tetrabutylammonium bormide (TBAB, 77 mg, 0.24 mmol, 1.2 equiv), and thioxanthone in acetone (c = 0.50 mM, 4.0 mL, 2.0 μmol, 1.0 mol%). The tube was sealed with a rubber septum and immersed in a liquid nitrogen/acetone bath at –94 °C. A balloon filled with ethylene gas was connected to a long needle, which was inserted to the tube through the rubber septum. Roughly 100 ml of ethylene gas was bubbled through the solution over a time period of approximately 5 minutes, and excess gas was released through the side arm, all while avoiding exposure to light. The valve of the side arm was closed, and the needle was withdrawn from the solution but remained in the headspace of the vessel. The reaction mixture was then subjected to irradiation by one Kessil PR160-390 nm LED with 100% intensity (40 W, ~6 cm away) for 10 minutes, all while maintaining the bath temperature at –94°C. Then, the solvent was removed, and NaCN (20 mg, 0.40 mmol, 2.0 equiv) and DMF (1 mL) were added. The resulting mixture was stirred at 55 ºC for 24 h without exposure to light. The resulting solution was diluted with ethyl acetate (20 mL), washed by brine (3 × 10 mL), dried over Na_2SO_4, and concentrated under reduced pressure. The resulting residue was purified by chromatography on silica gel (hexanes/EtOAc = 5:1) to afford **20** as a colorless liquid in 81% yield (31.8 mg).

R*f* = 0.20 (hexanes/EtOAc = 5:1).

NMR Spectroscopy:

^{1}H NMR (500 MHz, $CDCl_3$, 23 ºC, δ): 7.26 (d, *J* = 2.2 Hz, 1H), 7.13 (dd, *J* = 8.4, 2.2 Hz, 1H), 6.92 (d, *J* = 8.4 Hz, 1H), 3.91 (s, 3H), 2.90 (t, *J* = 7.3 Hz, 2H), 2.61 (t, *J* = 7.4 Hz, 2H).

^{13}C NMR (125 MHz, $CDCl_3$, 23 ºC, δ): 154.2, 131.1, 130.0, 127.7, 122.7, 118.9, 112.4, 56.2, 30.5, 19.5.

HRMS-EI (m/z) calc'd for $C_{10}H_{10}ClNO$ $[M]^+$, 195.0445; found, 195.0448; deviation: –1.3 ppm.

Calone-derived arylethylamine 21

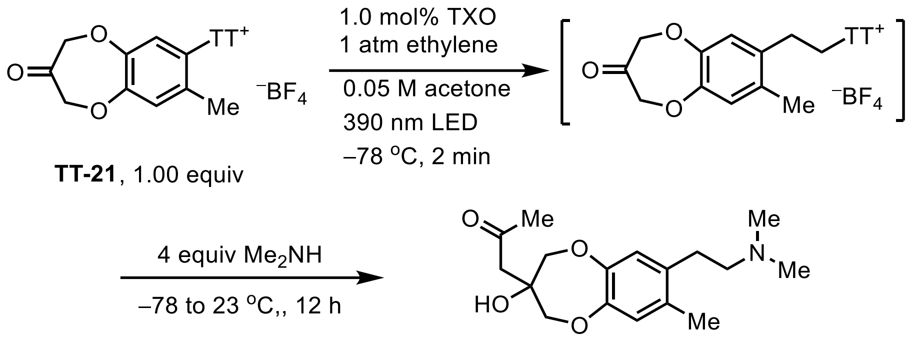

TT-21, 1.00 equiv

21, 45% yield
from ***calone***

To a 5-mL Schlenk tube containing a Teflon-coated magnetic stirring bar were added **TT-21** (95.8 mg, 0.200 mmol, 1.00 equiv) and thioxanthone in acetone (c = 0.50 mM, 4.0 mL, 2.0 μmol, 1.0 mol%). The tube was sealed with a rubber septum and immersed in a dry ice/acetone bath at –78°C. A balloon filled with ethylene gas was connected to a long needle, which was inserted to the tube through the rubber septum. Roughly 100 ml of ethylene gas was bubbled through the solution over a time period of approximately 5 minutes, and excess gas was released through the side arm, all while avoiding exposure to light. The valve of the side arm was closed, and the needle was withdrawn from the solution but remained in the headspace of the vessel. The reaction mixture was then subjected to irradiation by one Kessil PR160-390 nm LED with 100% intensity (40 W, ~6 cm away) for 2 minutes, all while maintaining the bath temperature at –78°C. Then, dimethylamine in THF (c = 2 M, 0.4 mL, 0.8 mmol, 4 equiv) was added. The resulting mixture was stirred for 12 h without exposure to light. During this period, the reaction temperature was gradually allowed to rise to 23 ºC. Subsequently, silica gel (5–10 mL) was added, and the reaction mixture was concentrated to dryness under reduced pressure. The resulting residue was purified by chromatography on silica gel (hexanes/EtOAc = 2:1) first and further purified by HPLC (YMC Pack Pro C18 (30×150 mm: 5 μm), 55:45 to 90:10 MeOH/ 20 mM NH_4HCO_3 (aq.) (v/v), flow rate = 42 mL/min, 35 ºC) to afford **21** as a colorless liquid in 45% yield (27.7 mg).

R*f* = 0.27 (hexanes/EtOAc = 2:1).

NMR Spectroscopy:

^{1}H NMR (500 MHz, $CDCl_3$, 23 ºC, δ): 6.78 (s, 1H), 6.76 (s, 1H), 4.06 (dd, *J* = 12.1, 1.3 Hz, 2H), 3.99 (dd, *J* = 12.1, 0.9 Hz, 2H), 2.89 (s, 2H), 2.72 – 2.64 (m, 2H), 2.48 – 2.42 (m, 2H), 2.32 (s, 6H), 2.28 (s, 3H), 2.23 (s, 3H).

^{13}C NMR (125 MHz, $CDCl_3$, 23 ºC, δ): 209.9, 148.2, 148.2, 133.8, 131.4, 122.5, 121.5, 77.2, 77.2, 73.4, 60.2, 46.2, 45.4, 31.8, 30.8, 18.5.

HRMS-ESIpos (m/z) calc'd for $C_{17}H_{26}NO_4$ $[M+H]^+$, 308.1856; found, 308.1857; deviation: –0.3 ppm.

Arylethylamine 22

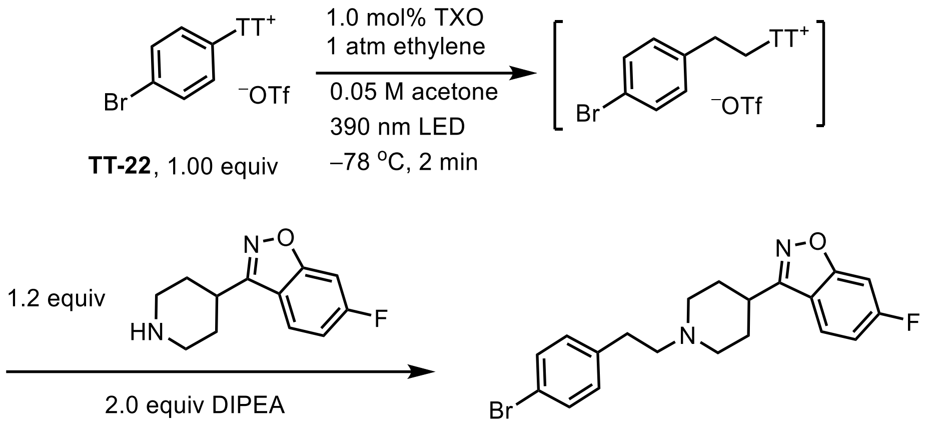

TT-22, 1.00 equiv

22, 60%

To a 5-mL Schlenk tube containing a Teflon-coated magnetic stirring bar were added **TT-22** (104 mg, 0.200 mmol, 1.00 equiv) and thioxanthone in acetone (c = 0.50 mM, 4.0 mL, 2.0 μmol, 1.0 mol%). The tube was sealed with a rubber septum and immersed in a dry ice/acetone bath at –78°C. A balloon filled with ethylene gas was connected to a long needle, which was inserted to the tube through the rubber septum. Roughly 100 ml of ethylene gas was bubbled through the solution over a time period of approximately 5 minutes, and excess gas was released through the side arm, all while avoiding exposure to light. The valve of the side arm was closed, and the needle was withdrawn from the solution but remained in the headspace of the vessel. The reaction mixture was then subjected to irradiation by one Kessil PR160-390 nm LED with 100% intensity (40 W, ~6 cm away) for 2 minutes, all while maintaining the bath temperature at –78°C. Then, 6-fluoro-3-(4-piperidinyl)-1,2-benzisoxazole (53 mg, 0.24 mmol, 1.2 equiv) and DIPEA (70 μL, 52 mg, 0.40 mmol, 2.0 equiv) were added. The resulting mixture was stirred for 12 h without exposure to light. During this period, the reaction temperature was gradually allowed to rise to 23 ºC. Subsequently, silica gel (5–10 mL) was added, and the reaction mixture was concentrated to dryness under reduced pressure. The resulting residue was purified by chromatography on silica gel (hexanes/EtOAc = 1:1) to afford **22** as a colorless liquid in 60% yield (48.1 mg).

$\mathbf{R}_f$ = 0.21 (hexanes/EtOAc = 1:1).

NMR Spectroscopy:

^{1}H NMR (500 MHz, $CDCl_3$, 23 ºC, δ): 7.73 (dd, *J* = 9.0, 5.1 Hz, 1H), 7.47 – 7.39 (m, 2H), 7.26 (d, *J* = 8.5 Hz, 1H), 7.13 (d, *J* = 7.8 Hz, 2H), 7.08 (t, *J* = 9.0 Hz, 1H), 3.15 (d, *J* = 11.9 Hz, 3H), 2.83 (t, *J* = 7.9 Hz, 2H), 2.66 (t, *J* = 8.1 Hz, 2H), 2.36 – 2.21 (m, 2H), 2.12 (d, *J* = 8.1 Hz, 4H).

^{13}C NMR (125 MHz, $CDCl_3$, 23 ºC, δ): 164.1 (d, *J* = 250.8 Hz), 163.9 (d, *J* = 13.7 Hz), 161.0, 131.5, 130.5, 122.6 (d, *J* = 11.1 Hz), 119.9, 117.3, 112.5, 112.3, 97.5 (d, *J* = 26.8 Hz), 60.4, 53.5, 34.5, 33.1, 30.5.

^{19}F NMR (471 MHz, $CDCl_3$, 23 ºC, δ): –109.59.

HRMS-ESIpos (m/z) calc'd for $C_{20}H_{21}FBrN_2O$ [M+H]$^+$, 403.0816; found, 403.0819; deviation: –0.7 ppm.

Duloxetine-derived arylethylamine 23

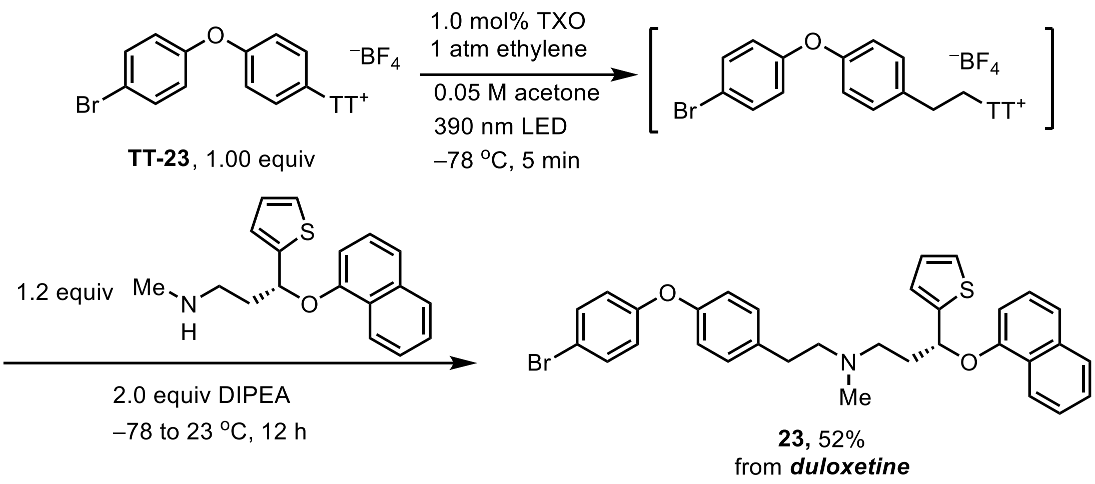

To a 5-mL Schlenk tube containing a Teflon-coated magnetic stirring bar were added **TT-23** (110 mg, 0.200 mmol, 1.00 equiv) and thioxanthone in acetone (c = 0.50 mM, 4.0 mL, 2.0 μmol, 1.0 mol%). The tube was sealed with a rubber septum and immersed in a dry ice/acetone bath at –78°C. A balloon filled with ethylene gas was connected to a long needle, which was inserted to the tube through the rubber septum. Roughly 100 ml of ethylene gas was bubbled through the solution over a time period of approximately 5 minutes, and excess gas was released through the side arm, all while avoiding exposure to light. The valve of the side arm was closed, and the needle was withdrawn from the solution but remained in the headspace of the vessel. The reaction mixture was then subjected to irradiation by one Kessil PR160-390 nm LED with 100% intensity (40 W, ~6 cm away) for 5 minutes, all while maintaining the bath temperature at –78°C. Then, duloxetine (71 mg, 0.24 mmol, 1.2 equiv) and DIPEA (70 μL, 52 mg, 0.40 mmol, 2.0 equiv) were added. The resulting mixture was stirred for 12 h without exposure to light. During this period, the reaction temperature was gradually allowed to rise to 23 ºC. Subsequently, silica gel (5–10 mL) was added, and the reaction mixture was concentrated to dryness under reduced pressure. The resulting residue was purified by chromatography on silica gel (hexanes/EtOAc/Et_3N = 75:25:1) to afford **23** as a colorless solid in 52% yield (59.2 mg).

R_f = 0.21 (hexanes/EtOAc/Et_3N = 75:25:1).

NMR Spectroscopy:

^{1}H NMR (500 MHz, $CDCl_3$, 23 ºC, δ): 8.50 – 8.34 (m, 1H), 7.93 – 7.78 (m, 1H), 7.54 – 7.48 (m, 2H), 7.41 (d, J = 8.0 Hz, 3H), 7.32 – 7.24 (m, 1H), 7.24 (d, J = 4.9 Hz, 1H), 7.13 (d, J = 6.0 Hz, 2H), 7.04 – 7.00 (m, 1H), 6.98 – 6.93 (m, 1H), 6.94 – 6.89 (m, 2H), 6.89 – 6.82 (m, 3H), 5.75 (t, J = 6.0 Hz, 1H), 2.72 (h, J = 6.1 Hz, 3H), 2.64 (t, J = 7.2 Hz, 3H), 2.46 (dt, J = 13.4, 6.7 Hz, 1H), 2.36 (d, J = 2.5 Hz, 3H), 2.22 (dt, J = 14.2, 6.9 Hz, 1H).

^{13}C NMR (125 MHz, $CDCl_3$, 23 ºC, δ): 156.9, 154.7, 153.5, 145.4, 136.1, 134.6, 132.6, 130.1, 127.5,

126.5, 126.3, 126.2, 125.8, 125.2, 124.7, 124.6, 122.2, 120.5, 120.1, 119.1, 115.3, 107.1, 74.5, 59.5, 53.6, 42.2, 36.7, 33.1.

HRMS-ESIpos (m/z) calc'd for $C_{32}H_{31}BrSNO_2$ $[M+H]^+$, 572.1254; found, 572.1262; deviation: –1.41 ppm.

Arylethyl bromide 24

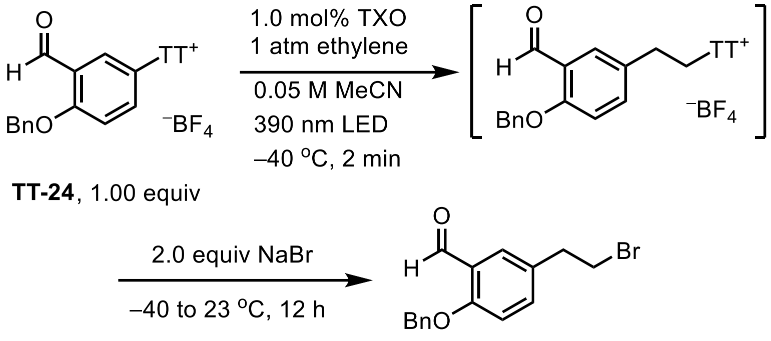

TT-24, 1.00 equiv

24, 70%

To a 5-mL Schlenk tube containing a Teflon-coated magnetic stirring bar were added **TT-24** (102 mg, 0.200 mmol, 1.00 equiv) and thioxanthone in MeCN (c = 0.50 mM, 4.0 mL, 2.0 μmol, 1.0 mol%). The tube was sealed with a rubber septum and immersed in a dry ice/MeCN bath at around –40°C. A balloon filled with ethylene gas was connected to a long needle, which was inserted to the tube through the rubber septum. Roughly 100 ml of ethylene gas was bubbled through the solution over a time period of approximately 5 minutes, and excess gas was released through the side arm, all while avoiding exposure to light. The valve of the side arm was closed, and the needle was withdrawn from the solution but remained in the headspace of the vessel. The reaction mixture was then subjected to irradiation by one Kessil PR160-390 nm LED with 100% intensity (40 W, ~6 cm away) for 2 minutes, all while maintaining the bath temperature at –40°C. Then, sodium bromide (42 mg, 0.40 mmol, 2.0 equiv) was added. The resulting mixture was stirred for 12 h without exposure to light. During this period, the reaction temperature was gradually allowed to rise to 23 ºC. Subsequently, silica gel (5–10 mL) was added, and the reaction mixture was concentrated to dryness under reduced pressure. The resulting residue was purified by chromatography on silica gel (hexanes/EtOAc = 3:1) first and further purified by HPLC (YMC Pro C18 (30×150 mm: 5 μm), 60:40 to 90:10 MeCN/H_2O (v/v), flow rate = 20.0 mL/min, 35 ºC) to afford **24** as a colorless liquid in 70% yield (44.6 mg).

R*f* = 0.18 (hexanes/EtOAc = 3:1).

NMR Spectroscopy:

^{1}H NMR (500 MHz, $CDCl_3$, 23 ºC, δ): 10.56 (s, 1H), 7.73 (d, *J* = 2.4 Hz, 1H), 7.51 – 7.33 (m, 6H), 7.04 (d, *J* = 8.5 Hz, 1H), 5.21 (s, 2H), 3.57 (t, *J* = 7.3 Hz, 2H), 3.16 (t, *J* = 7.3 Hz, 2H).

^{13}C NMR (125 MHz, $CDCl_3$, 23 ºC, δ): 189.6, 160.1, 136.3, 136.0, 131.6, 128.8, 128.3, 128.2, 127.3, 125.1, 113.3, 70.6, 38.1, 32.9.

HRMS-ESIpos (m/z) calc'd for $C_{16}H_{15}BrNaO_2$ $[M+Na]^+$, 341.0148; found, 341.0149; deviation: –0.4 ppm.

Arylethyl phosphonate 25

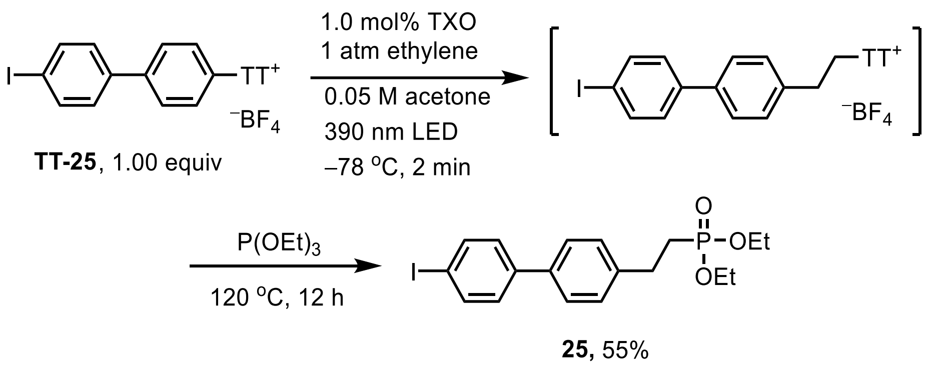

To a 5-mL Schlenk tube containing a Teflon-coated magnetic stirring bar were added **TT-25** (116 mg, 0.200 mmol, 1.00 equiv), thioxanthone in acetone (c = 0.50 mM, 4.0 mL, 2.0 μmol, 1.0 mol%), and MeCN (2 mL). The tube was sealed with a rubber septum and immersed in a dry ice/acetone bath at –78°C. A balloon filled with ethylene gas was connected to a long needle, which was inserted to the tube through the rubber septum. Roughly 100 ml of ethylene gas was bubbled through the solution over a time period of approximately 5 minutes, and excess gas was released through the side arm, all while avoiding exposure to light. The valve of the side arm was closed, and the needle was withdrawn from the solution but remained in the headspace of the vessel. The reaction mixture was then subjected to irradiation by one Kessil PR160-390 nm LED with 100% intensity (40 W, ~6 cm away) for 2 minutes, all while maintaining the bath temperature at –78°C. Then, the solvent was removed and triethyl phosphite (0.25 mL) was added. The resulting mixture was stirred at 120 ºC for 12 h without exposure to light. Subsequently, the reaction mixture was cooled down to room temperature and diluted with ethyl acetate (10 mL). Silica gel (5–10 mL) was added, and the reaction mixture was concentrated to dryness under reduced pressure. The resulting residue was purified by chromatography on silica gel (DCM/EtOAc = 1:1) first and further purified by HPLC (YMC Pack Pro C18 (30×150 mm: 5 μm), 80:20 to 95:5 MeOH/H_2O (v/v), flow rate = 42 mL/min, 35 ºC) to afford **25** as a colorless solid in 55% yield (48.9 mg).

$\mathbf{R}_f$ = 0.20 (DCM/EtOAc = 1:1).

NMR Spectroscopy:

^{1}H NMR (500 MHz, $CDCl_3$, 23 ºC, δ): 7.77 (d, J = 8.4 Hz, 2H), 7.51 (d, J = 8.1 Hz, 2H), 7.33 (d, J = 8.4 Hz, 2H), 7.30 (d, J = 8.1 Hz, 2H), 4.25 – 4.00 (m, 4H), 3.13 – 2.90 (m, 2H), 2.26 – 2.02 (m, 2H), 1.35 (t, J = 7.1 Hz, 6H).

^{13}C NMR (125 MHz, $CDCl_3$, 23 ºC, δ): 140.6 (d, J = 17.3 Hz), 140.4, 138.2, 137.8, 128.8, 128.6, 127.0, 92.9, 61.6 (d, J = 6.5 Hz), 28.3 (d, J = 4.3 Hz), 27.6 (d, J = 139.5 Hz), 16.5 (d, J = 6.0 Hz).

^{31}P NMR (203 MHz, $CDCl_3$, 23 ºC, δ): 30.6.

HRMS-EI (m/z) calc'd for $C_{18}H_{22}IPO_3$ $[M]^+$, 444.0346; found, 444.0347; deviation: –0.3 ppm.

Arylethyl thiocyanate 26

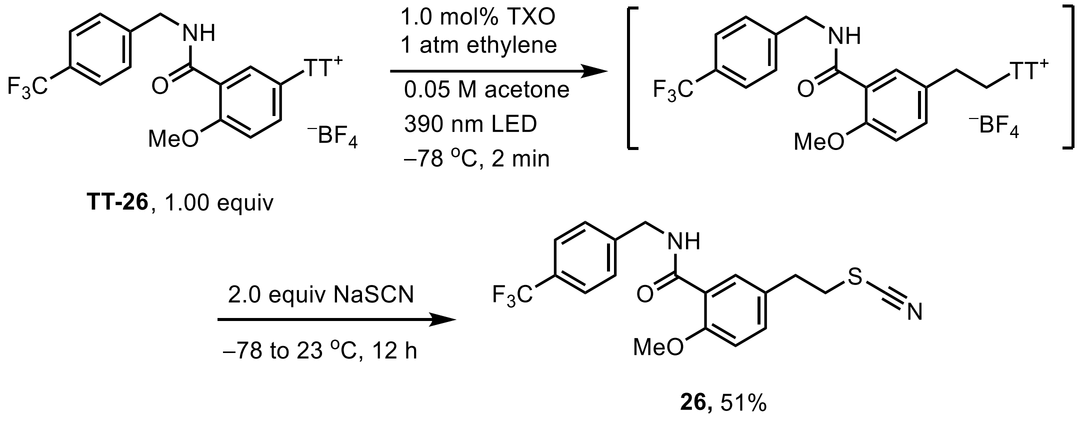

To a 5-mL Schlenk tube containing a Teflon-coated magnetic stirring bar were added **TT-26** (122 mg, 0.200 mmol, 1.00 equiv) and thioxanthone in acetone (c = 0.50 mM, 4.0 mL, 2.0 µmol, 1.0 mol%). The tube was sealed with a rubber septum and immersed in a dry ice/acetone bath at –78°C. A balloon filled with ethylene gas was connected to a long needle, which was inserted to the tube through the rubber septum. Roughly 100 ml of ethylene gas was bubbled through the solution over a time period of approximately 5 minutes, and excess gas was released through the side arm, all while avoiding exposure to light. The valve of the side arm was closed, and the needle was withdrawn from the solution but remained in the headspace of the vessel. The reaction mixture was then subjected to irradiation by one Kessil PR160-390 nm LED with 100% intensity (40 W, ~6 cm away) for 2 minutes, all while maintaining the bath temperature at –78°C. Then, sodium thiocyanate (32 mg, 0.40 mmol, 2.0 equiv) was added. The resulting mixture was stirred for 12 h without exposure to light. During this period, the reaction temperature was gradually allowed to rise to 23 ºC. Subsequently, silica gel (5–10 mL) was added, and the reaction mixture was concentrated to dryness under reduced pressure. The resulting residue was purified by chromatography on silica gel (hexanes/EtOAc = 2:1) to afford **26** as a colorless liquid in 51% yield (40.1 mg).

$\mathbf{R}_f$ = 0.19 (hexanes/EtOAc = 2:1).

NMR Spectroscopy:

^{1}**H NMR** (500 MHz, $CDCl_3$, 23 ºC, δ): 8.34 (t, *J* = 5.9 Hz, 1H), 8.12 (d, *J* = 2.5 Hz, 1H), 7.61 (d, *J* = 8.1 Hz, 2H), 7.49 (d, *J* = 8.0 Hz, 2H), 7.37 (dd, *J* = 8.4, 2.5 Hz, 1H), 7.00 (d, *J* = 8.4 Hz, 1H), 4.75 (d, *J* = 5.9 Hz, 2H), 3.98 (s, 3H), 3.23 – 3.16 (m, 2H), 3.17 – 3.10 (m, 2H).

^{13}**C NMR** (125 MHz, $CDCl_3$, 23 ºC, δ): 165.2, 156.8, 142.9, 133.6, 132.3, 130.6, 129.5 (q, *J* = 32.3 Hz), 127.7, 125.6 (q, *J* = 3.7 Hz), 124.2 (d, *J* = 271.8 Hz), 121.3, 111.9, 111.8, 56.2, 43.3, 35.1, 35.0.

^{19}**F NMR** (471 MHz, $CDCl_3$, 23 ºC, δ): –62.45.

HRMS-ESIpos (m/z) calc'd for $C_{19}H_{18}F_3SN_2O_2$ $[M+H]^+$, 395.1036; found, 395.1037; deviation: –0.3 ppm.

Pyriproxyfen- and cytisine-derived arylethylamine 27

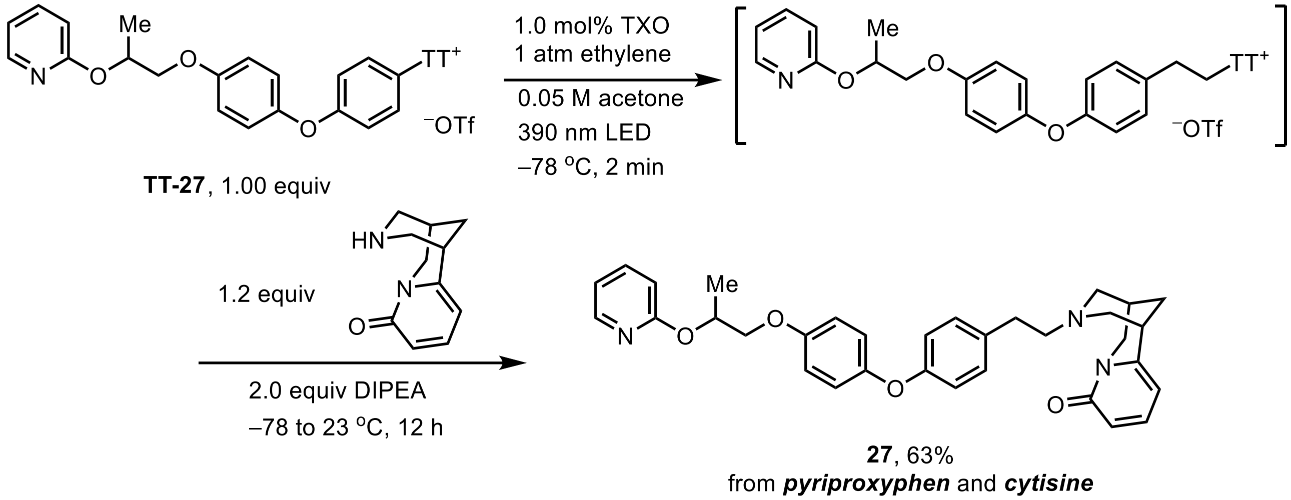

To a 5-mL Schlenk tube containing a Teflon-coated magnetic stirring bar were added **TT-27** (137 mg, 0.200 mmol, 1.00 equiv) and thioxanthone in acetone (c = 0.50 mM, 4.0 mL, 2.0 μmol, 1.0 mol%). The tube was sealed with a rubber septum and immersed in a dry ice/acetone bath at –78°C. A balloon filled with ethylene gas was connected to a long needle, which was inserted to the tube through the rubber septum. Roughly 100 ml of ethylene gas was bubbled through the solution over a time period of approximately 5 minutes, and excess gas was released through the side arm, all while avoiding exposure to light. The valve of the side arm was closed, and the needle was withdrawn from the solution but remained in the headspace of the vessel. The reaction mixture was then subjected to irradiation by one Kessil PR160-390 nm LED with 100% intensity (40 W, ~6 cm away) for 2 minutes, all while maintaining the bath temperature at –78°C. Then, cytisine (46 mg, 0.24 mmol, 1.2 equiv) and DIPEA (70 μL, 52 mg, 0.40 mmol, 2.0 equiv) were added. The resulting mixture was stirred for 12 h without exposure to light. During this period, the reaction temperature was gradually allowed to rise to 23 ºC. Subsequently, silica gel (5–10 mL) was added, and the reaction mixture was concentrated to dryness under reduced pressure. The resulting residue was purified by chromatography on silica gel (pure EtOAc) to afford **27** as a colorless solid in 63% yield (67.6 mg).

R*f* = 0.20 (pure EtOAc).

NMR Spectroscopy:

^{1}H NMR (500 MHz, CD_3CN, 23 ºC, δ): 8.16 (ddd, *J* = 5.0, 2.1, 0.8 Hz, 1H), 7.66 (ddd, *J* = 8.4, 7.1, 2.0 Hz, 1H), 7.30 (dd, *J* = 9.0, 6.9 Hz, 1H), 6.98 – 6.89 (m, 7H), 6.74 (d, *J* = 8.3 Hz, 1H), 6.70 (d, *J* = 8.6 Hz, 2H), 6.25 (dd, *J* = 9.0, 1.3 Hz, 1H), 6.07 (dd, *J* = 6.9, 1.4 Hz, 1H), 5.63 – 5.52 (m, 1H), 4.18 (dd, *J* = 10.3, 6.0 Hz, 1H), 4.12 (dd, *J* = 10.3, 4.1 Hz, 1H), 3.83 (d, *J* = 15.3 Hz, 1H), 3.77 (ddd, *J* = 15.4, 6.4, 1.0 Hz, 1H), 2.97 (d, *J* = 27.4 Hz, 1H), 2.56 (d, *J* = 6.8 Hz, 1H), 2.44 (d, *J* = 23.7 Hz, 1H), 2.33 (t, *J* = 13.0 Hz, 1H), 1.88 – 1.80 (m, 1H), 1.79 – 1.74 (m, 1H), 1.42 (d, *J* = 6.4 Hz, 3H).

^{13}C NMR (125 MHz, CD_3CN, 23 ºC, δ): 163.2, 163.1, 156.1, 155.0, 152.5, 150.7, 146.9, 139.1, 139.0, 135.3, 129.9, 120.4, 116.9, 115.7, 115.6, 111.2, 104.6, 71.0, 69.3, 60.2, 59.9, 59.2, 49.9, 35.3, 31.5,

28.0, 25.4, 16.0.

HRMS-ESIpos (m/z) calc'd for $C_{33}H_{36}N_3O_4$ $[M+H]^+$, 538.2700; found, 538.2702; deviation: –0.3 ppm.

Scope of alkenes

We have tested various alkenes beyond ethylene, including α-olefins, styrenes, and Michael acceptors (Table S1). All of these substrates exhibit desired reactivity. The yield is lower than with ethylene itself, likely due to competitive elimination of the secondary or tertiary alkyl thianthrenium salts formed by the addition of ArTTs to substituted alkenes. However, substituted alkenes can be utilized in the presence of bromide nucleophiles to afford alkyl bromides in 53–81% yields. Unactivated monosubstituted alkenes, such as propene, can be applied and potentially used to synthesize drug-related amphetamine candidates via amination. 1,1- and 1,2-Disubstituted alkenes like 2-methylpentene, 2-methylacrylate, and fumarate also work well, providing secondary and tertiary alkyl bromides that otherwise difficult to synthesize from other methods. Styrene and Michael acceptors, such as acrylonitrile, acrylamide, vinyl sulfone, and vinyl phosphonate, also perform well, yielding activated alkyl bromides suitable for further transformations.

Table S1 Substrate scope of alkenes

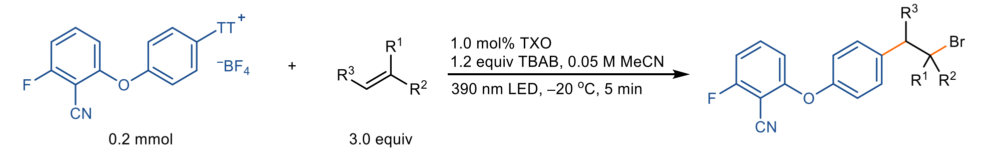

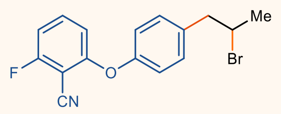

28, 80%

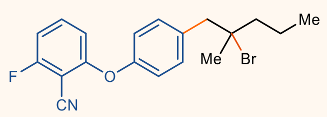

29, 65%[b]

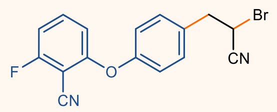

30, 78%

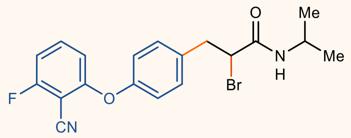

31, 63%

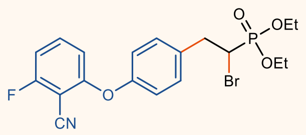

32, 61%

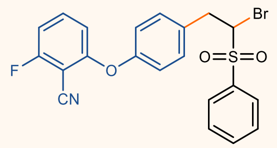

33, 81%

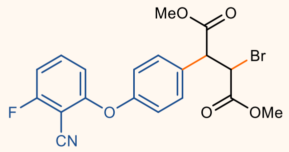

34, 53%

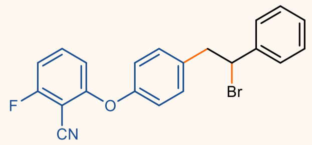

35, 75%[c]

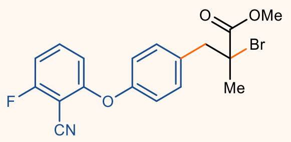

36, 79%

[a]Reaction conditions: arylthianthrenium salt (0.20 mmol), alkenes (0.60 mmol, 3.0 equiv), TBAB (77 mg, 0.24 mmol, 1.2 equiv), and TXO in MeCN (c = 0.50 mM, 4.0 mL, 2.0 μmol, 1.0 mol%) at –20 °C under 40 W 390 nm LED for 5 min. [b]10 equiv alkene used. [c]15 min.

Arylethyl bromide 28

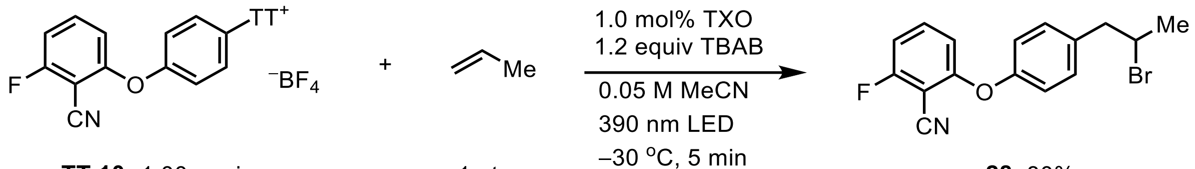

TT-10, 1.00 equiv 1 atm **28**, 80%

To a 5-mL Schlenk tube containing a Teflon-coated magnetic stirring bar were added **TT-10** (103 mg, 0.200 mmol, 1.00 equiv), tetrabutylammonium bormide (TBAB, 77 mg, 0.24 mmol, 1.2 equiv), and thioxanthone in acetonitrile (c = 0.50 mM, 4.0 mL, 2.0 μmol, 1.0 mol%). The tube was sealed with a rubber septum and immersed in a methanol/water (40/60) dry ice bath at –30°C. A balloon filled with propylene gas was connected to a long needle, which was inserted to the tube through the rubber septum. Roughly 100 ml of propylene gas was bubbled through the solution over a time period of approximately 5 minutes, and excess gas was released through the side arm, all while avoiding exposure to light. The valve of the side arm was closed, and the needle was withdrawn from the solution but remained in the headspace of the vessel. The reaction mixture was then subjected to irradiation by one Kessil PR160-390 nm LED with 100% intensity (40 W, ~6 cm away) for 5 minutes, all while maintaining the bath temperature at –30°C. Then, the reaction temperature was allowed to rise to 23 ºC. Silica gel (5–10 mL) was added, and the reaction mixture was concentrated to dryness under reduced pressure. The resulting residue was purified by chromatography on silica gel (hexanes/EtOAc = 10:1) to afford **28** as a colorless liquid in 80% yield (53.4 mg).

R*f* = 0.21 (hexanes/EtOAc = 10:1).

NMR Spectroscopy:

^{1}H NMR (500 MHz, $CDCl_3$, 23 ºC, δ): 7.45 (td, *J* = 8.5, 6.4 Hz, 1H), 7.29 (d, *J* = 8.5 Hz, 2H), 7.08 (d, *J* = 8.5 Hz, 2H), 6.90 (td, *J* = 8.4, 0.8 Hz, 1H), 6.64 (dt, *J* = 8.7, 0.9 Hz, 1H), 4.32 (h, *J* = 6.7 Hz, 1H), 3.22 (dd, *J* = 14.2, 7.5 Hz, 1H), 3.13 (dd, *J* = 14.2, 6.6 Hz, 1H), 1.75 (d, *J* = 6.7 Hz, 3H).

^{13}C NMR (125 MHz, $CDCl_3$, 23 ºC, δ): 164.1 (d, *J* = 259.7 Hz), 161.1 (d, *J* = 4.1 Hz), 153.3, 136.0, 134.9 (d, *J* = 10.2 Hz), 131.0, 120.3, 111.8 (d, *J* = 3.5 Hz), 111.1, 109.6 (d, *J* = 19.5 Hz), 93.6 (d, *J* = 18.0 Hz), 50.3, 46.6, 25.8.

HRMS-ESIpos (m/z) calc'd for $C_{16}H_{13}FBrNONa$ $[M+Na]^+$, 356.0057; found, 356.0055; deviation: +0.6 ppm.

Arylethyl bromide 29

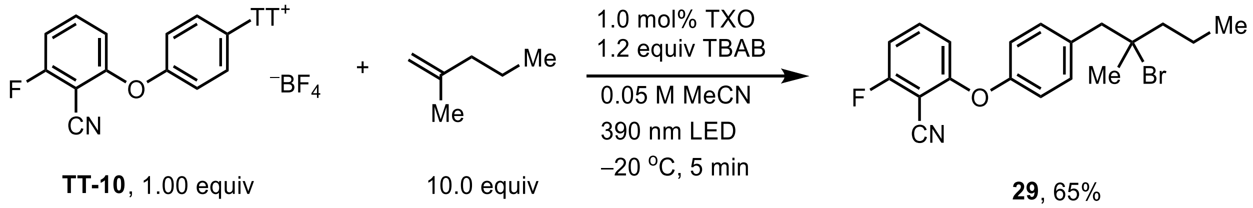

TT-10, 1.00 equiv 10.0 equiv **29**, 65%

To a 5-mL Schlenk tube containing a Teflon-coated magnetic stirring bar were added **TT-10** (103 mg, 0.200

mmol, 1.00 equiv), tetrabutylammonium bormide (TBAB, 77 mg, 0.24 mmol, 1.2 equiv), and thioxanthone in acetonitrile (c = 0.50 mM, 4.0 mL, 2.0 μmol, 1.0 mol%). The tube was sealed with a rubber septum and immersed in a methanol/water (30/70) dry ice bath at –20°C. A balloon filled with argon gas was connected to a long needle, which was inserted to the tube through the rubber septum. Roughly 100 ml of argon gas was bubbled through the solution over a time period of approximately 5 minutes, and excess gas was released through the side arm, all while avoiding exposure to light. The valve of the side arm was closed, and the needle was withdrawn from the solution but remained in the headspace of the vessel. 2-Methyl-1-pentene (246 μL, 168 mg, 2.00 mmol, 10.0 equiv) was added and the reaction mixture was then subjected to irradiation by one Kessil PR160-390 nm LED with 100% intensity (40 W, ~6 cm away) for 5 minutes, all while maintaining the bath temperature at –20°C. Then, the reaction temperature was allowed to rise to 23 ºC. Silica gel (5–10 mL) was added, and the reaction mixture was concentrated to dryness under reduced pressure. The resulting residue was purified by chromatography on silica gel (hexanes/EtOAc = 10:1) to afford **29** as a colorless liquid in 65% yield (48.8 mg).

R*f* = 0.30 (hexanes/EtOAc = 10:1).

NMR Spectroscopy:

^{1}H NMR (300 MHz, $CDCl_3$, 23 ºC, δ): 7.53 – 7.40 (m, 1H), 7.35 (d, *J* = 8.5 Hz, 2H), 7.07 (d, *J* = 8.6 Hz, 2H), 6.96 – 6.85 (m, 1H), 6.66 (d, *J* = 8.6 Hz, 1H), 3.27 (d, *J* = 14.0 Hz, 1H), 3.16 (d, *J* = 14.0 Hz, 1H), 1.97 – 1.57 (m, 4H), 1.72 (s, 3H), 1.00 (t, *J* = 7.0 Hz, 3H).

^{13}C NMR (75 MHz, $CDCl_3$, 23 ºC, δ): 164.0 (d, J = 259.5 Hz), 161.0 (d, J = 4.1 Hz), 153.3, 134.8 (d, J = 10.4 Hz), 134.5, 132.6, 119.7, 111.8 (d, J = 3.5 Hz), 111.1, 109.6 (d, J = 19.5 Hz), 93.6 (d, J = 17.9 Hz), 71.8, 50.6, 47.3, 31.0, 19.3, 14.0.

HRMS-ESIpos (m/z) calc'd for $C_{19}H_{19}FBrNONa$ $[M+Na]^+$, 398.0526; found, 398.0524; deviation: +0.6 ppm.

Arylethyl bromide 30

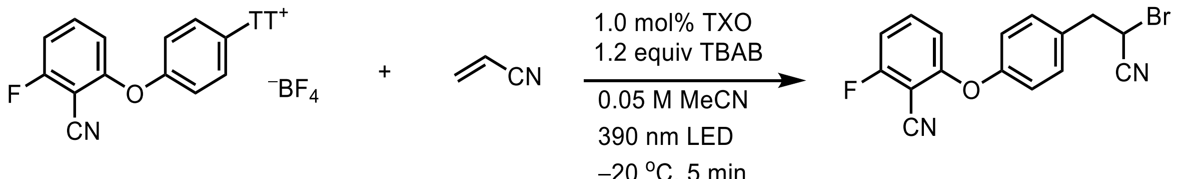

TT-10, 1.00 equiv 3.0 equiv **30**, 78%

To a 5-mL Schlenk tube containing a Teflon-coated magnetic stirring bar were added **TT-10** (103 mg, 0.200 mmol, 1.00 equiv), tetrabutylammonium bormide (TBAB, 77 mg, 0.24 mmol, 1.2 equiv), and thioxanthone in acetonitrile (c = 0.50 mM, 4.0 mL, 2.0 μmol, 1.0 mol%). The tube was sealed with a rubber septum and immersed in a methanol/water (30/70) dry ice bath at –20°C. A balloon filled with argon gas was connected to a long needle, which was inserted to the tube through the rubber septum. Roughly 100 ml of argon gas was bubbled through the solution over a time period of approximately 5 minutes, and excess gas was released through the side arm, all while avoiding exposure to light. The valve of the side arm was closed, and the

needle was withdrawn from the solution but remained in the headspace of the vessel. Acrylonitrile (40 μL, 32 mg, 0.60 mmol, 3.0 equiv) was added and the reaction mixture was then subjected to irradiation by one Kessil PR160-390 nm LED with 100% intensity (40 W, ~6 cm away) for 5 minutes, all while maintaining the bath temperature at –20°C. Then, the reaction temperature was allowed to rise to 23 ºC. Silica gel (5–10 mL) was added, and the reaction mixture was concentrated to dryness under reduced pressure. The resulting residue was purified by chromatography on silica gel (hexanes/EtOAc = 5:1) to afford **30** as a colorless liquid in 78% yield (53.8 mg).

R*f* = 0.20 (hexanes/EtOAc = 5:1).

NMR Spectroscopy:

^{1}H NMR (300 MHz, $CDCl_3$, 23 ºC, δ): 7.48 (td, *J* = 8.5, 6.4 Hz, 1H), 7.39 (d, *J* = 8.6 Hz, 2H), 7.13 (d, *J* = 8.6 Hz, 2H), 6.94 (td, *J* = 8.4, 0.8 Hz, 1H), 6.67 (dd, *J* = 8.5, 0.8 Hz, 1H), 4.48 (t, *J* = 7.3 Hz, 1H), 3.42 (d, *J* = 7.3 Hz, 2H).

^{13}C NMR (75 MHz, $CDCl_3$, 23 ºC, δ): 164.0 (d, *J* = 259.8 Hz), 160.4 (d, *J* = 4.0 Hz), 154.5, 134.9 (d, *J* = 10.3 Hz), 131.8, 131.3, 120.6, 116.7, 112.1 (d, *J* = 3.5 Hz), 110.9, 110.0 (d, *J* = 19.5 Hz), 93.9 (d, *J* = 18.2 Hz), 41.7, 27.1.

HRMS-ESIpos (m/z) calc'd for $C_{16}H_{10}FBrN_2ONa$ $[M+Na]^+$, 366.9853; found, 366.9852; deviation: +0.1 ppm.

Arylethyl bromide 31

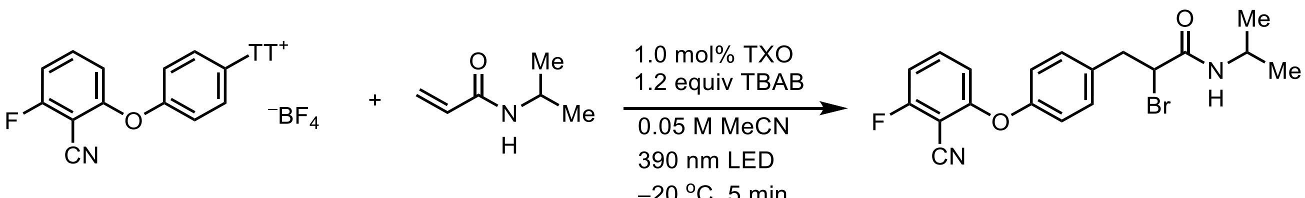

TT-10, 1.00 equiv   3.0 equiv   **31**, 63%

To a 5-mL Schlenk tube containing a Teflon-coated magnetic stirring bar were added **TT-10** (103 mg, 0.200 mmol, 1.00 equiv), *N*-Isopropylacrylamide (68 mg, 0.60 mmol, 3.0 equiv), tetrabutylammonium bormide (TBAB, 77 mg, 0.24 mmol, 1.2 equiv), and thioxanthone in acetonitrile (c = 0.50 mM, 4.0 mL, 2.0 μmol, 1.0 mol%). The tube was sealed with a rubber septum and immersed in a methanol/water (30/70) dry ice bath at –20°C. A balloon filled with argon gas was connected to a long needle, which was inserted to the tube through the rubber septum. Roughly 100 ml of argon gas was bubbled through the solution over a time period of approximately 5 minutes, and excess gas was released through the side arm, all while avoiding exposure to light. The valve of the side arm was closed, and the needle was withdrawn from the solution but remained in the headspace of the vessel. The reaction mixture was then subjected to irradiation by one Kessil PR160-390 nm LED with 100% intensity (40 W, ~6 cm away) for 5 minutes, all while maintaining the bath temperature at –20°C. Then, the reaction temperature was allowed to rise to 23 ºC. Silica gel (5–10 mL) was added, and the reaction mixture was concentrated to dryness under reduced pressure. The resulting residue was purified by chromatography on silica gel (hexanes/EtOAc = 3:1) to afford **31** as a colorless solid in 63%

yield (51.0 mg).

R*f* = 0.30 (hexanes/EtOAc = 2:1).

NMR Spectroscopy:

^{1}H NMR (500 MHz, $CDCl_3$, 23 ºC, δ): 7.48 – 7.39 (m, 1H), 7.31 (d, *J* = 8.5 Hz, 2H), 7.05 (d, *J* = 8.5 Hz, 2H), 6.90 (t, *J* = 8.4 Hz, 1H), 6.61 (d, *J* = 8.5 Hz, 1H), 6.13 (d, *J* = 7.9 Hz, 1H), 4.46 (t, *J* = 6.5 Hz, 1H), 4.10 – 3.99 (m, 1H), 3.53 (dd, *J* = 14.4, 5.8 Hz, 1H), 3.33 (dd, *J* = 14.3, 7.2 Hz, 1H), 1.14 (d, *J* = 6.6 Hz, 6H).

^{13}C NMR (125 MHz, $CDCl_3$, 23 ºC, δ): 166.7, 164.1 (d, *J* = 259.4 Hz), 161.0 (d, *J* = 4.1 Hz), 153.6, 134.9 (d, *J* = 10.2 Hz), 134.5, 131.4, 120.2, 111.9 (d, *J* = 3.5 Hz), 111.1, 109.7 (d, *J* = 19.4 Hz), 93.6 (d, *J* = 18.3 Hz), 51.4, 42.3, 40.8, 22.4, 22.3.

HRMS-ESIpos (m/z) calc'd for $C_{19}H_{18}FBrN_2O_2Na$ $[M+Na]^+$, 427.0428; found, 427.0427; deviation: +0.3 ppm.

Arylethyl bromide 32

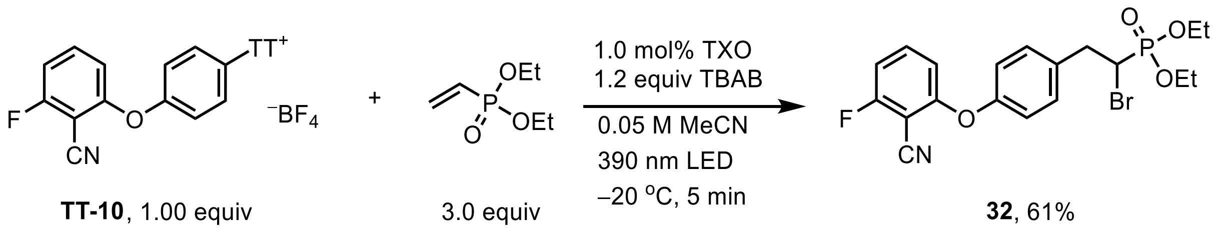

TT-10, 1.00 equiv 3.0 equiv **32**, 61%

To a 5-mL Schlenk tube containing a Teflon-coated magnetic stirring bar were added **TT-10** (103 mg, 0.200 mmol, 1.00 equiv), tetrabutylammonium bormide (TBAB, 77 mg, 0.24 mmol, 1.2 equiv), and thioxanthone in acetonitrile (c = 0.50 mM, 4.0 mL, 2.0 μmol, 1.0 mol%). The tube was sealed with a rubber septum and immersed in a methanol/water (30/70) dry ice bath at –20°C. A balloon filled with argon gas was connected to a long needle, which was inserted to the tube through the rubber septum. Roughly 100 ml of argon gas was bubbled through the solution over a time period of approximately 5 minutes, and excess gas was released through the side arm, all while avoiding exposure to light. The valve of the side arm was closed, and the needle was withdrawn from the solution but remained in the headspace of the vessel. Diethyl vinylphosphonate (93 μL, 99 mg, 0.60 mmol, 3.0 equiv) was added and the reaction mixture was then subjected to irradiation by one Kessil PR160-390 nm LED with 100% intensity (40 W, ~6 cm away) for 5 minutes, all while maintaining the bath temperature at –20°C. Then, the reaction temperature was allowed to rise to 23 ºC. Silica gel (5–10 mL) was added, and the reaction mixture was concentrated to dryness under reduced pressure. The resulting residue was purified by chromatography on silica gel (hexanes/EtOAc = 1:2) to afford **32** as a colorless liquid in 61% yield (55.6 mg).

R*f* = 0.30 (hexanes/EtOAc = 1:2).

NMR Spectroscopy:

^{1}**H NMR** (300 MHz, CDCl$_3$, 23 ºC, δ): 7.43 (td, *J* = 8.5, 6.4 Hz, 1H), 7.30 (d, *J* = 8.5 Hz, 2H), 7.06 (d, *J* = 8.5 Hz, 2H), 6.88 (td, *J* = 8.4, 0.8 Hz, 1H), 6.61 (d, *J* = 8.6 Hz, 1H), 4.37 – 4.16 (m, 4H), 3.97 (ddd, *J* = 11.3, 9.9, 3.4 Hz, 1H), 3.59 (ddd, *J* = 14.9, 7.2, 3.4 Hz, 1H), 3.10 (ddd, *J* = 14.9, 11.3, 8.3 Hz, 1H), 1.37 (t, *J* = 7.0 Hz, 6H).

^{13}**C NMR** (75 MHz, CDCl$_3$, 23 ºC, δ): 163.9 (d, *J* = 259.4 Hz), 160.8 (d, *J* = 4.1 Hz), 153.4, 134.8 (d, *J* = 10.3 Hz), 134.6, 130.9, 120.2, 111.7 (d, *J* = 3.5 Hz), 111.0, 109.6 (d, *J* = 19.5 Hz), 93.5 (d, *J* = 18.1 Hz), 64.0 (d, *J* = 7.1 Hz), 63.5 (d, *J* = 6.9 Hz), 42.4 (d, *J* = 155.7 Hz), 37.9, 16.4 (d, *J* = 2.4 Hz), 16.3 (d, *J* = 2.4 Hz).

^{31}**P NMR** (122 MHz, CDCl$_3$, 23 ºC, δ): 19.27.

HRMS-ESIpos (m/z) calc'd for $C_{19}H_{20}FBrNPO_4Na$ [M+Na]$^+$, 478.0190; found, 478.0191; deviation: –0.2 ppm.

Arylethyl bromide 33

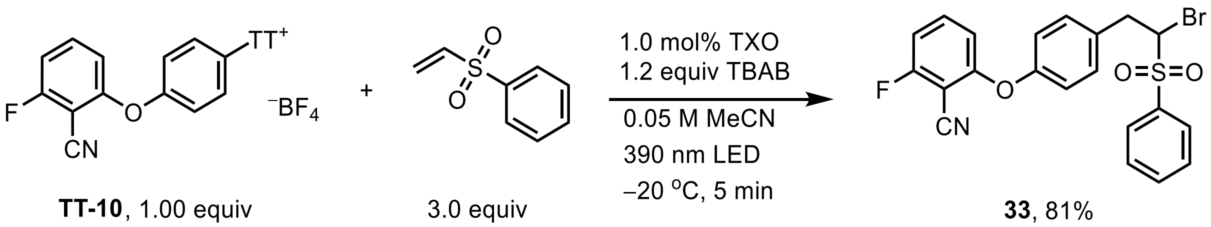

TT-10, 1.00 equiv 3.0 equiv **33**, 81%

To a 5-mL Schlenk tube containing a Teflon-coated magnetic stirring bar were added **TT-10** (103 mg, 0.200 mmol, 1.00 equiv), tetrabutylammonium bormide (TBAB, 77 mg, 0.24 mmol, 1.2 equiv), phenyl vinyl sulfone (102 mg, 0.60 mmol, 3.0 equiv), and thioxanthone in acetonitrile (c = 0.50 mM, 4.0 mL, 2.0 μmol, 1.0 mol%). The tube was sealed with a rubber septum and immersed in a methanol/water (30/70) dry ice bath at –20°C. A balloon filled with argon gas was connected to a long needle, which was inserted to the tube through the rubber septum. Roughly 100 ml of argon gas was bubbled through the solution over a time period of approximately 5 minutes, and excess gas was released through the side arm, all while avoiding exposure to light. The valve of the side arm was closed, and the needle was withdrawn from the solution but remained in the headspace of the vessel. The reaction mixture was then subjected to irradiation by one Kessil PR160-390 nm LED with 100% intensity (40 W, ~6 cm away) for 5 minutes, all while maintaining the bath temperature at –20°C. Then, the reaction temperature was allowed to rise to 23 ºC. Silica gel (5–10 mL) was added, and the reaction mixture was concentrated to dryness under reduced pressure. The resulting residue was purified by chromatography on silica gel (hexanes/acetone = 4:1) to afford **33** as a colorless solid in 81% yield (74.5 mg).

$\mathbf{R}_f$ = 0.20 (hexanes/acetone = 4:1).

NMR Spectroscopy:

^{1}**H NMR** (500 MHz, CDCl$_3$, 23 ºC, δ): 8.04 (d, *J* = 7.0 Hz, 2H), 7.76 (t, *J* = 7.5 Hz, 1H), 7.64 (t, *J* = 7.9 Hz, 2H), 7.45 (td, *J* = 8.5, 6.4 Hz, 1H), 7.30 (d, *J* = 8.6 Hz, 2H), 7.07 (d, *J* = 8.5 Hz, 2H), 6.91 (t, *J* = 8.4 Hz, 1H), 6.62 (d, *J* = 8.6 Hz, 1H), 4.88 (dd, *J* = 11.4, 2.7 Hz, 1H), 3.95 (dd, *J* = 14.6, 2.7 Hz, 1H), 3.13

(dd, *J* = 14.6, 11.4 Hz, 1H).

^{13}C NMR (125 MHz, $CDCl_3$, 23 ºC, δ): 164.0 (d, *J* = 259.8 Hz), 160.7 (d, *J* = 4.1 Hz), 153.9, 135.1, 135.0 (d, *J* = 10.2 Hz), 134.8, 132.6, 131.3, 130.1, 129.3, 120.5, 112.0 (d, *J* = 3.5 Hz), 111.1, 109.9 (d, *J* = 19.6 Hz), 93.8 (d, *J* = 18.0 Hz), 66.2, 36.8.

HRMS-ESIpos (m/z) calc'd for $C_{21}H_{15}FBrNO_3SNa$ $[M+Na]^+$, 481.9832; found, 481.9830; deviation: +0.6 ppm.

Arylethyl bromide 34

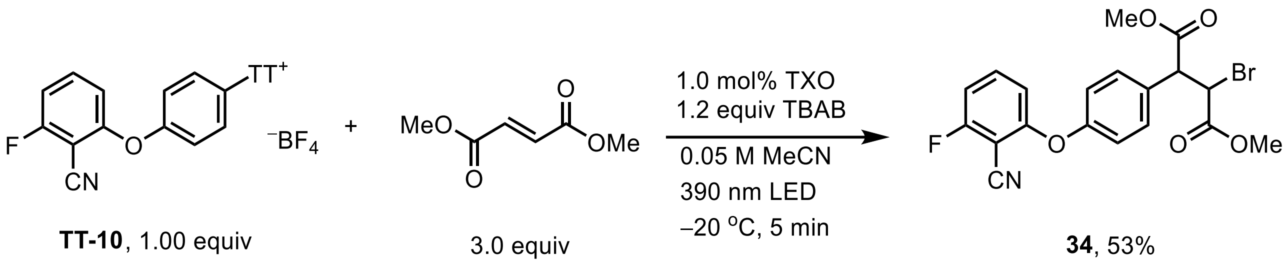

TT-10, 1.00 equiv 3.0 equiv **34**, 53%

To a 5-mL Schlenk tube containing a Teflon-coated magnetic stirring bar were added **TT-10** (103 mg, 0.200 mmol, 1.00 equiv), tetrabutylammonium bormide (TBAB, 77 mg, 0.24 mmol, 1.2 equiv), dimethyl fumarate (86 mg, 0.60 mmol, 3.0 equiv), and thioxanthone in acetonitrile (c = 0.50 mM, 4.0 mL, 2.0 µmol, 1.0 mol%). The tube was sealed with a rubber septum and immersed in a methanol/water (30/70) dry ice bath at –20°C. A balloon filled with argon gas was connected to a long needle, which was inserted to the tube through the rubber septum. Roughly 100 ml of argon gas was bubbled through the solution over a time period of approximately 5 minutes, and excess gas was released through the side arm, all while avoiding exposure to light. The valve of the side arm was closed, and the needle was withdrawn from the solution but remained in the headspace of the vessel. The reaction mixture was then subjected to irradiation by one Kessil PR160-390 nm LED with 100% intensity (40 W, ~6 cm away) for 5 minutes, all while maintaining the bath temperature at –20°C. Then, the reaction temperature was allowed to rise to 23 ºC. Silica gel (5–10 mL) was added, and the reaction mixture was concentrated to dryness under reduced pressure. The resulting residue was purified by chromatography on silica gel (hexanes/EtOAc = 5:1) to afford **34** as a colorless solid in 53% yield (46.2 mg).

R_f = 0.20 (hexanes/EtOAc = 5:1).

NMR Spectroscopy:

Major isomer

^{1}H NMR (500 MHz, $CDCl_3$, 23 ºC, δ): 7.51 – 7.44 (m, 1H), 7.44 (d, *J* = 8.5 Hz, 2H), 7.07 (d, *J* = 8.6 Hz, 2H), 6.94 (td, *J* = 8.4, 0.8 Hz, 1H), 6.65 (d, *J* = 8.5 Hz, 1H), 4.84 (d, *J* = 11.6 Hz, 1H), 4.35 (d, *J* = 11.6 Hz, 1H), 3.79 (s, 3H), 3.61 (s, 3H).

^{13}C NMR (125 MHz, $CDCl_3$, 23 ºC, δ): 170.6, 168.0, 164.1 (d, *J* = 260.0 Hz), 160.2 (d, *J* = 4.0 Hz), 155.0, 134.9 (d, *J* = 10.2 Hz), 131.3, 130.3, 120.4, 112.5 (d, *J* = 3.5 Hz), 110.9, 110.3 (d, *J* = 19.6 Hz), 94.2 (d, *J* = 17.8 Hz), 55.4, 53.1, 52.8, 43.9.

HRMS-ESIpos (m/z) calc'd for $C_{19}H_{15}FBrNO_5Na$ [M+Na]$^+$, 458.0010; found, 458.0007; deviation: +0.7 ppm.

Arylethyl bromide 35

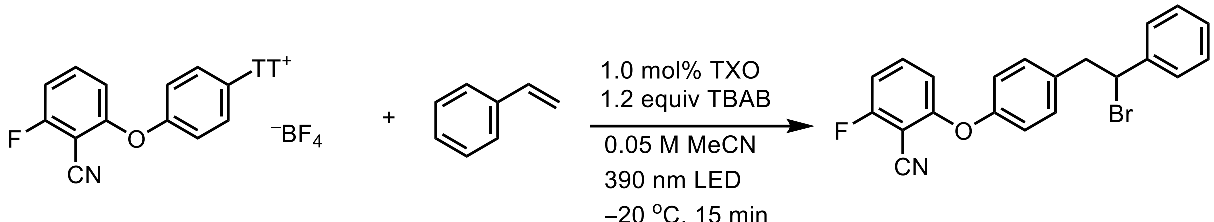

TT-10, 1.00 equiv　　3.0 equiv　　**35**, 75%

To a 5-mL Schlenk tube containing a Teflon-coated magnetic stirring bar were added **TT-10** (103 mg, 0.200 mmol, 1.00 equiv), tetrabutylammonium bormide (TBAB, 77 mg, 0.24 mmol, 1.2 equiv), and thioxanthone in acetonitrile (c = 0.50 mM, 4.0 mL, 2.0 μmol, 1.0 mol%). The tube was sealed with a rubber septum and immersed in a methanol/water (30/70) dry ice bath at –20°C. A balloon filled with argon gas was connected to a long needle, which was inserted to the tube through the rubber septum. Roughly 100 ml of argon gas was bubbled through the solution over a time period of approximately 5 minutes, and excess gas was released through the side arm, all while avoiding exposure to light. The valve of the side arm was closed, and the needle was withdrawn from the solution but remained in the headspace of the vessel. Styrene (69 μL, 62 mg, 0.60 mmol, 3.0 equiv) was added and the reaction mixture was then subjected to irradiation by one Kessil PR160-390 nm LED with 100% intensity (40 W, ~6 cm away) for 15 minutes, all while maintaining the bath temperature at –20°C. Then, the reaction temperature was allowed to rise to 23 ºC. Silica gel (5–10 mL) was added, and the reaction mixture was concentrated to dryness under reduced pressure. The resulting residue was purified by chromatography on silica gel (hexanes/EtOAc = 10:1) to afford **35** as a colorless liquid in 75% yield (59.4 mg).

R*f* = 0.21 (hexanes/EtOAc = 10:1).

NMR Spectroscopy:

^{1}H NMR (300 MHz, $CDCl_3$, 23 ºC, δ): 7.48 – 7.27 (m, 6H), 7.18 (d, *J* = 8.5 Hz, 2H), 7.01 (d, *J* = 8.5 Hz, 2H), 6.89 (td, *J* = 8.4, 0.9 Hz, 1H), 6.58 (d, *J* = 8.6 Hz, 1H), 5.14 (t, *J* = 7.5 Hz, 1H), 3.56 (qd, *J* = 14.2, 7.6 Hz, 2H).

^{13}C NMR (75 MHz, $CDCl_3$, 23 ºC, δ): 164.0 (d, *J* = 259.5 Hz), 161.0 (d, *J* = 4.1 Hz), 153.2, 141.1, 135.5, 134.8 (d, *J* = 10.3 Hz), 131.0, 128.6, 128.5, 127.4, 120.2, 111.7 (d, *J* = 3.4 Hz), 111.1, 109.5 (d, *J* = 19.5 Hz), 93.5 (d, *J* = 18.3 Hz), 55.1, 45.7.

HRMS-ESIpos (m/z) calc'd for $C_{21}H_{15}FBrNONa$ [M+Na]$^+$, 418.0213; found, 418.0212; deviation: +0.2 ppm.

Arylethyl bromide 36

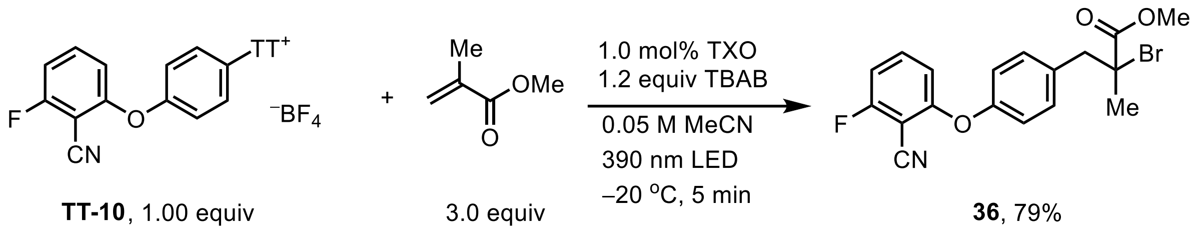

TT-10, 1.00 equiv   3.0 equiv   **36**, 79%

To a 5-mL Schlenk tube containing a Teflon-coated magnetic stirring bar were added **TT-10** (103 mg, 0.200 mmol, 1.00 equiv), tetrabutylammonium bormide (TBAB, 77 mg, 0.24 mmol, 1.2 equiv), and thioxanthone in acetonitrile (c = 0.50 mM, 4.0 mL, 2.0 μmol, 1.0 mol%). The tube was sealed with a rubber septum and immersed in a methanol/water (30/70) dry ice bath at –20°C. A balloon filled with argon gas was connected to a long needle, which was inserted to the tube through the rubber septum. Roughly 100 ml of argon gas was bubbled through the solution over a time period of approximately 5 minutes, and excess gas was released through the side arm, all while avoiding exposure to light. The valve of the side arm was closed, and the needle was withdrawn from the solution but remained in the headspace of the vessel. Methyl methacrylate (64 μL, 60 mg, 0.60 mmol, 3.0 equiv) was added and the reaction mixture was then subjected to irradiation by one Kessil PR160-390 nm LED with 100% intensity (40 W, ~6 cm away) for 5 minutes, all while maintaining the bath temperature at –20°C. Then, the reaction temperature was allowed to rise to 23 ºC. Silica gel (5–10 mL) was added, and the reaction mixture was concentrated to dryness under reduced pressure. The resulting residue was purified by chromatography on silica gel (hexanes/EtOAc = 20:1) to afford **36** as a colorless liquid in 79% yield (61.9 mg).

R_f = 0.16 (hexanes/EtOAc = 20:1).

NMR Spectroscopy:

^{1}H NMR (500 MHz, $CDCl_3$, 23 ºC, δ): 7.45 (td, *J* = 8.5, 6.4 Hz, 1H), 7.30 (d, *J* = 8.6 Hz, 2H), 7.06 (d, *J* = 8.5 Hz, 2H), 6.91 (td, *J* = 8.4, 0.8 Hz, 1H), 6.63 (d, *J* = 8.6 Hz, 1H), 3.83 (s, 3H), 3.61 (d, *J* = 13.9 Hz, 1H), 3.41 (d, *J* = 13.9 Hz, 1H), 1.88 (s, 3H).

^{13}C NMR (125 MHz, $CDCl_3$, 23 ºC, δ): 171.3, 164.0 (d, *J* = 259.7 Hz), 160.7 (d, *J* = 4.0 Hz), 153.8, 134.8 (d, *J* = 10.3 Hz), 133.2, 132.3, 120.0, 111.9 (d, *J* = 3.4 Hz), 111.0, 109.7 (d, *J* = 19.5 Hz), 93.7 (d, *J* = 18.2 Hz), 59.6, 53.2, 47.2, 27.4.

HRMS-ESIpos (m/z) calc'd for $C_{18}H_{15}FBrNO_3Na$ $[M+Na]^+$, 414.0112; found, 414.0110; deviation: +0.4 ppm.

Condition optimization

General procedure for condition optimization of two-carbon homologation of ArTTs

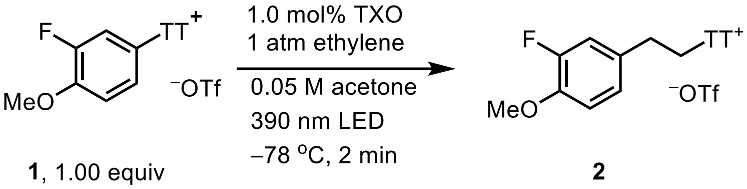

1, 1.00 equiv **2**

To a 5-mL Schlenk tube containing a Teflon-coated magnetic stirring bar were added arylthianthrenium salt (0.050 mmol, 1.00 equiv) and thioxanthone in acetone (c = 0.50 mM, 1.0 mL, 0.50 μmol, 1.0 mol%). The tube was sealed with a rubber septum and immersed in a dry ice/acetone bath at –78°C. A balloon filled with ethylene gas was connected to a long needle, which was inserted to the tube through the rubber septum. Roughly 50 ml of ethylene gas was bubbled through the solution over a time period of approximately 5 minutes, and excess gas was released through the side arm, all while avoiding exposure to light. The valve of the side arm was closed, and the needle was withdrawn from the solution but remained in the headspace of the vessel. The reaction mixture was then subjected to irradiation by one Kessil PR160-390 nm LED with 100% intensity (40 W, ~6 cm away) for 2 minutes, all while maintaining the bath temperature at –78°C. Subsequently, 1,3,5-trimethoxybenzene (50 μL, 0.050 mmol, 1.0 M in MeCN, 1.0 equiv) was added as internal standard. The reaction mixture was concentrated and analyzed by ^{1}H NMR spectroscopy. The conversions of **1** and the yields of **2** were determined by the integrating the signal of the characteristic protons at 8.52 ppm (d) and 4.04 ppm (t) respectively.

Table S2. Condition optimization of two-carbon homologation of ArTTs

entry	changes from reaction conditions	conv. of **1**/%	yield of **2**/%
1	no change	>99	82
2	0.5 mol% instead of 1.0 mol%	95	83
3	0.25 mol% instead of 1.0 mol%	35	24
4	15 min instead of 2 min	>99	52
5	–20 ^oC instead of –78 ^oC	>99	46
6	0.1 M instead of 0.05 M	>99	81
7	MeCN instead of acetone, –40 ^oC	>99	80
8	DCM instead of acetone	>99	78
9	XO, 3CzClIPN, Mes-AcrClO_4, Ru1, Ir1, Ir2, Ir3 or Ir4 instead of TXO	<5	<1
10	Ir4 and 40 mol% TT instead of TXO	16	12
11	BP instead of TXO	<5	<1
12	20 mol% BP instead of TXO	43	36
13[a]	20 mol% BP instead of TXO	>99	72
14[b]	254 nm, w/o TXO	>99	<1
15[b]	254 nm, w/o TXO, with 2.0 equiv TBAB	>99	23[c]
16	2.0 equiv TEMPO was added	3	2
17	w/o TXO	<2	<1
18	w/o light	<2	<1

[a]20 min; [b]MeCN, –40 to 23 ^oC, 12 h instead of acetone, –78 ^oC, 2 min; [c]arylethyl bromide instead of **2** as product.

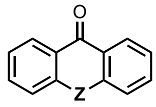

Z = H H, BP
Z = O, XO
Z = S, TXO

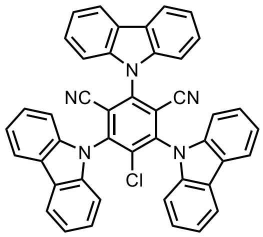

3CzClIPN

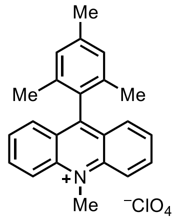

Mes-AcrClO$_4$

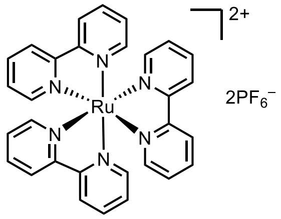

Ru1

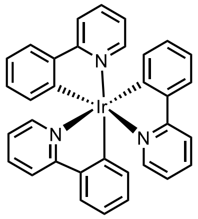

Ir1

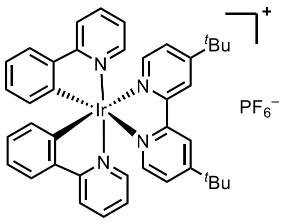

Ir2

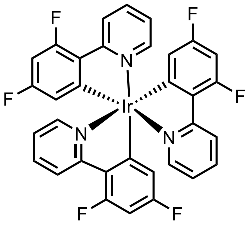

Ir3

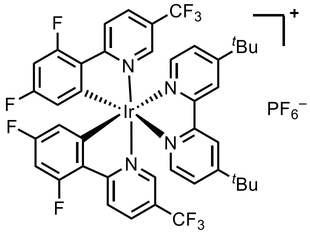

Ir4

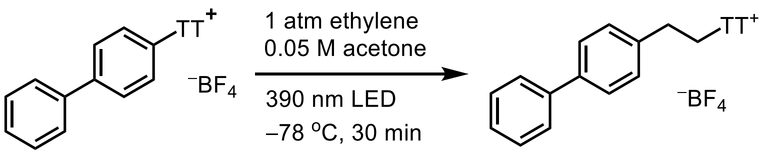

S2, 0.1 mmol **S3**, < 2% yield

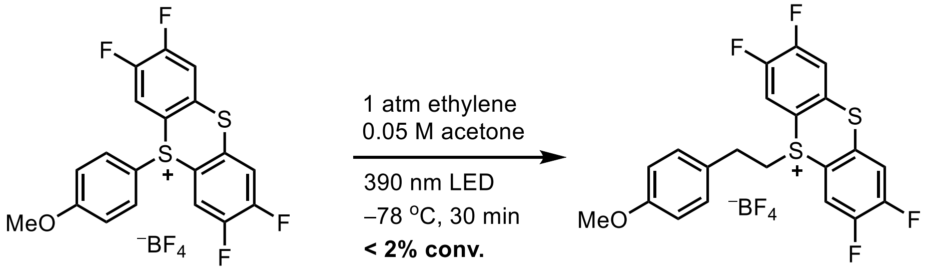

S4, 0.1 mmol **S5**, < 2% yield

Scheme S1. Reactions with substrates having extended conjugation in the absence of TXO

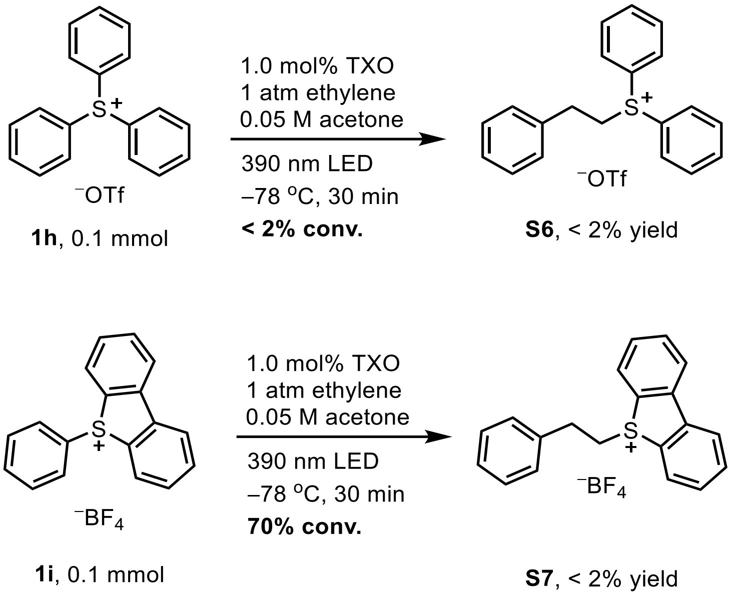

Scheme S2. Reactions of triphenyl sulfonium salt (**1h**) and 5-phenyldibenzothiophenium salt (**1i**) with an extended reaction time

Table S3 Light source screening

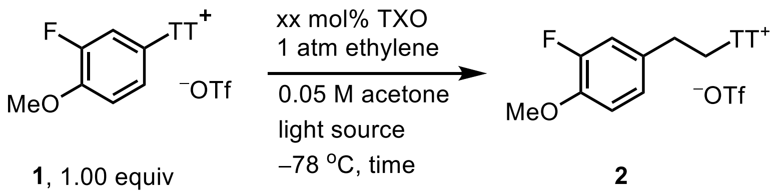

entry	light source	cat. loading (mol%)	time	conv. of **1** (%)	yield of **2** (%)
1	Kessel 390 nm LED (40 W)	1	2 min	>99	82
2	Kessel 390 nm LED (20 W)	1	2 min	98	78
3	EvoluChem™ 405 nm LED (18 W)	1	2 min	19	15
4	EvoluChem™ 405 nm LED (18 W)	1	15 min	94	77
5	EvoluChem™ 425 nm LED (18 W)	1	15 min	3	<2
6	EvoluChem™ 425 nm LED (18 W)	10	15 min	14	10
7[a]	EvoluChem™ 425 nm LED (18 W)	10	15 min	32	24
8	EvoluChem™ 425 nm LED (18 W)	10	60 min	57	29
9[b]	EvoluChem™ 425 nm LED (18 W)	10	15 min	7	6
10	Kessel 456 nm LED (80 W)	10	15 min	<2	<2

[a]0.025 M; [b]2-isopropylthioxanthone was used.

Reaction with high pressure ethylene at room temperature

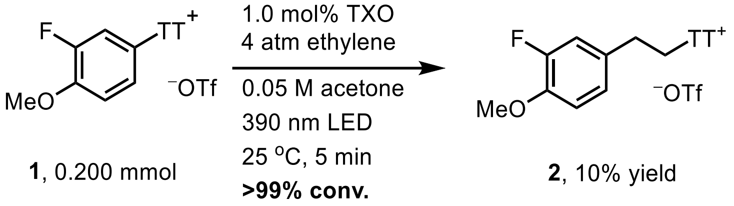

To a 20-mL microwave vial containing a Teflon-coated magnetic stirring bar were added arylthianthrenium salt **1** (98.0 mg, 0.200 mmol, 1.00 equiv) and thioxanthone in acetone (c = 0.50 mM, 4.0 mL, 2.0 μmol, 1.0 mol%). The vial was sealed with an aluminium crimp seal with septum. The reaction mixture was then bubbled with ethylene using a syringe needle for 15 minutes in an ice bath. Afterward, gaseous ethylene was introduced into the headspace using a syringe (48 mL) and sealed promptly with two layers of Parafilm®. The reaction mixture was then subjected to irradiation by one Kessil PR160-390 nm LED with 100% intensity (40 W, ~3 cm away) for 5 minutes at room temperature (23–25 °C). Subsequently, 1,3,5-trimethoxybenzene (200 μL, 0.200 mmol, 1.0 M in MeCN, 1.0 equiv) was added as internal standard. The reaction mixture was concentrated and analyzed by ^{1}H NMR spectroscopy. The conversions of **1** (>99%) and the yields of **2** (10%) were determined by the integrating the signal of the characteristic protons at 8.52 ppm (d) and 4.04 ppm (t) respectively.

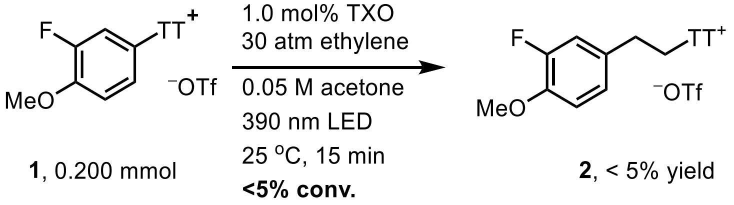

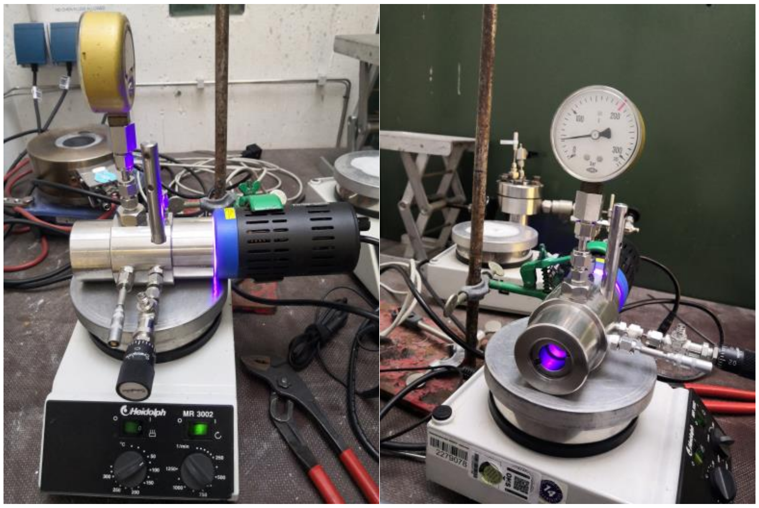

To a 10-mL autoclave with two glass windows containing a Teflon-coated magnetic stirring bar were added arylthianthrenium salt **1** (98.0 mg, 0.200 mmol, 1.00 equiv) and thioxanthone in acetone (c = 0.50 mM, 4.0 mL, 2.0 μmol, 1.0 mol%). The autoclave was purged three times with ethylene gas and pressed to 30–50 atm. The reaction mixture was then subjected to irradiation by one Kessil PR160-390 nm LED with 100% intensity (40 W, ~3 cm away) for 15 minutes at room temperature (23–25 °C). Subsequently, 1,3,5-

trimethoxybenzene (200 μL, 0.200 mmol, 1.0 M in MeCN, 1.0 equiv) was added as internal standard. The reaction mixture was concentrated and analyzed by ^{1}H NMR spectroscopy. The conversions of **1** and the yields of **2** were determined to be less than 5% by the integrating the signal of the characteristic protons at 8.52 ppm (d) and 4.04 ppm (t) respectively.

General procedure for optimization of base for aminoarylation of ethylene

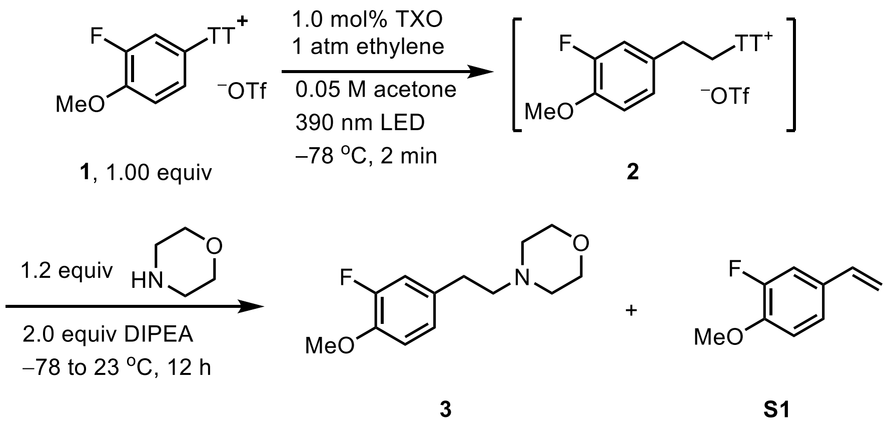

To a 5-mL Schlenk tube containing a Teflon-coated magnetic stirring bar were added arylthianthrenium salt (0.050 mmol, 1.00 equiv) and thioxanthone in acetone (c = 0.50 mM, 1.0 mL, 0.50 μmol, 1.0 mol%). The tube was sealed with a rubber septum and immersed in a dry ice/acetone bath at –78°C. A balloon filled with ethylene gas was connected to a long needle, which was inserted to the tube through the rubber septum. Roughly 50 ml of ethylene gas was bubbled through the solution over a time period of approximately 5 minutes, and excess gas was released through the side arm, all while avoiding exposure to light. The valve of the side arm was closed, and the needle was withdrawn from the solution but remained in the headspace of the vessel. The reaction mixture was then subjected to irradiation by one Kessil PR160-390 nm LED with 100% intensity (40 W, ~6 cm away) for 2 minutes, all while maintaining the bath temperature at –78°C. Then, morpholine (5.1 μL, 5.2 mg, 0.060 mmol, 1.2 equiv) and base (0.10 mmol, 2.0 equiv) were added. The resulting mixture was stirred for 12 h without exposure to light. During this period, the reaction temperature was gradually allowed to rise to 23 ºC. Subsequently, 1,3,5-trimethoxybenzene (50 μL, 0.050 mmol, 1.0 M in MeCN, 1.0 equiv) was added as internal standard. The reaction mixture was concentrated and analyzed by ^{1}H NMR spectroscopy. The conversion of **1** and the yields of **2**, **3**, and **S1** were determined by the integrating the signal of the characteristic protons at 8.52 ppm (d), 4.04 ppm (t), 2.74 (t), and 5.61 (d) respectively.

Table S4. Base optimization of 1,2-aminoarylation of ethylene

entry	base	conv. of **1**/%	yield of **2**/%	yield of **3**/%	yield of **S1**/%
1	DIPEA	>99	<2	75	9
2	$NaHCO_3$	>99	<2	74	10
3	K_2CO_3	>99	<2	65	14
4	K_3PO_4	>99	<2	61	16
5	Et_3N	>99	<2	20	62
6	pyridine	>99	17	<2	56
7	DBU	>99	<2	2	78
8	DABCO	>99	<2	5	11(68)[a]
9	w/o base	>99	14	58	<2

[a]the yield in the bracket referred to the substitution product by DABCO

Investigation of adding nucleophiles from the beginning of the reaction

Before conducting the experiment in which nucleophiles were added at the beginning of the reaction, we first investigated the reactivity of an isolable TT radical cation (0.02 mmol, 1.0 equiv) with six different nucleophiles (0.05 mmol, 2.5 equiv) in acetonitrile (0.04 M) at 25 ^{o}C: chloride, bromide, iodide, aliphatic amine, carboxylate, and thiophenolate. Each of these nucleophiles is known to work well when added after the photoreaction. In these preliminary tests, the characteristic purple color of the TT radical cation disappeared within 1 to 20 seconds, turning colorless, or yellow with bromide, and brown with iodide (see Scheme S3). These results indicate that all six nucleophiles can react with the TT radical cation rapidly.

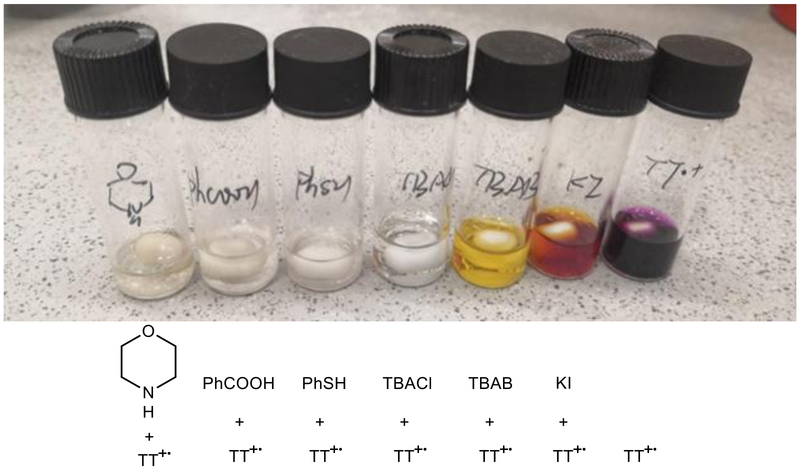

Scheme S3. Color change after mixing TT radical cation and different nucleophiles

Next, we explored reactions where the nucleophile was added at the beginning of the reaction. The outcomes varied significantly and are summarized in Scheme S4. Using chloride and bromide as nucleophiles, the desired products were obtained in 77-88% yields. With iodide, 50% conversion was observed, yielding 22% of the desired product along with 22% aryl iodide by-product. When morpholine was used, low conversion was observed, and the desired product was not detected. With benzoate as the nucleophile, the reaction showed full conversion, yielding 22% of the desired product and 22% of the arylethyl thianthrenium salt intermediate. Thiophenolate led to full conversion as well; however, the desired product was not observed, and direct sulfidation products along with ethyl arene were formed.

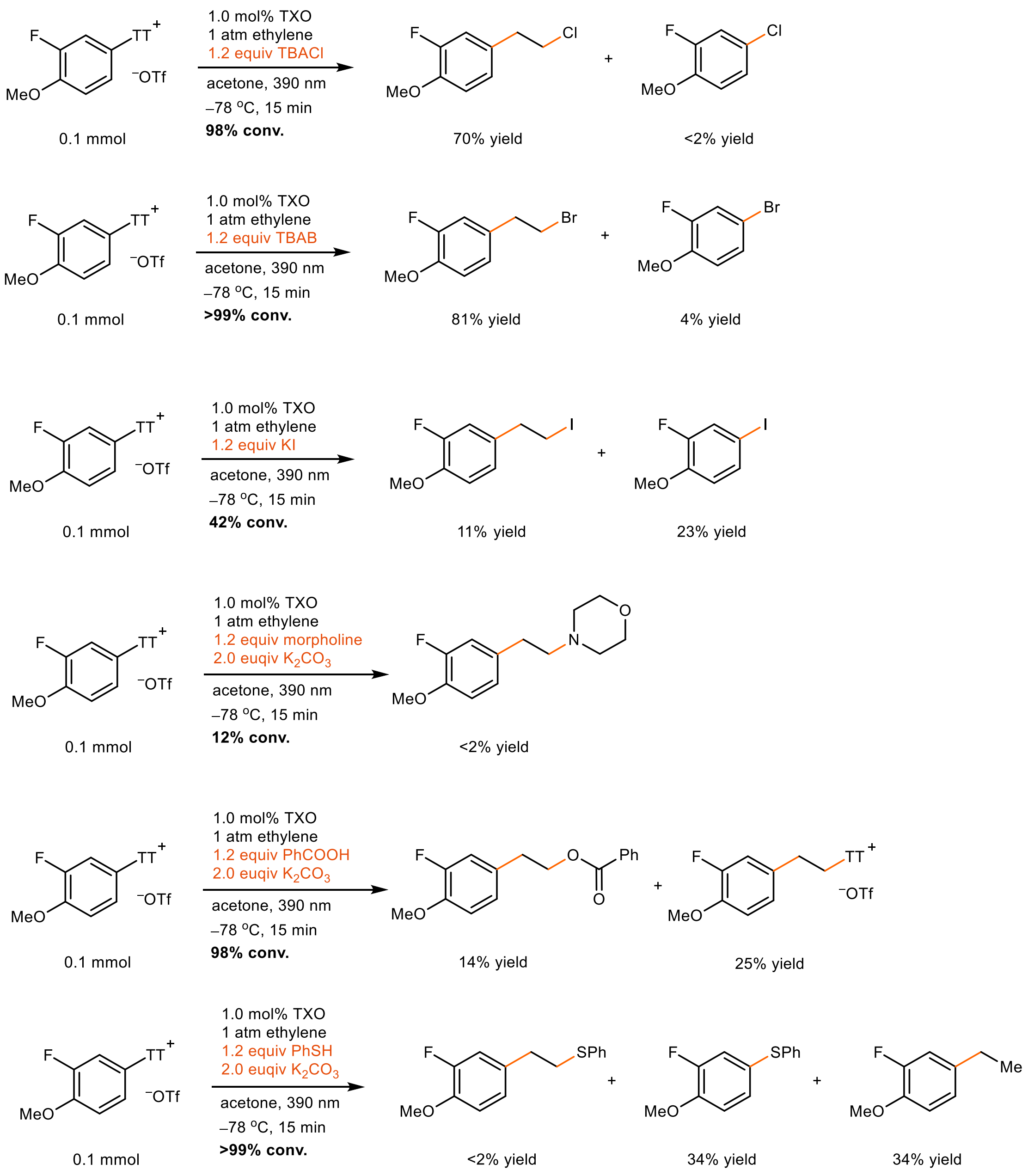

Scheme S4. Reactions with nucleophiles added from the beginning of the reaction

Chloride and bromide can still be added at the beginning of the reaction, likely because even when the nucleophile reacts with the TT radical cation, the resulting halide radical can dimerize to form molecular halogen. This halogen can then react with the aryl ethyl radical, ultimately leading to the formation of the same alkyl halide product via a two-step process. Other nucleophiles, such as iodide, aliphatic amines, carboxylates, and thiophenolates, are likely unable to participate due to their competitive reaction with the thianthrenium radical cation.

Further optimization of the reaction conditions

The main side products during the first photoreaction step are hydrodefunctionalization (**bp1**, 8%) and the Minisci byproduct (**bp2**, 9%). The hydrodefunctionalization byproduct was reduced to 4% by further decreasing the reaction temperature from −78 °C to −94 °C, which increased the solubility of ethylene. The Minisci byproduct can be reduced by carefully controlling the reaction time or suppressed by adding a bromide nucleophile to capture the alkyl thianthrenium salt directly. Overall, the photoreaction yield can now be increased from 82% to 91% (eq. 2). In the second substitution step, the primary side reaction is elimination, leading to the formation of styrene (**bp3**, 9%, eq. 1), particularly when N and O-based nucleophiles, as well as $^{-}$F and $^{-}$CN, are used. This issue is addressed to some extent by converting alkylthianthrenium salts to alkyl bromides in situ and then adding the nucleophile (eq. 2). This approach results in fewer elimination byproduct (4%), likely because uncharged alkyl bromides are less prone to elimination. Utilizing this strategy, along with the optimized conditions from the first photoreaction step, the yield of product 3 can be improved from 73% to 81%. Additionally, the yields of other substrates (**4**, **5**, **6**, **20**) with previously moderate results (41–55%) are also improved (60–81%), as shown in Scheme S5.

Previous conditions:

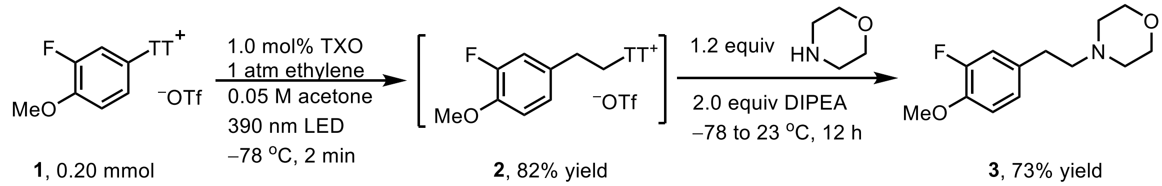

1, 0.20 mmol **2**, 82% yield **3**, 73% yield eq. 1

Optimized conditions:

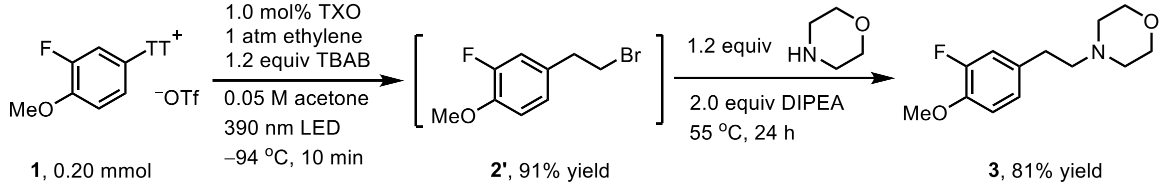

1, 0.20 mmol **2'**, 91% yield **3**, 81% yield eq. 2

byproducts

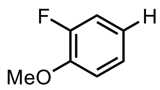

bp1
previous conditions: 8% yield
optimized conditions: 4% yield

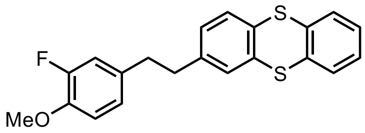

bp2
previous conditions: 9% yield
optimized conditions: <2% yield

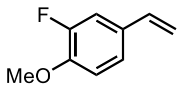

bp3
previous conditions: 8% yield
optimized conditions: 4% yield

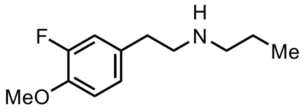

4
previous conditions: 49%
optimized conditions: 72%

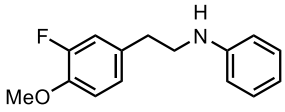

5
previous conditions: 45%
optimized conditions: 63%

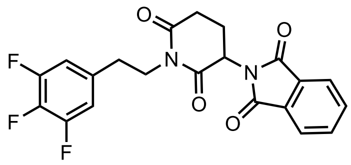

6
previous conditions: 55%
optimized conditions: 60%

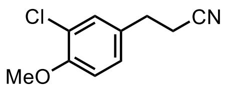

20
previous conditions: 41%
optimized conditions: 81%

Scheme S5 Further optimization of reaction conditions and improvement of yields for several substrates

Mechanistic studies

Transient absorption spectroscopy and related spectroscopic studies

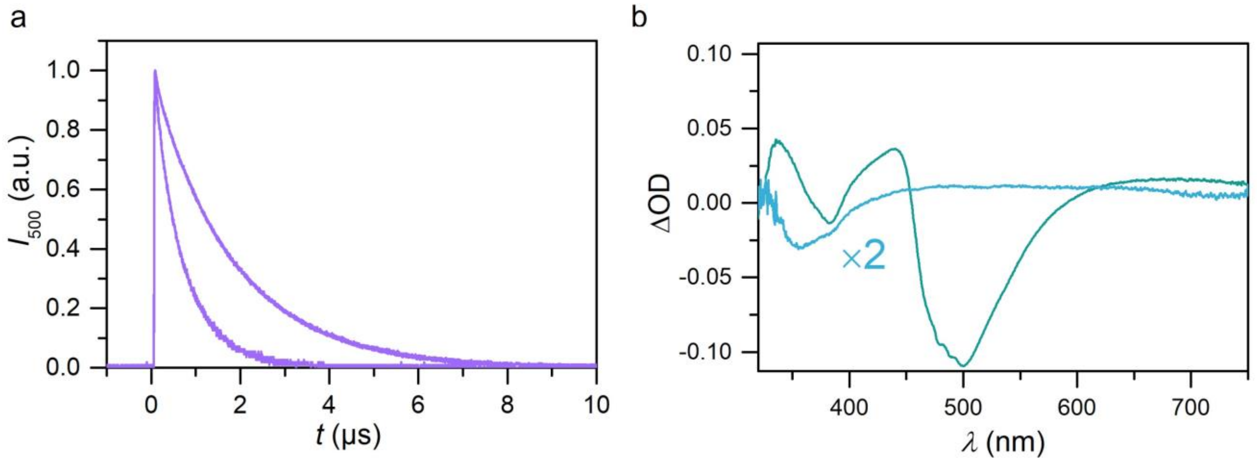

Figure S1. Mechanistic investigations of Ir(dFppy)$_3$ with 1 mM $ArTT^+$ in Ar-saturated acetone after 355 nm laser pulse excitation. **a**, time-resolved emission recorded at 500 nm in the absence and presence of $ArTT^+$. **b**, TA spectra recorded 100 ns (green) and 5 µs (blue) after excitation.

The photocatalyst Ir(dFppy)$_3$ has a triplet energy of 2.75 eV, similar to that of TXO[4]. Kinetic traces monitoring the phosphorescence at 500 nm reveal that the triplet state is deactivated in the presence of 1 mM $ArTT^+$ in argon-saturated acetone (Fig. S1a, quenching efficiency ~63%). However, no signals corresponding to the thianthrenium radical cation were detected post-quenching (Fig. S1b). Instead, a ground-state bleach of the photocatalyst in the 350-400 nm region was observed, usually related to electron transfer processes with Ir-based photocatalysts[5,6]. The reduction of thianthrenium salts by an Ir-photocatalyst, as reported by Yang et al., generates the neutral thianthrene species after cleavage, which is transparent in the selected detection range[7].

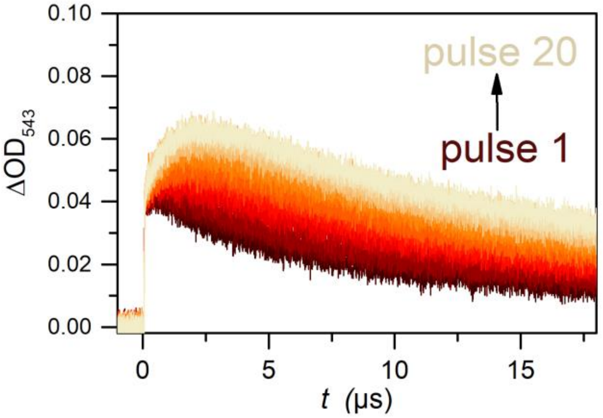

Figure S2. The time-resolved transient absorption at 543 nm of a 1 mM $PhTT^+$ and 50 µM TXO solution in acetone saturated with argon (3 mL in a cuvette with 1 cm path length). Kinetic traces following each 355 nm pulse on the same cuvette, from the first to the 20th pulse, are presented.

Upon repeated excitation of TXO in the presence of thianthrenium salts using 355 nm laser pulses, we observed

accelerated quenching of TXO and the formation of novel signals in the transient absorption spectrum. Exemplary time-resolved measurements at 543 nm are shown for each individual pulse (Fig. S2). We attribute this observation to the rapid degradation of the system due to the high quantum yield of the sensitized reaction and the absence of a scavenging reagent. The irreversible fragmentation of $PhTT^+$ generates a highly reactive aryl radical, while the thianthrenium radical cation eventually reacts with traces of water, forming products known to quench or interact with triplet-excited TXO[8–10]. Consequently, all measurements were recorded with a fresh solution that was replaced after each single-pulse experiment. Relatively high laser pulse intensities had to be used to obtain an acceptable signal-to-noise ratio.

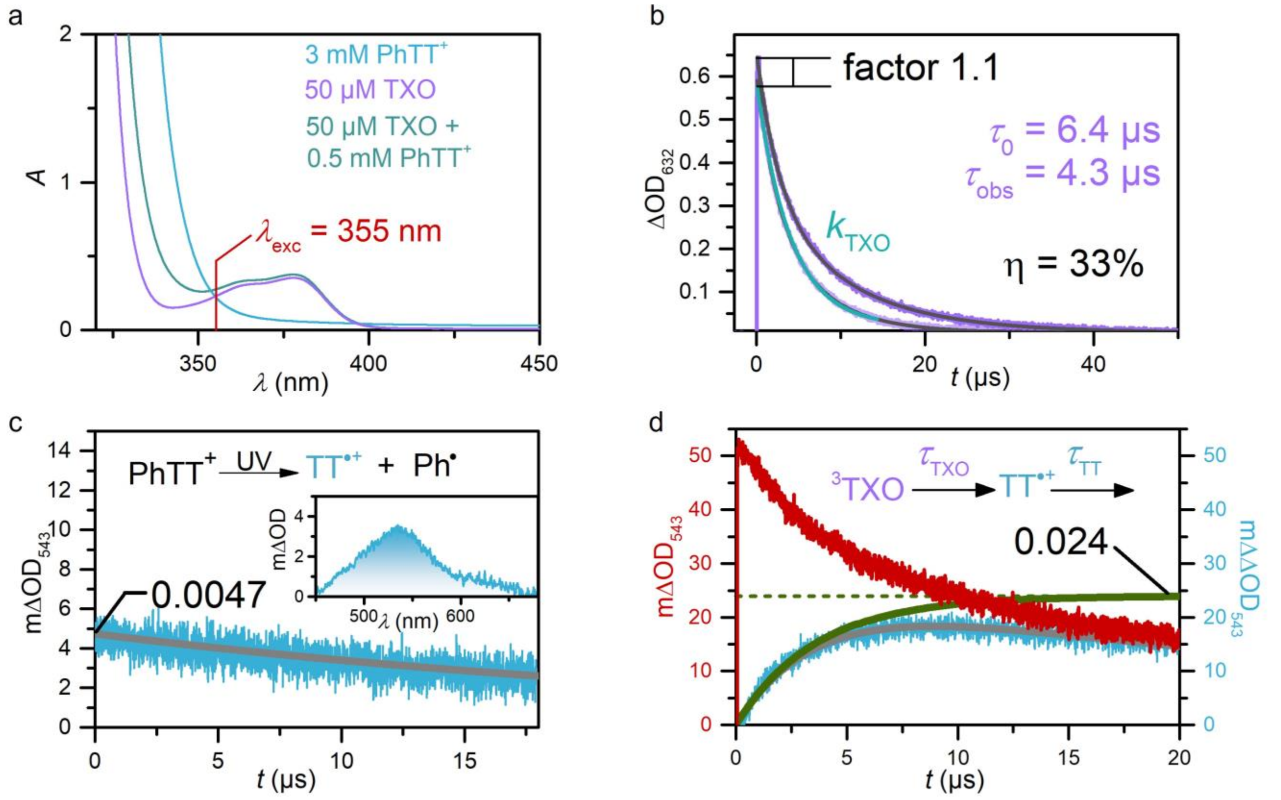

Figure S3. Relative comparison of directly excited and sensitized homolytic cleavage of $PhTT^+$ using 355 nm laser pulses (29.2 mJ) in Ar-saturated acetone. a, absorption spectra of the employed solutions. **b,** time-resolved transient absorption of ^3TXO in the absence and presence of 0.5 mM $PhTT^+$. **c,** kinetic traces of $TT^{\bullet+}$ produced upon direct excitation of 3 mM $PhTT^+$. Inset: TA spectrum recorded after 700 ns. **d,** kinetic traces at 543 nm of 50 µM TXO and 0.5 mM $PhTT^+$ (red), isolated formation and decay of $TT^{\bullet+}$ (cyan), simulated formation of $TT^{\bullet+}$ (green).

The formation of $TT^{\bullet+}$ upon direct excitation and sensitization with ^3TXO was analyzed by relative actinometry. The thianthrenium salts weakly absorb in the UVA range allowing us to directly excite them at 355 nm when high concentrations are employed. Two solutions containing 3 mM $PhTT^+$ or 50 µM TXO were prepared ensuring identical excitation conditions at 355 nm (Fig. S3a) with 29.2 mJ laser pulses. A concentration of 0.5 mM $PhTT^+$ was added to the TXO solution. Quenching studies of TXO in the presence and absence of $PhTT^+$ yielded the triplet energy transfer efficiency for the selected quencher concentration of 0.33 (Fig. S3b). Due to the higher laser intensity (29.2 mJ) and self-quenching effects of ^3TXO the lifetimes τ_0 and τ_{obs} were obtained

from a biexponential fits by averaging τ_1 and τ_2 with the corresponding amplitudes from the fit functions[11]. The weak absorption of $PhTT^+$ at 355 nm leads to a slightly reduced formation of ^{3}TXO after excitation with constant laser intensity, which can be corrected through measurements at 632 nm, where ^{3}TXO can be studied in isolation (Fig. S3b, correction factor of 1.1). The $TT^{•+}$ signal at 543 nm is superimposed with the simultaneous deactivation of ^{3}TXO (Fig. S3d, red curve). A straightforward separation of the kinetic trace at 543 nm is possible with the isolated ^{3}TXO signal recorded at 632 nm and the relative molar absorption coefficients of ^{3}TXO at these two wavelengths[4]. This separation allows us to isolate the $TT^{•+}$ signal (Fig. S3d, cyan curve). Due to the moderate quenching efficiency under our diluted conditions, $TT^{•+}$ already decays to some degree before reaching the maximum signal intensity. Fitting the data to the following equation for A → B → C kinetics enables us to determine the overall $TT^{•+}$ signal.

$$\Delta\mathrm{OD}_{543} = \Delta\mathrm{OD}_{543,\mathrm{max}} \cdot \frac{k_{\mathrm{TXO}}}{k_{\mathrm{TT}} - k_{\mathrm{TXO}}} \left(e^{-k_{\mathrm{TXO}} \cdot t} - e^{-k_{\mathrm{TT}} \cdot t}\right)$$

Where $\Delta OD_{543,max}$ represents the cumulative $TT^{•+}$ signal, k_{TXO} is the quenching rate of ^{3}TXO that was obtained from Fig. S3b and k_{TT} is the pseudo-first-order decay rate of $TT^{•+}$, which can be used to describe the $TT^{•+}$ decay over the short time range of the experiments in Fig. 3d. The fit functions yields k_{TT} 3.04×10^4 s^{-1} corresponding to a lifetime of ~33 µs for $TT^{•+}$ and a signal $\Delta OD_{543,max}$ of 0.024 (Table S5). For comparison, a curve is simulated showing the cumulative concentration of $TT^{•+}$ (Fig. S3d, green curve). The molar absorption coefficients of ^{3}TXO (30 000 M^{-1} cm^{-1} at λ_{max})[12] and $TT^{•+}$ (12 000 M^{-1} cm^{-1} at 543 nm)[13] from the literature enable us to estimate a concentration of 19 µM for ^{3}TXO and 2.2 µM for $TT^{•+}$, giving an overall quantum yield $\Phi_{obs} = 0.12$ for this reaction in the presence of 0.5 mM $PhTT^+$ as ^{3}TXO quencher. The intrinsic quantum yield for homolytic cleavage after sensitization (Φ_{EnT}) of $PhTT^+$ is calculated from the following equation: $\Phi_{obs} = \Phi_{EnT} \times \eta$, where η corresponds to the energy transfer efficiency (0.33), yielding a value of 0.36.

Table S5. Key values obtained for the sensitized fragmentation of $PhTT^+$.

Compound	$m\Delta OD_{max}$	*c* (µM)	*k* (s^{-1})	Φ_{obs}	Φ_{EnT}	η
^{3}TXO	570 [a]	19.0 [c]	2.78×10^5 [e]	0.12	0.36	0.33
$TT^{•+}$	24 [b]	2.0 [c] (2.2)[d]	3.04×10^4			

[a] At 632 nm. [b] At 543 nm. [c] Calculated from literature molar absorption coefficients[12,13]. [d] multiplied with correction factor of 1.1. [e] Obtained from the kinetic measurements in Fig. S3b. The high ^{3}TXO concentrations generated as a result of the high laser intensity causes slight deviations from a monoexponential decay of ^{3}TXO due to self-quenching effects[11]. Hence, a kinetic analyzis with biexpoential fit functions was carried out as explained in the text. The quenching rate k_{TXO} of is therefore determined by fitting only the time window of interest (0-15 µs). (Self-quenching of ^{3}TXO is not a problem when deactivation by a quencher is much more efficient (e.g. quencher concentrations of 50 mM (as employed in the preparative photolysis experiments) and non-pulsed excitation light sources are employed. Hence, under lab-scale conditions the efficiency is likely even higher).

Under identical excitation conditions, the cleavage following direct excitation of $PhTT^+$ was traced at 543 nm (Fig. S3c). A much weaker signal intensity (ΔOD_{543} = 0.0047) is detected directly after excitation. By comparing the relative signal intensities of sensitized and direct formation of $TT^{•+}$ we calculated a quantum yield Φ_{UV} of 0.02. The fraction of the $TT^{•+}$ that is generated by direct excitation of $PhTT^+$ in the solution containing 50 µM and 0.5 mM $PhTT^+$ can be neglected, since the signal is below the detection limit and has no effect on the final result. The absolute quantum yields crucially depend on the molar absorption coefficients as explained above. These values were obtained in different solvents but the TA spectra of both ^{3}TXO and $TT^{•+}$ are largely solvent independent. We therefore assume that the molar absorption coefficients of these species in acetone are similar as well (expected maximum relative error, 25 %). However, we stress that the relative quantum yield improvement of the EnT approach compared to direct UV excitation (by a factor of 18) does not depend on the molar absorption coefficients.

Analysis of energy transfer rate and expected timescales

The quenching of TXO by $ArTT^+$ (**1**) was observed with an energy transfer rate of $k_q = 5.0 \times 10^7$ M^{-1} s^{-1} at 20 °C. The energy transfer rate is indeed quite rapid, and based on the viscosity parameters of acetone, we estimated the quenching efficiencies at –78°C. The viscosity of acetone is reported as 0.323 mPa·s at 20 °C and 1.43 mPa·s at -78 °C (https://www.ddbst.com/ddb.html). We assume that the energy transfer rate constant is inversely proportional to the viscosity of the solvent.

$$\text{Energy transfer rate} \quad k_\mathrm{q} \sim \frac{1}{\eta}$$

We calculated k_q (-78°C) to be approximately 1.1×10^7 M^{-1} s^{-1}, which was used to estimate the quenching efficiency alongside the triplet lifetime of TXO at 20°C (t = 8.85 µs). Even at the low temperatures employed in the reaction, we estimate a quenching efficiency of η_{EnT} = 83% (c_q = 0.05 M) at the start of the reaction and η_{EnT} = 33% at 90% conversion (c_q = 0.005 M) (see equation below). This indicates that highly efficient quenching is still achievable at -78°C.

$$\text{Quenching efficiency} \quad \eta_{\mathrm{EnT}} = \frac{\tau_0 - \left(\frac{\tau_0}{1+k_\mathrm{q}\cdot c_\mathrm{q}\cdot \tau_0}\right)}{\tau_0}$$

Since a nearly identical setup was used for both the quantum yield determination and the irradiation experiments, we can estimate the reaction times by simply comparing the amount of substance with the photon flux, which was also determined through ferrioxalate actinometry (1.26 10^{-6} Einstein s^{-1}). In this estimation, we account for the fact that the LED power was set to 25% for the quantum yield determination and 100% for the general procedure. Additionally, the volume increases from 1 mL to 4 mL, resulting in a fourfold increase in the irradiated volume.

$$\text{Reaction time} \quad t = \frac{n_{\mathrm{ArTT+}}}{photon\ flux \cdot \Phi \cdot f} \sim \frac{0.200\ \mathrm{mmol}}{(4 \cdot 4 \cdot 1.26 \cdot 10^{-6}\ \mathrm{Einstein\ s^{-1}}\ )\cdot 0.1 \cdot 0.94} \sim \frac{0.200\ \mathrm{mmol}}{2 \cdot 10^{-5}\ \mathrm{Einstein\ s^{-1}} \cdot 0.1 \cdot 0.94} \sim 106\ \mathrm{s}$$

The fraction of absorbed light by TXO (f = 0.94) is identical to the conditions in the quantum yield determination. The estimated reaction time (106 s) for the general procedure aligns well with the

experimentally observed irradiation time of 120 seconds. This straightforward calculation does not account for the gradual decrease in energy transfer efficiency toward the end of the reaction, as mentioned earlier. However, this has only a minimal impact on the overall irradiation time.

In conclusion, the short reaction times are not indicative of a chain reaction mechanism but are consistent with the estimated and expected duration for highly efficient energy transfer reactions.

UV-vis absorption spectrum of reaction mixtures

As shown in Figure S4 and S5, interaction between TXO and $ArTT^+$ **1** at ground states was not observed under the reaction concentrations.

All UV-Vis spectrum measurements were recorded on a Shimadzu UV-Vis spectrophotometer UV-2600 with temperature controller using a quartz cuvette (10 × 10 mm, 3.5 mL) in acetone as solvent.

UV-Vis spectra of the reaction mixture were measured with the following concentrations:

TXO (2.1 mg, 10 μmol, c = 0.5 mM) in acetone (20 mL);

$ArTT^+$ **1** (490 mg, 1.0 mmol, c = 50 mM) in acetone (20 mL);

TXO (2.1 mg, 10 μmol, c = 0.5 mM) and $ArTT^+$ **1** (490 mg, 1.0 mmol, c = 50 mM) in acetone (20 mL);

TXO (2.1 mg, 10 μmol, c = 0.5 mM) and $ArTT^+$ **1** (196 mg, 0.4 mmol, c = 20 mM) in acetone (20 mL);

TXO (2.1 mg, 10 μmol, c = 0.5 mM) and $ArTT^+$ **1** (98 mg, 0.2 mmol, c = 10 mM) in acetone (20 mL);

TXO (2.1 mg, 10 μmol, c = 0.5 mM) and $ArTT^+$ **1** (49 mg, 0.1 mmol, c = 5 mM) in acetone (20 mL);

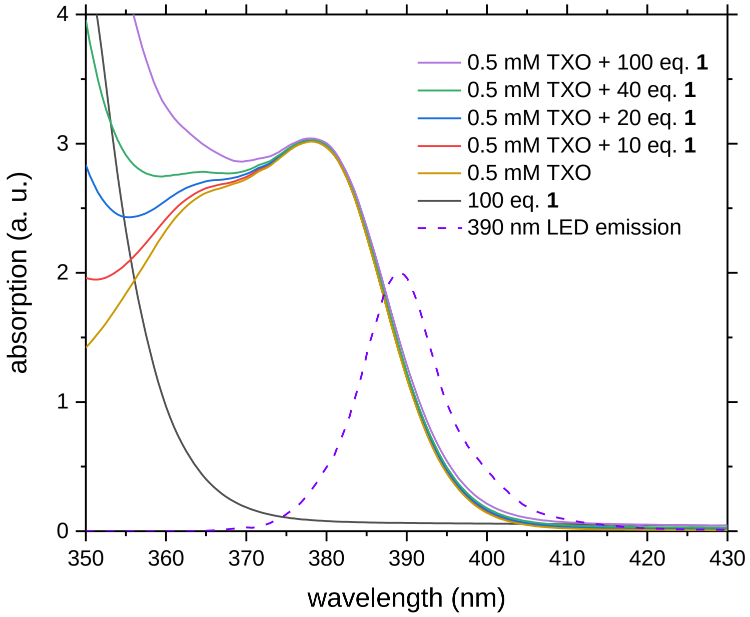

Figure S4. UV-Vis absorption spectra of $ArTT^+$ **1** and TXO mixtures in various ratio in acetone.

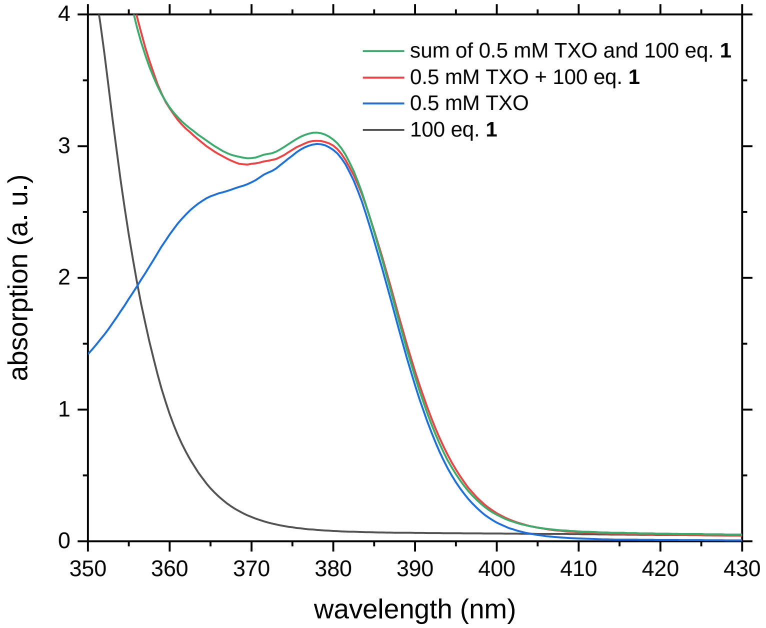

Figure S5. UV-Vis absorption spectra of TXO and ArTT$^+$ **1** mixtures in acetone, and the mathematical summation of individual spectra

Cyclic voltammograms

Determination of redox potentials of TXOs at ground state

Cyclic voltammograms were recorded using an Autolab PGSTAT204 potentiostat and a Pt working electrode, an Ag/AgCl reference electrode and a Pt wire auxiliary electrode. The voltammograms were recorded at room temperature in 0.1 M tetrabutylammonium hexafluorophosphate in acetone (3 mL, c = 3.3 mM) containing 2-substituted TXO (10 μmol). The scan rate was 100 mV·s^{-1}.

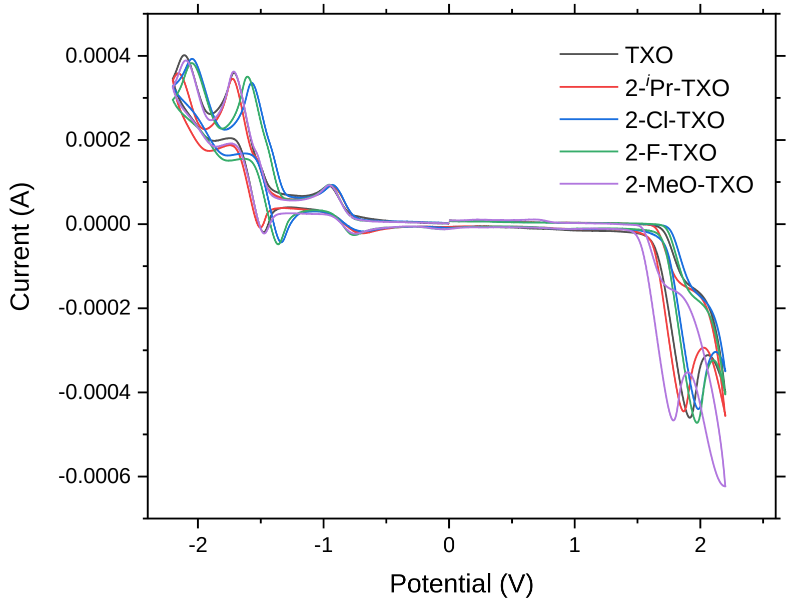

Figure S6. Cyclic voltammetry of 2-substituted thioxanthones in acetone (c = 3.3 mM).

Determination of redox potentials of TXOs at excited state

The excited state redox potentials of thioxanthones were calculated through the equations below using the ground state potentials above and the calculated triplet energy in this study (see page S69, Table S13).

$E_{p/2}\,(PC^{*}/PC^{-}) = E_{p/2}\,(PC/PC^{-}) + E_T$

$E_{p/2}\,(PC^{+}/PC^{*}) = E_{p/2}\,(PC^{+}/PC) - E_T$

Table S6. Summary of reduction potentials for 2-substituted thioxanthones at ground and triplet states

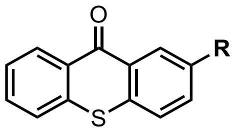

entry	R	E_T/eV	$E_{p/2}(PC/PC^{-})$	$E_{p/2}(PC^{+}/PC)$	$E_{p/2}(PC^{+}/PC^{*})$	$E_{p/2}(PC^{*}/PC^{-})$
1	**2-H (TXO)**	2.80	–1.60 V	1.81 V	–0.99 V	1.20 V
2	**2-**^{i}Pr**	2.75	–1.62 V	1.75 V	–1.00 V	1.13 V
3	**2-Cl**	2.71	–1.46 V	1.87 V	–0.84 V	1.25 V
4	**2-F**	2.68	–1.60 V	1.84 V	–0.84 V	1.08 V
5	**2-MeO**	2.55	–1.72 V	1.65 V	–0.90 V	0.83 V

Relationship between reaction rates and triplet energy or redox potentials

Kinetic reaction profile of reactant 1 with 1.0 mol% 2-substituted thioxanthones.

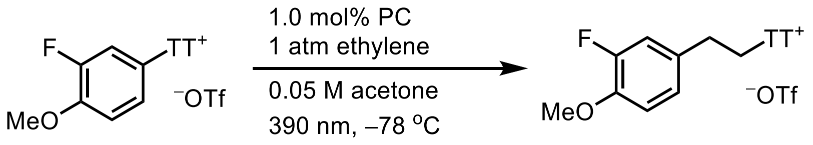

1 **2**

To a 5-mL Schlenk tube containing a Teflon-coated magnetic stirring bar were added arylthianthrenium salt **1** (50 mg, 0.10 mmol, 1.0 equiv) and 2-substituted thioxanthone in acetone (c = 0.50 mM, 2.0 mL, 1.0 μmol, 1.0 mol%). The tube was sealed with a rubber septum and immersed in a dry ice/acetone bath at –78°C. A balloon filled with ethylene gas was connected to a long needle, which was inserted to the tube through the rubber septum. Roughly 100 ml of ethylene gas was bubbled through the solution over a time period of approximately 5 minutes, and excess gas was released through the side arm, all while avoiding exposure to light. The valve of the side arm was closed, and the needle was withdrawn from the solution but remained in the headspace of the vessel. The reaction mixture was then subjected to irradiation by one Kessil PR160-390 nm LED with 100% intensity (40 W, ~6 cm away), all while maintaining the bath temperature at –78°C. After

being irradiated for indicated time, the light was turned off, and approximately 0.2 mL reaction mixture was extracted, diluted with 0.4 mL $CDCl_3$, and analyzed by ^{19}F NMR with triflate counterion as internal standard. The change in concentration of starting material **1** was determined as follow:

Table S7. Concentration of $ArTT^+$ **1** as a function of time with different 2-substituted thioxanthone as photosensitizer

Time (s)	TXO (M)	Time (s)	2-^{i}Pr-TXO (M)	Time (s)	2-Cl-TXO (M)	2-F-TXO (M)	2-OMe-TXO (M)
**0**	0.050	**0**	0.050	**0**	0.050	0.050	0.050
**10**	0.045	**30**	0.046	**60**	0.045	0.049	0.050
**20**	0.038	**60**	0.041	**120**	0.037	0.048	0.050
**30**	0.033	**90**	0.036	**180**	0.027	0.043	0.050
**40**	0.028	**120**	0.031	**240**	0.017	0.039	0.049
**50**	0.023	**150**	0.024	**300**	0.012	0.034	0.049

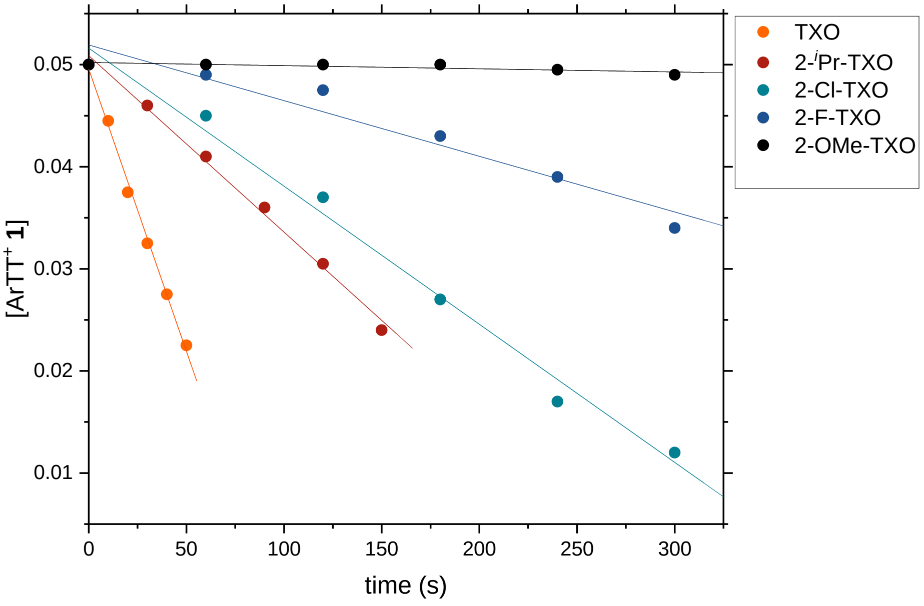

Figure S7. Concentration of $ArTT^+$ **1** as a function of time with different 2-substituted thioxanthone as photosensitizer

Relationship between reaction rates and triplet energy or redox potentials

From the kinetic reaction profile, it was observed that $ArTT^+$ **1** exhibited zero-order kinetics. The initial rate can be directly determined by the slope of the kinetic curve.

Table S8. Summary of initial rates with different 2-substituted thioxanthone as photosensitizer

PC	Initial rate ($mol•L^{-1}•s^{-1}$)
**TXO**	5.53×10^{-4}
**2-^{i}Pr-TXO**	1.73×10^{-4}
**2-Cl-TXO**	1.35×10^{-4}
**2-F-TXO**	0.55×10^{-4}
**2-OMe-TXO**	0.03×10^{-4}

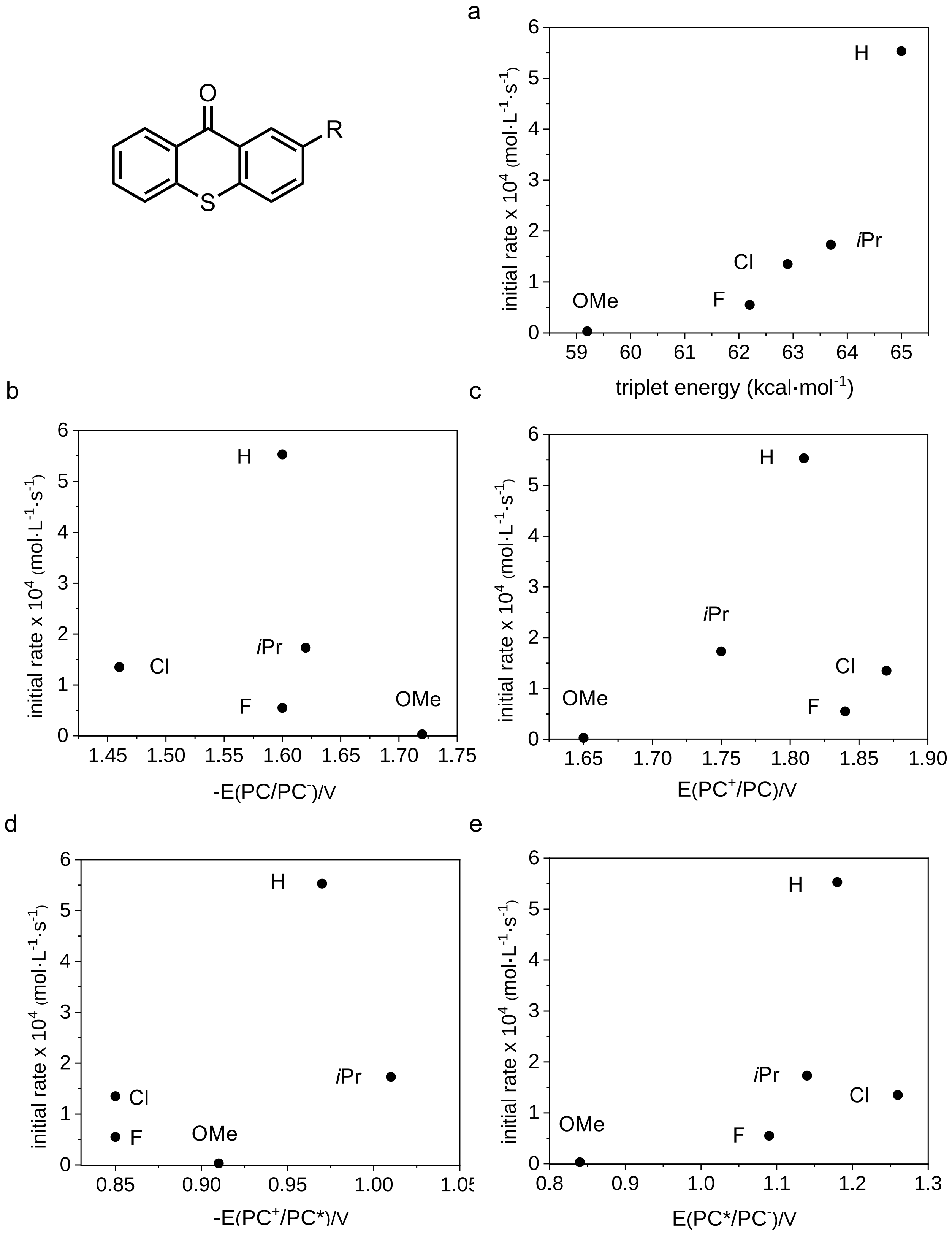

Figure S8. Correlation analysis: reaction rates vs triplet energy or redox potentials; Data sourced from Table S6 and S8.

Quantum yield measurement

Determination of the light intensity of Kessil PR160-390nm LEDs

Standard ferrioxalate actinometry[14,15] was used to determine the radiant flux of Kessil PR160-390 nm LED with 25% intensity. A solution of ferrioxalate (0.15 M) was prepared by dissolving potassium ferrioxalate trihydrate (737 mg, 1.50 mmol) in 10.0 mL of 0.20 M aqueous H_2SO_4. A buffered solution of 1,10-phenanthroline (0.15 M) was prepared by dissolving NaOAc (1.23 g, 15.0 mmol) and 1,10-phenanthroline (541 mg, 3.00 mmol) in 20 mL of 0.20 M aqueous H_2SO_4.

To a 4-mL borosilicate vial equipped with a stir bar was added 1.0 mL of the ferrioxalate solution. The vial was sealed with a septum-cap and placed 6 cm away from one Kessil PR160-390 nm LED with 25% intensity (10 W). After being irradiated for 10 seconds, 2.0 mL of the buffered solution was added. The resulting mixture was then allowed to rest for 1 hour to allow the formed ferrous ions to react completely with 1,10-phenanthroline. The mixture was diluted to 14.0 mL with 0.20 M aqueous sulfuric acid. An aliquot (100 µL) of the resulting solution was diluted with 3.0 mL of 0.20 M aqueous sulfuric acid, and the absorbance in a cuvette (l = 1.0 cm) at 510 nm was measured by UV-Vis spectrometer. The above procedure was repeated with different irradiation time, and the average absorption was used for the calculation of photon flux. A non-irradiated sample was also prepared and the absorbance at 510 nm was measured.

Table S9. Calculation of radiant flux

Irradiation time (s)	Absorbance	ΔA	mol Fe^{2+}(mol)	photo flux (Einstein/s)
non-irradiation	0.027	-	-	-
10	0.398	0.371	1.40×10^{-5}	1.24×10^{-6}
20	0.818	0.791	2.99×10^{-5}	1.32×10^{-6}
30	1.198	1.171	4.43×10^{-5}	1.31×10^{-6}

The photon flux was calculated as follows:

$$\text{mol Fe}^{2+} = \frac{V \times \Delta A\ (510\ nm)}{l \times \varepsilon}$$

where V is the total volume (0.42 L) of the solution that was analyzed, ΔA is the difference between the average absorption of irradiated and non-irradiated solutions at 510 nm, l is the path length (1.00 cm), and ε is the molar absorptivity of the ferrioxalate actinometer at 510 nm (11,100 L mol^{-1} cm^{-1}).

$$\text{photon flux} = \frac{\text{mol Fe}^{2+}}{\Phi \times t \times f}$$

where Φ is the quantum yield for the ferrioxalate actinometer (approximated as 1.13[16], which was reported for a 0.15 M solution at λ = 392 nm), t is the irradiation time, and f is the fraction of light absorbed at λ = 390 nm by the ferrioxalate actinometer. This value is calculated using the following equation where A(390 nm) is the measured absorbance of the 0.15 M solution of potassium ferrioxalate at 390 nm. An absorption spectrum gave

an A(390) value of >3, indicating the fraction of absorbed light (f) is >0.999.

$$f = 1 - 10^{-A(390\ nm)}$$

The average photon flux was thus calculated to be 1.29 × 10^{-6} einsteins s^{-1}

Determination of quantum yield

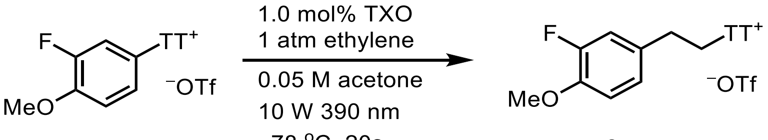

1 **2**, 4.4%

To a 5-mL Schlenk tube containing a Teflon-coated magnetic stirring bar were added arylthianthrenium salt **1** (25 mg, 0.050 mmol, 1.0 equiv) and thioxanthone in acetone (c = 0.50 mM, 1.0 mL, 0.50 μmol, 1.0 mol%). The tube was sealed with a rubber septum and immersed in a dry ice/acetone bath at –78°C. A balloon filled with ethylene gas was connected to a long needle, which was inserted to the tube through the rubber septum. Roughly 100 ml of ethylene gas was bubbled through the solution over a time period of approximately 5 minutes, and excess gas was released through the side arm, all while avoiding exposure to light. The valve of the side arm was closed, and the needle was withdrawn from the solution but remained in the headspace of the vessel. The reaction mixture was then subjected to irradiation by one Kessil PR160-390 nm LED with 25% intensity (10 W, ~6 cm away), all while maintaining the bath temperature at –78°C. After being irradiated for 20 s, the reaction mixture was warmed to room temperature. 1,3,5-Trimethoxybenzene (50 μL, 0.050 mmol, 1.0 M in MeCN, 1.0 equiv) was added as an internal standard. The ^{1}H NMR yield of product **2** was determined to be 4.4%.

The quantum yield was calculated as follows:

$$\Phi = \frac{\text{mol product}}{\text{photon flux} \times t \times f}$$

where mol product is the product **2** formation rate (1.1 × 10^{-7} mol/s), photon flux is determined by above ferrioxalate actinometry (1.29 × 10^{-6} einsteins s^{-1}), and f is the fraction of light absorbed by the reaction mixture at 390 nm. This value is calculated using the following equation where *A*(390 nm) is the absorption of the non-irradiated reaction mixture (1.29) at 390 nm. The fraction of light absorbed at 390 nm was calculated:

$$f = 1 - 10^{-A(390\ nm)} = 1 - 10^{-1.29} = 0.949$$

Thus, the quantum yield was calculated as Φ = 0.1.

Reaction in the presence of triplet quencher

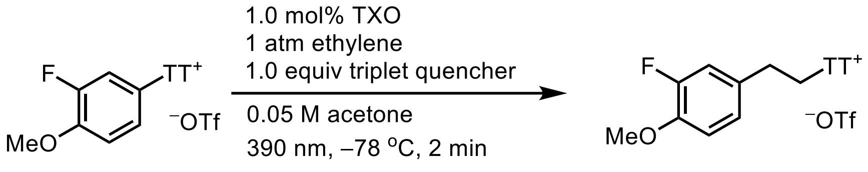

1 **2**

To a 5-mL Schlenk tube containing a Teflon-coated magnetic stirring bar were added arylthianthrenium salt **1** (25 mg, 0.050 mmol, 1.0 equiv)), triplet quencher (0.050 mmol, 1.0 equiv), and thioxanthone in acetone (c = 0.50 mM, 1.0 mL, 0.50 μmol, 1.0 mol%). The tube was sealed with a rubber septum and immersed in a dry ice/acetone bath at –78°C. A balloon filled with ethylene gas was connected to a long needle, which was inserted to the tube through the rubber septum. Roughly 100 ml of ethylene gas was bubbled through the solution over a time period of approximately 5 minutes, and excess gas was released through the side arm, all while avoiding exposure to light. The valve of the side arm was closed, and the needle was withdrawn from the solution but remained in the headspace of the vessel. The reaction mixture was then subjected to irradiation by one Kessil PR160-390 nm LED with 25% intensity (10 W, ~6 cm away), all while maintaining the bath temperature at –78°C. The reaction mixture was irradiated for 2 minutes, all while maintaining the temperature at –78°C. Subsequently, 1,3,5-trimethoxybenzene (50 μL, 0.050 mmol, 1.0 M in MeCN, 1.0 equiv) was added as internal standard. The reaction mixture was concentrated and analyzed by ^{1}H NMR spectroscopy.

Table S10. Reaction results in the presence of triplet quenchers

triplet quencher	structure	E_T (kcal•mol^{-1})	conv. (%)	yield (%)
anthracene	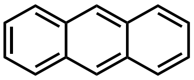	42.6	29	8
cyclooctatetraene	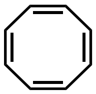	51.3	<2	<2

Radical scrambling experiment

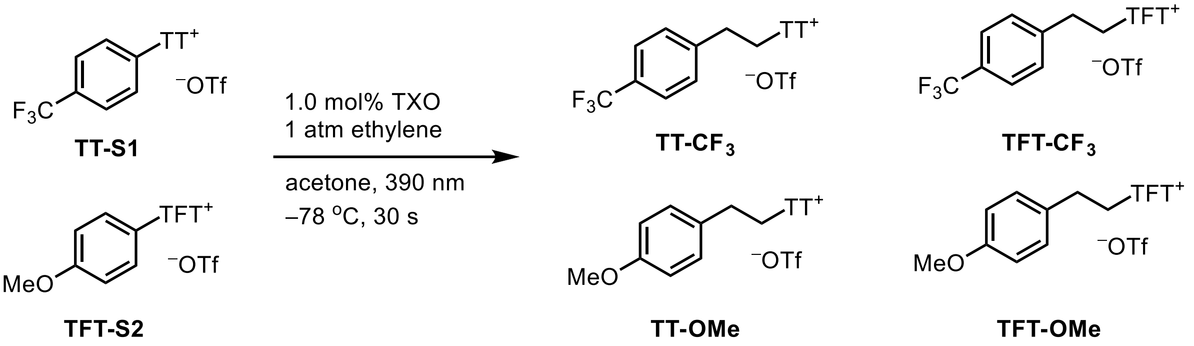

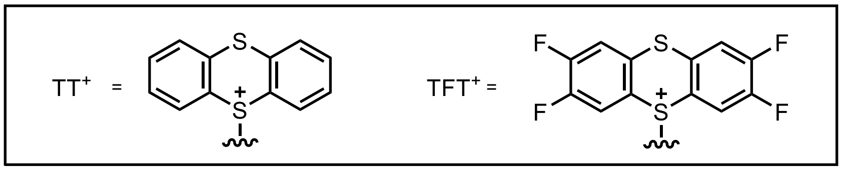

To a 5-mL Schlenk tube containing a Teflon-coated magnetic stirring bar were added **TT-S1** (26 mg, 0.050 mmol, 1.0 equiv), **TFT-S2** (27 mg, 0.050 mmol, 1.0 equiv), and thioxanthone in acetone (c = 0.50 mM, 2.0 mL, 1.0 µmol, 1.0 mol%). The tube was sealed with a rubber septum and immersed in a dry ice/acetone bath at –78°C. A balloon filled with ethylene gas was connected to a long needle, which was inserted to the tube through the rubber septum. Roughly 100 ml of ethylene gas was bubbled through the solution over a time period of approximately 5 minutes, and excess gas was released through the side arm, all while avoiding exposure to light. The valve of the side arm was closed, and the needle was withdrawn from the solution but remained in the headspace of the vessel. The reaction mixture was then subjected to irradiation by one Kessil PR160-390 nm LED with 100% intensity (40 W, ~6 cm away) for 30 seconds, all while maintaining the bath temperature at –78°C. Then the reaction mixtures was warmed to room temperature and measured by high-resolution mass spectrometry (HRMS). All four arylethyl (tetrafluoro)thianthrenium salts were detected.

HRMS-ESIpos (m/z) of **TT-CF$_3$** calc'd for $C_{21}H_{16}F_3S_2$ [M–OTf]$^+$, 389.0640; found, 389.0644; deviation: –1.0 ppm.

HRMS-ESIpos (m/z) of **TT-OMe** calc'd for $C_{21}H_{19}OS_2$ [M–OTf]$^+$, 351.0872; found, 351.0877; deviation: –1.4 ppm.

HRMS-ESIpos (m/z) of **TFT-CF$_3$** calc'd for $C_{21}H_{12}F_7S_2$ [M–OTf]$^+$, 461.0263; found, 461.0265; deviation: –0.4 ppm.

HRMS-ESIpos (m/z) of **TFT-OMe** calc'd for $C_{21}H_{15}F_4OS_2$ [M–OTf]$^+$, 423.0495; found, 423.0495; deviation: +0.1 ppm.

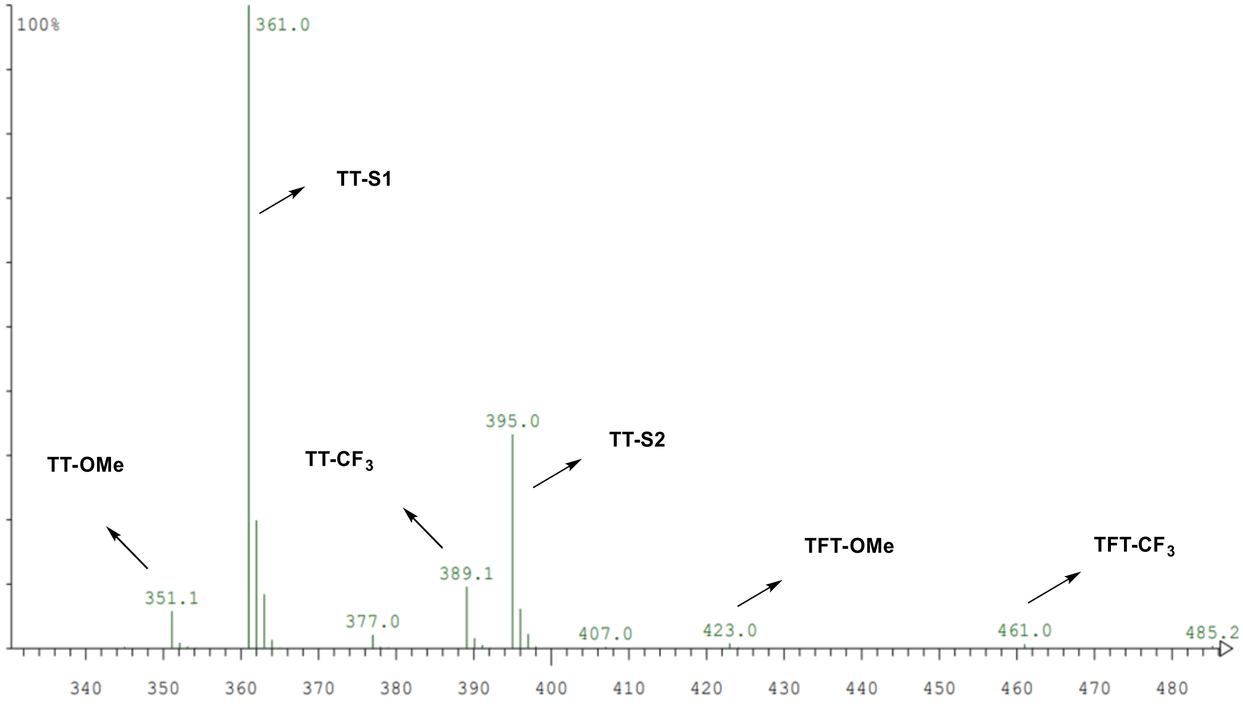

Figure S9. MS spectrum of reaction mixture.

Hammett plot

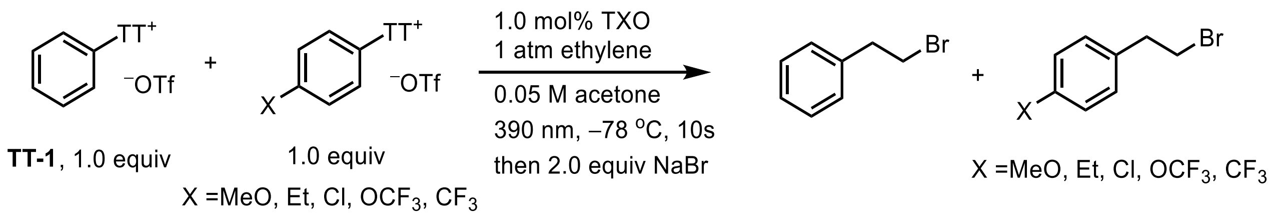

To a 5-mL Schlenk tube containing a Teflon-coated magnetic stirring bar were added arylthianthrenium salt **TT-1** (11.1 mg, 0.025 mmol, 1.00 equiv), *para*-substituted arylthianthrenium salt (0.025 mmol, 1.00 equiv), and thioxanthone (1.0 mL, 0.50 mM in acetone, 0.5 μmol, 1.0 mol%). The tube was sealed with a rubber septum and immersed in a dry ice/acetone bath at –78°C. A balloon filled with ethylene gas was connected to a long needle, which was inserted to the tube through the rubber septum. Roughly 100 ml of ethylene gas was bubbled through the solution over a time period of approximately 5 minutes, and excess gas was released through the side arm, all while avoiding exposure to light. The valve of the side arm was closed, and the needle was withdrawn from the solution but remained in the headspace of the vessel. The reaction mixture was then subjected to irradiation by one Kessil PR160-390 nm LED with 100% intensity (40 W, ~6 cm away) for 10 seconds to have a conversion of 10–30%. Subsequently, internal standard 1,3,5-trimethoxybenzene (50 μL, 0.050 mmol, 1.0 M in MeCN, 1.0 equiv) and tetrabutylammonium bromide (16 mg, 0.050 mmol, 2.0 equiv) were added and the resulting mixture was stirred for 10 minutes. The solution was concentrated and analyzed by ^{1}H NMR spectroscopy. The bromination process exhibits notable cleanliness, facilitating determination of product ratios through integration of characteristic proton signals at the benzylic

position of the arylethyl bromides. The values of log(k_X/k_H) were plotted versus the σ.

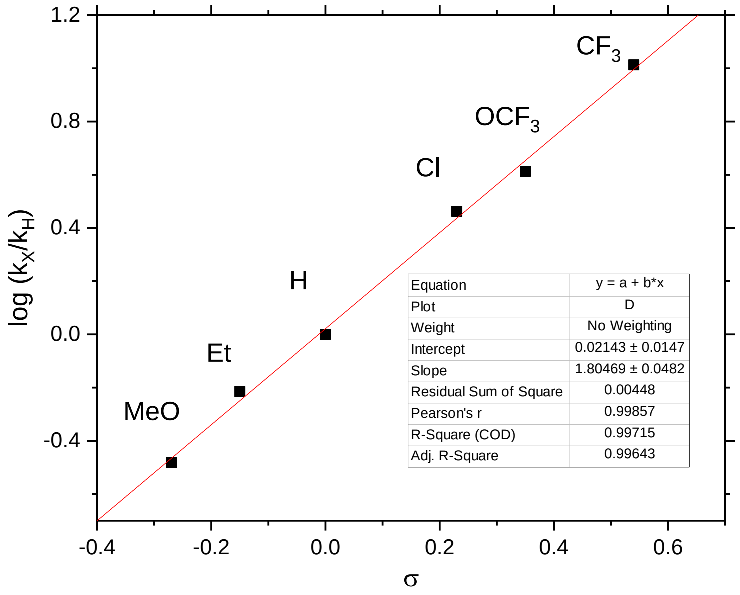

Figure S10. Hammett correlation using the σ-value of the para-substituent. ρ = 1.8, R^2 = 0.99.

DFT CALCULATIONS

Methods

Density Functional Theory (DFT) calculations were performed on the Max-Planck-Institut für Kohlenforschung computer cluster using the ORCA 5.0 program package (Version 5.0 Stable)[17]. Structural optimizations and frequency calculations were performed with the B3LYP functional[18] with D3 dispersion correction[19] and Becke-Johnson damping (BJ),[20] utilizing the def2/J auxiliary basis set[21] and the def2-TZVPP basis set[22] on all atoms. Tight SCF convergence and geometry optimization criterions were chosen. Frequency calculations at the same level had been performed to confirm each stationary point to be either a minimum or a transition structure. Solvent effects of acetone were taken into account using the conductor-like polarized continuum model (CPCM).[23] Time-dependent density functional theory (TD-DFT) was used to calculate the triplet energies for 2-substituted thioxanthone derivatives.[2,24] Molecular orbital energies and pictures were generated through the LargePrint command. Input files were created using Avogadro 1.2[25] and images were generated using Chemcraft 1.8.[26]

Comparison with reported data

Before evaluating the triplet energies of the molecules, we compared the structural properties of the structure obtained by DFT calculations with the reference XRAY structure.[27] As shown in Table S11, the structure (without counteranion) obtained from DFT calculations and the XRAY structure of the aryl thianthrenium cation as a triflate salt **1** shows comparable bond lengths, bond angles, and overall flagship-confirmation.

Table S11. Exemplary bond length and angle comparison between XRAY structure of $PhTT^+$ and DFT structure at CPCM(acetone)-B3LYP-D3BJ/def2-TZVPP level of theory.

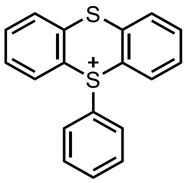

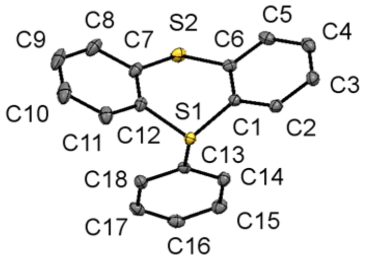

Bond length (Å) in	XRAY structure	DFT structure	Difference
S(1)–C(1)	1.764	1.764	0.000
S(1)–C(13)	1.805	1.805	0.000
S(2)–C(6)	1.755	1.762	0.007
**Bond angle (°) in**	**XRAY structure**	**DFT structure**	**Difference**
C(12)–S(1)–C(1)	103.05	103.05	0.00

C(6)–S(2)–C(7)	103.22	102.49	-0.73
S(2)–C(7)–C(12)	123.94	123.60	-0.34
S(1)–C(12)–C(7)	121.12	120.31	-0.81
S(1)–C(13)–C(14)	118.69	118.83	0.14
C(12)–S(1)–C(13)	102.99	103.32	0.33

To ensure calculations were carried out with a high enough computational level in replicating triplet state molecules, we conducted further evaluations of the triplet energies of known compounds.[28] As illustrated in Table S12 and Figure S11, the experimental values align closely with computational estimations, demonstrating both qualitative and quantitative agreement.

Triplet energies were determined by calculating the energy difference between the optimized singlet ground state structure and the first excited optimized triplet state structure.

Table S12. Comparative analysis: experimental triplet energies vs. computed values for arenes and biphenyl halides at CPCM(acetone)-B3LYP-D3BJ/def2-TZVPP level of theory.

**Compounds**	**Experimental E_T[18] (kcal•mol^{-1})**	**DFT calculated E_T (kcal•mol^{-1})**	**Difference (kcal•mol^{-1})**
benzene	84.4	82.4	-2.0
anisole	80.8	77.5	-3.3
biphenyl	65.5	64.5	-1.0
biphenyl chloride	63.6	62.9	-0.7
biphenyl bromide	n.a.	62.9	-
biphenyl iodide	62.9	62.4	-0.5

n.a. not available

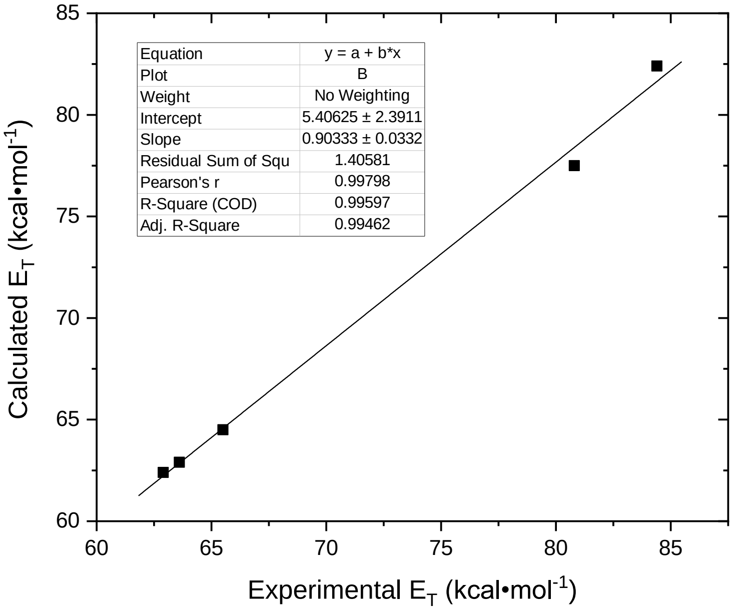

Figure S11. Correlation analysis: experimental triplet energies vs computed values

The triplet energies of TXOs were determined through time-dependent density functional theory (TD-DFT) calculations.[2,24] As detailed in Table S13, our computational estimations exhibited close agreement with both Booker-Milburn's experimental data and their computational values.[2]

Table S13. Comparison analysis: experimental triplet energies vs computed values for TXOs at CPCM(acetone)-B3LYP-D3BJ/def2-TZVPP level of theory.

Substituted TXO	Reported experimental E_T (kcal•mol^{-1})[2]	Reported calculated E_T (kcal•mol^{-1})[2]	Current calculated E_T (kcal•mol^{-1})
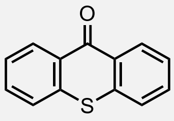	65.5	65.5	65.0
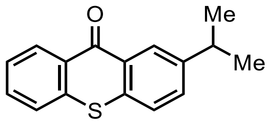	63.5	64.5	63.7
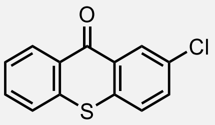	n.a.	n.a.	62.9
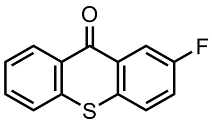	62.4	62.9	62.2

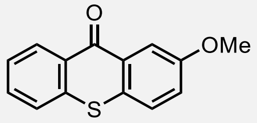	57.8	60.2	59.2

n.a. not available

Visualization of frontier molecular orbitals and triplet energy of arylpseudohalides

As indicated in Table S14, in the calculated triplet state of $PhTT^{+}$, the geometry of the TT framework changes from folded to near-planar, indicating an altered electron configuration of TT. Both the HOMO and LUMO in the ground state and the α-HOMO and α-HOMO-1 in the triplet state of $PhTT^{+}$ are predominantly situated on the TT framework rather than on the aryl ring. Introduction of an electron-donating group to the phenyl ring causes the HOMO and α-HOMO-1 to shift from TT to the aryl group, while maintaining a near-planar geometry of TT in the triplet state. Conversely, introduction of an electron-withdrawing group to the exocyclic phenyl ring leads to the LUMO and α-HOMO shifting from TT to the aryl group, resulting in a folded geometry of TT in the triplet state.

Table S14. Visualization of frontier molecular orbitals and triplet energy of arylpseudohalides

**ArX** (E_T/kcal•mol^{-1})	**Ground state**		**Triplet state**	
	HOMO	LUMO	α-HOMO-1	α-HOMO
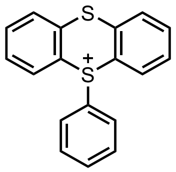 **(65.5)**	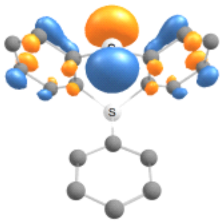	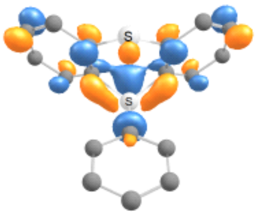	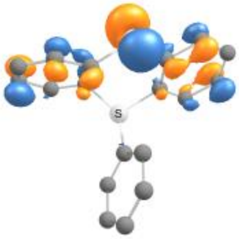	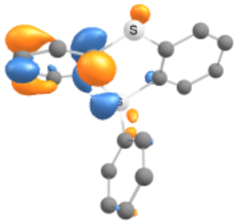
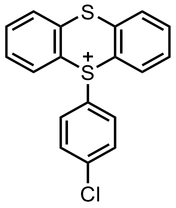 **(65.2)**	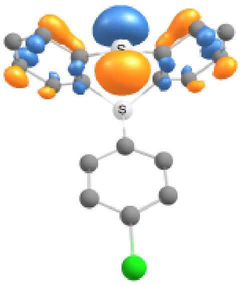	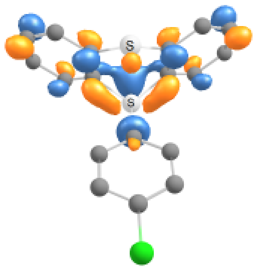	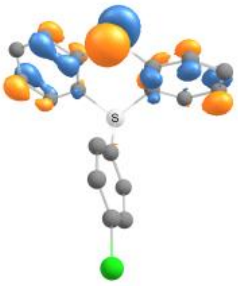	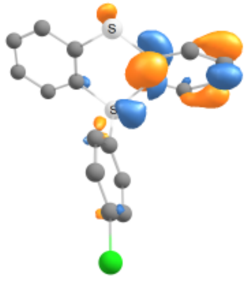

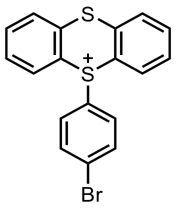

(65.3)

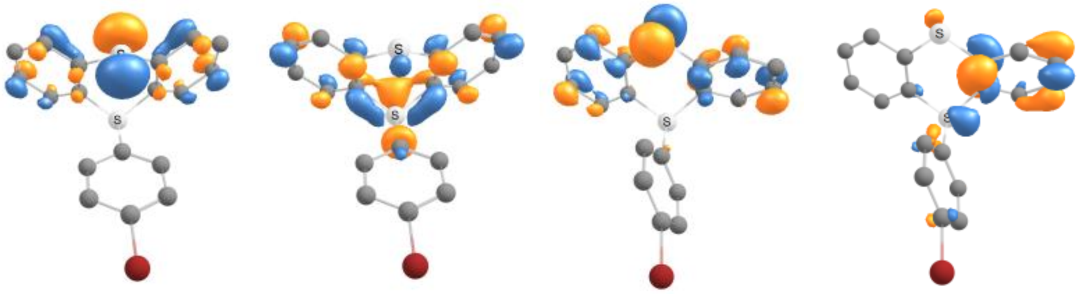

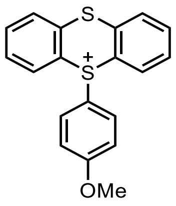

(65.6)

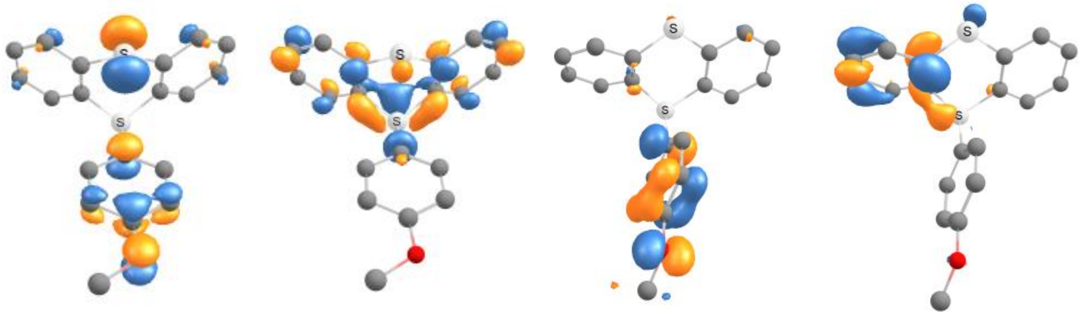

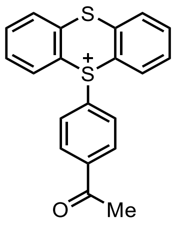

(63.3)

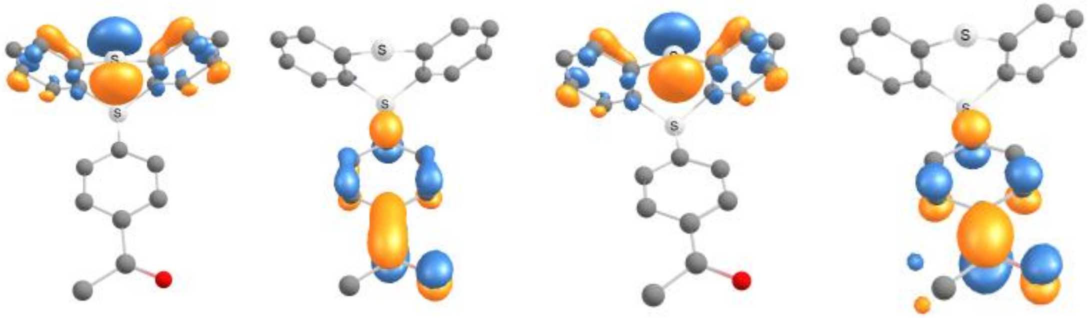

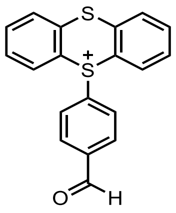

(62.0)

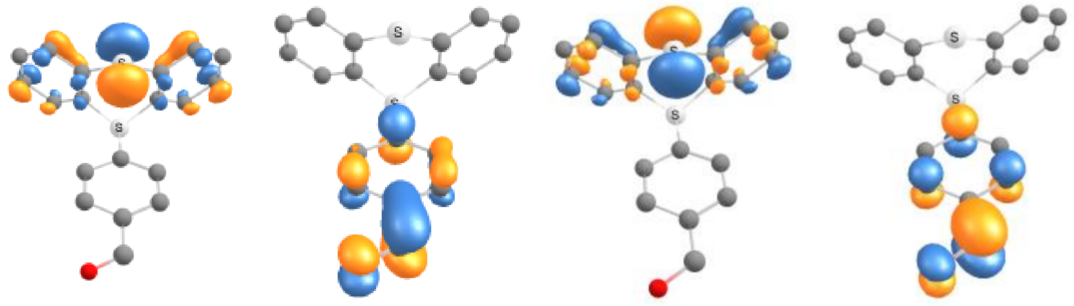

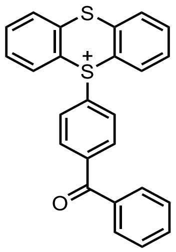

(60.3)

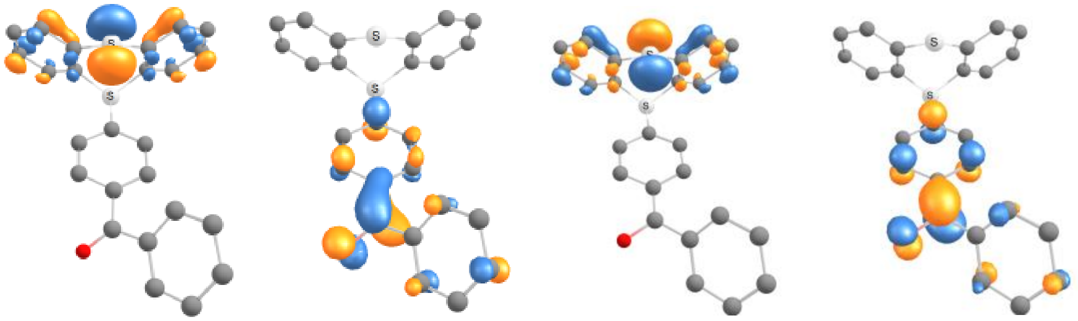

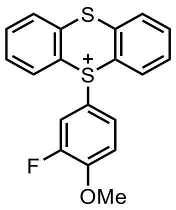

(65.6)

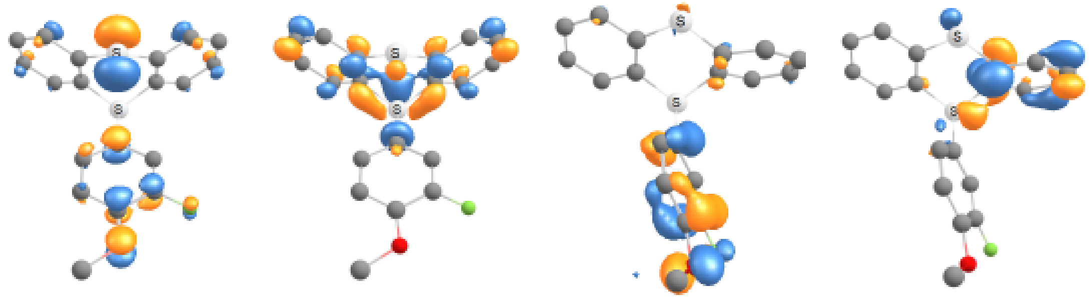

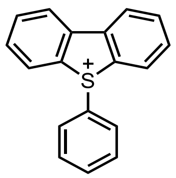

(61.7)

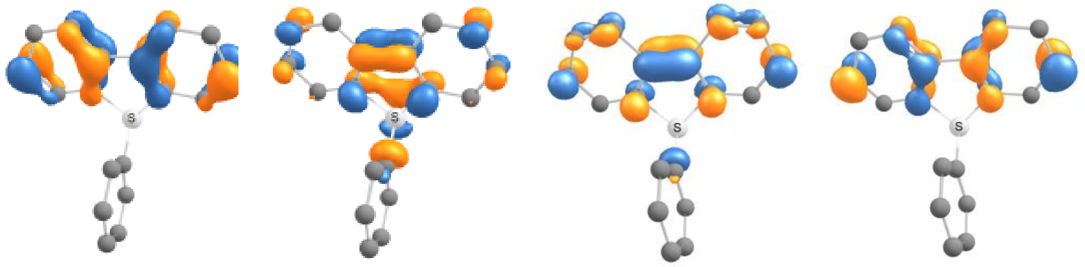

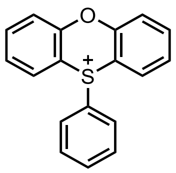

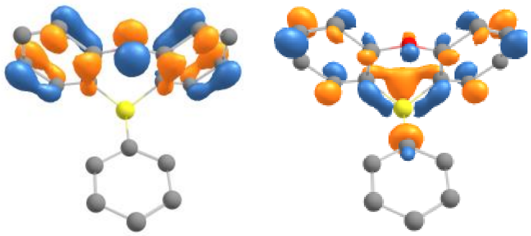

Exocyclic C–S bond cleaved during structure optimization

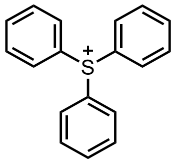

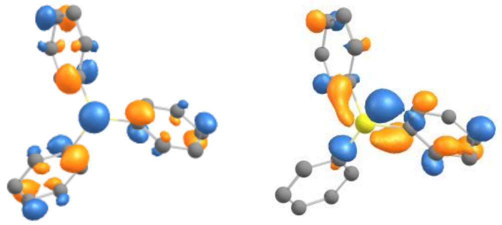

C–S bond cleaved during structure optimization

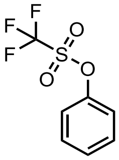	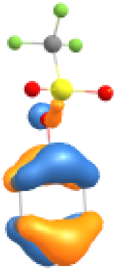	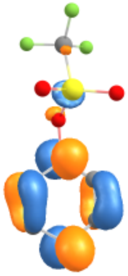	O–S single bond cleaved during structure optimization	
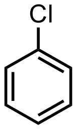	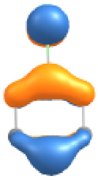	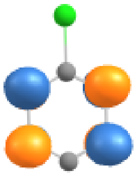	Triplet structure optimization failed	
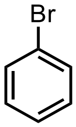	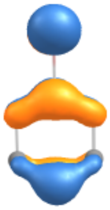	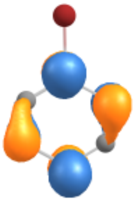	Triplet structure optimization failed	
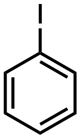	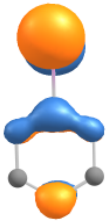	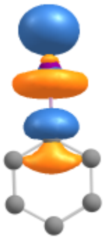	C–I single bond cleaved during structure optimization	
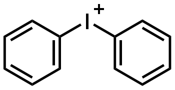	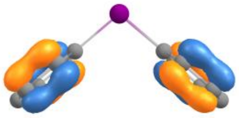	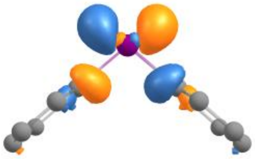	Triplet structure optimization failed	
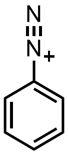 **(61.8)**	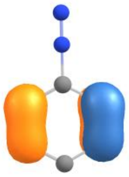	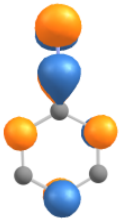	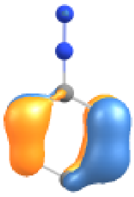	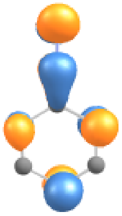

HOMO/LUMO energy of arylpseudohalides

Table S15. HOMO/LUMO energy of arylpseudohalides at ground state

p-Y-C_6H_4X	HOMO/eV	LUMO/eV	HOMO-LUMO gap
C_6H_6	-6.97	-0.31	6.66
C_6H_5Cl	-6.85	-0.68	6.17
C_6H_5Br	-6.79	-0.72	6.07
$C_6H_5TT^+$	-9.72	-5.11	4.61
p-Cl-C_6H_4Cl	-6.80	-1.03	5.77
p-Cl-C_6H_4Br	-6.77	-1.06	5.71
p-Cl-$C_6H_4TT^+$	-9.77	-5.19	4.58
p-Br-C_6H_4Br	-6.75	-1.08	5.67
p-Br-$C_6H_4TT^+$	-9.76	-5.19	4.57
C_6H_5OMe	-6.11	-0.30	5.81
p-MeO-C_6H_4Cl	-6.14	-0.66	5.48
p-MeO-C_6H_4Br	-6.15	-0.70	5.45
p-MeO-$C_6H_4TT^+$	-9.43	-4.97	4.46
C_6H_5CHO	-7.18	-2.01	5.17
p-CHO-C_6H_4Cl	-7.29	-2.21	5.08
p-CHO-C_6H_4Br	-7.22	-2.25	4.97
p-CHO-$C_6H_4TT^+$	-9.86	-5.38	4.48
C_6H_5COMe	-6.97	-1.76	5.21
p-Ac-C_6H_4Cl	-7.13	-1.97	5.16
p-Ac-C_6H_4Br	-7.08	-2.00	5.08
p-Ac-$C_6H_4TT^+$	-9.68	-5.21	4.47
C_6H_5COPh	-6.86	-1.94	4.92
p-PhCO-C_6H_4Cl	-6.95	-2.11	4.84
p-PhCO-C_6H_4Br	-6.93	-2.13	4.80
p-PhCO-$C_6H_4TT^+$	-9.27	-5.13	4.14

**PhOTf**	-7.55	-1.01	6.54
**PhI**	-6.56	-0.97	5.59
**$PhDBT^+$**	-10.04	-5.37	4.67
**$PhPT^+$**	-10.11	-5.04	5.07
**Ph_3S^+**	-10.49	-4.96	5.53
**Ph_2I^+**	-11.03	-5.96	5.07
**PhN_2^+**	-12.56	-8.02	4.54

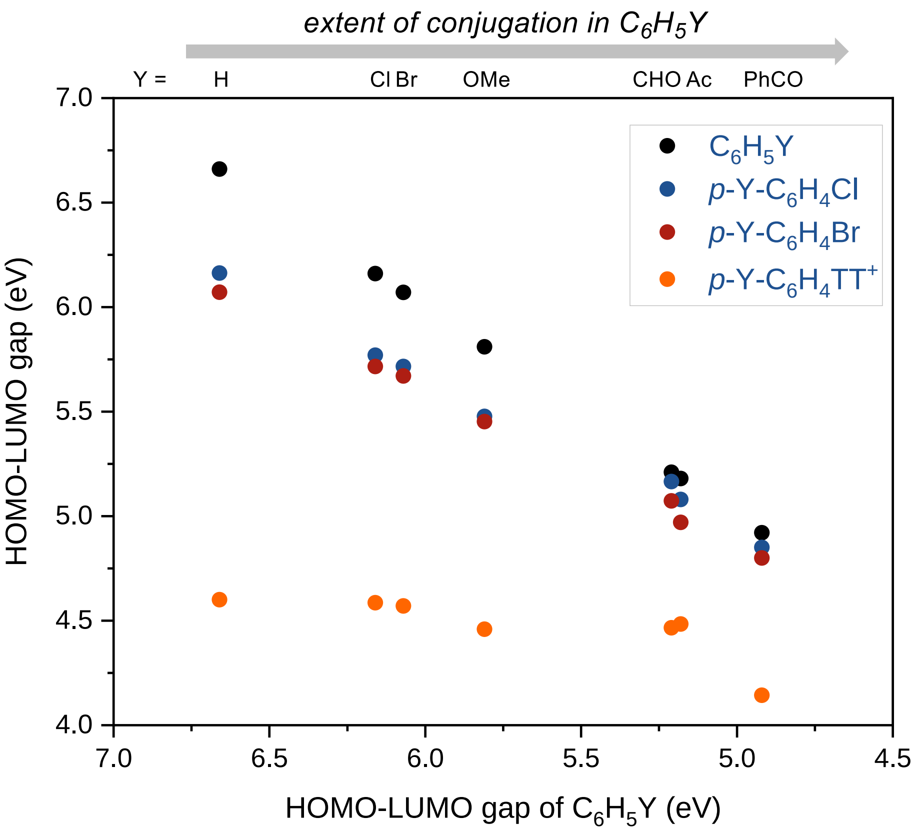

Figure S12. Relationship between HOMO-LUMO gap of aryl(pseudo)halides *p*-Y-C_6H_4X and conjugation extent of their parent C_6H_5Y.

Visualization of frontier molecular orbitals of TXO

As shown in Figure S13, the Dexter energy transfer process involves the simultaneous exchange of electrons between excited photosensitizer and substrate at ground state to accomplish targeted energy transfer. Specifically, the process entails one electron migrating from the *α*-HOMO of the excited photosensitizer to the LUMO of the ground state substrate, while the other electron travels in the reverse direction from the HOMO

of the ground state substrate to the *β*-LUMO of excited photosensitizer.

As depicted in Table S16, the *α*-HOMO and *β*-LUMO of the excited TXO predominantly situate on the carbon of the carbonyl group and the sulfur atom, respectively. The HOMO and LUMO orbitals of the $PhTT^+$ are primarily located on the neutral sulfur and positively charged sulfur atoms, respectively. The spatial alignment of frontier molecular orbitals coincides precisely between excited TXO and $PhTT^+$, given the analogous tricyclic fused ring system of TXO and TT, and the shared location of the middle ring for all frontier orbitals involved in energy transfer.

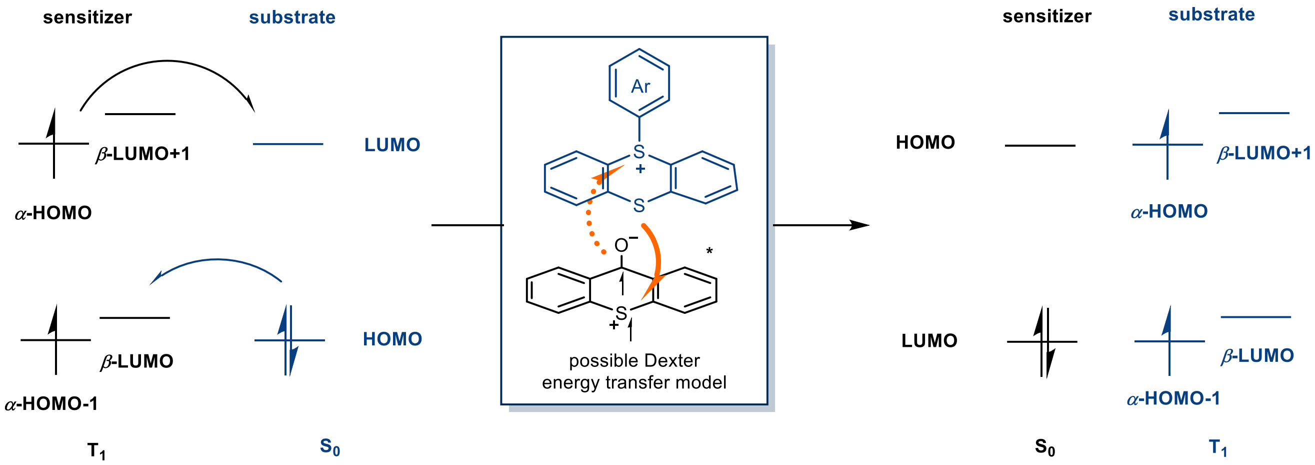

Figure S13. Dexter energy transfer mechanism

Table S16. Frontier molecular orbitals of TXO at ground and excited state

Ground state		Triplet state	
HOMO	LUMO	*α*-HOMO	*β*-LUMO
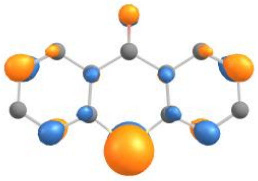	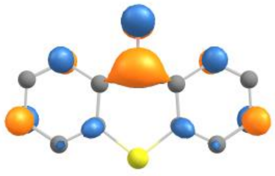	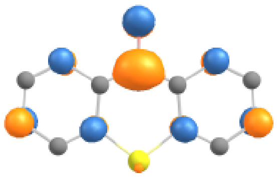	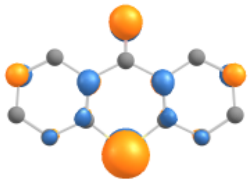

Bond dissociation energy of thianthrenium salts

Table S17. Thermodynamic quantities of the investigated species at the CPCM(acetone)-B3LYP-D3BJ/def2-SVP level of theory.

**Compounds**	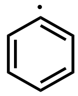	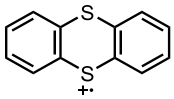	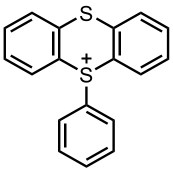

**Enthalpy (Eh)**	-231.17115	-1257.14139	-1488.41480
**Compounds**	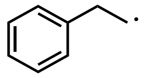	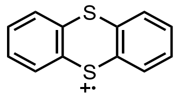	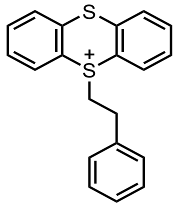
**Enthalpy (Eh)**	-309.65722	-1257.14139	-1566.88394

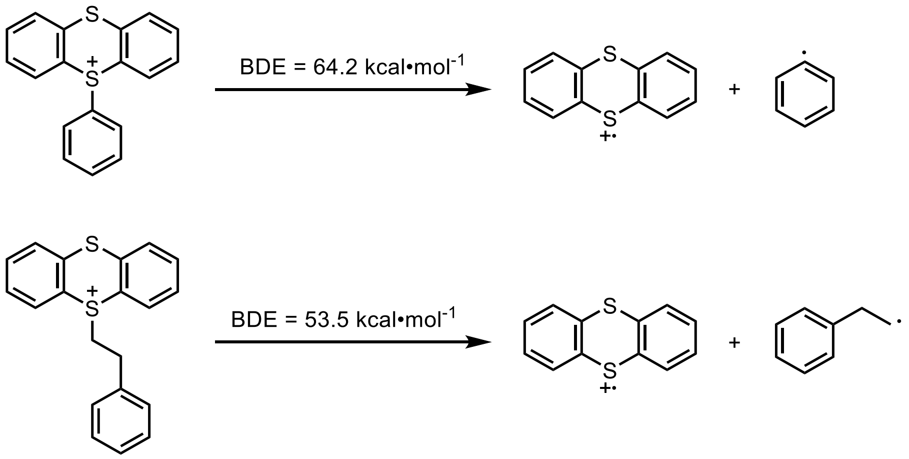

Figure S14. Bond dissociation energy of thianthrenium salts.

Cartesian coordinates of the optimized species

Calculated coordinates of arylthianthrenium salts in singlet and triplet states:

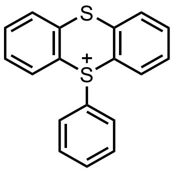

Singlet

C	-0.61901973001864	1.86702414888472	0.67159027587191
C	-1.74121615409666	1.09038548027600	0.37965365642808
C	-1.54518433070999	-0.15897086656888	-0.21409075152442
C	-0.25268872346613	-0.61181763931714	-0.49576094288486
C	0.85527096191198	0.18163658910933	-0.18389754926285
C	0.68167889900754	1.43427311980144	0.41027191032387
H	1.54547011874645	2.05759992099798	0.64665342436887
H	-2.74982988850184	1.44834932529598	0.59240550105264
H	-2.41037239656325	-0.77840019903097	-0.45932252569689
H	1.86434501935613	-0.17138702380617	-0.40654814325192
S	-0.85854350666715	3.44244152113066	1.54871826604638
C	-2.32809864729414	4.09875344926907	0.79232621582754
C	-2.32244765951945	4.45325646311945	-0.56802154850284

S	-0.88434439226467	4.35493989986711	-1.60030921761648
C	0.40976062422729	4.82360222354670	-0.48307100565460
C	0.42674092959400	4.47194423449813	0.87791242752364
C	1.48879068430548	5.56578469495544	-0.98490470491131
C	2.53520980843853	5.94320945879163	-0.14084599146262
C	2.51837723402622	5.61286706282438	1.21971700543613
C	1.45414848831169	4.87591393230809	1.73872231105292
C	-3.52699251759328	4.88629530528743	-1.14076237182539
C	-4.68546868888258	4.96522509395819	-0.36506744901566
C	-4.66444798248480	4.63999776110076	0.99650466336202
C	-3.47637694996611	4.20814011072015	1.58558756995408
H	-3.54573385671892	5.18209901898468	-2.19169750995397
H	-5.61395802987860	5.30752419169160	-0.82726776963762
H	-5.57125295157125	4.72316938510595	1.59852482896000
H	-3.44015057468403	3.94158461352076	2.64421308886804
H	1.49249651458008	5.86448165576782	-2.03529513981112
H	3.36629079185481	6.52313079085474	-0.54876536566455
H	3.33189980669448	5.92892984589224	1.87530933903372
H	1.42525204313194	4.60081470411879	2.79539880390659
H	-0.10806494330512	-1.58916827295607	-0.96113130133930

Triplet

C	-0.58149244425263	2.05242327400765	0.80944277540042
C	-0.94822893434918	1.77058447891708	-0.51312184470849
C	-0.59639836448928	0.53150036696863	-1.05207390492833
C	0.11286980823246	-0.39888644138491	-0.28494193065330
C	0.47489628064448	-0.09861917496941	1.03371353798926
C	0.13544514258532	1.13427399545038	1.59088727710043
H	0.41493870215851	1.37553554087837	2.61900431178718
H	-1.51608246196929	2.48553591784040	-1.10868965072839
H	-0.88292539230153	0.29181477838873	-2.07814259699240
H	1.01855510526060	-0.83050870596940	1.63514228644740
S	-0.84444487494945	3.66118768557004	1.55994609000995
C	-2.42974231845482	4.17484956979536	0.89641014118686
C	-2.58318007153747	4.89206671987683	-0.30645026570344
S	-1.31174884772567	5.42566691378554	-1.40583979256298
C	0.19070767718259	4.98443113138459	-0.69165320556313
C	0.28130299337519	4.80203764854567	0.75544942197644
C	1.38561650043599	5.19667988434686	-1.46007133975166
C	2.58227392495980	5.36592523849626	-0.81930376219176
C	2.63275292204930	5.34420419716004	0.62966534006860
C	1.51087647872487	5.03296045048550	1.39140112433447
C	-3.88557865740658	5.23124661720805	-0.72598230060407
C	-4.99457963295995	4.85295932869642	0.02710858232952
C	-4.82980400907370	4.13948639897280	1.21898446605582
C	-3.54384605568198	3.80447939121080	1.65194526495059
H	-4.01850158793353	5.79138093353993	-1.65509461950739

H	-5.99445527246728	5.12432731512266	-0.31721512061563
H	-5.69567470927417	3.85570401514745	1.81974813425659
H	-3.40187719990011	3.25608775759275	2.58647174545247
H	1.31609427170719	5.27163834452035	-2.54956455583444
H	3.49867675051249	5.53450743027386	-1.38607612521324
H	3.57875206050242	5.55917013870557	1.13218817282947
H	1.59453258299503	4.93478479457108	2.47834903901146
H	0.38180963340040	-1.36580593513601	-0.71488669562827

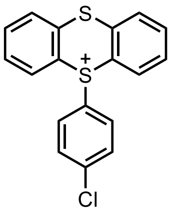

Singlet

C	-0.61984213166609	1.87149933482124	0.67782992263461
C	-1.73879190568175	1.09004767832519	0.38356260334583
C	-1.55231704600446	-0.15439695165193	-0.21637659103207
C	-0.25556673892450	-0.60076956973930	-0.50319444048355
C	0.85885336320513	0.18882810480276	-0.19091479102476
C	0.67858609986937	1.43437928198245	0.40890703716089
H	1.54625209464696	2.05294573456647	0.64403892274479
H	-2.74917768389177	1.44157746768630	0.59903282328703
H	-2.40909273845146	-0.78114051870132	-0.46718271899837
H	1.86167195270671	-0.17268051877347	-0.42218498912031
S	-0.85846345114841	3.44335377010757	1.55442931796939
C	-2.32780572287918	4.09716883396822	0.79499099311866
C	-2.32098333436562	4.44672853205071	-0.56673521850401
S	-0.88207039110592	4.34502859104786	-1.59768981956521
C	0.41144481002440	4.81729432334519	-0.48116473925660
C	0.42788374259416	4.47017516493420	0.88110566748502
C	1.49078433287645	5.55784193010848	-0.98469006794730
C	2.53648660569841	5.93831025372168	-0.14102478025966
C	2.51878505308544	5.61266420015018	1.22069602412237
C	1.45442302412865	4.87723330028416	1.74159300656378
C	-3.52532489041245	4.87703900581407	-1.14190994564378
C	-4.68434967761611	4.95844978531440	-0.36719960674722
C	-4.66422623449476	4.63850367532459	0.99567767100443
C	-3.47651222000365	4.20920784991428	1.58736890216861
H	-3.54354318946016	5.16895849735612	-2.19395649052204
H	-5.61266686315890	5.29856785647118	-0.83135543248395
H	-5.57151004754083	4.72375756974862	1.59668744168914
H	-3.44101023772987	3.94684428981899	2.64710088236085
H	1.49522625321246	5.85318037289893	-2.03604213450104
H	3.36773758608016	6.51704072980743	-0.55029187956373

H	3.33173318944091	5.93130163488188	1.87575734347660
H	1.42474270967852	4.60604945429156	2.79929117813460
Cl	-0.02874631271181	-2.14273966467867	-1.25282609161301

Triplet

C	-0.70450901798049	2.04398903619637	0.81232359020763
C	-1.18836507269430	0.95556017982814	1.55545270662019
C	-1.16582888116652	-0.31770182339274	0.99419587474533
C	-0.67350488040538	-0.49202521528170	-0.30783158665804
C	-0.19742388011504	0.59916814589132	-1.04780513166570
C	-0.21578334422955	1.87608364119533	-0.49111915956723
H	0.16991084605696	2.71807220853002	-1.06509413814059
H	-1.57085204429343	1.09794332588018	2.56881645042947
H	-1.52211533590295	-1.17982563673398	1.55948707159669
H	0.18721747407669	0.44080588980228	-2.05613688791830
S	-0.92751279644366	3.65357094009897	1.56077230877173
C	-2.26811848008689	4.45954599768863	0.67707125394271
C	-2.14006837032225	4.66734348759213	-0.76327582346737
S	-0.76984817206788	5.49942080553240	-1.38894375526773
C	0.52865272048933	5.31758999633194	-0.20940651667947
C	0.49986598761650	4.57783403273367	0.98923683130362
C	1.71625916786217	5.99485228865002	-0.55406848301128
C	2.83881258188546	5.92125901263415	0.26757378731647
C	2.79837337816854	5.18245489830945	1.45466140126957
C	1.62470405532204	4.51356723271671	1.81362997064314
C	-3.29442596106604	4.54745344254732	-1.60892485126949
C	-4.53016044825351	4.37474171407188	-1.04747575870279
C	-4.66495346985017	4.32952546781103	0.39500367931068
C	-3.55260842563639	4.33704051008132	1.23003989480288
H	-3.17793078736168	4.64646751969359	-2.69243253590568
H	-5.41927323771091	4.28708887178074	-1.67295696855960
H	-5.66350677771077	4.26887753168976	0.83431874579554
H	-3.67614854976243	4.21288815003207	2.31047837410323
H	1.75020857193137	6.57630897773803	-1.47904876903860
H	3.74974445655239	6.45024074212872	-0.01966486638831
H	3.67131975468402	5.13597391929409	2.10864137876503
H	1.57905487948925	3.94045272820245	2.74287580654849
Cl	-0.64857594107444	-2.07431801927443	-1.00706389393220

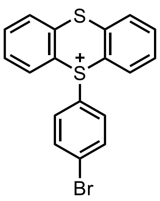

Singlet

C	-0.62047451846390	1.87931358307973	0.69123763643065

C	-1.73910237637356	1.10858977549565	0.36991540352871
C	-1.55028892651458	-0.13104713883040	-0.24107368122150
C	-0.25215284997117	-0.58226846396050	-0.51213496274733
C	0.86167387505533	0.19481417361168	-0.16905552739432
C	0.67935753170345	1.43532844939887	0.44214426201582
H	1.54736540257249	2.04525081498399	0.69824108807441
H	-2.75110965543999	1.46491113869037	0.56969239379052
H	-2.41149176530096	-0.74376496851667	-0.51141538048318
H	1.86869241168757	-0.16577675292714	-0.38367140243502
S	-0.85963540610871	3.45201777593913	1.56707498116855
C	-2.32944032712735	4.10296099027379	0.80618447730823
C	-2.32445597627372	4.44016847746860	-0.55864257639730
S	-0.88653853652401	4.32970653333977	-1.59000751099538
C	0.40877094249936	4.81056802662235	-0.47879157332005
C	0.42547800442747	4.47598615361599	0.88659972751165
C	1.48999323792486	5.54272740946355	-0.99000853136045
C	2.53674833437025	5.92900335156049	-0.15026928316726
C	2.51813801649445	5.61798380299466	1.21467750125658
C	1.45239832182215	4.89016791713263	1.74303872483645
C	-3.53014926278643	4.86320536353874	-1.13629569576229
C	-4.68811597306428	4.95177189112924	-0.36088532979182
C	-4.66557162840679	4.64618751093616	1.00527552806190
C	-3.47695964971872	4.22283425428141	1.59907085497355
H	-3.55058680869945	5.14272775233695	-2.19189045570829
H	-5.61749881022096	5.28544868698003	-0.82772960652326
H	-5.57174892436393	4.73652589537082	1.60708220972713
H	-3.43993977562520	3.97187044307429	2.66154220206125
H	1.49573506641225	5.82625515455262	-2.04471697218266
H	3.36959095346171	6.50066866985244	-0.56569381447728
H	3.33192908213089	5.94104540841226	1.86646658005423
H	1.42255322376401	4.63000758351718	2.80350145394732
Br	-0.00057323334254	-2.25293966341872	-1.35612272077959

Triplet

C	-0.69973644878446	2.04604974887153	0.81450272850507
C	-1.21189048123802	0.95977354672728	1.54144586848815
C	-1.20366803143025	-0.30950487658544	0.96930896811985
C	-0.69753502540186	-0.48220689799845	-0.32768531418465
C	-0.19109576882353	0.60624171053857	-1.05039082645172
C	-0.19538637737645	1.87940205421711	-0.48305530865419
H	0.21609415349705	2.71785361467634	-1.04464679370009
H	-1.60688125532470	1.10007281473026	2.55055441257347
H	-1.58417345834046	-1.16606337569369	1.52768872672556
H	0.20807942201535	0.45659314227587	-2.05473516280866
S	-0.91368397059342	3.65328188743027	1.56973777468404
C	-2.25709700391123	4.46187674367820	0.68534940821932
C	-2.13272059451486	4.64904205946274	-0.75743836648572

S	-0.75988511496579	5.46361144758827	-1.40005187139156
C	0.53557234962332	5.30848150729360	-0.21292212586348
C	0.50911865718419	4.58112307958434	0.99378152410089
C	1.71786648222345	5.99398581015516	-0.55909308158948
C	2.83707690738983	5.94154312948906	0.26887246682041
C	2.79872782751807	5.21588028109174	1.46394076077136
C	1.63031424424733	4.53811169155635	1.82417103341974
C	-3.28931137396954	4.51642300731427	-1.59835246671387
C	-4.52368402825594	4.35677557634326	-1.03000566686010
C	-4.65495458954743	4.33504709125264	0.41305565073171
C	-3.53965164210290	4.35016575219522	1.24457205924153
H	-3.17598454210756	4.59505677264166	-2.68361188967307
H	-5.41475020319646	4.26148994982085	-1.65165465234043
H	-5.65247855405084	4.28550844464493	0.85598611660988
H	-3.65976867518079	4.24224561273932	2.32695449560225
H	1.75017445693085	6.56759995254056	-1.48909028624735
H	3.74316219952588	6.47832399979469	-0.01960422771929
H	3.66840541695249	5.18639025074453	2.12297429595140
H	1.58572540714644	3.97492889289677	2.75970748195602
Br	-0.69339038513772	-2.20285442201796	-1.10692573183698

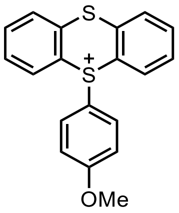

Singlet

C	-0.61035846651115	1.87956685776285	0.64348438682947
C	-1.72812155516078	1.08706936080408	0.34986072451736
C	-1.53797101701238	-0.14492165289122	-0.25662579456789
C	-0.23841047690712	-0.60217876074101	-0.56447348542133
C	0.87170070898431	0.20541987909175	-0.25162514906307
C	0.68326569022906	1.44637750726852	0.35767427277185
H	1.55078635236044	2.06695545941671	0.58886562430522
H	-2.73945403566661	1.42961572413418	0.57557968693323
H	-2.38471247415168	-0.78461471818166	-0.51027724213842
H	1.88538725347424	-0.12022490636261	-0.48148484883986
S	-0.85232194710310	3.43221132833561	1.53987638319187
C	-2.32257513495830	4.09775848580027	0.78889025965256
C	-2.31930258315081	4.46431521694071	-0.56795661195976
S	-0.88046664259256	4.38631392889090	-1.60046414817005
C	0.41208665013458	4.84287106338125	-0.47696592604632
C	0.42916962183930	4.47574902376221	0.87975813735411
C	1.48945902185452	5.59362209068657	-0.96995470107060
C	2.53526176023138	5.96344320565080	-0.12190511219852

C	2.51906483358201	5.61702363322439	1.23461690556574
C	1.45594711833183	4.87201317958510	1.74455383415451
C	-3.52561592394101	4.89718950373394	-1.13715787239263
C	-4.68456287750684	4.96395036942214	-0.36117386183760
C	-4.66164406582967	4.62743877286613	0.99757109971231
C	-3.47142581500778	4.19569701241401	1.58248187341479
O	-0.16242650059014	-1.80173440932335	-1.14896348649161
C	1.10407174581623	-2.34971210807069	-1.48439528089725
H	1.63446532372606	-1.71285638942954	-2.21265556716570
H	0.90449857619963	-3.32863186578700	-1.93625456634533
H	1.73081239219488	-2.48393402409033	-0.58660734930238
H	-3.54525668241770	5.20243760097286	-2.18536073548429
H	-5.61468595242166	5.30547905129165	-0.82060415757950
H	-5.56864557882497	4.70125185140701	1.60051712430910
H	-3.43389251230023	3.92004641762889	2.63871608696045
H	1.49168596468133	5.90516141422211	-2.01660290881280
H	3.36483874737806	6.55018228907011	-0.52307591551860
H	3.33175676126454	5.92708563864155	1.89410684450211
H	1.42711171977212	4.58477296847103	2.79800147712885

Triplet

C	-0.52027930105378	2.05151214698435	0.57812506357797
C	-0.91027855541300	1.87137103527863	-0.76123870629339
C	-0.57969946012059	0.69195715662044	-1.40777680551045
C	0.15001581027463	-0.31654365918215	-0.73993858199646
C	0.54158697886488	-0.12059717909297	0.59996836302287
C	0.21098639252587	1.06465015077752	1.25220332332880
H	0.51760973144178	1.21297748803043	2.29050422615075
H	-1.48699536744698	2.63163977306032	-1.28824148324491
H	-0.87822145004328	0.51278081626976	-2.44181598414471
H	1.09563946810017	-0.88708177211604	1.14086778982232
S	-0.76980701741129	3.58215716723900	1.46113755655738
C	-2.39258574742110	4.11580158534410	0.90872999742112
C	-2.60815373301577	4.92595471482697	-0.22291722486741
S	-1.39130441516533	5.58620182102481	-1.31920915707926
C	0.14453422495186	5.09073490363859	-0.71664136723110
C	0.29683219511956	4.80149370222457	0.71173089494028
C	1.30681077135094	5.40392271749017	-1.50447466828486
C	2.51553084479213	5.57236794636734	-0.88995671195587
C	2.61710332363655	5.45148651816066	0.55479422709942
C	1.53544311882400	5.03889107875437	1.32588025797435
C	-3.93302920980846	5.26684683163143	-0.56146545472299
C	-5.00312696331742	4.80053025121903	0.19874471663134
C	-4.77624649327766	3.99430600699378	1.31914064269766
C	-3.46724791271587	3.65598875351690	1.67221201557413
O	0.41573529445517	-1.41477408062293	-1.45279631895907
C	1.13545549326701	-2.48915511829574	-0.86427734934207

H	2.14518467400596	-2.17116491646000	-0.55475846945186
H	1.22052901190756	-3.26133966455275	-1.63866774647930
H	0.59609479693290	-2.90210717694983	0.00475847994069
H	-4.11515414193279	5.89913621200906	-1.43439200970923
H	-6.02135900138008	5.07534735353640	-0.08365380738778
H	-5.61122123117723	3.64026082932697	1.92622506766700
H	-3.27588918975636	3.03406547564292	2.55046277209966
H	1.19870067585895	5.55214433320015	-2.58315461405016
H	3.40683283220101	5.81295277922803	-1.47072586587538
H	3.57022416560326	5.67157183349678	1.04110951097481
H	1.65926938634286	4.86592218537878	2.39947742110568

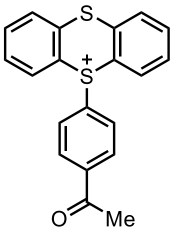

Singlet

C	-0.50167781906619	1.81360632502719	0.82925887516386
C	-1.59561322438970	1.00219800479078	0.51856314451923
C	-1.34975860295464	-0.26578705103569	-0.00095458547789
C	-0.03643504735221	-0.72138059367097	-0.19703876980004
C	1.03962334825755	0.11292958489404	0.13696185094649
C	0.81553130239756	1.38986365010465	0.65730780480908
H	1.65755051471486	2.03792288184620	0.90580422762031
H	-2.61966948844279	1.35115012252940	0.66115501815186
H	-2.16986708504013	-0.93380288459416	-0.27150013766008
H	2.06784695951769	-0.22127980515308	-0.00876167228636
S	-0.80310795717375	3.42869376005132	1.61024714951552
C	-2.27996334842230	3.99256760376942	0.79698833935289
C	-2.26270881161481	4.26699780237983	-0.58166028270577
S	-0.80553280720367	4.14848367518074	-1.58558565012570
C	0.45558896117945	4.72587900215821	-0.48106827438785
C	0.46227053765543	4.45575022783425	0.89852810178787
C	1.51780235072883	5.47282535475569	-1.01071745949669
C	2.53768267913454	5.93364998759777	-0.17574845873526
C	2.50974945143899	5.68349115521614	1.20180772484874
C	1.46167588622504	4.94367909659621	1.74882291727071
C	-3.47031497607887	4.62756904332004	-1.19630062153246
C	-4.64319038582335	4.71433569353455	-0.44338910488381
C	-4.63467584142117	4.46887561330807	0.93507906053119
C	-3.44383247541499	4.11015589916696	1.56601277874404
H	-3.48110696160635	4.86051396037141	-2.26314608536126
H	-5.57423434369906	4.99915242362727	-0.93828470145288

H	-5.55367224668447	4.55667528857119	1.51761657186409
H	-3.41735338593752	3.90539163164578	2.63871617085091
H	1.52829227429756	5.71004681693957	-2.07679260123707
H	3.35589278340434	6.51620682681749	-0.60546651012181
H	3.30198311699384	6.06486315231695	1.84870585740369
H	1.42411751168139	4.73185085102799	2.81986488921499
C	0.14061503636307	-2.11335400722746	-0.77112406300813
O	-0.84197134563997	-2.76355483366004	-1.05263272540011
C	1.54188494983988	-2.63996235975891	-0.97060058530745
H	2.10719983244036	-1.99555175771953	-1.66371374382954
H	1.48493693826048	-3.65518075327125	-1.38174098878556
H	2.09286171943509	-2.66050138928807	-0.01617346099975

Triplet

C	-0.50971148661954	1.82784036650511	0.83202153743635
C	-1.60281516384173	0.99724570582550	0.52632300014622
C	-1.37055642357988	-0.26480011759704	0.01513912581403
C	-0.03663952464873	-0.74017013303999	-0.19644997562434
C	1.04825497685329	0.12792673170720	0.13642371392277
C	0.81391102635948	1.39173435868826	0.64789133914713
H	1.65750414410145	2.04115339341019	0.88853822173386
H	-2.62826766702879	1.34105732667223	0.67316257005900
H	-2.21686235096260	-0.90744725534568	-0.23508878692323
H	2.07849428873442	-0.19727156183897	-0.01402696611277
S	-0.80744925097930	3.42237739468920	1.61616564246174
C	-2.28184757245356	3.99711364765932	0.79780105768193
C	-2.26286050191775	4.26442184505597	-0.58178357589904
S	-0.80434265198635	4.14319811731034	-1.58282374428720
C	0.45540543450513	4.72051491195863	-0.47713349406287
C	0.45602476526968	4.45754393974312	0.90337318344135
C	1.51957126218462	5.46460815514923	-1.00710929767268
C	2.53735852550824	5.92858976085066	-0.17139625622629
C	2.50422060003807	5.68514236213911	1.20692907428074
C	1.45317381336082	4.94866365941113	1.75339573350867
C	-3.46847242981677	4.62617460903201	-1.19999947187189
C	-4.64244136785094	4.71951189572803	-0.44971134527106
C	-4.63613886614102	4.48062547100003	0.92960730476239
C	-3.44650370858317	4.12172432104148	1.56333909665698
H	-3.47628008530130	4.85557039192116	-2.26766370771025
H	-5.57194211374678	5.00472240705937	-0.94730916945318
H	-5.55555614635579	4.57388615117265	1.51068756707520
H	-3.42132833187228	3.92187988863470	2.63703981998345
H	1.53296240996048	5.69720011487875	-2.07418801031654
H	3.35767310343725	6.50805546002656	-0.60130029531736
H	3.29467410051340	6.06863983813420	1.85478754087831
H	1.41168964653241	4.74086259657205	2.82510562360642
C	0.19232755134133	-2.03419033332022	-0.72277938436050

O	-0.78676318536905	-2.83188714278213	-1.02887348362866
C	1.55358947311611	-2.64875891636848	-0.99089493988242
H	2.11131801511513	-2.01660767340015	-1.70058305256146
H	1.44947523779644	-3.65309746145676	-1.42226002105982
H	2.11757045432761	-2.72878422682679	-0.04731617435494

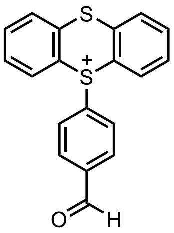

Singlet

C	-0.63522924616290	1.91201456167810	0.72238577396592
C	-1.75454957178454	1.14403721718771	0.40086973153065
C	-1.54968317911342	-0.10329887617108	-0.19306826096894
C	-0.25168402882808	-0.56232595026450	-0.44716522227095
C	0.85569163058367	0.22599066252543	-0.09859076568309
C	0.67257823161459	1.47152863855111	0.49566271847508
H	1.53234669745448	2.09040386348766	0.75745982895409
H	-2.76587894154841	1.50867974639932	0.58759297945630
H	-2.40977341891410	-0.72103377008228	-0.46564544903741
H	1.85627903459024	-0.15844510095821	-0.30875894613600
S	-0.88129340292531	3.49784672778326	1.58177327101304
C	-2.34307429676579	4.14493050037888	0.80388986126059
C	-2.32431399129202	4.47229285245522	-0.56354702335924
S	-0.87782956873986	4.34331549630304	-1.58086075882323
C	0.40910809539876	4.83342919972092	-0.46359466854188
C	0.41298597130951	4.51164852514849	0.90517815542223
C	1.49719796633488	5.55661655965227	-0.97250816361032
C	2.53788005671599	5.94723440393654	-0.12702016394323
C	2.50626719619434	5.64951818849867	1.24044659034622
C	1.43368835160840	4.93026954218868	1.76687624485582
C	-3.52234846290653	4.89840648119865	-1.15449182755577
C	-4.68606381046563	5.00036369796987	-0.38916411061047
C	-4.67693438642186	4.70475129629264	0.97915022729504
C	-3.49627909698652	4.27802425348249	1.58640905038972
H	-3.53223964533285	5.17033095842663	-2.21219562717428
H	-5.60923268818814	5.33713170321038	-0.86600973491722
H	-5.58741930139545	4.80590421511628	1.57268314870045
H	-3.47000425530064	4.03550359711878	2.65113895244512
H	1.51373855561342	5.82927808133972	-2.02993234050620
H	3.37664958248275	6.51148042675010	-0.54062948425921
H	3.31580272722390	5.97556984332964	1.89602014234984
H	1.39456039528247	4.67983177389981	2.82934547127169

C	-0.02681143205972	-1.89166508137137	-1.09522282562242
O	1.06961034956935	-2.32225921790313	-1.34884966041947
H	-0.95681211684499	-2.46616501727975	-1.33293711429250

Triplet

C	-0.63551578576474	1.92516384229089	0.72095700849009
C	-1.75603306053291	1.13490510768546	0.40859729747320
C	-1.56865378272531	-0.10237113281383	-0.17902300715621
C	-0.25474188303376	-0.58551937162621	-0.46040976281419
C	0.86248882966417	0.23821967031713	-0.11698916860162
C	0.67144228337922	1.47547145813054	0.47097084411043
H	1.53564994537961	2.09447430726080	0.71856348997223
H	-2.76821602096213	1.49171244494384	0.60716967822404
H	-2.43711767357063	-0.71339622072279	-0.43422841833198
H	1.87604303555137	-0.11033079720996	-0.32393387498785
S	-0.87930382227081	3.49039085939898	1.58438305342852
C	-2.33936741782660	4.14698197806661	0.80335131064856
C	-2.32058029539796	4.46976166586629	-0.56434286634091
S	-0.87228130171742	4.34521237320268	-1.57942820611058
C	0.41259564406288	4.83341500377482	-0.45923911575987
C	0.41273156365581	4.51327299847283	0.90931507229358
C	1.50087565836408	5.55802468205140	-0.96619258516682
C	2.53980209373667	5.94993842599240	-0.11937807072970
C	2.50537577839043	5.65269731395204	1.24821393747155
C	1.43150444029217	4.93320435663130	1.77187344674011
C	-3.51830734744272	4.89369790173133	-1.15775363302155
C	-4.68314000951284	4.99623627264545	-0.39447710280985
C	-4.67467892690386	4.70443006788483	0.97474771205517
C	-3.49350789734262	4.28097538050129	1.58344730041075
H	-3.52678874657661	5.16354806179874	-2.21603148586005
H	-5.60627517680380	5.33035844590926	-0.87324783446990
H	-5.58579074670979	4.80588438345602	1.56730002119643
H	-3.46694995162210	4.04052676947314	2.64868597894935
H	1.51839683322638	5.83095036592045	-2.02356594418351
H	3.37888567345083	6.51476397586563	-0.53157901390056
H	3.31324010000808	5.97950282289956	1.90550279261136
H	1.38928498771887	4.68266148344679	2.83423844594831
C	-0.07463048164130	-1.84904713922505	-1.06686699433141
O	1.06836641042661	-2.37540177400360	-1.36828480893607
H	-0.92787294894923	-2.49917598396910	-1.33565549651103

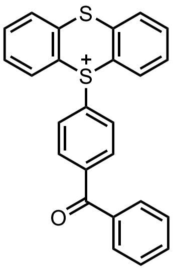

Singlet

C	-0.64353163870870	1.96464636636667	0.90395708538304
C	-1.77034351891386	1.15000991815977	0.80019791733115
C	-1.60840846778761	-0.15113172713384	0.31851221422726
C	-0.33791065370593	-0.62871000209072	-0.03467758329279
C	0.78026809847130	0.20514814884918	0.12276304873244
C	0.63830880791293	1.51170523713652	0.58384983389433
H	1.51063101647348	2.16003004531668	0.68002315394978
H	-2.76218679247527	1.51486570673210	1.07270882870897
H	-2.48089648243432	-0.80053426970871	0.23487043397352
H	1.76684787735243	-0.19380098779883	-0.12008203868979
S	-0.84058369697224	3.62905819286821	1.60900729977796
C	-2.35464296971341	4.19286826446051	0.86458179137507
C	-2.42807519056167	4.37857262298472	-0.52698529923444
S	-1.04969819695253	4.15029950561426	-1.61888282396279
C	0.30372316569220	4.75123386711982	-0.64278951212409
C	0.39842669862637	4.56943481984617	0.74808032440896
C	1.35140501506077	5.41949865063321	-1.29212833963564
C	2.44385457968617	5.89131156575760	-0.56109475979525
C	2.50438687072917	5.73125625073443	0.82842389307146
C	1.47229330727755	5.07014578115053	1.49338538703375
C	-3.66526723277284	4.74101625972105	-1.07845910464607
C	-4.77809713198894	4.91731674042604	-0.25350013168687
C	-4.67772318440241	4.76187642673408	1.13420040637017
C	-3.45597222576536	4.40266318763959	1.70269834149086
H	-3.74540872752132	4.90508233455060	-2.15526212809317
H	-5.73301013921359	5.20217628585320	-0.70072007751778
H	-5.54865784989651	4.92044680314944	1.77278273733532
H	-3.35804202319729	4.27001972255451	2.78253532163018
H	1.29645283617244	5.58513696468872	-2.37023487510546
H	3.25050206068255	6.41020350554015	-1.08369016253957
H	3.35412623546631	6.11919292308179	1.39318473647743
H	1.50472385340959	4.92642378693347	2.57570807865717
C	-0.08372262558916	-2.05472291071754	-0.47625896562196
O	0.97262688619925	-2.57030966936149	-0.16104356824644
C	-1.11440421938041	-2.78807056302175	-1.26342750159845
C	-1.08218506039807	-4.19425286924993	-1.23219675918954
H	-0.33019256295368	-4.68487614774226	-0.61145638717324

C	-1.99427037301397	-4.93490741959703	-1.97988272110601
C	-2.93315225526894	-4.28057912410771	-2.78694763277679
H	-1.97278227958990	-6.02636103686155	-1.94099092675402
C	-2.95542240677702	-2.88383483200778	-2.84600699088398
H	-3.64442580936035	-4.86240784852411	-3.37819751336540
C	-2.05459227266385	-2.13820027102448	-2.08366688294429
H	-3.67342078690454	-2.37494224027870	-3.49332205086877
H	-2.06251053432881	-1.04902796537663	-2.15716609697623

Triplet

C	-0.65413253352298	1.99037005782703	0.80020279335093
C	-1.79026693311398	1.20315440311934	0.54116814534303
C	-1.63056967682312	-0.06222714777843	0.00501103202669
C	-0.33244549770134	-0.57686539358960	-0.28697905357192
C	0.79933617123058	0.24255318292191	0.01431267987861
C	0.64214672068589	1.50628425403638	0.55246203105749
H	1.52085567608531	2.12070829471057	0.75748854547655
H	-2.79320128198680	1.57777316876013	0.75312691129223
H	-2.51250030588455	-0.67711577336646	-0.17802241169759
H	1.80116235502787	-0.13217254543309	-0.20293919824240
S	-0.84696256235656	3.58729015569719	1.60980131132777
C	-2.34649579615395	4.22183333312879	0.88646515500570
C	-2.40449764950770	4.49135635196292	-0.49157873100667
S	-1.01788636305812	4.31696570288463	-1.58236334534411
C	0.33214200281213	4.83990196682656	-0.55840624852894
C	0.40645625775643	4.57881316694143	0.82024243046691
C	1.39394500232542	5.53351497276379	-1.15649882027030
C	2.48176887712361	5.95164775509440	-0.38722103282058
C	2.52216060362875	5.71362844098785	0.99162722434932
C	1.47468463666034	5.02673691291379	1.60499017081781
C	-3.63225865032803	4.90064972923227	-1.03194477825394
C	-4.75188235857460	5.03843515836552	-0.20912068372624
C	-4.66723238536621	4.79755888905744	1.16731832165662
C	-3.45464793690764	4.39122596169485	1.72379475428222
H	-3.69903609706866	5.13122354094391	-2.09742171428787
H	-5.69923547463956	5.36005803090375	-0.64747319579715
H	-5.54310737779289	4.92556052862039	1.80606628393112
H	-3.36862237835019	4.19049211914867	2.79409344308967
H	1.35308725297313	5.76139624469682	-2.22385213192230
H	3.30021250265036	6.49033597234105	-0.86990381433088
H	3.36797444594609	6.06078673425039	1.58797507867204
H	1.49066920423391	4.82218329918435	2.67787444172472
C	-0.13523574012078	-1.88176327880682	-0.81637209026408
O	1.04873262684985	-2.41575239510552	-0.90965130485558
C	-1.14041305437867	-2.79202274274708	-1.39472284621499
C	-1.06241108485996	-4.17715495894388	-1.10363481057218
H	-0.28530786064653	-4.53619728968314	-0.42767614790159

C	-1.98957387729918	-5.05810075178111	-1.65443929755455
C	-2.98557394362163	-4.58088399799692	-2.51263422414885
H	-1.93695831675713	-6.12192715872574	-1.41307178944791
C	-3.04838023288252	-3.21227265287063	-2.83058980162857
H	-3.71137968677952	-5.27243545456702	-2.94656186331214
C	-2.12060832088709	-2.32430993131763	-2.30034191198345
H	-3.82218109681621	-2.84706620324211	-3.50988409485430
H	-2.14328986180352	-1.26920065306100	-2.57831541121036

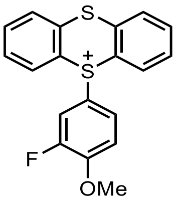

Singlet

C	-0.59296873161189	1.88940248461515	0.64279824533270
C	-1.71674300771400	1.10852152770388	0.34118202761040
C	-1.51946363401375	-0.11978795299307	-0.26386411658256
C	-0.22174439446700	-0.60390164266271	-0.56789842749795
C	0.87845096532685	0.20411925570868	-0.24040640574757
C	0.69830158429102	1.44959812004099	0.37006967826914
H	1.56874707368913	2.06273847759037	0.60769105871155
H	-2.73787884356942	1.43474527260714	0.54287007020822
F	-2.56440794620597	-0.87404025503575	-0.57835397510160
H	1.89020674051265	-0.13249685687492	-0.46366732001252
S	-0.83917685300514	3.44042475734455	1.54254090081848
C	-2.31263111588502	4.09882897404266	0.79282224820298
C	-2.31127708201365	4.46207723146560	-0.56491267735845
S	-0.87206725649591	4.38704329416544	-1.59763537672125
C	0.41874871761414	4.85291014431758	-0.47527845916908
C	0.43878054818525	4.48655674979135	0.88176376204808
C	1.49204368299777	5.60885733496750	-0.96861556677092
C	2.53494767652086	5.98625768641214	-0.12016769489350
C	2.52019558374739	5.64203346084245	1.23673750350219
C	1.46187790482003	4.89096086053630	1.74718900526826
C	-3.52061045044605	4.88544709429192	-1.13462453321388
C	-4.67948996997236	4.94798632575749	-0.35812887942357
C	-4.65417798262495	4.61583900176133	1.00165807670036
C	-3.46151877159749	4.19219769920630	1.58710773014965
O	-0.16705363989997	-1.80096168993428	-1.15005415355844
C	1.09488571594556	-2.36272324141448	-1.48398047605543
H	1.63150468219782	-1.73012076855700	-2.21116017237765
H	0.88427757584839	-3.33871328511066	-1.93695647124068
H	1.71747845152590	-2.50347151997581	-0.58434791499159
H	-3.54358074430806	5.18503365286905	-2.18464010384332

H	-5.61199840671713	5.28148933337899	-0.81879356318144
H	-5.56134607545338	4.68509777723487	1.60474899477696
H	-3.42211754353067	3.92015371056255	2.64430665332642
H	1.49377929390226	5.91843599806261	-2.01598504331269
H	3.36096195022082	6.57751320758291	-0.52160668538901
H	3.33045698607246	5.95821287303193	1.89626892416311
H	1.43414731611351	4.60592490666692	2.80129313735458

Triplet

C	-0.55189824669836	2.07093348322817	0.91734433004330
C	-1.16587380464300	1.00426878885608	1.60200569321544
C	-1.07652032693899	-0.26642981540475	1.06810487471130
C	-0.39135133067007	-0.51669791730726	-0.15042078884728
C	0.20356269562066	0.56936810627706	-0.81253121026859
C	0.11555102944379	1.86292305712381	-0.29146701915373
H	0.59367476065794	2.68562421941219	-0.82230149349737
H	-1.69967142585331	1.14350172746359	2.54433099641033
F	-1.63433892144337	-1.29183481054744	1.69879759073556
H	0.74327646107657	0.41081543305628	-1.74557523888853
S	-0.86359271838849	3.69445907865378	1.59697403912200
C	-2.19699732074218	4.39043211694985	0.61780008206928
C	-2.01049318441890	4.49003952417288	-0.83073502430601
S	-0.66300081494399	5.36488533420678	-1.45068509860864
C	0.58551655987760	5.32884329718861	-0.20152886615338
C	0.54043787255621	4.65902173433573	1.03719753275210
C	1.74772517472228	6.05550423007605	-0.52839625021194
C	2.83040867786306	6.09711697001478	0.34776890629602
C	2.77429280478424	5.42613919413890	1.57351547506856
C	1.62434450537514	4.70943555595988	1.91591934674472
C	-3.11916957403674	4.24236633128338	-1.70981657034281
C	-4.36950753201187	4.06198324962577	-1.18614455050373
C	-4.57047881314597	4.13691564001983	0.25046112717590
C	-3.49923303488873	4.26216824210739	1.12749141281911
O	-0.37168438418411	-1.78432860676733	-0.55782258038776
C	0.30012934494643	-2.12719038029078	-1.76202020570631
H	-0.14821557378538	-1.61151521400158	-2.62807417177431
H	0.17930659659432	-3.21044490876940	-1.88104108797111
H	1.37421066418201	-1.88320704896557	-1.70094695318476
H	-2.95858408844449	4.25126572031546	-2.79195845871775
H	-5.22491322874897	3.87770366793849	-1.83726691257314
H	-5.58552589261933	4.07275032644573	0.64936562454547
H	-3.66583728328457	4.22514774943365	2.20847854091405
H	1.79421976945195	6.58652793695843	-1.48278876098749
H	3.72149877786634	6.66489010715783	0.07196850689492
H	3.61429108044842	5.47025047595783	2.26918320875366
H	1.56398072442390	4.18855740369573	2.87478395381291

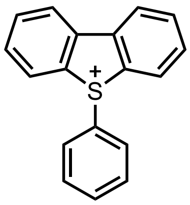

Singlet

C	-0.78931911237228	1.68146931378166	0.97169554586254
C	-0.78218831149676	0.60662029309260	1.85354031535624
C	-0.51588149843394	-0.66232140191405	1.34986540866057
C	-0.26732345004172	-0.83163963583051	-0.00575693120379
C	-0.28175588555691	0.26051888355586	-0.87392864248913
C	-0.54276889902250	1.53155710682680	-0.39026060989648
H	-0.55632153556201	2.38493466832736	-1.05331047146465
H	-0.97926378368055	0.75381446426024	2.90645071992127
H	-0.50485157935268	-1.51159699747113	2.01797958631853
H	-0.08845149802899	0.11797913852467	-1.92773112350356
S	-1.12023274445338	3.28079418862686	1.68346238274816
C	-2.28372778955777	4.07191873816854	0.59108723929573
C	-1.68379607495835	5.12418521450698	-0.11284871863467
C	-0.26057792642157	5.27580891807242	0.19098439283553
C	0.20053520755547	4.33919805587448	1.12440615526523
C	0.65231640256320	6.19598505607750	-0.31494875337684
C	1.97499180670678	6.15040392584622	0.11162887693043
C	2.40333252977315	5.20427086323631	1.04158736475673
C	1.50682845274939	4.28106893827648	1.57079260394394
C	-2.47938621833829	5.86600859705947	-0.98039754418483
C	-3.82319496976840	5.53823797625302	-1.12302495221491
C	-4.39048693613270	4.48334420868153	-0.40956348327210
C	-3.61849662147421	3.73400060075434	0.47266359136669
H	-2.05820985957060	6.69014042640026	-1.53876563885445
H	-4.44029966579785	6.11419046410031	-1.79818897881142
H	-5.43745499082475	4.24756392315075	-0.53456637791840
H	0.33793346568451	6.94041590908263	-1.03286437926097
H	2.68390323947518	6.86448649790451	-0.28331954455544
H	3.43546760763735	5.18853787634933	1.36079533638480
H	1.82631978490742	3.54757117350974	2.29749301403876
H	-0.06143610297032	-1.81944242761438	-0.39381449751244
H	-4.04969304323585	2.91918504252888	1.03683811346889

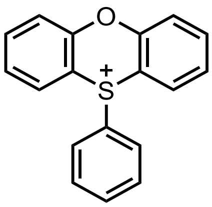

Singlet

C	-0.62122514496083	1.86996353328285	0.76122109086516
C	-1.73749404937722	1.11055897057466	0.43637300448604
C	-1.54418989510040	-0.12639881430150	-0.16632001568149
C	-0.25813399093538	-0.58676246315900	-0.42513106316581
C	0.84521563874619	0.18658736261892	-0.08240888046404
C	0.67238239401463	1.42627182520610	0.52075142545533
H	1.53011797742085	2.03039063543758	0.77784368376178
H	-2.73711849292589	1.47168117836131	0.62877288333436
H	-2.40251041321808	-0.72568270312082	-0.43527323944377
H	1.84511950678610	-0.16942243168383	-0.28637825669208
S	-0.85633457147573	3.43602087266017	1.63596331522108
C	-2.28115830252036	4.11367750418632	0.85757328886037
C	-2.15783827024170	4.63010761024545	-0.43270274961994
O	-0.95672484331443	4.74557824311876	-1.07664832424248
C	0.19319759568880	4.95099909402917	-0.36496917859113
C	0.37918085805165	4.47226966297688	0.93234205128028
C	1.20944498615161	5.67328927104006	-0.97453232697328
C	2.38197142242671	5.92312400045536	-0.27572446439182
C	2.55109449690676	5.47483559057061	1.03390844894459
C	1.54530972846326	4.74572320263136	1.64534201701590
C	-3.29488937167651	5.05402289318872	-1.10600501346942
C	-4.52893251532559	4.97621471639464	-0.47628238913925
C	-4.64491868312239	4.49477273320135	0.82740786230637
C	-3.51626063829315	4.06093259011957	1.50190994449523
H	-3.19270098215840	5.45211083767680	-2.10529739691934
H	-5.41065158886958	5.31239690007929	-1.00354664686623
H	-5.60970741352340	4.45571000530845	1.31135749838583
H	-3.58427533599050	3.67227148285583	2.50875481381014
H	1.05901779623415	6.04117755073472	-1.97932848479749
H	3.16830474882065	6.49038768910892	-0.75353895763516
H	3.46251902202061	5.69110150144123	1.57156291377022
H	1.65932329294390	4.37867758760612	2.65614642304924
H	-0.11559496164633	-1.54896863284609	-0.89640327694919

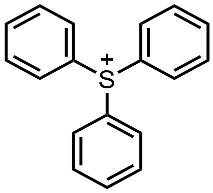

Singlet

C	-0.64920767915086	1.79956054835707	0.58650559441182
C	-1.30706915779013	0.67758935853365	1.08402761681557
C	-1.07711749025361	-0.55138392491211	0.48148539344588
C	-0.19243487828694	-0.64864077514432	-0.58781713333198

C	0.46784883752843	0.48052039916376	-1.06154909403160
C	0.24305800069667	1.71979387459668	-0.47653954543984
H	0.75895971880022	2.59767584509316	-0.83559378183877
H	-1.98424724238057	0.76111151987468	1.92325659235266
H	-1.58040360984281	-1.43198155173875	0.85449678254553
H	1.16155987851766	0.39910833201656	-1.88638646741705
S	-1.02144432629326	3.34215984158898	1.39820292587848
C	-2.41665781600470	4.01600695207594	0.51524017606098
C	-2.76933367668427	3.58048241080980	-0.75644389611138
C	0.33863993419079	5.24187580115648	-0.11481499711052
C	0.34385518376574	4.41940203269426	1.00584780025985
C	1.45018522881294	6.03857894867694	-0.35697373698603
C	2.53854781143319	6.00571471485114	0.50894427451747
C	2.52334236473575	5.18175529153026	1.62984579586967
C	1.41767797430595	4.38462901992087	1.89152638743547
C	-3.86470891838432	4.16757264242154	-1.37734193182337
C	-4.58286737770301	5.16905135495549	-0.73259087975322
C	-4.21869606311773	5.58816280256394	0.54325223270875
C	-3.13341964345948	5.00604568403139	1.18254006675647
H	-4.15594566039210	3.83860982275530	-2.36483700411113
H	-5.43491458102480	5.61896228676240	-1.22264796829595
H	-4.78437958102486	6.35949516758756	1.04622771703674
H	-2.85014634538789	5.31754454931620	2.17883248679403
H	1.46279257153087	6.68681171184703	-1.22176130484907
H	3.39871276921492	6.63073469186361	0.31381905891810
H	3.36581731583090	5.16561945454845	2.30653074927072
H	1.39248100642441	3.74878894385506	2.76611390243507
H	-0.01026117850461	-1.60972614996576	-1.04796053788638
H	-2.21292127399661	2.79813462089789	-1.25036259058030
H	-0.51229209610584	5.26944377741471	-0.77896468394666

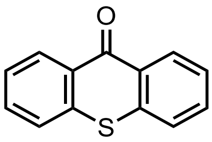

Singlet

C	-4.26418111827408	1.76225730188756	-0.36660612424045
C	-5.58310665499719	1.33392295302589	-0.37022410283185
C	-5.86625828135567	-0.03415438108231	-0.22420920348155
C	-4.83400111964824	-0.95133657694150	-0.07698343954840
C	-3.49347473182582	-0.51689934308247	-0.07292363942893
C	-3.19597955415522	0.85326300199579	-0.21910719930082
C	-1.81835790256059	1.41636178078399	-0.22934018149602
C	-0.65690763764921	0.49921230816614	-0.07068744121176
C	-0.76283968447168	-0.89752586437703	0.08764858741051

S	-2.28363975721211	-1.77490156647327	0.12121627736749
C	0.62830307942351	1.07986899479070	-0.08071396938551
C	1.77202619028535	0.30849848892699	0.06116495798166
C	1.65288047793684	-1.08219325853926	0.21842420791288
C	0.39983108582628	-1.68071078569133	0.23147106391876
H	-4.00181425951208	2.81568099166096	-0.47748269340907
H	-6.39603541961678	2.05422141114919	-0.48604621371057
H	-6.90127016001402	-0.38508152348511	-0.22565701661999
H	-5.05454051413550	-2.01577376365275	0.03681868034290
H	0.67802712109854	2.16294973765303	-0.20479137593275
O	-1.64257823621509	2.62042178680314	-0.36565191404501
H	0.30693795658245	-2.76291199907051	0.35348697808629
H	2.54606353698430	-1.70168268898499	0.33169826312164
H	2.75803558350605	0.77827299453713	0.05092549850055

Triplet

C	-4.26368681307311	1.76980371642922	-0.36542438590948
C	-5.58479399983675	1.34220966119120	-0.34703177993770
C	-5.88106302877264	-0.01956145734230	-0.20265674290338
C	-4.84125143298985	-0.94541340088338	-0.07882152202438
C	-3.50550765913075	-0.53205325621209	-0.10008806159064
C	-3.19043835314711	0.84761699179219	-0.24292619328407
C	-1.83119125058588	1.31133265155339	-0.25988851490541
C	-0.66094228114473	0.49491805911645	-0.09506710908801
C	-0.75234585492966	-0.91575914676360	0.06202477699906
S	-2.28213976288425	-1.79836353522273	0.03961089493950
C	0.62985800893062	1.08721530984145	-0.07997705249452
C	1.77329862387281	0.31636309282794	0.08437609266005
C	1.66852108874659	-1.07178714420340	0.24219280217523
C	0.40852956848299	-1.67693611768116	0.23099166354433
H	-4.03178831212387	2.83156704460870	-0.47304794719212
H	-6.39135157993249	2.07281882215891	-0.44383266527588
H	-6.91748292883321	-0.36330317765919	-0.18505683960514
H	-5.06678272180350	-2.00891263028643	0.03588090313083
H	0.71042248942385	2.16994576817741	-0.19815836173387
O	-1.63988316331360	2.61597054662976	-0.42226402003580
H	0.32050144740843	-2.75956691475868	0.35458285338558
H	2.56277208306027	-1.68439713275008	0.37491026242155
H	2.75386583257588	0.79805224943642	0.09210094672427

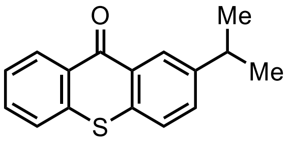

Singlet

C	-4.28137472871017	1.64329274761347	-0.83111129418725

C	-5.58441226168651	1.17204918268076	-0.88502697358960
C	-5.86212407492724	-0.14279956122835	-0.47565723606344
C	-4.83993151751574	-0.96523375575469	-0.02119508066739
C	-3.51474626507803	-0.48802071512623	0.03183304181787
C	-3.22301694118599	0.83014543725552	-0.37500680752978
C	-1.86172464563846	1.43264501805606	-0.35883306213441
C	-0.70899317025609	0.60834871146045	0.09382593980054
C	-0.81274162588390	-0.72507817039868	0.53332297571927
S	-2.31650739047752	-1.62686519673239	0.62219742824618
C	0.56416650257545	1.21426429427092	0.07229360424709
C	1.71792536047288	0.54610336654812	0.46546635361722
C	1.58414291988177	-0.78919382981383	0.90233642224648
C	0.34733273817376	-1.41502444593001	0.93762570863247
H	-4.02408467399008	2.65816879103021	-1.13878307571896
H	-6.38963096314809	1.81751033969768	-1.24288655901260
H	-6.88485004808217	-0.52634553725339	-0.51313623350654
H	-5.05532685266714	-1.98853979209494	0.29691737706745
H	0.60449863249037	2.24896895654545	-0.27444192828995
O	-1.69334463670537	2.59150675538599	-0.71843186485862
H	0.26607465918254	-2.45020907260138	1.27926833007638
C	3.07509956746353	1.22708115187596	0.42587921259242
C	4.03228830354442	0.52374783037623	-0.54915778329544
C	3.69089389238518	1.34307475273806	1.82881321518395
H	2.90620035132139	2.25017146024397	0.04921478474751
H	4.25338997293545	-0.50536155559893	-0.22025023635930
H	4.99038924161619	1.06446672411587	-0.61383358524264
H	3.60026832719446	0.46686627378126	-1.56036246883563
H	3.88991712338599	0.34861575520008	2.26188560882175
H	3.01860930451321	1.87975193381654	2.51617495344046
H	4.64922383114850	1.88613796019466	1.79096316839105
H	2.46704906766741	-1.34917581035445	1.22092606464348

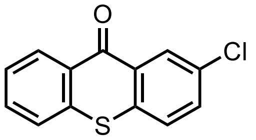

Singlet

C	-4.14140510745255	1.61795009787126	-0.00428837181324
C	-5.39447238073739	1.02427542265438	-0.01352258180518
C	-5.49591914242969	-0.37675624717220	-0.02715908047235
C	-4.35076021462502	-1.16219972284290	-0.03147688896840
C	-3.07681933877476	-0.55996252638910	-0.02214863268531
C	-2.96135157102202	0.84516847480892	-0.00828073560767
C	-1.66912805767579	1.58100690311590	0.00284644896013
C	-0.39476944654616	0.80819040336201	0.00017367043839

C	-0.31874143654678	-0.59862807352735	-0.01373707357433
S	-1.71189399703886	-1.66489731440817	-0.02917850069110
C	0.79857973709052	1.55694472427456	0.01266985701567
C	2.03039319494130	0.92240274743896	0.01135948150823
C	2.10874730356113	-0.48048408368655	-0.00325872524140
C	0.94177464146860	-1.22966781650629	-0.01562307700977
H	-4.02001656770031	2.70236143801357	0.00640678023624
H	-6.29610500352090	1.64065788187928	-0.01013077178040
H	-6.47737800618185	-0.85738437662586	-0.03444135766293
H	-4.43017917493818	-2.25225221606284	-0.04207126439852
H	0.71452375510341	2.64379557526323	0.02346785591771
H	0.99923944331004	-2.32101257105345	-0.02679395177928
O	-1.64840437968907	2.80449468373802	0.01455345925216
Cl	3.50193152751750	1.86102477511162	0.02824269316283
H	3.08375422188685	-0.97031817925701	-0.00465923300149

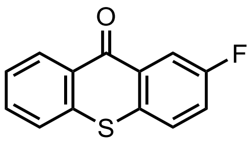

Singlet

C	-4.23912835113695	1.73784376766159	-0.01742080525130
C	-5.48555341521875	1.13107538469620	-0.03967459404537
C	-5.57157523741552	-0.27014816138260	-0.09475593149839
C	-4.41807479328509	-1.04227458665072	-0.12644109509092
C	-3.15029338219701	-0.42678714277186	-0.10419322096619
C	-3.05036725964070	0.97870099838431	-0.04923844217420
C	-1.76683006500034	1.72843362474265	-0.02282087833803
C	-0.48345649092676	0.97221019587326	-0.06563273859516
C	-0.39127935644898	-0.43308194474341	-0.12051334890236
S	-1.77403830223990	-1.51553602419718	-0.14775347342002
C	0.70077001521024	1.73554437730694	-0.04960363464276
C	1.93061555480737	1.10767490150025	-0.08782735605370
C	2.03464910045378	-0.28787018383387	-0.14124164735313
C	0.87563009955188	-1.05059445687951	-0.15711809579025
H	-4.12944564171506	2.82271003997862	0.02527815527702
H	-6.39392940780612	1.73698143111795	-0.01464336686411
H	-6.54774633204522	-0.76118681487265	-0.11336559621207
H	-4.48564507067680	-2.13233770324679	-0.16955054841601
H	0.61865850738330	2.82216001305740	-0.00703252262068
O	-1.75908040110006	2.95111495570626	0.03141150612014
F	3.05382399926801	1.83754171668450	-0.07438373120509
H	3.02173304973778	-0.75319369354832	-0.17016718735775
H	0.94376318044088	-2.14045069458303	-0.19930144659968

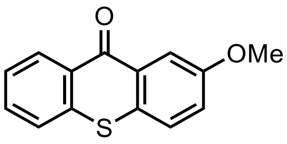

Singlet

C	-4.27100604881051	1.59693921974824	0.17446122303328
C	-5.53675288998149	1.04999756998749	0.03215665761230
C	-5.67230926304418	-0.33035512380361	-0.19417937436335
C	-4.54814741266566	-1.14061017467076	-0.27403409971073
C	-3.25967702108285	-0.58622123172664	-0.12926476676807
C	-3.11030450116793	0.79807626607426	0.09788734468393
C	-1.80259984082955	1.48513413287543	0.26498001558663
C	-0.54871014075386	0.68742929640869	0.19182597981275
C	-0.50786039053903	-0.70000119353222	-0.03042111031786
S	-1.92357862423603	-1.71539761896200	-0.25115438645275
C	0.65840337781578	1.39874121116458	0.35837847371139
C	1.88629492335836	0.74839285514167	0.30405246184767
C	1.91736263932357	-0.64580961872115	0.07778850765469
C	0.74178883995420	-1.35472262545842	-0.08555725517164
H	-4.12322650769732	2.66362551472003	0.35095514996537
H	-6.42260520419714	1.68589070259823	0.09542803123863
H	-6.66455930897204	-0.77400415640689	-0.30873976610957
H	-4.65406235202079	-2.21418280789461	-0.44992888515590
H	0.56355734015817	2.47005160386292	0.52817798578987
O	3.08803888772801	1.35302151063975	0.45262782802636
H	2.88774018591193	-1.14435070160732	0.03558186175663
H	0.77811676208973	-2.43319313621451	-0.25947562794023
O	-1.75572363176060	2.69444615749357	0.46001995594670
C	3.11719397727070	2.74545159756079	0.68474349653190
H	2.59019085831391	3.01349040130766	1.61785945471340
H	2.66233145691147	3.30699011497429	-0.15070216480763
H	4.17502388892315	3.02561023444049	0.77476300888619

Calculated coordinates of phenylethyl thianthrenium salt:

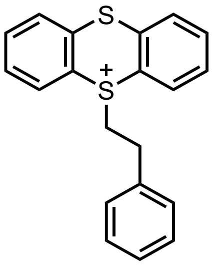

Singlet

S	-1.14427553808528	2.46721542900947	1.50287356387674
C	-2.44796018568802	3.28917534734357	0.61523405418551
C	-2.29366952925438	3.63842162545916	-0.73776636661337

S	-0.80973459234719	3.35633940654512	-1.67190268904773
C	0.46315994785136	3.60069631024284	-0.45796567900265
C	0.32982683510390	3.25803363081854	0.89866497534018
C	1.68155353771901	4.14733315576530	-0.88679790294152
C	2.72290343578110	4.34516398804768	0.02156089550928
C	2.56387265454165	4.02810960694944	1.37552449262392
C	1.35836410193364	3.48792073718346	1.82136648770391
C	-3.38353347176863	4.22318588898352	-1.39891751948231
C	-4.58072877043511	4.45002310619260	-0.71781463057862
C	-4.70841186238571	4.12466347823949	0.63738901540279
C	-3.63458622764604	3.54724939524993	1.31337068362703
H	-3.28158000118033	4.51511969029996	-2.44644749316837
H	-5.41854194458948	4.90661127044324	-1.24933038556529
H	-5.64153050167081	4.32236093526375	1.16815252569589
H	-3.71630105862989	3.28764075734166	2.37128045244702
H	1.80157116262169	4.43368658910172	-1.93398811045814
H	3.66411922124542	4.77276161585738	-0.33088908421012
H	3.37597657600122	4.20255223181189	2.08366958262978
H	1.21764387596634	3.23091933268118	2.87362618284356
C	-1.08288755884551	0.78827308373992	0.70906682852067
C	0.12284958760304	-0.00811984203347	1.20334154793764
H	-1.06971517626763	0.94386942532869	-0.37791534743957
H	-2.04057433373394	0.33325973614747	1.00269731013796
C	0.12629073506200	-1.36383664555390	0.52977594492737
H	0.07888393952138	-0.11953748159691	2.29873811747792
H	1.04960810502151	0.53485616637099	0.96116659571542
C	-0.56070238874957	-2.44498273467583	1.10055250799327
C	-0.59022348002392	-3.68424303459367	0.45671622272811
C	0.06443236964682	-3.85320748690909	-0.76691245518599
C	0.74876297118924	-2.77947828256530	-1.34436642676615
C	0.77788690587612	-1.54083144654025	-0.69965911914747
H	1.32271801606592	-0.70604617639203	-1.15102815873288
H	1.26758956401394	-2.90907978151249	-2.29700664104158
H	-1.06665230221180	-2.32139792486598	2.06280550268398
H	-1.12024509857024	-4.52242723295766	0.91495631063823
H	0.04601047931821	-4.82351387022143	-1.26838179126440

Calculated coordinates of fragmentation products:

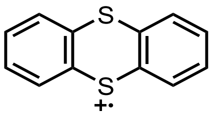

Doublet

S	-1.01387259975569	4.48890104424526	1.98655400300225
C	-2.41172276222772	4.55936663989084	0.95173313649123
C	-2.41797602043189	4.91969560923794	-0.42137490260198

S	-1.02888711045162	5.36782993836606	-1.36917112704680
C	0.36900590696369	5.29670746051935	-0.33460379831258
C	0.37537080061059	4.93637094580631	1.03850068149632
C	1.58513025128265	5.63798290154483	-0.96694885647374
C	2.77313490800745	5.62146697663478	-0.25528770396479
C	2.77953293126032	5.26290638521172	1.10815946839916
C	1.59765634487964	4.92473393438777	1.74623330811232
C	-3.64022952610546	4.93103402141899	-1.12925348771474
C	-4.82210341570269	4.59272055063494	-0.49128645585732
C	-4.81576244341513	4.23419293869671	0.87216784012903
C	-3.62780129777567	4.21783976606729	1.58393770013871
H	-3.64385491907818	5.21068248127470	-2.18553833360750
H	-5.76052706409833	4.60473793122891	-1.04932103106146
H	-5.74907705375574	3.96733894834831	1.37171544974520
H	-3.62165293026867	3.93924244502106	2.64042878526165
H	1.57888705095718	5.91644972077196	-2.02348701674750
H	3.70648187964632	5.88819821215362	-0.75484659453783
H	3.71798827258368	5.25073210642025	1.66613121716475
H	1.60143879687530	4.64494904211837	2.80246771798562

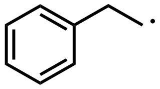

Doublet

C	-0.70366935098752	1.68908898908037	0.56344326384539
C	-1.76829299779579	0.89113363629548	0.12167964913140
C	-1.53620167896432	-0.34751178356209	-0.48382475707443
C	-0.22922772121355	-0.81041075833715	-0.65486610134428
C	0.84187027825916	-0.02521633857254	-0.21599230693755
C	0.60508776080887	1.21125901825773	0.38767167149601
H	1.44609566477449	1.81982456208737	0.73161068045012
H	-2.79473325470451	1.24519724995538	0.25553367904076
H	-2.38115433981177	-0.95450388868928	-0.81952519416239
H	1.86831365488083	-0.37894649898252	-0.34475660365135
H	-0.04520601926121	-1.77919698033886	-1.12613799281556
C	-0.94920284074508	3.03962072195090	1.20601473155086
H	-2.04426701691233	3.17082987220440	1.34252927886861
H	-0.66392792089395	3.84170173499737	0.49712129068717
C	-0.23911792033071	3.24848181435756	2.50104078972132
H	-0.03243974243837	2.40235580398928	3.16172505975380
H	-0.06325655466424	4.25841284530658	2.88050286144008

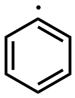

Doublet

C	-0.61421792899802	1.83351426607068	0.66017568264097
C	-1.74744896204828	1.09860188127919	0.37871603326966
C	-1.54851831039507	-0.15989772032757	-0.21700514380537
C	-0.25288316419871	-0.61028130244723	-0.49547354444960
C	0.85905649072652	0.18156418875158	-0.18692153321381
C	0.68527409561748	1.44351299239140	0.40944914157281
H	1.54525206714242	2.07225717558292	0.65660388435924
H	-2.75416381128852	1.46237101662281	0.60241760693628
H	-2.41205524983085	-0.78500768791725	-0.46180478025244
H	1.86816321828252	-0.17738878297648	-0.40894130159506
H	-0.10806844500949	-1.58973602703004	-0.95739604546268

Calculated coordinates of parent arenes in ground state:

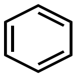

Singlet

C	-0.62064553082752	1.87781028162033	0.67702445727362
C	-1.72092693321760	1.08489123875789	0.36845307112228
C	-1.53670658336125	-0.16226134864716	-0.21900622601288
C	-0.25202373815649	-0.61669881778362	-0.49753785067402
C	0.84825851813773	0.17622040826972	-0.18896622606788
C	0.66403799874693	1.42337207604101	0.39849300386438
H	1.52000590632047	2.04021144013413	0.63834261786014
H	-2.72026773544724	1.43836842224712	0.58533480319326
H	-2.39267612170235	-0.77909900322949	-0.45885435969168
H	1.84759919437048	-0.17725727025299	-0.40584842797032
H	-0.10857522075627	-1.58721522490790	-0.95382840641226
H	-0.76408975410689	2.84832779775098	1.13331354351534

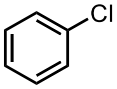

Singlet

C	-0.62821592679845	1.92899121737769	0.70205089102129
C	-1.72593673330948	1.13449022707594	0.39114357716561
C	-1.54939994811710	-0.11258966382757	-0.19660948443274
C	-0.26122869643404	-0.55413003705553	-0.46834660846356
C	0.84616114318430	0.22741862571635	-0.16655387426238
C	0.65455138225245	1.47230846257979	0.42130078011028
H	1.51405399121152	2.08455705931778	0.65917226486851
H	-2.72739984715050	1.48258896551918	0.60571560277861
H	-2.39638475017390	-0.73680222599639	-0.44123544363136
H	1.83908671665187	-0.13540968240723	-0.38868180466693
Cl	-0.02973779918351	-2.12283434906407	-1.20088849117884
H	-0.77153953213317	2.89807140076406	1.15983259069150

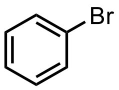

Singlet

C	-0.62994198369548	1.94082968613949	0.70605684599224
C	-1.72780618830252	1.14590974077066	0.39734179875173
C	-1.55169455372793	-0.10255362886263	-0.18909507351301
C	-0.26327723012701	-0.54126628818895	-0.46114293787228
C	0.84511014435727	0.23767552839128	-0.15914380847556
C	0.65299126303763	1.48367873033660	0.42752676248428
H	1.51264402136748	2.09599776983117	0.66480244258413
H	-2.72934412984753	1.49430829813908	0.61128324738031
H	-2.40131611609713	-0.72399607710857	-0.43091738795062
H	1.83958503674598	-0.12202766445737	-0.37855648719759
Br	-0.00954374161459	-2.25335416590501	-1.27176325665748
H	-0.77338652209616	2.91146807091425	1.16051785447388

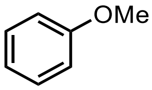

Singlet

C	-0.63554688045685	1.87499100006557	0.67787601180035
C	-1.69732229614465	1.04635303639569	0.34856315389171
C	-1.47995316043076	-0.19760817335986	-0.24064110818800
C	-0.17558468929739	-0.61420600910481	-0.50240505896334
C	0.89782994221513	0.21763034085349	-0.17406856388910
C	0.66519951473553	1.45024258149806	0.41147839986190
H	1.50464045901872	2.08545436720605	0.66210517789134
H	-2.71293392487440	1.36194771824935	0.54866813425173
H	-2.32365951200217	-0.82397017739442	-0.48643977470881
H	1.90147253914081	-0.12396541047137	-0.38663417577389
O	0.14981529258378	-1.80823517026469	-1.07091682613005
H	-0.81400465738911	2.83832266470817	1.13500790983198
C	-0.89879661758905	-2.69307630985079	-1.42282296070748
H	-0.42107568500775	-3.56933722984728	-1.85283611348922
H	-1.56762228589102	-2.24544956538582	-2.16294654410425
H	-1.48055803861082	-2.99110366329731	-0.54621766157489

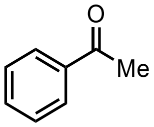

Singlet

C	-1.14653518638813	3.03377820686954	0.46783285311300
O	-2.08164404648340	2.27611898707067	0.30726515959305
C	-1.37786999904393	4.47127078483882	0.81270043058700
C	-0.32968381764046	5.37546470482098	0.99836724212273

H	0.69577025563495	5.05099793879378	0.89486771199196
C	-2.69479480727775	4.91878043593640	0.95266685621841
C	-0.59257877532547	6.70159754340966	1.31780315822451
H	-3.49573420065953	4.20821049030492	0.80595774699762
C	-2.95720804850531	6.24104781296849	1.27173619085934
C	-1.90572196482307	7.13577723291753	1.45482052511038
H	0.22576953829985	7.39454431734959	1.45963201217143
H	-3.97962237449382	6.57795222402467	1.37845496748196
H	-2.11062688180051	8.16858145868428	1.70357252126978
C	0.27798774968015	2.54299727274292	0.32403650396466
H	0.26152258160913	1.48511723721672	0.07803027593651
H	0.83552045120157	2.69360665388381	1.25032270141281
H	0.80007952601576	3.09190669816721	-0.46191685705512

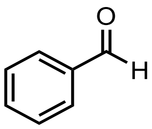

Singlet

C	-1.02888875352850	3.00626742005658	0.46212165234793
O	-1.85817519423995	2.14499304995731	0.28693151155981
C	-1.32571546651405	4.41333648820328	0.79796617668864
C	-0.27321131913256	5.30968631091159	0.98260622002932
H	0.74725979271844	4.96223295270838	0.87504360687528
C	-2.64584540934822	4.85210979601046	0.93380050278796
C	-0.53216199914540	6.63594368734141	1.30259925367094
H	-3.44715715935008	4.14131954851187	0.78578785066618
C	-2.90339929455226	6.17479346161950	1.25224474509039
C	-1.84725620967278	7.06671979666651	1.43710169231257
H	0.28416486422291	7.33048286471553	1.44690476964029
H	-3.92378826011092	6.51768825322818	1.35826058171983
H	-2.05272025144364	8.09932229266134	1.68641949271766
H	0.05253466009701	2.77424407740803	0.37661194389319

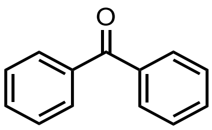

Singlet

C	-1.38805882752463	2.12633238134542	-1.13797284208747
O	-2.43393020998501	1.56686415157283	-0.86166488783704
C	-1.05748729988774	3.44701793109023	-0.51973340963068
C	-0.31879713745179	4.42402692786248	-1.19035755639101
H	0.06045288842920	4.22613363109685	-2.18265005458305
C	-1.57051213414152	3.72929906714798	0.74876129883904
C	-0.09349337971446	5.66084846574936	-0.59876631275118

H	-2.16037875428830	2.97333386927395	1.24756994950626
C	-1.32564410334197	4.95498691528620	1.34747495618211
C	-0.58699756683666	5.92413996970344	0.67358074065374
H	0.46665238592511	6.41786546345892	-1.13094340420562
H	-1.71370449275007	5.16033272334454	2.33612013337708
H	-0.40132704371185	6.88353317656913	1.13792158056037
H	1.35982861684856	2.30917228049513	-1.21711947926617
C	0.95429679587700	1.67402863870830	-1.99145386005905
C	-0.42700949555843	1.49625367969765	-2.09405859092252
C	1.81235437141650	1.01289215870898	-2.86134797448851
C	-0.93472860509650	0.64291983675650	-3.07722817143439
H	-2.00405429659992	0.49517345287229	-3.13168360320152
C	-0.07901152199716	0.00104215275252	-3.95840459606556
C	1.29725043913699	0.18389201671939	-3.85088165758609
H	-0.48077531241647	-0.64690481769339	-4.72583272990797
H	2.88179964706997	1.14379808701563	-2.76524195395795
H	1.96572503659915	-0.32331215953437	-4.53386757474280

Calculated coordinates of aryl chlorides in ground state:

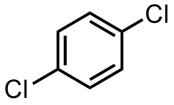

Singlet

C	-0.62351132416202	1.87953019976308	0.67783634659522
C	-1.72196876992444	1.08768280466560	0.36976812869412
C	-1.52749091732956	-0.15586057191579	-0.21617570556879
C	-0.24915763410487	-0.61841980479541	-0.49834657118943
C	0.84930004361797	0.17342783568150	-0.19027859675438
C	0.65482217673604	1.41697216229318	0.39566275047477
H	-2.72169704838722	1.43572253034662	0.58402820590671
H	1.84902809657248	-0.17461201105667	-0.40453944954876
H	-0.11125956458630	-1.58779449007245	-0.95420745114568
H	-0.76140779180147	2.84890499376832	1.13369745981117
Cl	-2.90687149572931	-1.14905810069919	-0.60268089482234
Cl	2.03420230509870	2.41017388002121	0.78215896854740

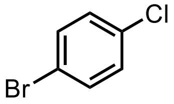

Singlet

C	-0.63358848423701	1.87171099098171	0.67714818489484
C	-1.73226337081763	1.07984568744650	0.36807988001313
C	-1.53777036815674	-0.16304133880878	-0.21961737742648
C	-0.25929459126249	-0.62666064886568	-0.50021116997986
C	0.83991154176842	0.16478006083351	-0.19141477430666

C	0.64349406067995	1.40823312664144	0.39309951972651
H	-2.73237350284963	1.42736860502959	0.58169133235495
H	1.83833593564139	-0.18637599202933	-0.40614617667787
H	-0.12242343626891	-1.59611059934142	-0.95640905177176
H	-0.77434122243397	2.84020102438744	1.13371415794716
Cl	-2.91733699612195	-1.15339416055073	-0.61095435332315
Br	2.15163851005857	2.50011267227575	0.80794301954918

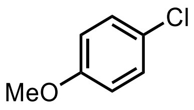

Singlet

C	-0.63409161552198	1.86387674546145	0.67008400696032
C	-1.70359128752957	1.04542614520078	0.35150868681455
C	-1.47857825916257	-0.19807718740369	-0.23384726973549
C	-0.17578421356209	-0.61670844899264	-0.49780007615393
C	0.89544861824013	0.21801486474177	-0.16979571373937
C	0.66999010426621	1.45253754638545	0.41181815119003
H	1.49919977586469	2.09711193891339	0.66466402839388
H	-2.71284966435140	1.37143100059322	0.55692636392277
H	-2.32464401263002	-0.82263342390108	-0.47512797369902
H	1.90066237984089	-0.11956714710235	-0.37993957391365
O	0.14998171991618	-1.80869552690539	-1.06571846655821
C	-0.89928408061818	-2.69105229310084	-1.42568830478623
H	-0.42126299872857	-3.56492207938508	-1.85960940364416
H	-1.56455369727976	-2.23755261429700	-2.16520238323904
H	-1.48263224293159	-2.99360750951879	-0.55192845105396
Cl	-0.91982274181237	3.42623214731083	1.39870319924150

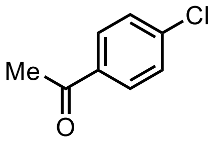

Singlet

C	-1.14654998519938	3.03523308573805	0.46805925574138
O	-2.08416019741575	2.28082593134033	0.30727027306185
C	-1.37652664959350	4.47160867256515	0.81345750678683
C	-0.33119232004006	5.37807717832653	0.99998222587196
H	0.69576638689583	5.05855659694153	0.89788207505336
C	-2.69252467974699	4.92148982844060	0.95396887932040
C	-0.58700262478327	6.70416386294919	1.31882882233629
H	-3.49555604054091	4.21318932972302	0.80781667702576
C	-2.96316229835674	6.24047029186098	1.27220166787701
C	-1.90364483572391	7.12462872069243	1.45194010628738
H	0.22107744989595	7.40600877876800	1.46302403564650
H	-3.97992810518526	6.58835914212553	1.38130517462787
C	0.27706209348911	2.54386997903082	0.32388578920264
H	0.26024048336426	1.48608937527306	0.07778246378820

H	0.83502319241287	2.69340948117697	1.25013885649185
H	0.79911019279581	3.09192247857778	-0.46278501304685
Cl	-2.23371709626807	8.78669173546998	1.84959417092757

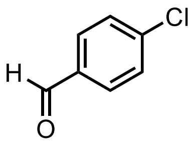

Singlet

C	-1.10158953929120	3.01000322182849	0.46454142561777
O	-1.94894682328645	2.16587510054828	0.29240938311498
C	-1.37417795929569	4.42082548670952	0.80101649306295
C	-0.31140423573921	5.30489324388843	0.98257075558401
H	0.70592251862679	4.94968879336962	0.87326515767391
C	-2.68646222397044	4.88097373695818	0.94178922465990
C	-0.54356495856021	6.63456107535655	1.30248463644166
H	-3.50029054833383	4.18387909366106	0.79732160884245
C	-2.93242600857425	6.20384512447210	1.25995737188773
C	-1.85556085975937	7.07095970963720	1.43808410511563
H	0.27339040004218	7.32607644347551	1.44595227281194
H	-3.94190963122665	6.57139133083263	1.37174062662663
Cl	-2.16224938584594	8.73540608426897	1.83826482099861
H	-0.02556894678570	2.75771165699345	0.37563355656183

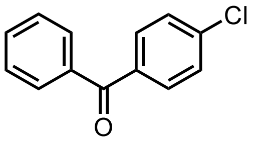

Singlet

C	-1.36915410987702	2.11018081770035	-1.11188457044627
O	-2.40631589740457	1.54346072510118	-0.81811675416092
C	-1.04443299873270	3.43675011451379	-0.50449224246676
C	-0.29781953715841	4.40865947349528	-1.17280174064968
H	0.09955289817662	4.20393480044994	-2.15629317713610
C	-1.57710068830187	3.73823339087745	0.75139480081776
C	-0.08190788253782	5.65496775273966	-0.60144846996465
H	-2.17390323791267	2.99066612157910	1.25447392359509
C	-1.35129245297097	4.96958233456579	1.34257845656367
C	-0.60387938270023	5.92212204000529	0.65715580678327
H	0.48251928967099	6.41314852106431	-1.12394473520077
H	-1.75104763032114	5.19849114057457	2.31956644362555
H	1.37989230779733	2.27884019697819	-1.20309832606978
C	0.96551950162718	1.65864191342689	-1.98503468308865
C	-0.41729458687830	1.48826780485808	-2.08108278577027
C	1.81474578674515	1.00703560291324	-2.87053336491028
C	-0.93564040592883	0.65164641866373	-3.07299925566242

H	-2.00587100135580	0.50891743403494	-3.12260579767946
C	-0.08859490878614	0.02038561692834	-3.97013048472013
C	1.28903969998497	0.19610696138494	-3.86948970532912
H	-0.49833097468366	-0.61362685016339	-4.74487993977719
H	2.88536439660358	1.13124401466600	-2.77932943220471
H	1.95053575708925	-0.30286562979983	-4.56514689101178
Cl	-0.32404596014495	7.47905339744212	1.38568336486362

Calculated coordinates of aryl bromides in ground state:

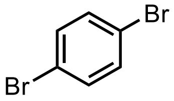

Singlet

C	-0.62333200626393	1.87998338219292	0.67883753913998
C	-1.72291295873854	1.08831260730048	0.36930569771278
C	-1.52631873421460	-0.15434980291142	-0.21670859079511
C	-0.24933699954553	-0.61886882823264	-0.49935606417829
C	0.85024405463294	0.17280209444100	-0.18982470686474
C	0.65364975800293	1.41546101720526	0.39619584492551
H	-2.72114594592062	1.44009893345754	0.58405449047599
H	1.84847706824868	-0.17898252664963	-0.40457618994708
H	-0.10830848895686	-1.58729477263749	-0.95609902656499
H	-0.76435982355558	2.84841075129100	1.13557826524087
Br	-3.03477498504288	-1.24327625396755	-0.63709978714357
Br	2.16210713735400	2.50437282651053	0.81661571899865

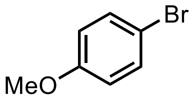

Singlet

C	-0.63078781367284	1.85206558174722	0.66849769940738
C	-1.70207404451311	1.03976760924511	0.34231653633021
C	-1.47774011533852	-0.20106920258288	-0.24974657754073
C	-0.17478243172250	-0.62111594078223	-0.51154300183599
C	0.89729177879263	0.21171309287024	-0.18094846073734
C	0.67315198314377	1.44370618963955	0.40748428922132
H	1.50547481906089	2.08293760808108	0.66303413454043
H	-2.71253213933872	1.36275228580490	0.54584348880201
H	-2.32473172700199	-0.82233397180919	-0.49629476116882
H	1.90247115908074	-0.12571803641424	-0.39162128902418
O	0.15002721789582	-1.81303226950086	-1.07841802171653
C	-0.89899357802707	-2.70230727266880	-1.42316947352293
H	-0.42053559863026	-3.58064568336125	-1.84733643917767
H	-1.56804711416936	-2.25996770233990	-2.16588773454236
H	-1.47789736853975	-2.99429466733552	-0.54295049110700

Br	-0.94210724301973	3.54935653740677	1.48978692207220

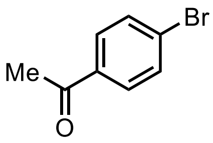

Singlet

C	-1.14382480843760	3.02713887592217	0.46339816549112
O	-2.08201977551649	2.27412090032130	0.30055428416912
C	-1.37390934683045	4.46445387484936	0.80722825992734
C	-0.32903130081190	5.37153860604837	0.99230564177686
H	0.69827321189098	5.05358843408839	0.88779562805921
C	-2.69008994448101	4.91256478082683	0.94938467902556
C	-0.58551564673904	6.69773628110345	1.31384356781491
H	-3.49303372505348	4.20409374152280	0.80350809515185
C	-2.96200824062448	6.23115211469788	1.27067755505093
C	-1.90247299907220	7.11404415324711	1.45092173697737
H	0.22507506239540	7.39682196843950	1.45650773335525
H	-3.98015742140012	6.57390597336341	1.38131243102319
C	0.27943614826391	2.53375448671403	0.32545662923891
H	0.26205840259859	1.47512493276411	0.08305582733846
H	0.83454425790715	2.68596442865729	1.25299684628997
H	0.80468487926750	3.07836139429336	-0.46147700834239
Br	-2.26769378735676	8.93022952214062	1.89688289465234

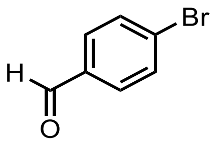

Singlet

C	-1.15231581248186	3.04989416010456	0.46899036631551
O	-2.00128887689857	2.20721666278980	0.29943264213389
C	-1.42103700696832	4.46159190353411	0.80627931454690
C	-0.35629551997698	5.34332824844282	0.98606958419580
H	0.66015821141820	4.98644160202707	0.87327255223466
C	-2.73232332504962	4.92303680060371	0.95051386303698
C	-0.58550246157496	6.67338142282986	1.30966195033378
H	-3.54786370747518	4.22775408962468	0.80676034108417
C	-2.97591968813321	6.24617337787188	1.27240735767851
C	-1.89666607323700	7.10912888262618	1.45021142988273
H	0.23570879120002	7.36000266785604	1.45144679963410
H	-3.98579132303157	6.61136110516580	1.38665558536202
Br	-2.23036077227095	8.92933669856130	1.89880003155968
H	-0.07666243552001	2.79541237796216	0.37761818200128

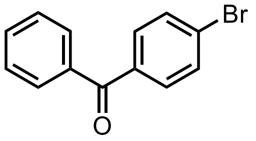

Singlet

C	-1.36721672419241	2.44259003572939	0.29381513597847
O	-2.47562977311195	1.95263561019130	0.17642410646169
C	-1.22269087593470	3.88233996191681	0.67033185033118
C	-0.14625483231731	4.35856682621966	1.42064724009424
H	0.64364765635350	3.68573049325439	1.72176173936423
C	-2.24765797938772	4.76461051777911	0.32122281491105
C	-0.08998006699312	5.68818179195760	1.81691603354290
H	-3.09003477817147	4.38693692091315	-0.24089034463994
C	-2.19344028855449	6.09876052163915	0.68966046876625
C	-1.11225554572232	6.54789836868378	1.43980778550596
H	0.73550647043552	6.04978263911092	2.41193490680021
H	-2.98083495318079	6.78127517130964	0.40584489517674
Br	-1.03852252685259	8.37736726484433	1.96957493724610
C	-0.22073412331036	0.24159987318927	0.32751726083861
C	-0.14626263378088	1.61220357775623	0.06673337176252
C	0.87237384664775	-0.57687273262229	0.09044378630814
C	1.03243948058575	2.14438675130352	-0.46050152256000
C	2.04445258845449	-0.03955561248573	-0.43514516370172
C	2.11947532067372	1.31902827292999	-0.71917641659649
H	-1.14507062092192	-0.16329996184771	0.71470294547942
H	0.81163030941468	-1.63458868169394	0.30865705041352
H	1.09222290620496	3.19802204981129	-0.69309592913812
H	3.02291428009023	1.73573517068403	-1.14357191316849
H	2.89502286357143	-0.68001483057390	-0.62698503917647

Calculated coordinates of other aryl psuedohalides in ground state:

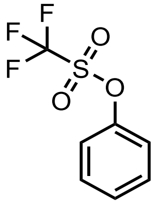

Singlet

C	-0.78204894045528	2.02499598641585	0.75111028840710
C	-1.82402218121171	1.16528700738829	0.45413830235361
C	-1.52708610124175	-0.04039290623930	-0.17077437921404
C	-0.21126403815839	-0.35838158369403	-0.48974163115585
C	0.81619731491413	0.52755327239897	-0.18395353009465
C	0.53596270473035	1.73568144409285	0.44434402164356
H	1.31430781160289	2.44272694982645	0.69034836172215
H	-2.83694073170327	1.44114958220870	0.70768767077797

H	-2.32630466332103	-0.72846249513826	-0.40901502069073
H	1.83910309868796	0.28110639135577	-0.43270611375691
S	-1.26160763597388	4.55921125779196	0.57431159479194
H	0.01346498948462	-1.29734847764509	-0.97666862828120
O	-0.00360236729694	5.01817993881185	0.08072727149789
O	-2.40322653142863	4.45510266564143	-0.27522699789762
C	-1.72779407554182	5.62011607700543	2.03846064732230
F	-0.76747649489838	5.60342960699718	2.95639202659370
F	-1.89183892252702	6.86287616427493	1.59065221913031
F	-2.86399007659412	5.18992139534861	2.57638002426349
O	-1.07845315906771	3.22752772315839	1.44003387258698

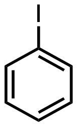

Singlet

C	-0.61521616061352	1.74570759177036	1.08358973376088
C	-1.72681275796642	1.00866551225746	0.69370147823798
C	-1.54347918763394	-0.14721852296890	-0.05788981657281
C	-0.26530361673872	-0.56069131557360	-0.41441758425718
C	0.83691731412706	0.18634860119074	-0.01556649880968
C	0.66892467761705	1.34426811124868	0.73643936230085
H	1.52719007797299	1.92278406076915	1.04537340226030
H	-2.72127574275028	1.32774184094365	0.96928547815298
H	-2.40694275787355	-0.72302802051156	-0.36346918263300
H	1.83550951433388	-0.12858592481626	-0.28777893718683
I	-0.88161093383211	3.50596567992855	2.22210565539252
H	-0.12901042664243	-1.45980761423827	-0.99958309064602

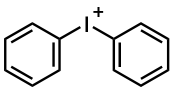

singlet

C	-2.94989146666077	3.53026259642115	0.93021899473390
C	-2.52151675829691	3.98459526980155	-0.31712852543803
I	-0.73502641772090	3.13515480727088	-1.14356746623994
C	0.71515848516342	4.39806628890700	-0.19621327566617
C	1.29686544933878	3.95011981446928	0.98994715217319
C	1.01516099321746	5.61997449943308	-0.79824592595068
C	1.95161969052534	6.43858342312620	-0.15864966044556
C	2.55454252750108	6.02562769602380	1.03408728849184
C	2.23189691615226	4.79006827206600	1.60420316613902
C	-3.18581932825440	4.96008043266443	-1.06081939165462
C	-4.34585995363294	5.51188555393641	-0.50660811670176
C	-4.80368252067845	5.08626000360414	0.74424500633594
C	-4.11161245169397	4.10255743759230	1.45844646884323

H	-2.82734243289090	5.28750557927270	-2.03801174855343
H	-4.89094009248899	6.27735011437203	-1.06348764072274
H	-5.71143193846843	5.52329766693123	1.16583437286164
H	-4.47494094168073	3.76934340542229	2.43320676274060
H	0.54588271754207	5.93626721418911	-1.73105070152083
H	2.20906044425553	7.40255183314838	-0.60320950773306
H	3.28617100810358	6.67257077891241	1.52281073861151
H	2.70769658347223	4.46928294903392	2.53365036232654
H	-2.41032511583595	2.75913765198697	1.48227539228021
H	1.04331460303162	2.98486671141469	1.43103625508918

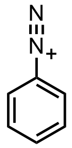

singlet

C	-0.61797792088719	2.05317317487209	0.64461154913356
C	-1.77001098984128	1.27817058741563	0.38104540153419
C	-1.57437481295408	0.00852563808464	-0.14533912998045
C	-0.27442923640229	-0.46038232607946	-0.39486937845193
C	0.85316559750593	0.33108884406912	-0.12287060342152
C	0.70112823314925	1.60668570712887	0.40346150086987
H	1.55789322270226	2.24559284812087	0.62518307274966
H	-2.76777836631155	1.67035509531867	0.58621121967097
H	-2.43833706388237	-0.62228967287101	-0.36356292707136
H	1.85577449215947	-0.05165219368766	-0.32359049326886
N	-0.79029651728013	3.31317898696598	1.16546752258543
H	-0.13738453428666	-1.46210113775490	-0.80922895154207
N	-0.92967210367136	4.33255444841715	1.58624121719249

triplet

C	-0.58764147800149	2.10187025443020	0.66334187967160
C	-1.77854492479327	1.25029069813441	0.37063543375526
C	-1.59088865721467	-0.01631706961579	-0.15471956200402
C	-0.30448147113955	-0.49790619161674	-0.40925489607311
C	0.85532041975068	0.35421172644730	-0.11348682384777
C	0.72312862811427	1.62711218803409	0.41124421643436
H	1.59112169788451	2.25157156550562	0.62668451052151
H	-2.77082758791199	1.65308284313382	0.58068091205108
H	-2.46372530292648	-0.63722047855459	-0.36835631640878
H	1.85714347641765	-0.03517928097548	-0.31690835498908
N	-0.77753153704772	3.31792128271820	1.16628341751750
H	-0.13918597405852	-1.49381543212312	-0.82283906324795
N	-0.96379728907342	4.36505789448208	1.59991464661939

SPECTROSCOPIC DATA

^{1}H NMR of arylethylamine 3

$CDCl_3$, 23 ºC

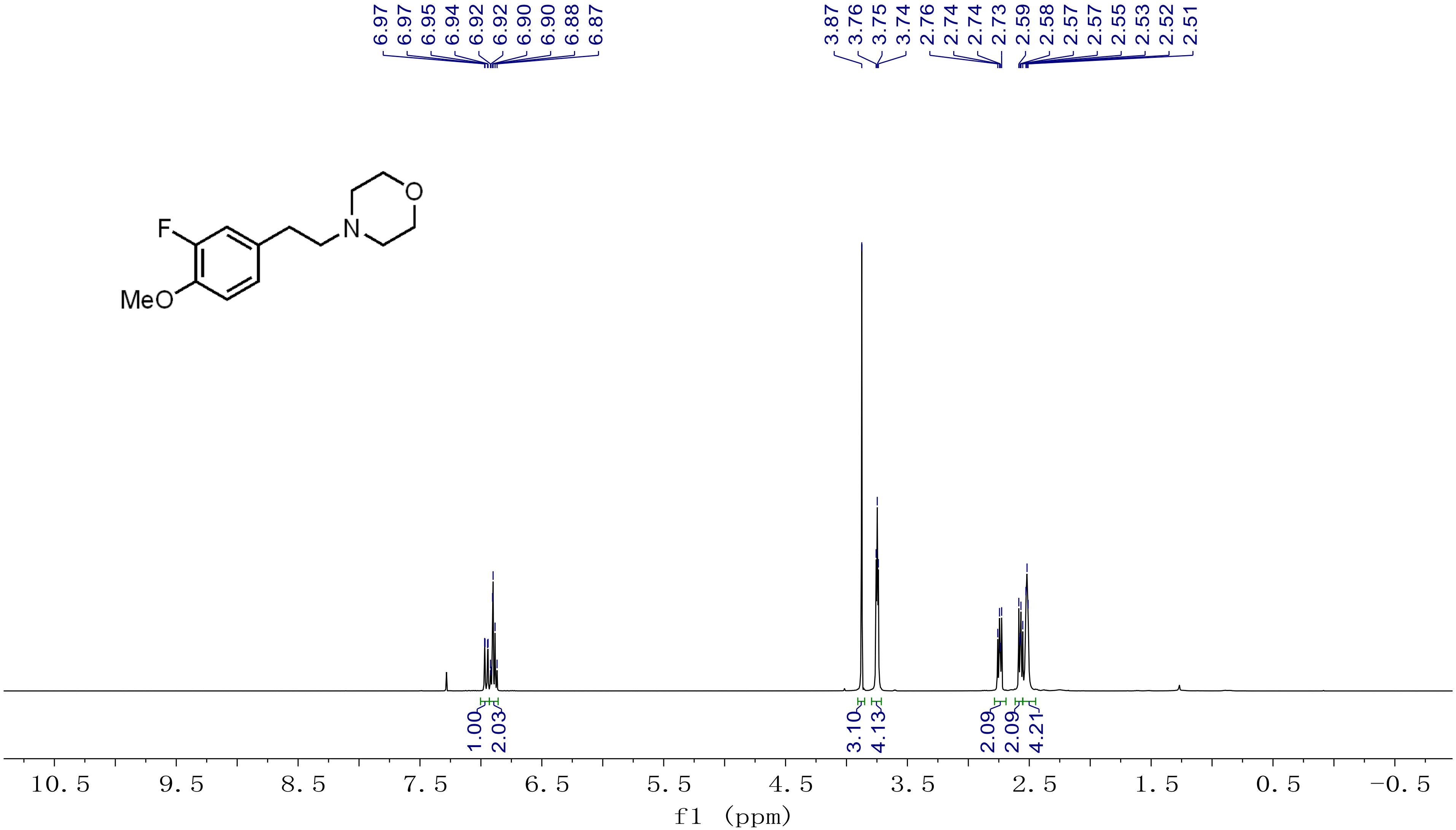

^{13}C NMR of arylethylamine 3

$CDCl_3$, 23 ºC

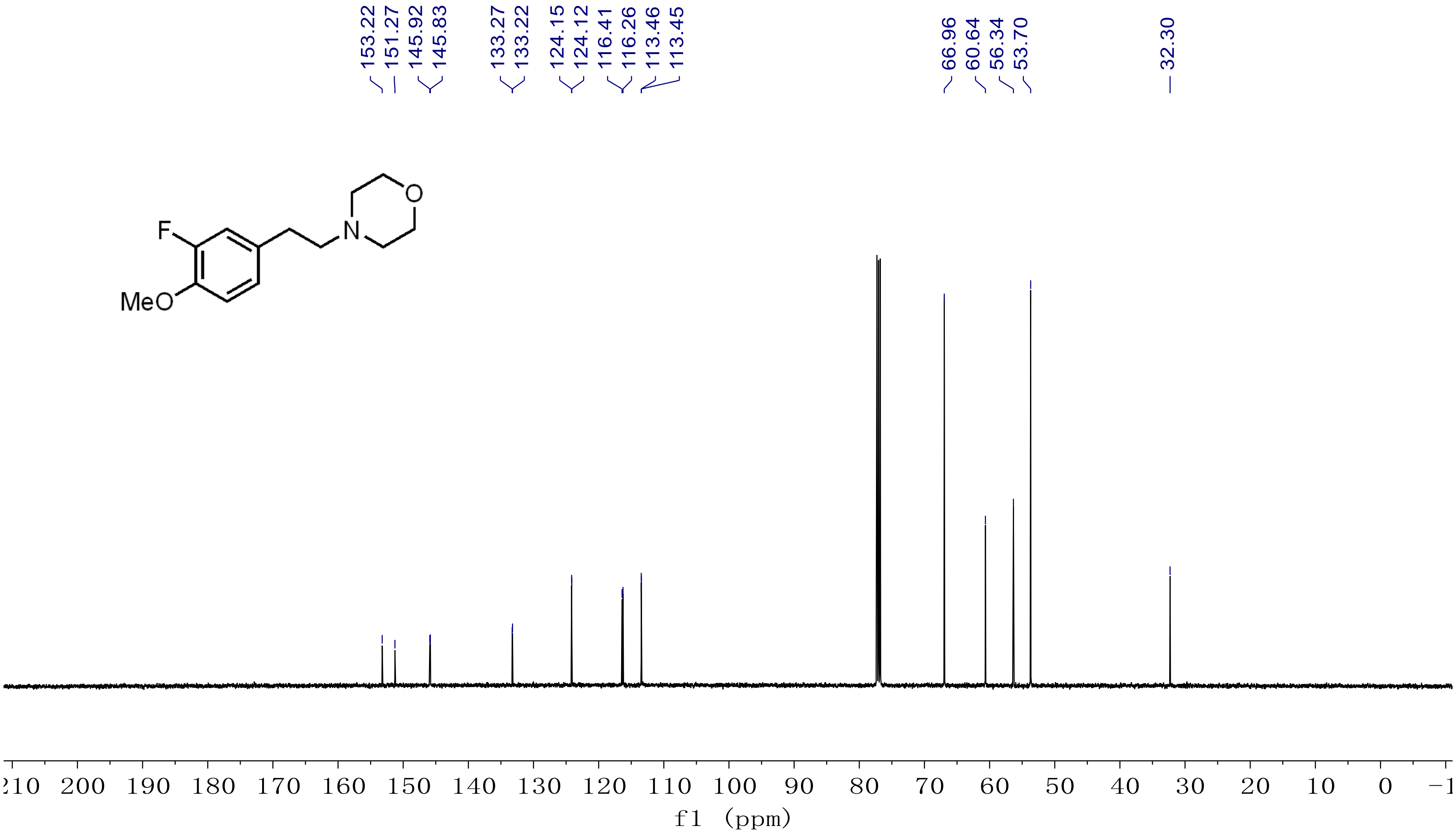

^{19}F NMR of arylethylamine 3

$CDCl_3$, 23 ºC

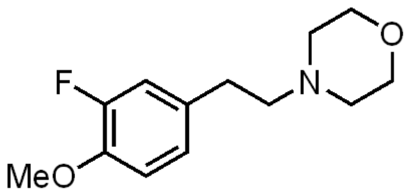

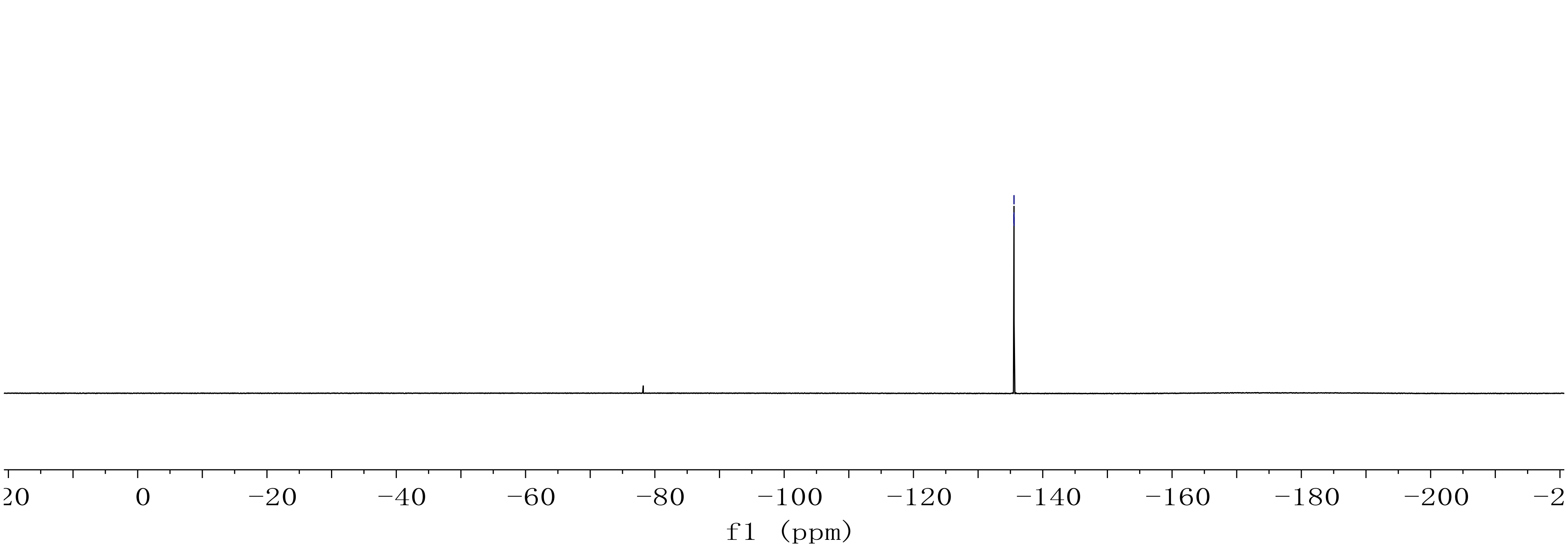

^{1}H NMR of arylethylamine 4

CDCl$_3$, 23 ºC

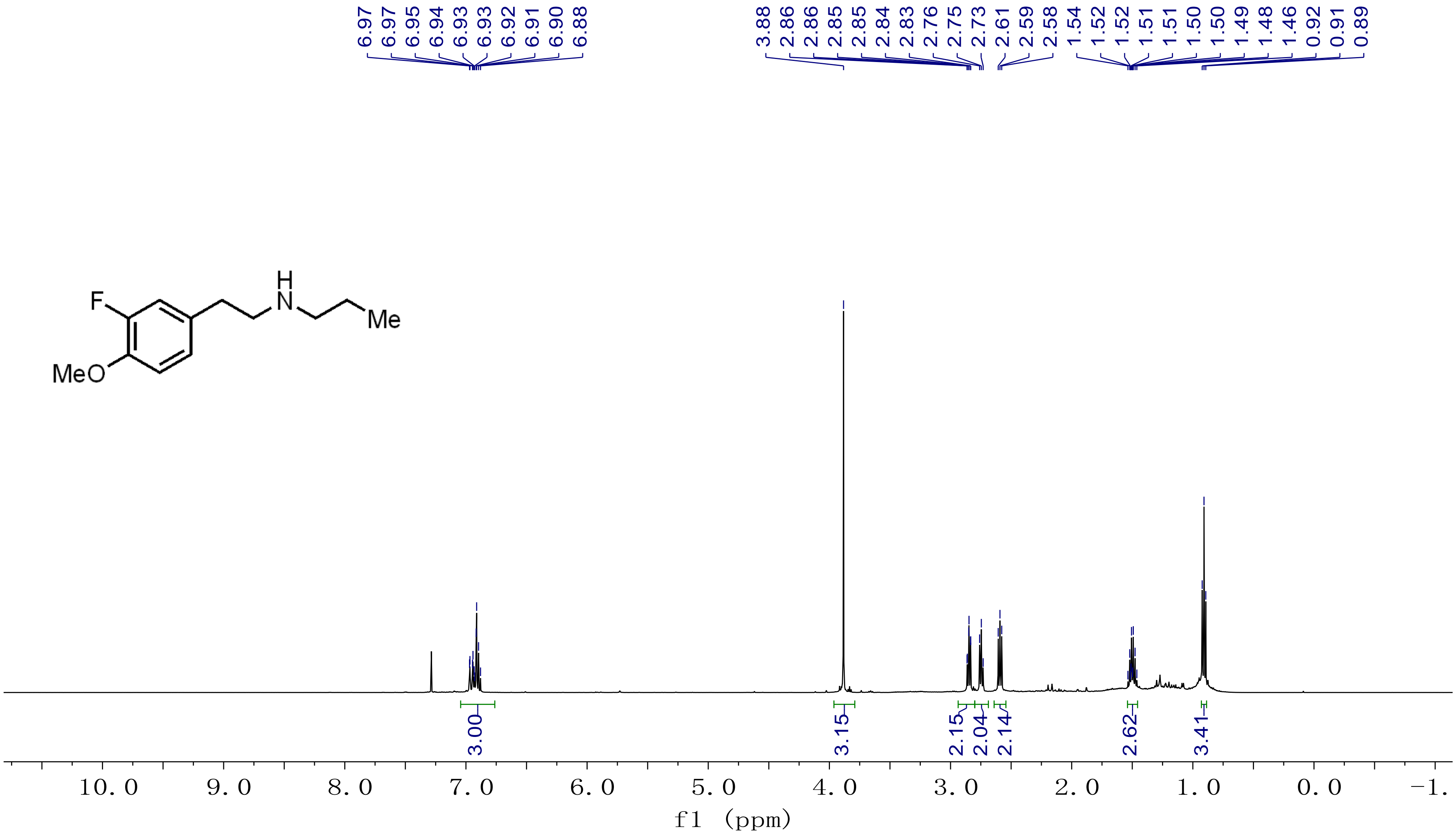

^{13}C NMR of arylethylamine 4

$CDCl_3$, 23 ºC

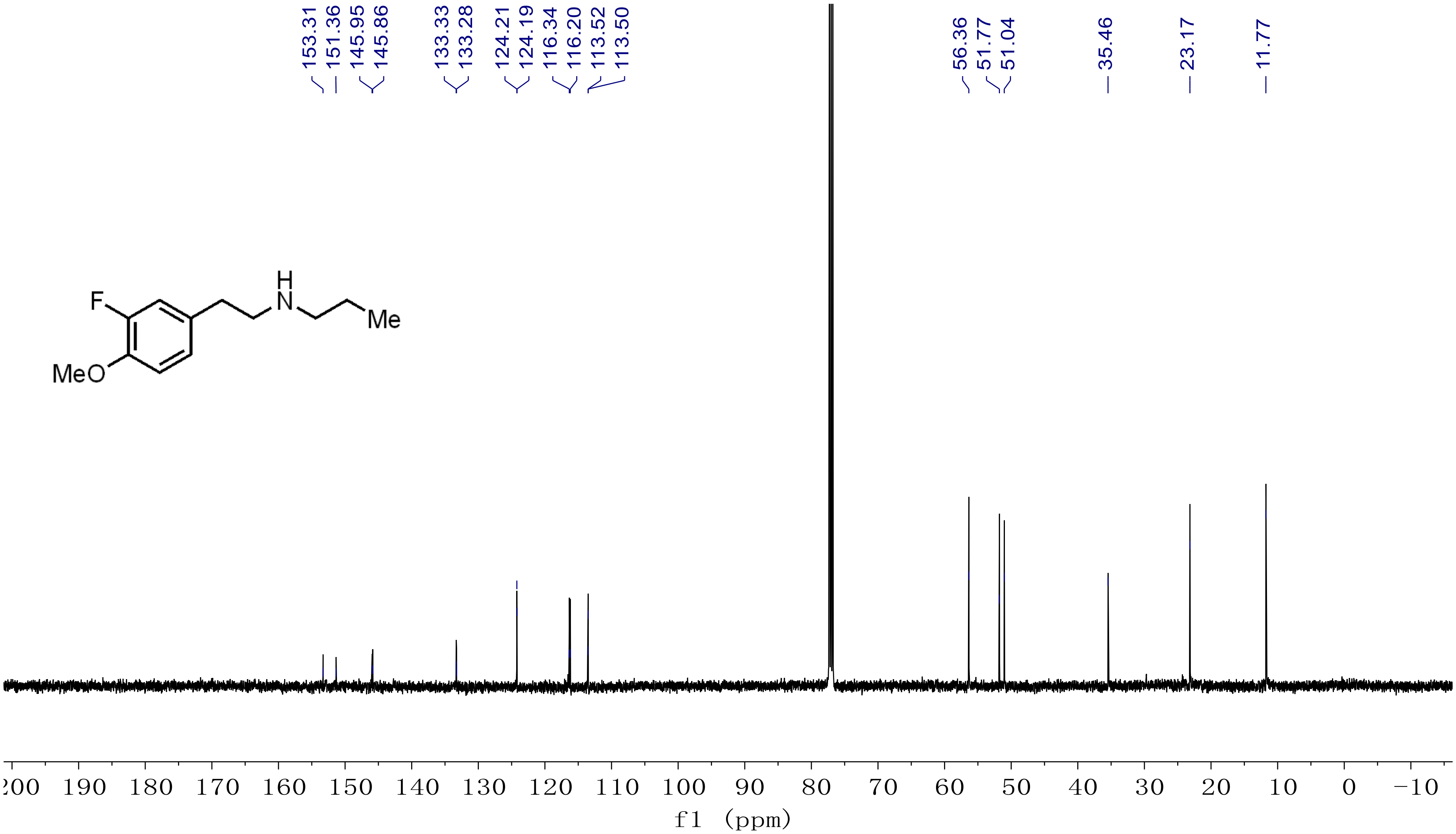

^{19}F NMR of arylethylamine 4

$CDCl_3$, 23 ºC

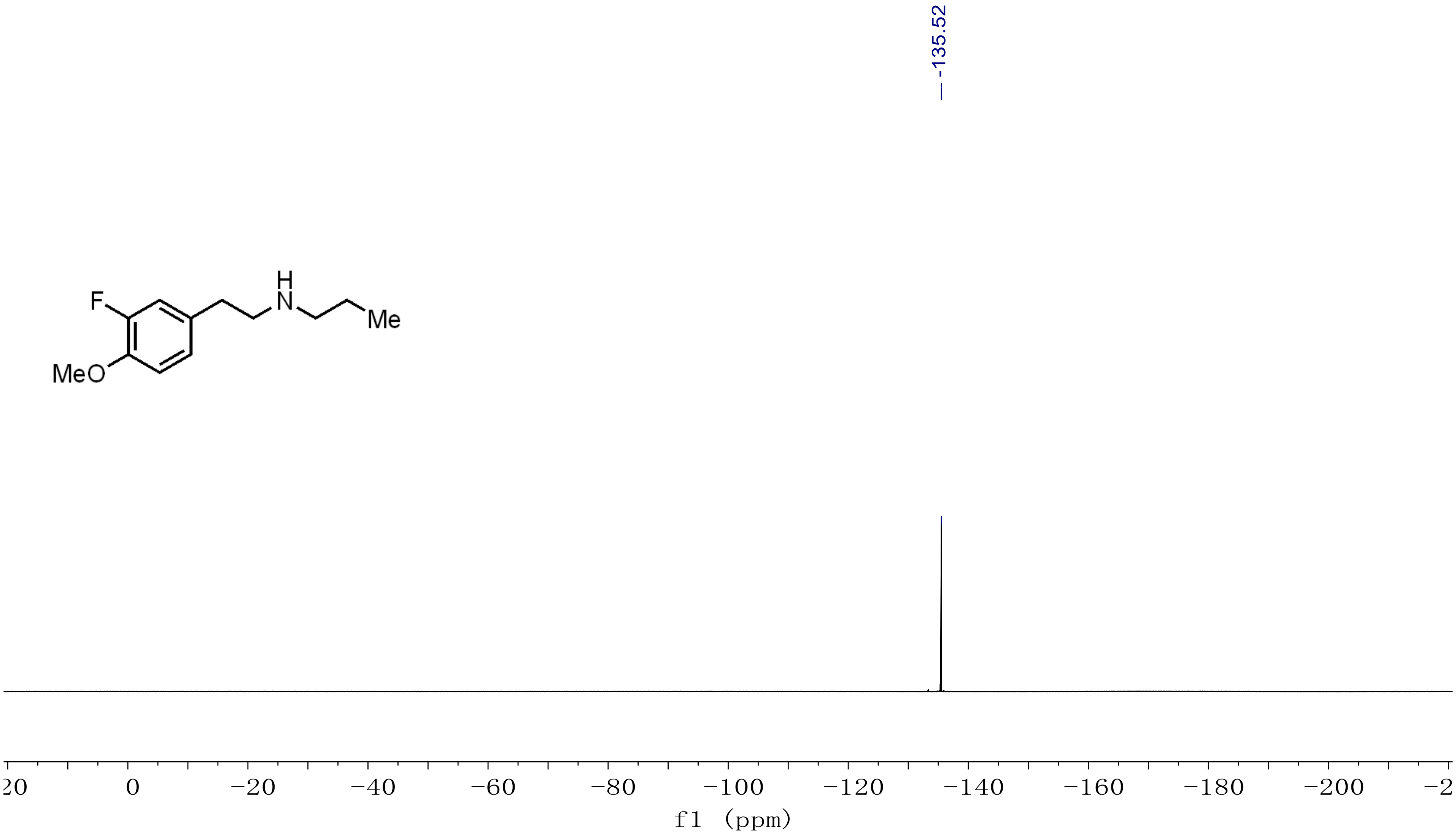

^{1}H NMR of arylethylamine 5

$CDCl_3$, 23 ºC

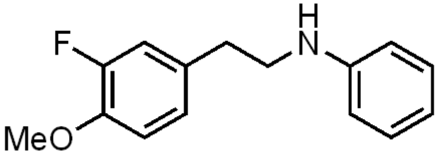

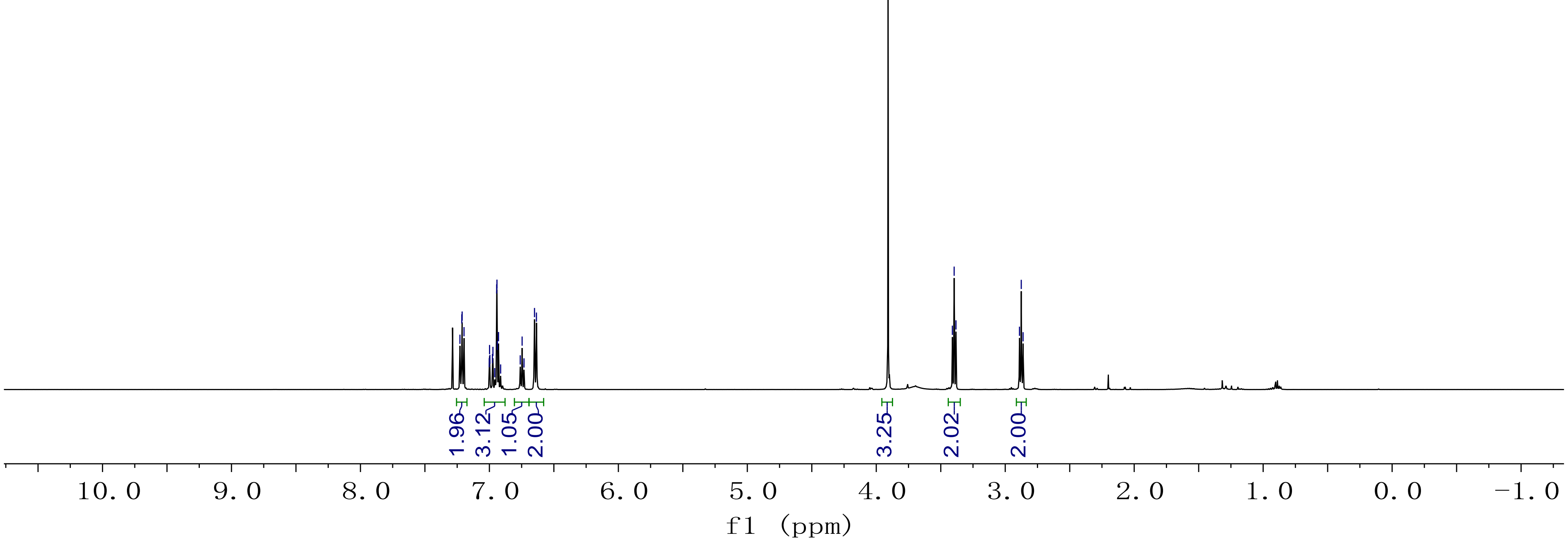

^{13}C NMR of arylethylamine 5

CDCl$_3$, 23 ºC

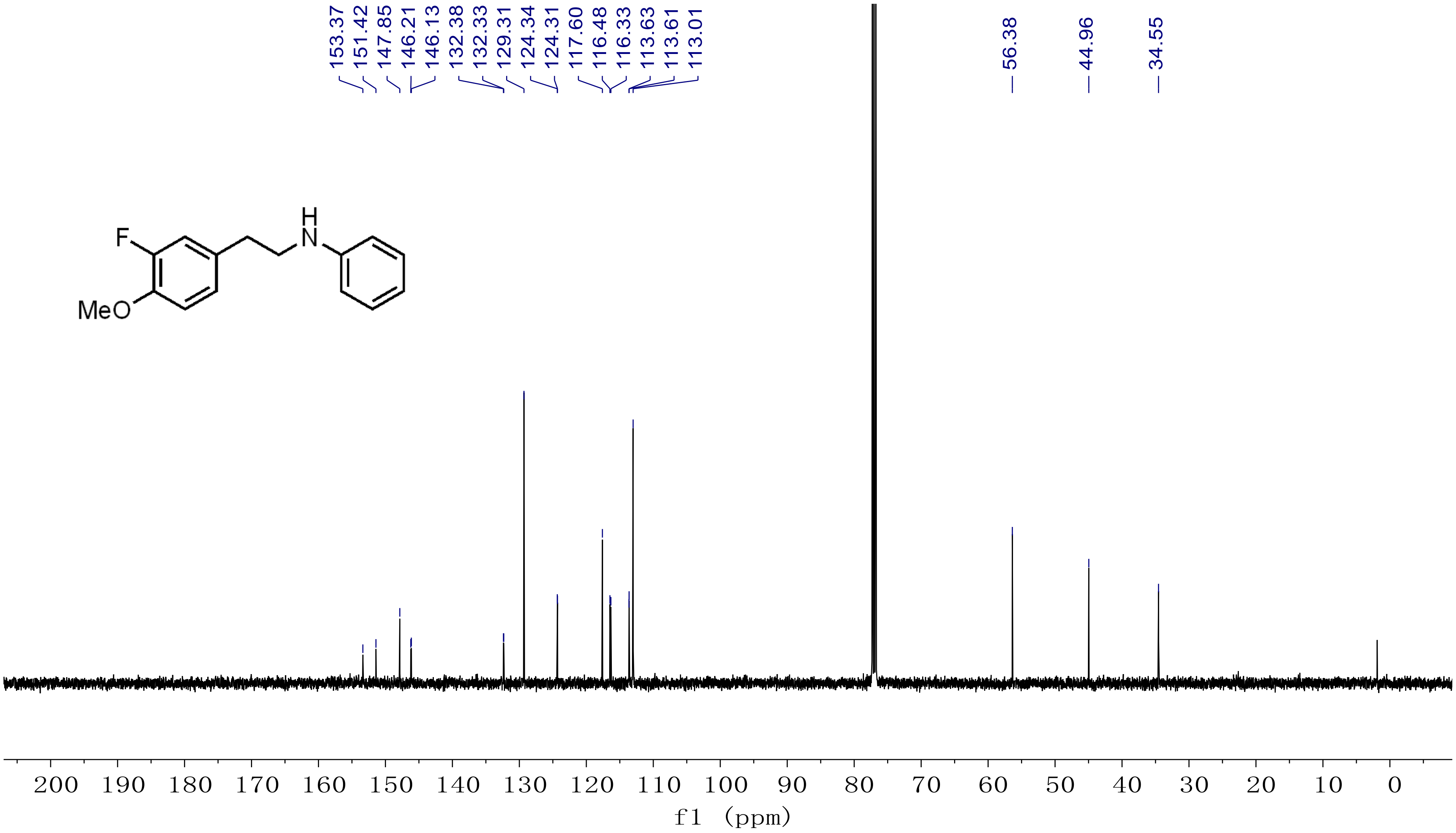

^{19}F NMR of arylethylamine 5

$CDCl_3$, 23 ºC

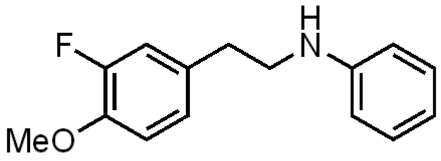

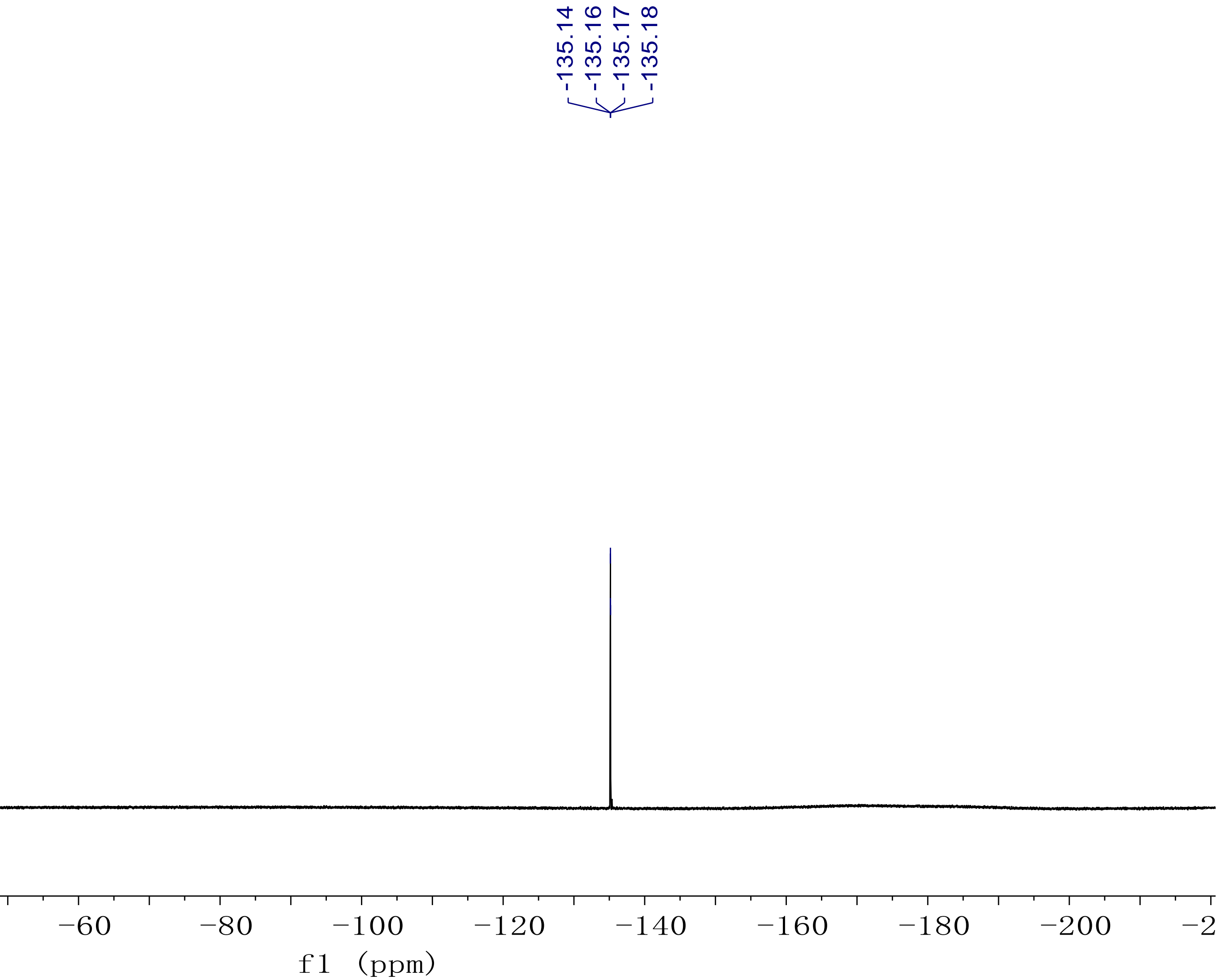

^{1}H NMR of thalidomide-derived arylethylamine 6

CDCl$_3$, 23 ºC

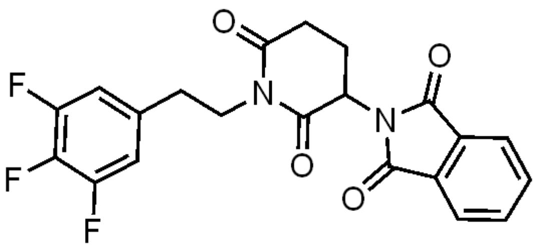

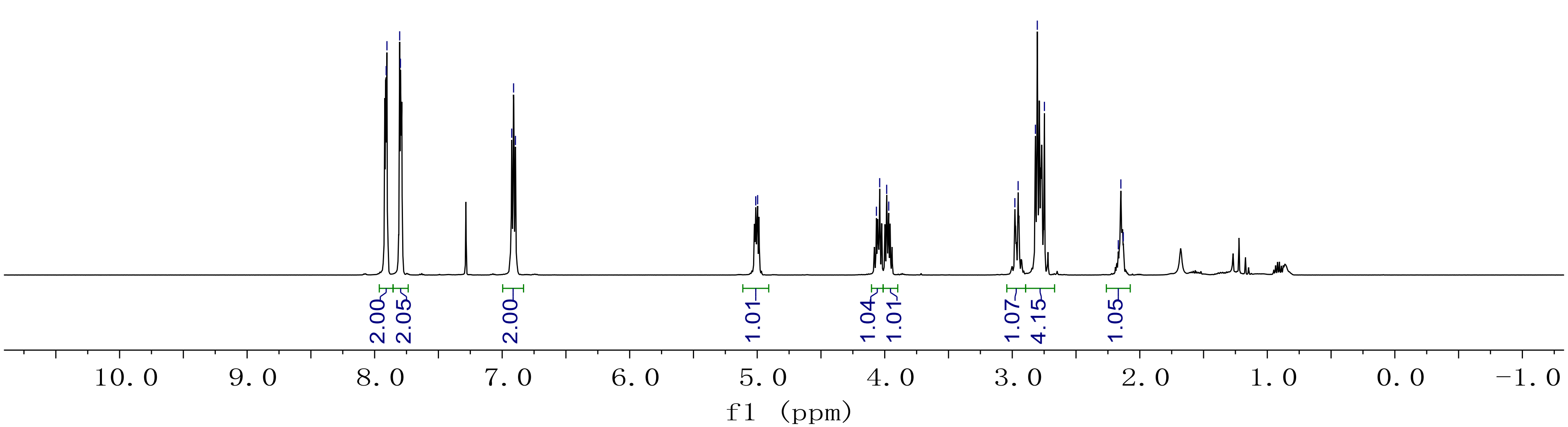

^{13}C NMR of thalidomide-derived arylethylamine 6

CDCl$_3$, 23 ºC

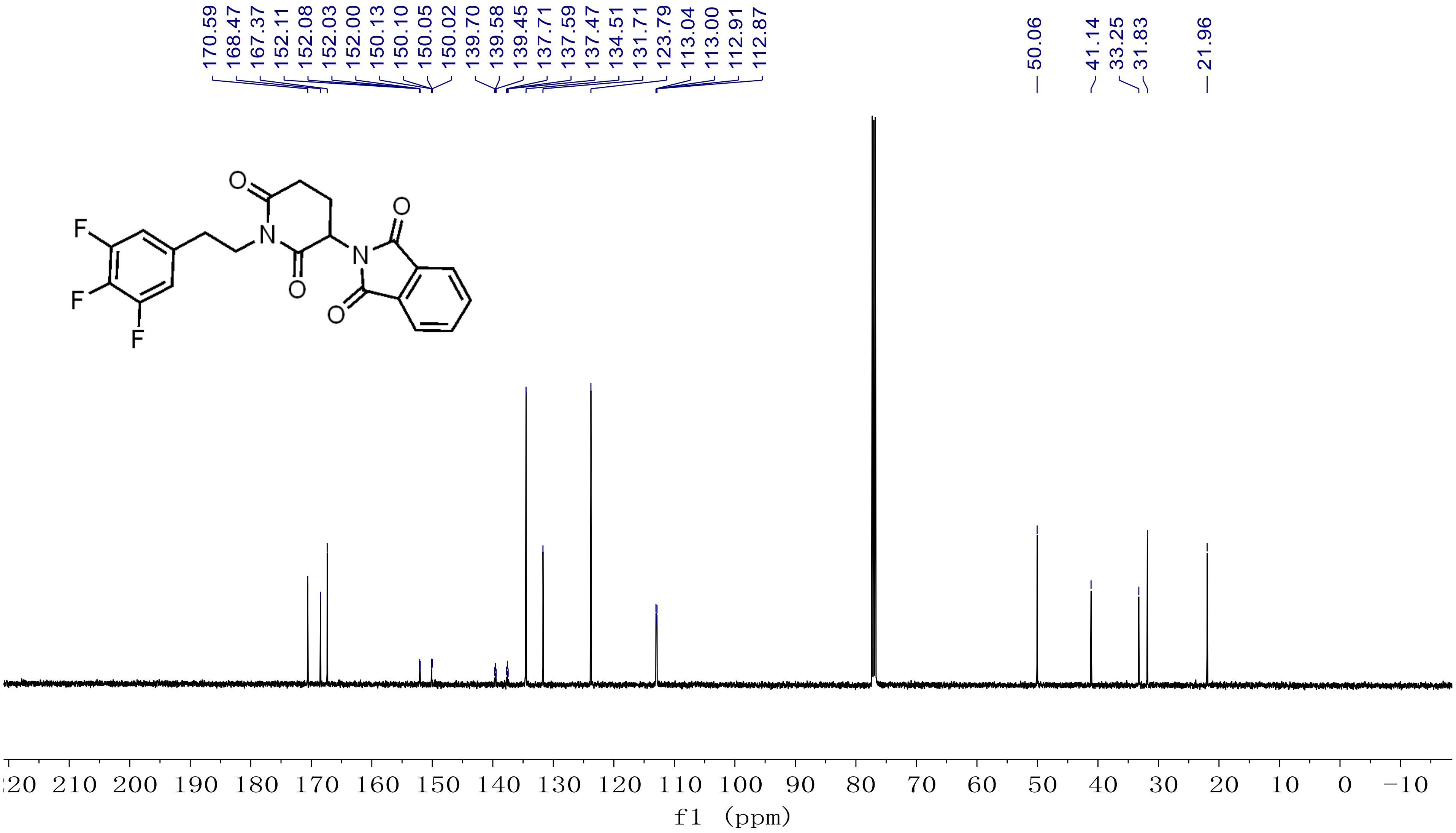

^{19}F NMR of thalidomide-derived arylethylamine 6

$CDCl_3$, 23 ºC

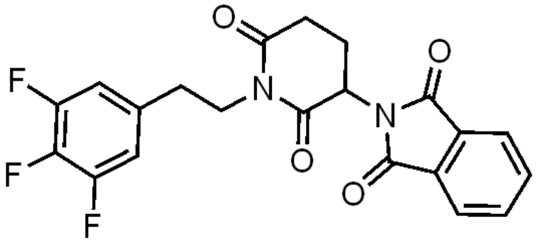

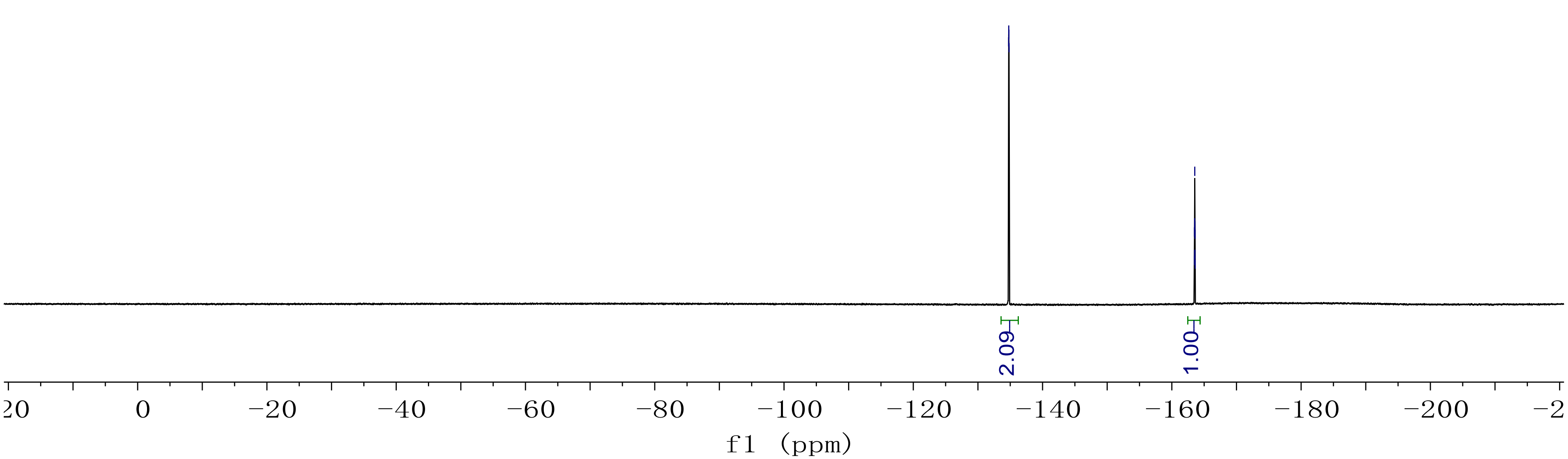

^{1}H NMR of fenofibrate-derived arylethyl chloride 7

$CDCl_3$, 23 ºC

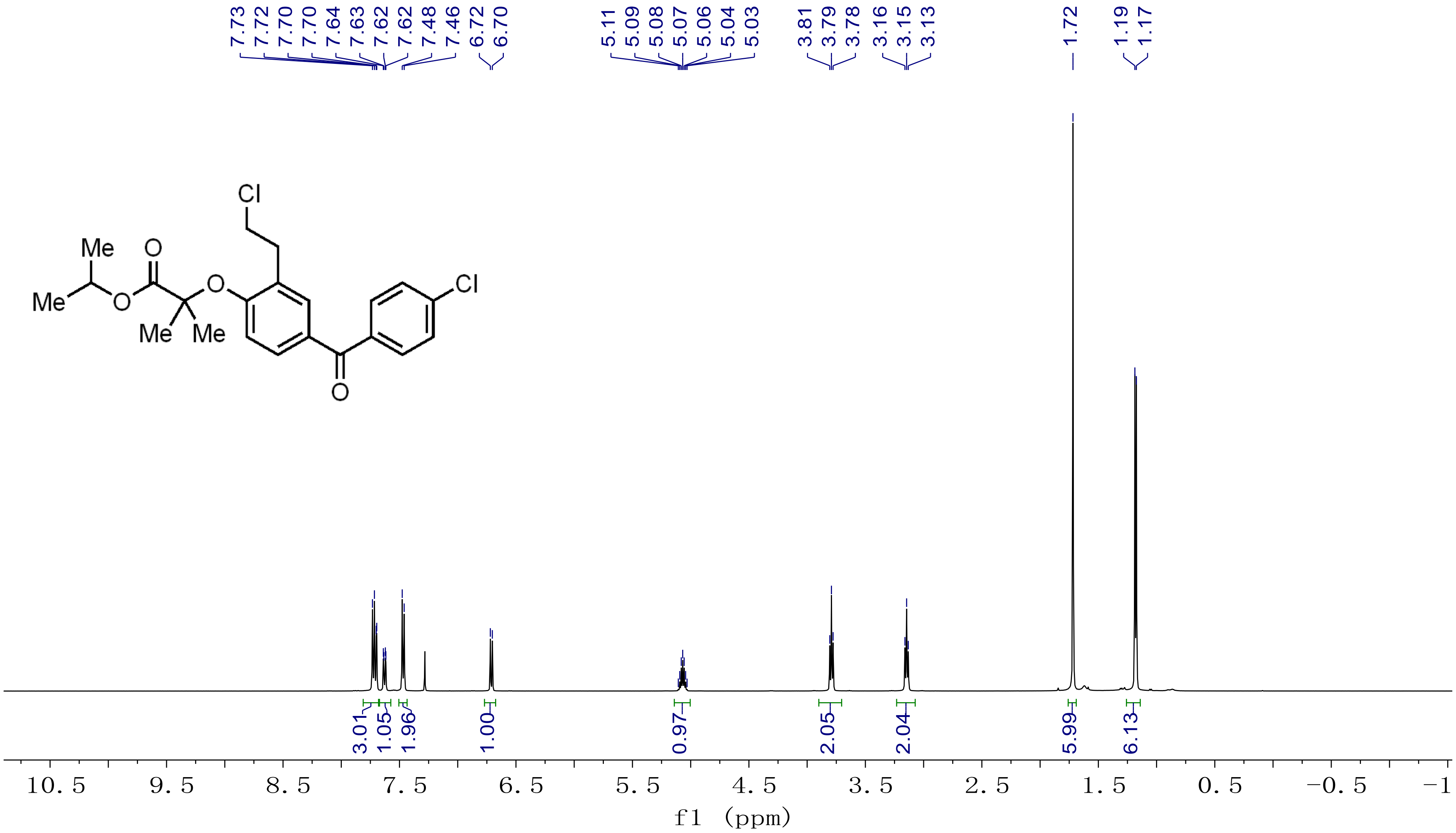

^{13}C NMR of fenofibrate-derived arylethyl chloride 7

$CDCl_3$, 23 ºC

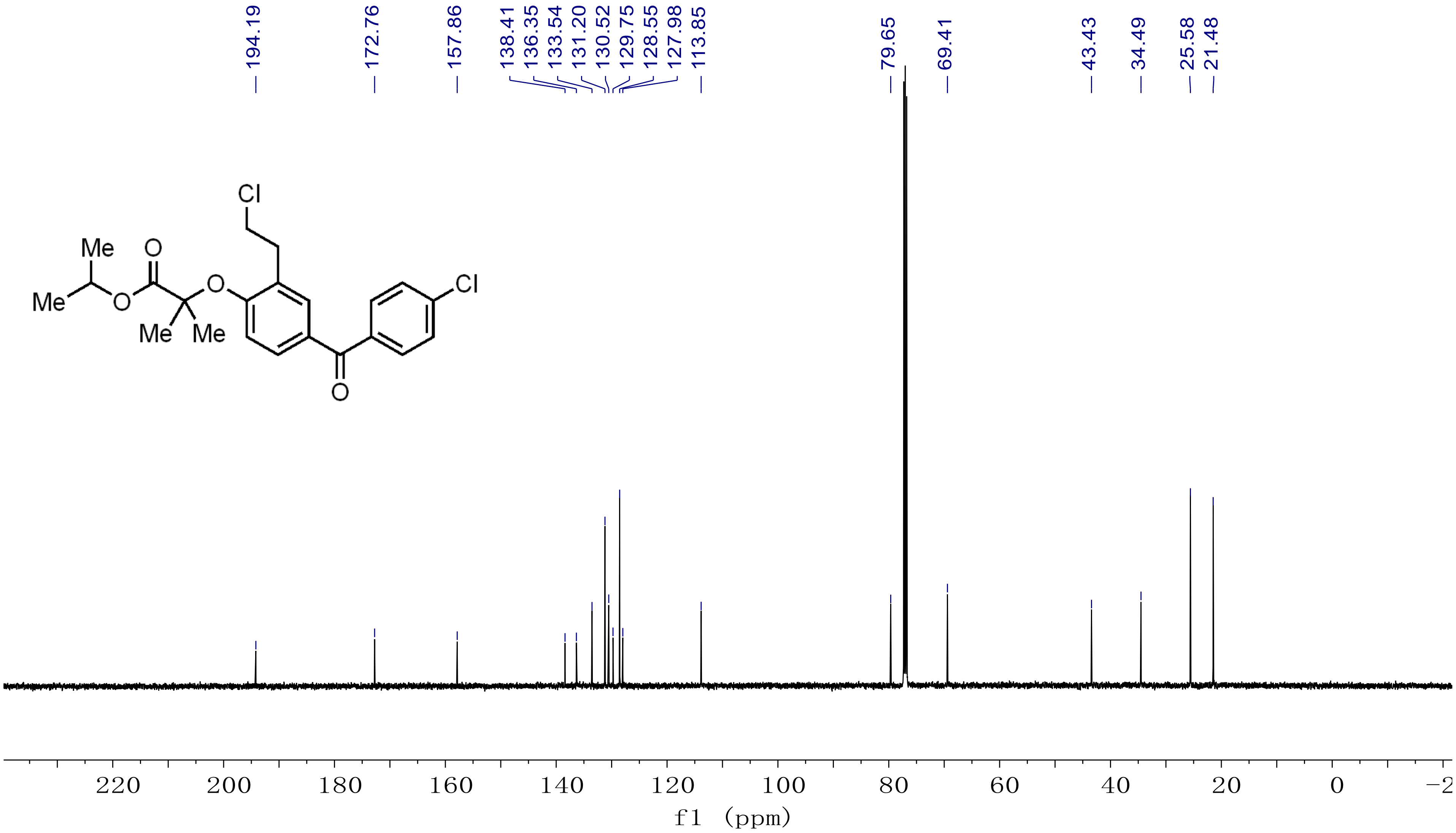

^{1}H NMR of vinyl arene 8

$CDCl_3$, 23 ºC

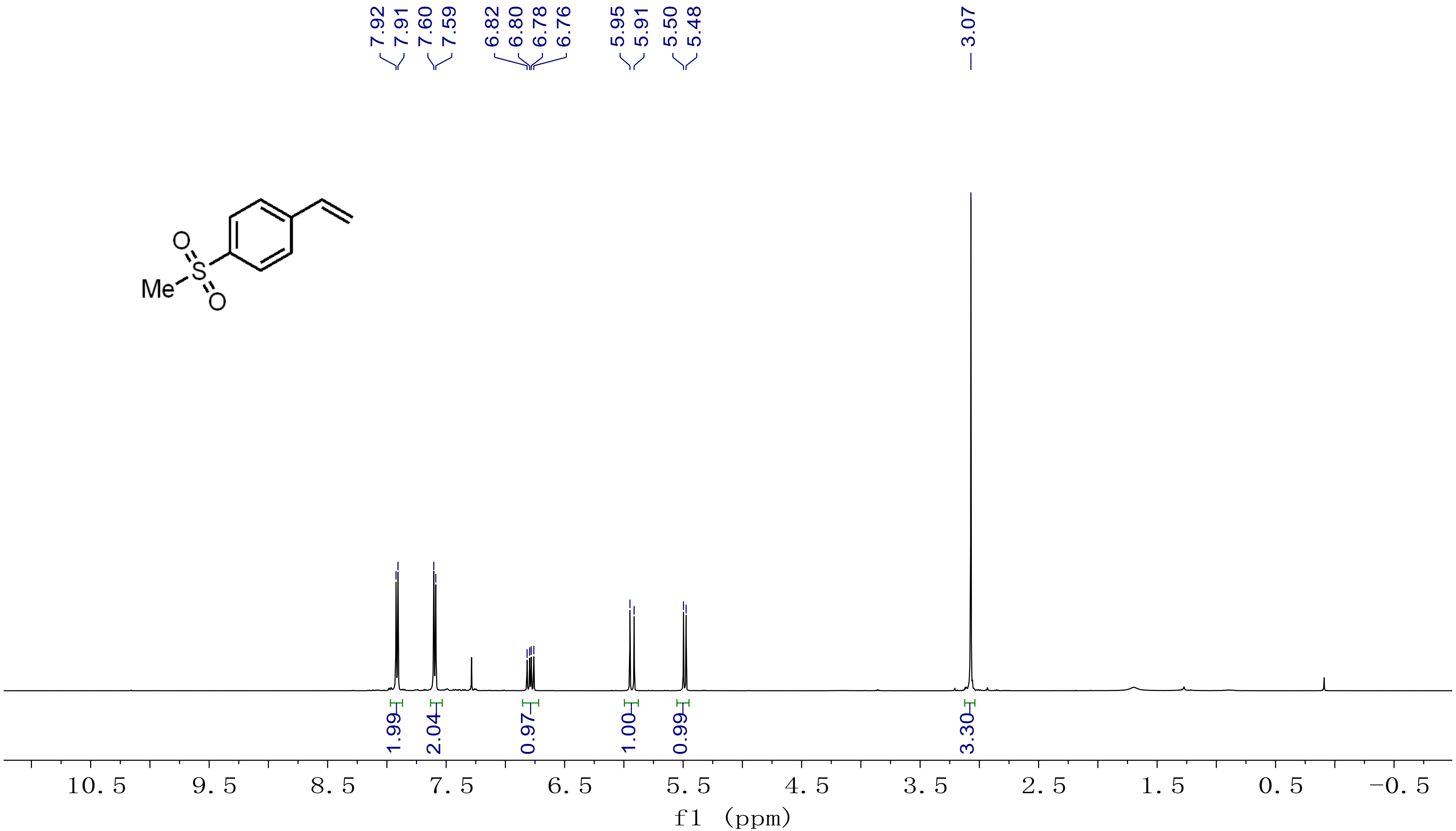

^{13}C NMR of vinyl arene 8

$CDCl_3$, 23 ºC

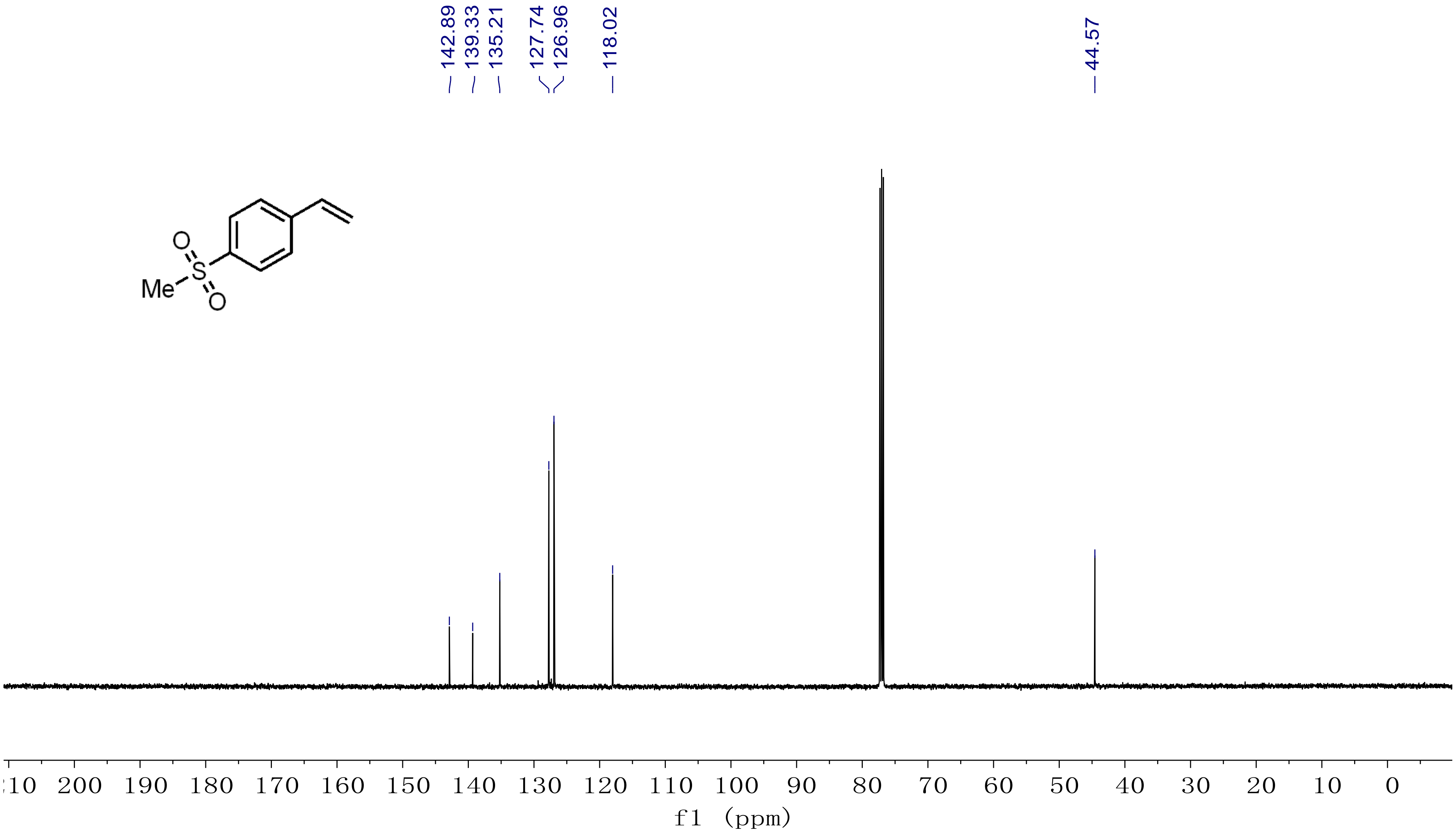

^{1}H NMR of arylethyl thioether 9

CDCl$_3$, 23 ºC

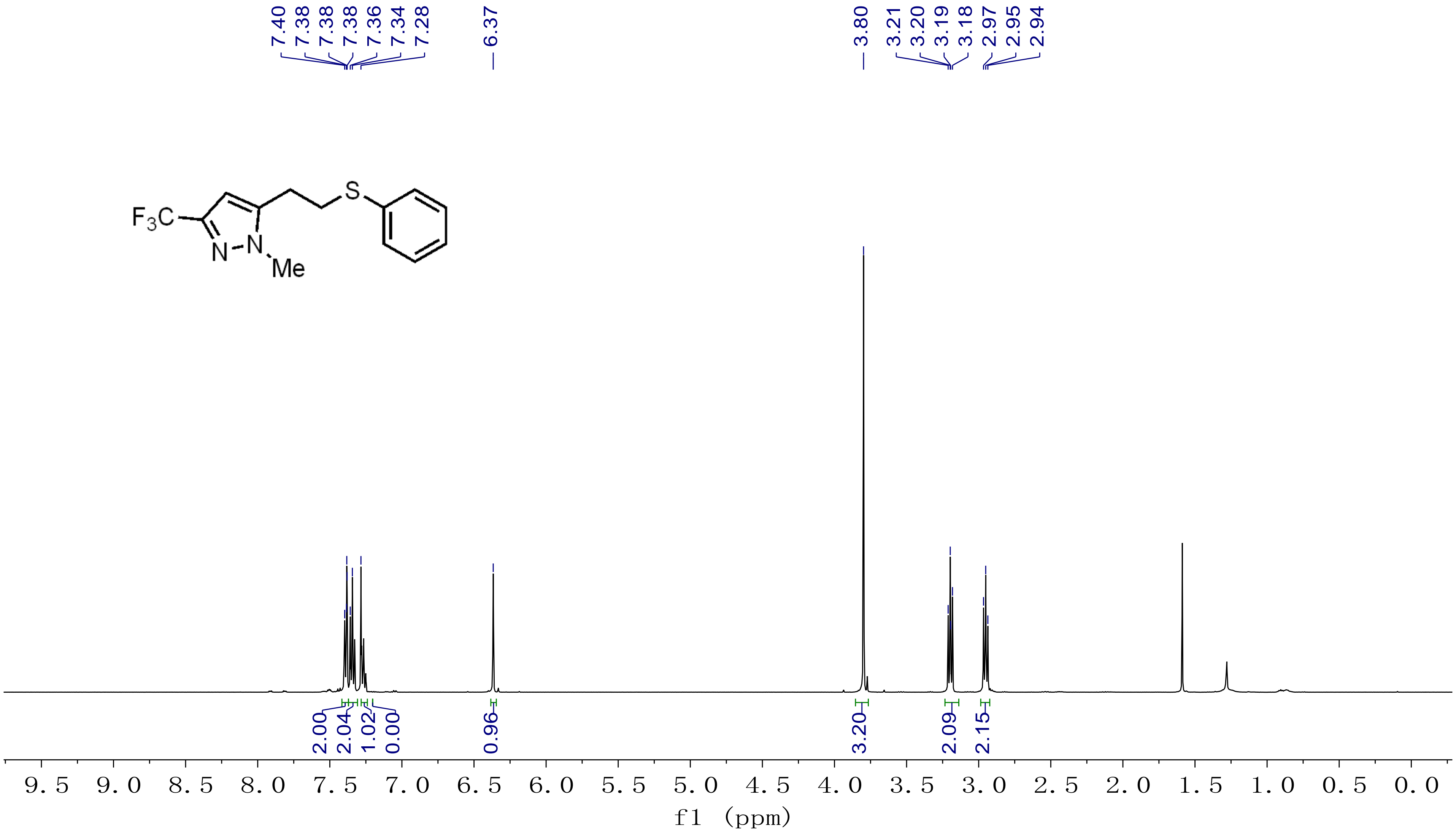

^{13}C NMR of arylethyl thioether 9

$CDCl_3$, 23 ºC

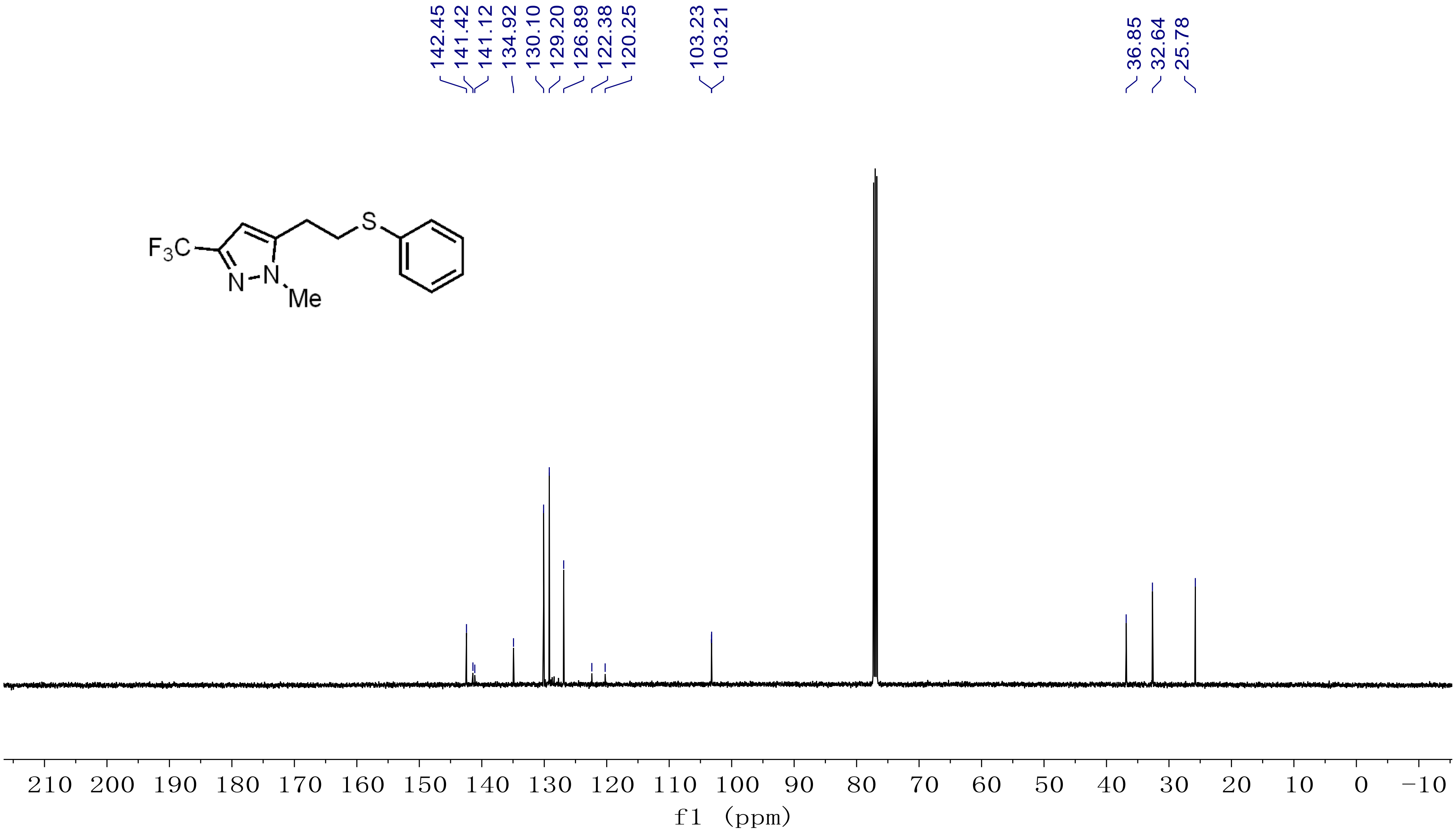

^{19}F NMR of arylethyl thioether 9

$CDCl_3$, 23 ºC

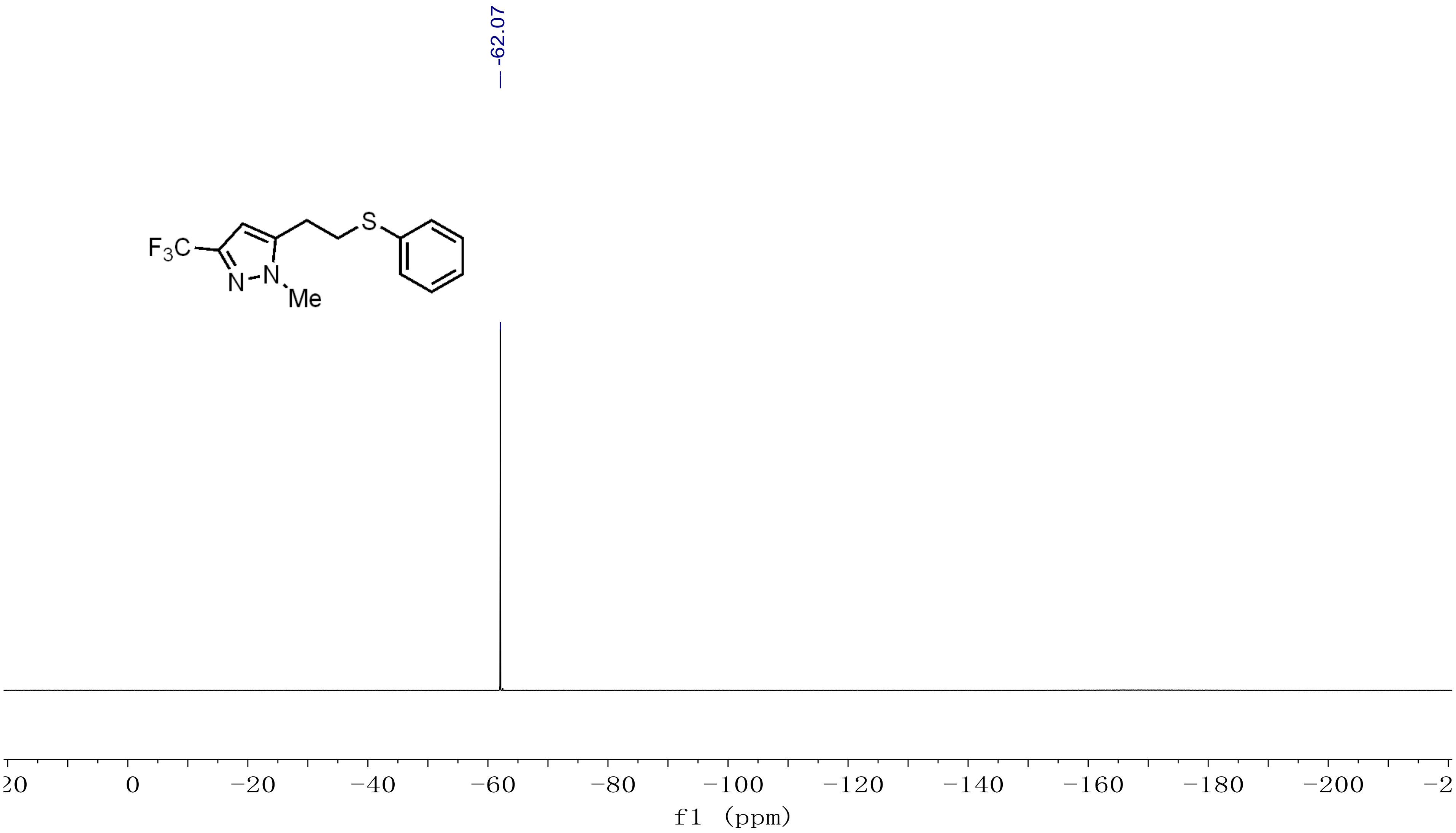

^{1}H NMR of arylethyl selenoether 10

$CDCl_3$, 23 ºC

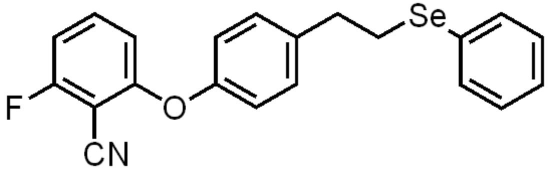

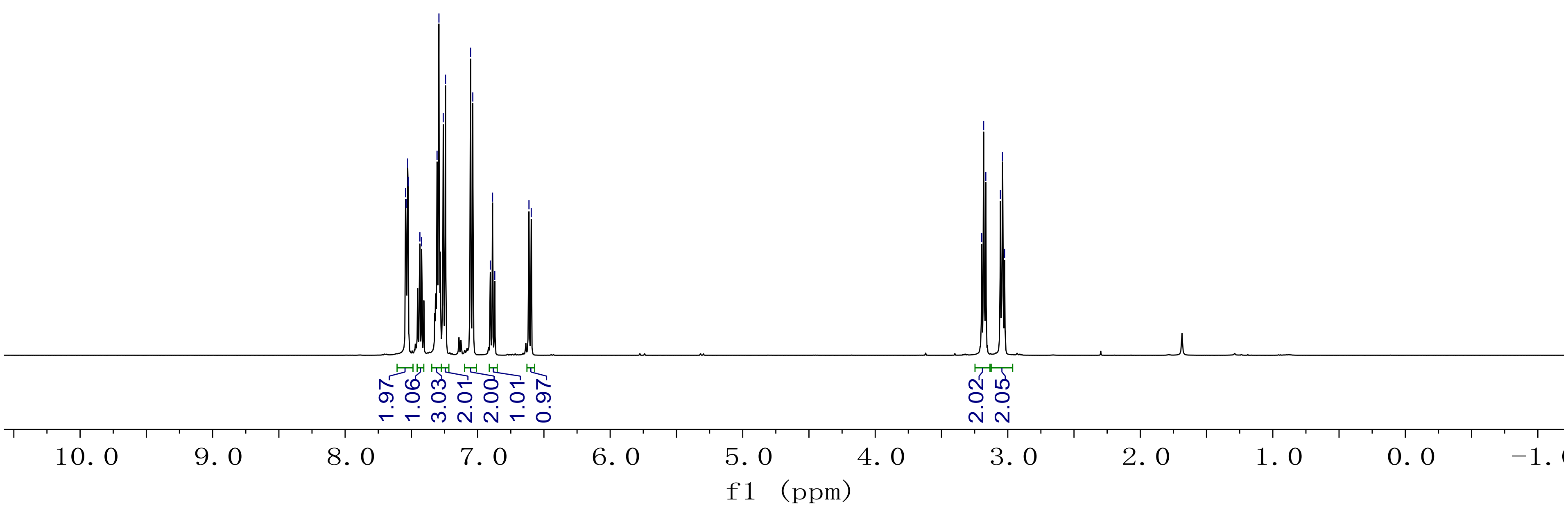

^{13}C NMR of arylethyl selenoether 10

$CDCl_3$, 23 ºC

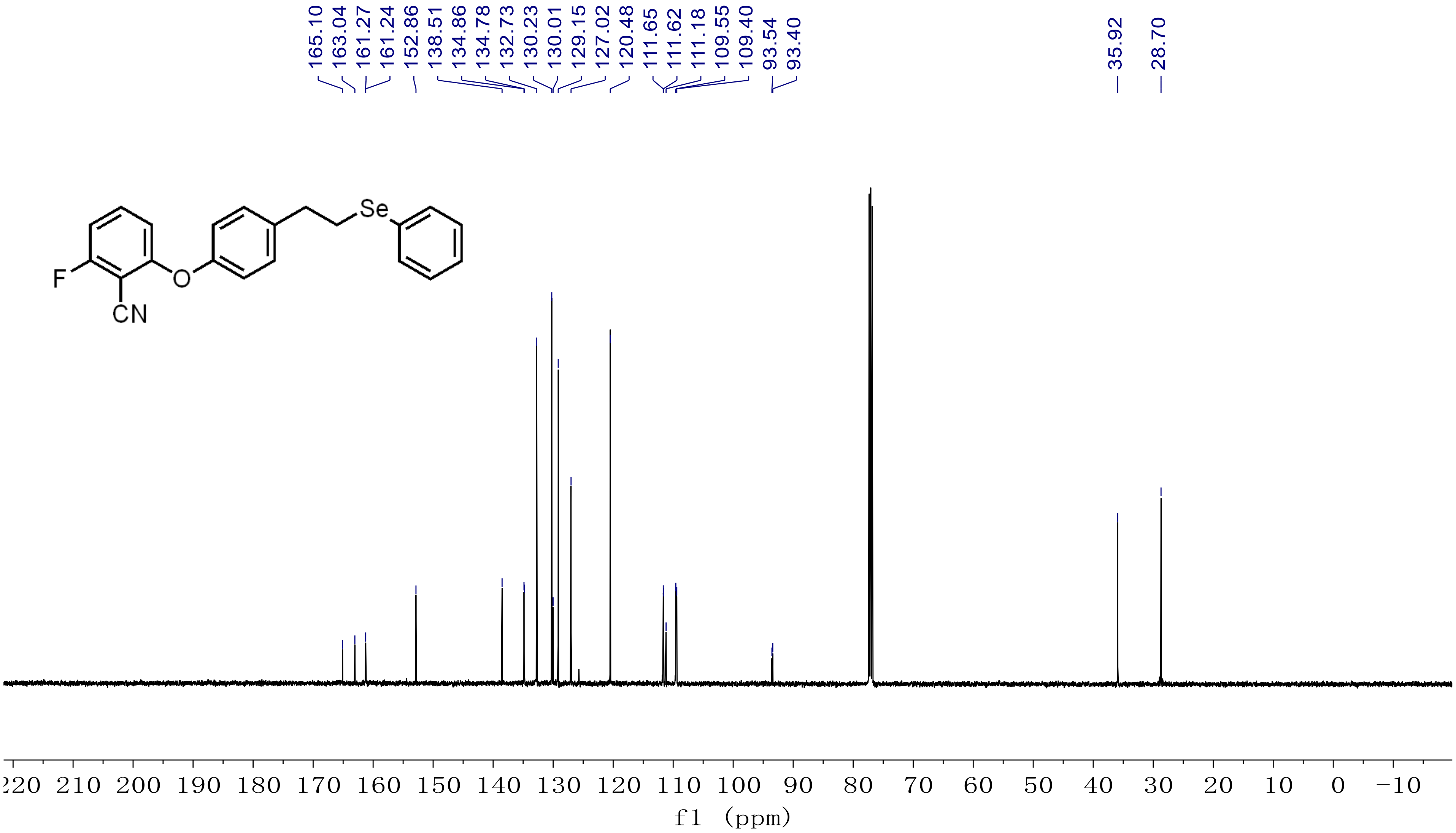

^{19}F NMR of arylethyl selenoether 10

$CDCl_3$, 23 ºC

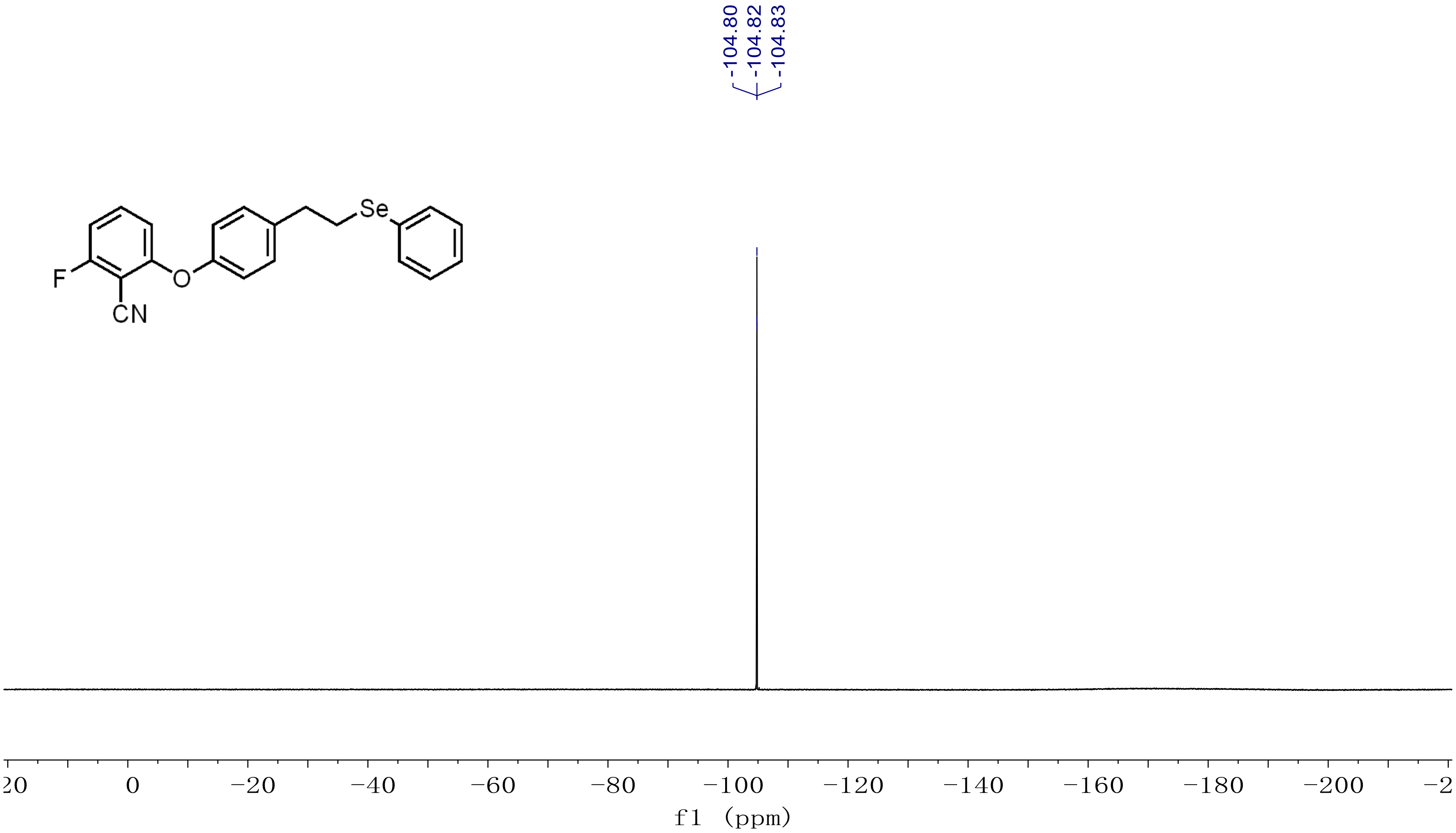

^{1}H NMR of trimetazidine-derived arylethylamine 11

$CDCl_3$, 23 ºC

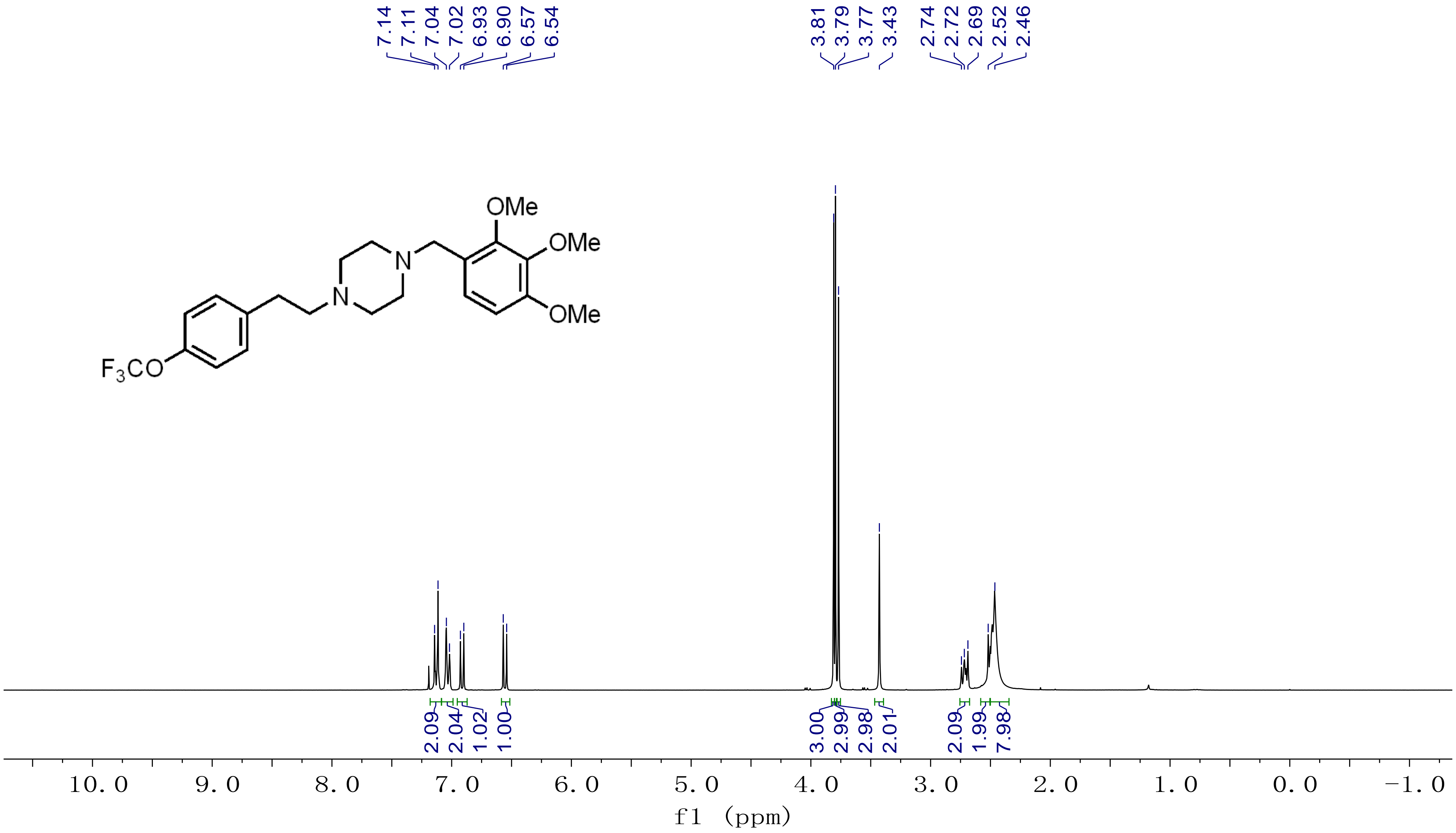

^{13}C NMR of trimetazidine-derived arylethylamine 11

$CDCl_3$, 23 ºC

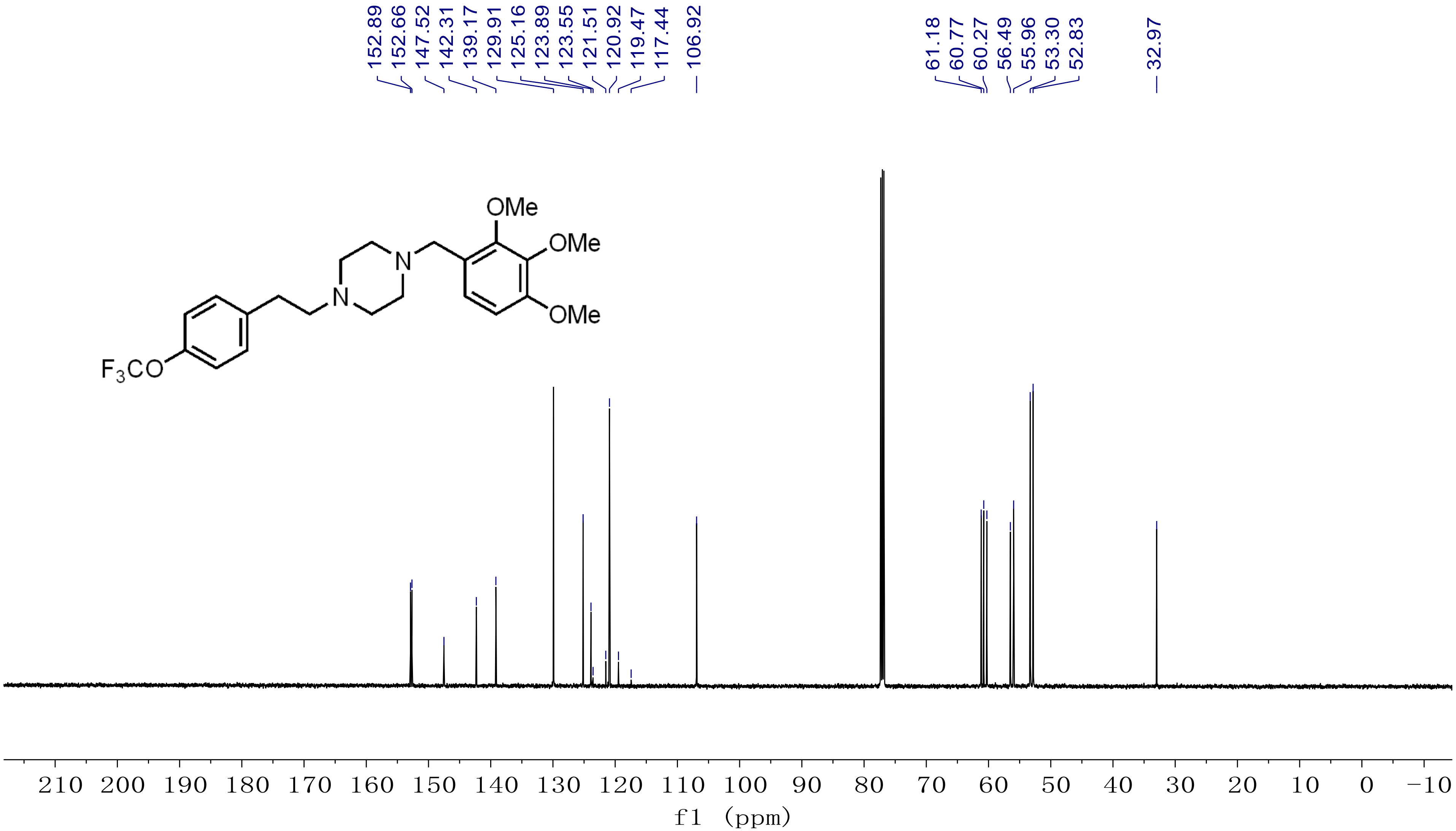

^{19}F NMR of trimetazidine-derived arylethylamine 11

CDCl3, 23 ºC

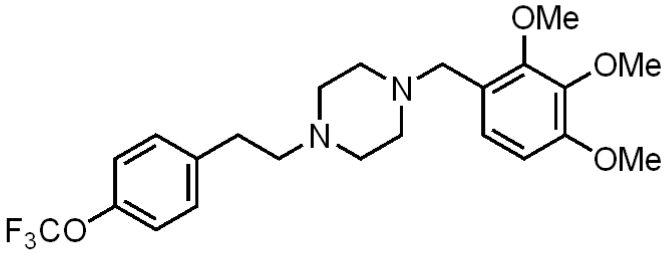

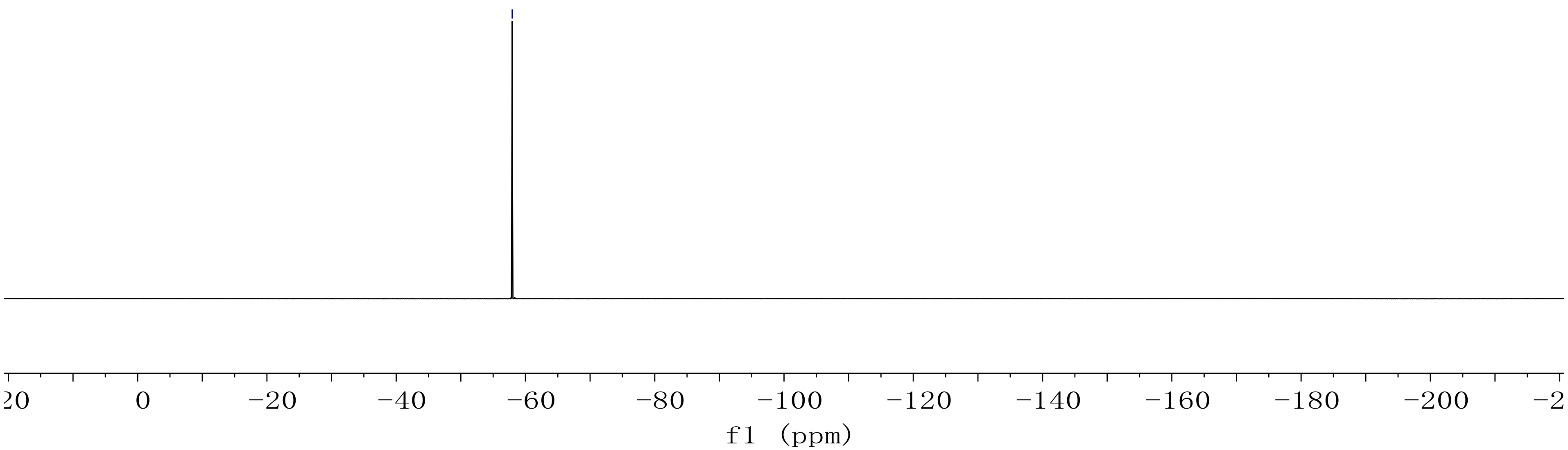

^{1}H NMR of arylethyl iodide 12

CDCl₃, 23 ºC

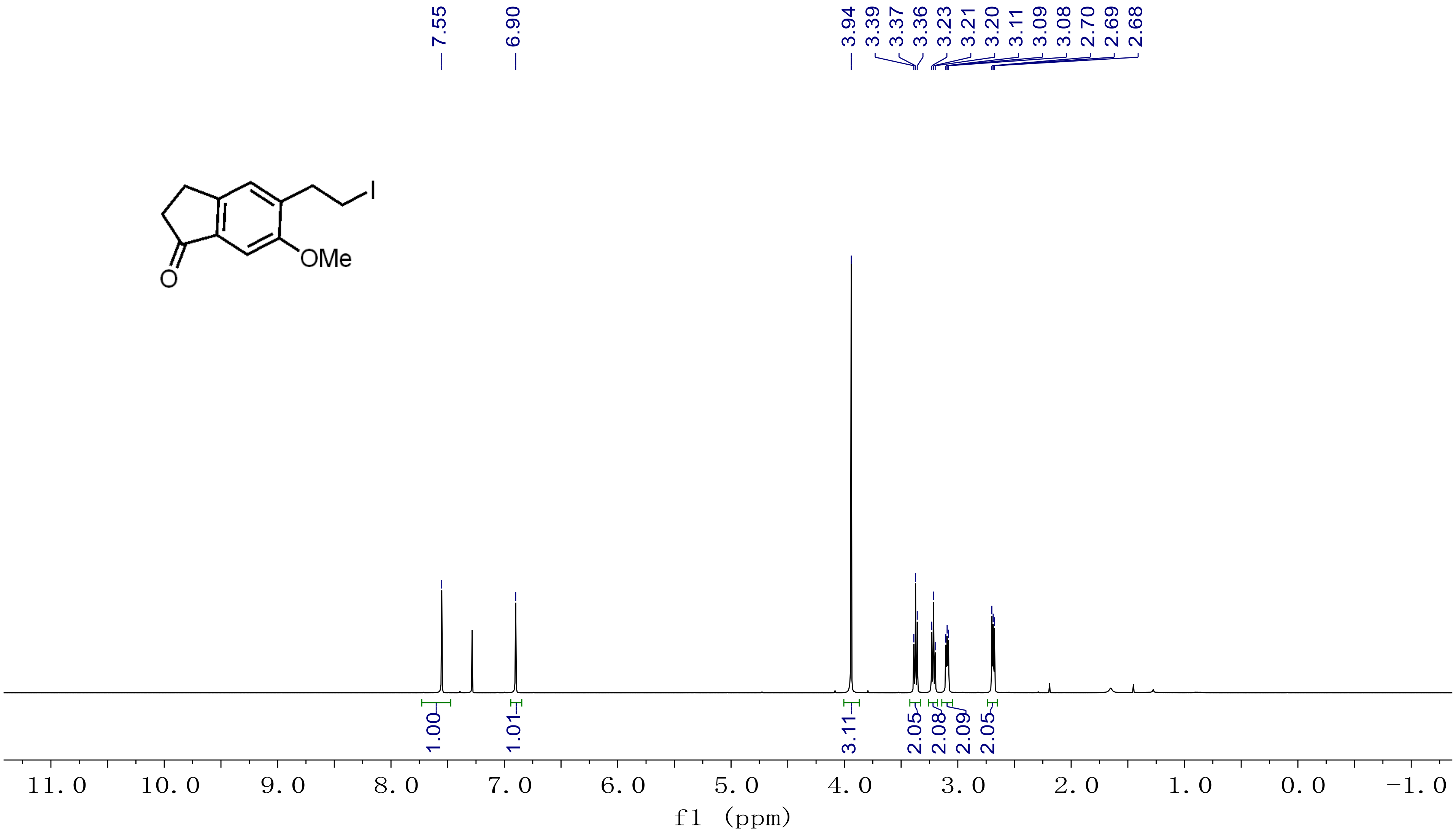

^{13}C NMR of arylethyl iodide 12

$CDCl_3$, 23 ºC

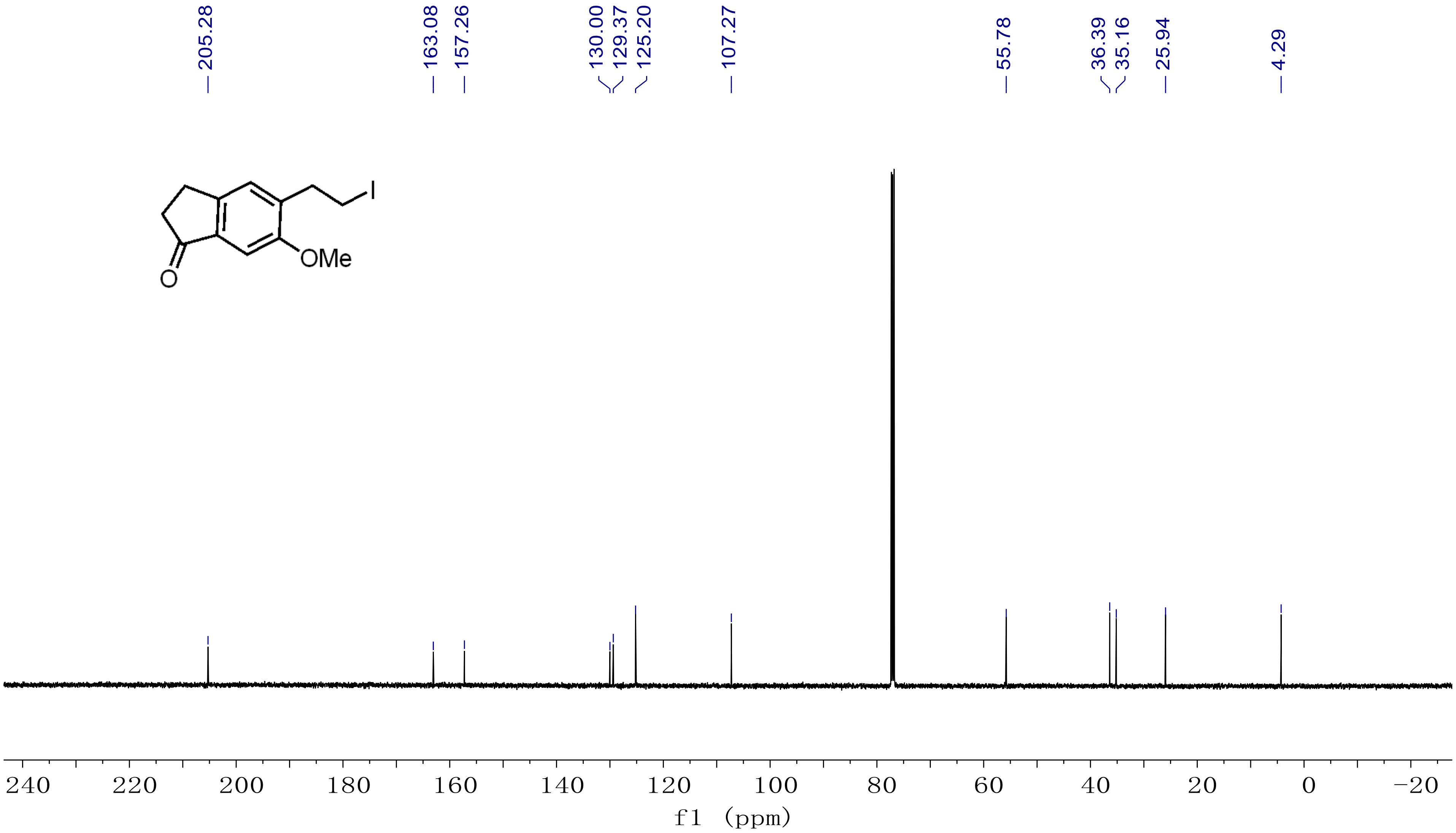

^{1}H NMR of arylethylammonium salt 13

CD_3CN, 23 ºC

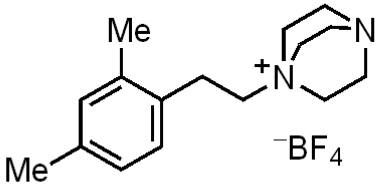

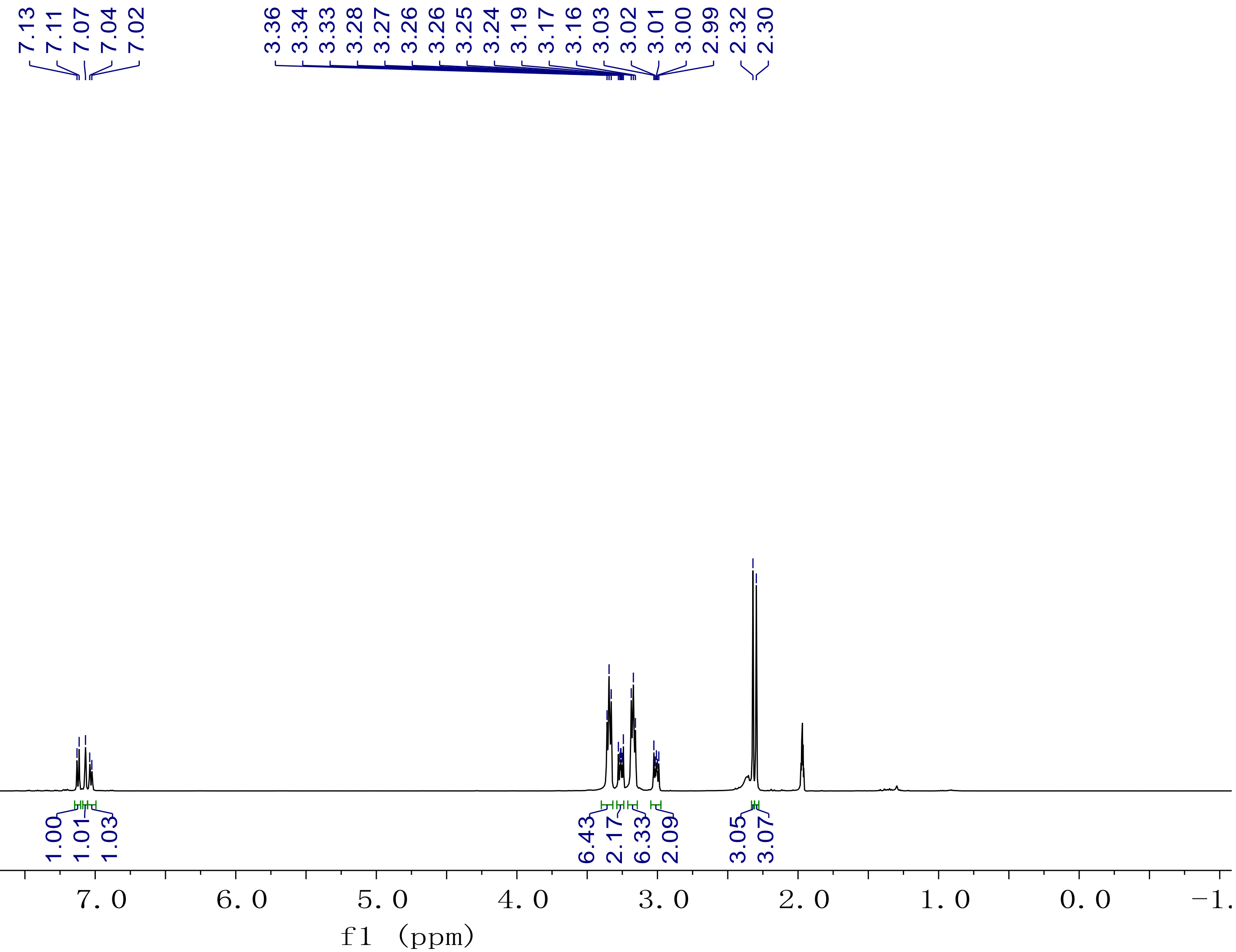

^{13}C NMR of arylethylammonium salt 13

CD_3CN, 23 ºC

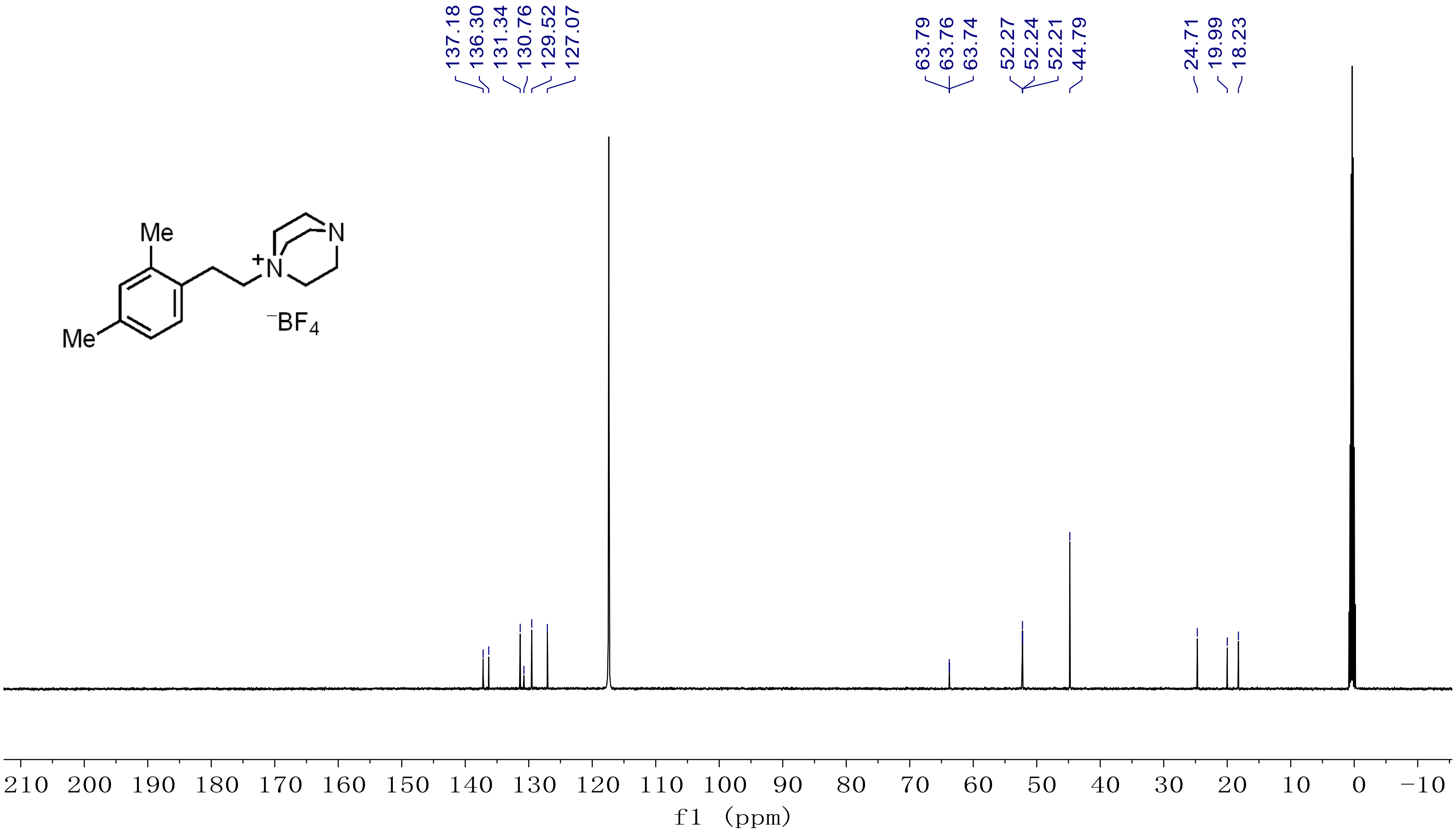

^{19}F NMR of arylethylammonium salt 13

CD_3CN, 23 ºC

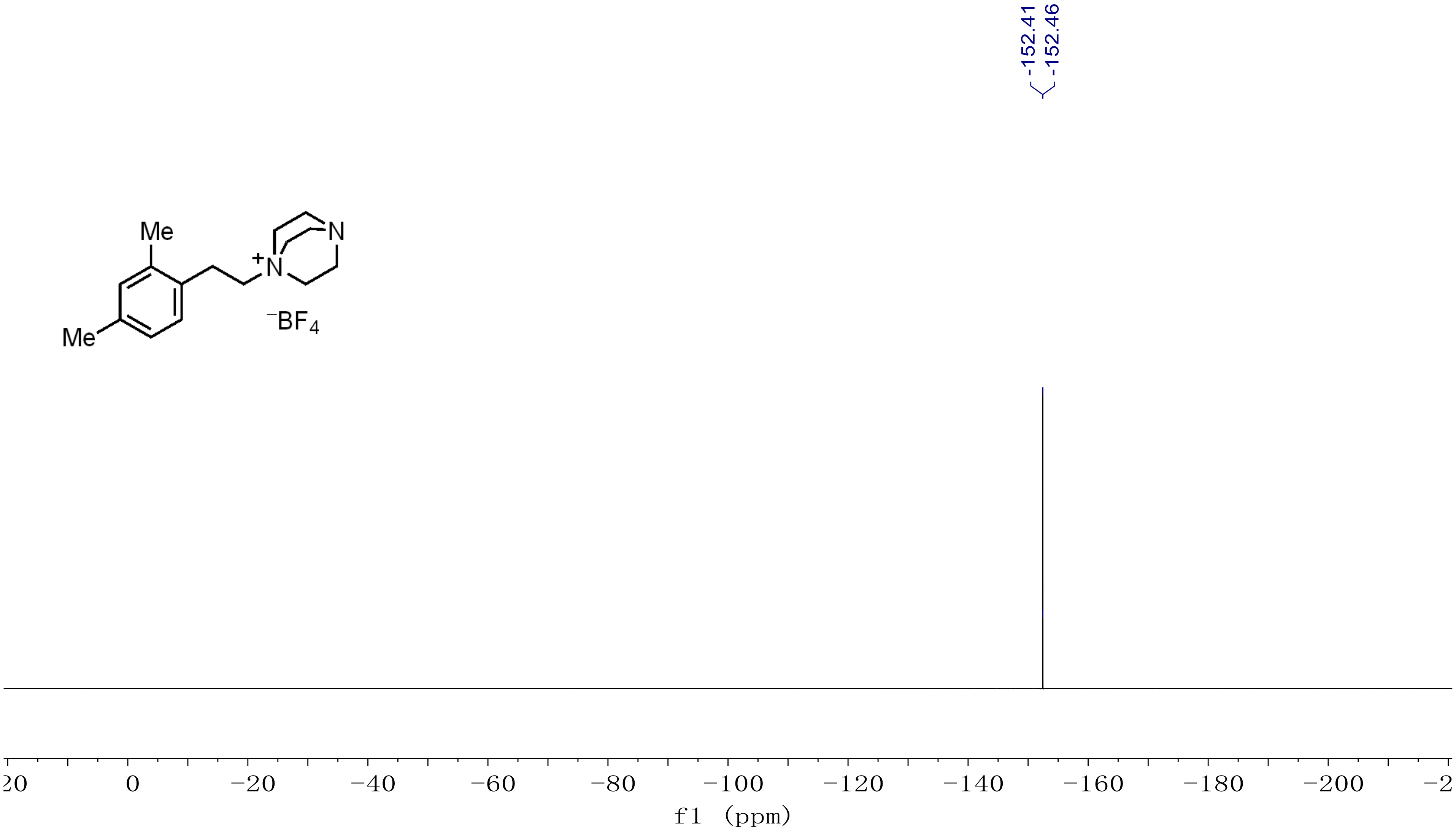

^{1}H NMR of arylethyl benzoate 14

$CDCl_3$, 23 ºC

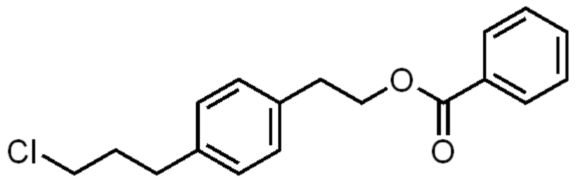

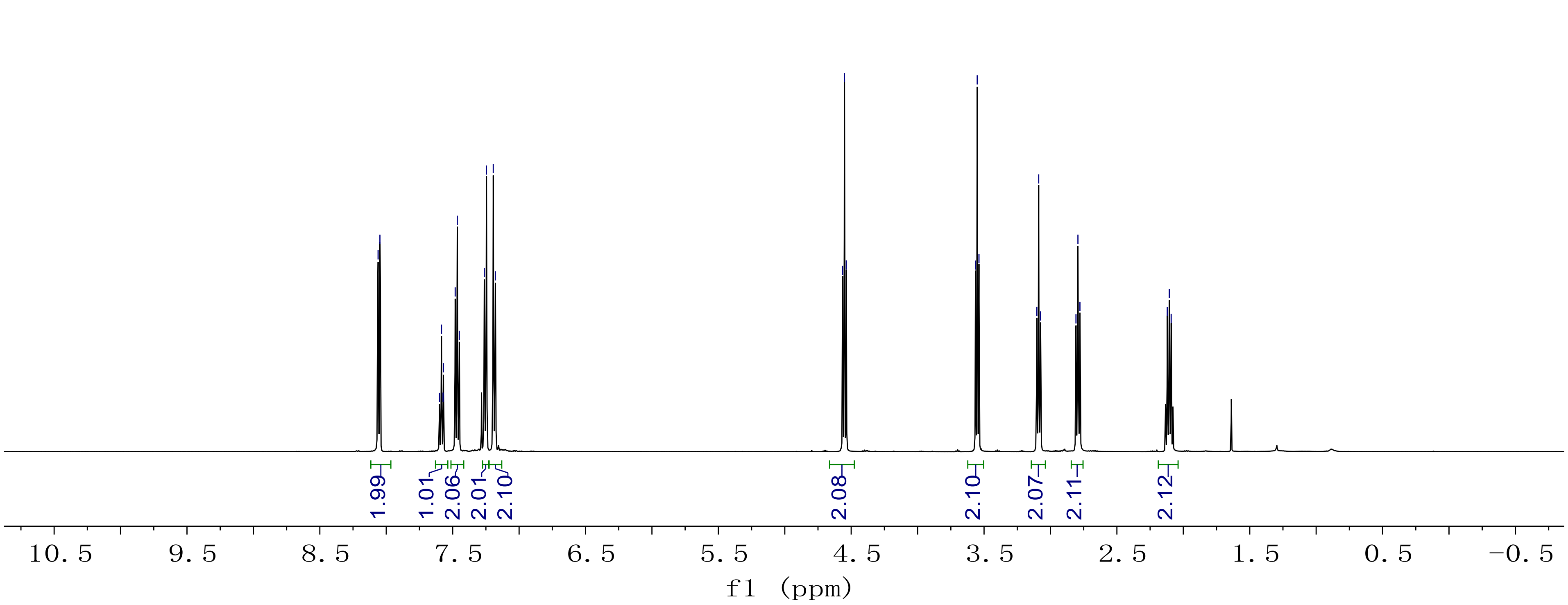

^{13}C NMR of arylethyl benzoate 14

$CDCl_3$, 23 ºC

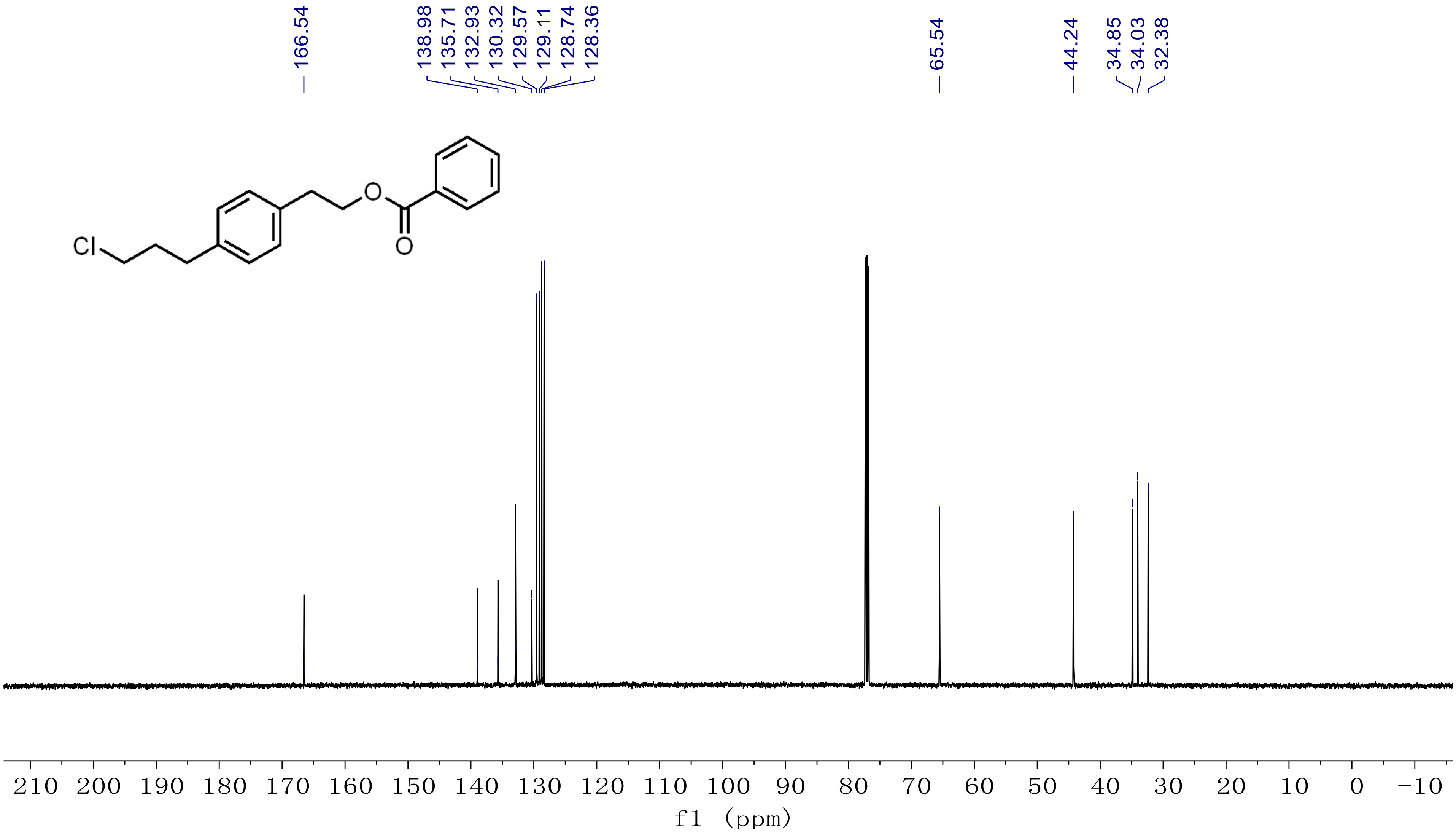

^{1}H NMR of fenofibrate-derived 2-arylethanol 15

$CDCl_3$, 23 ºC

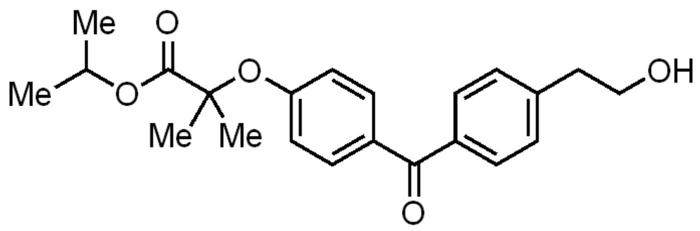

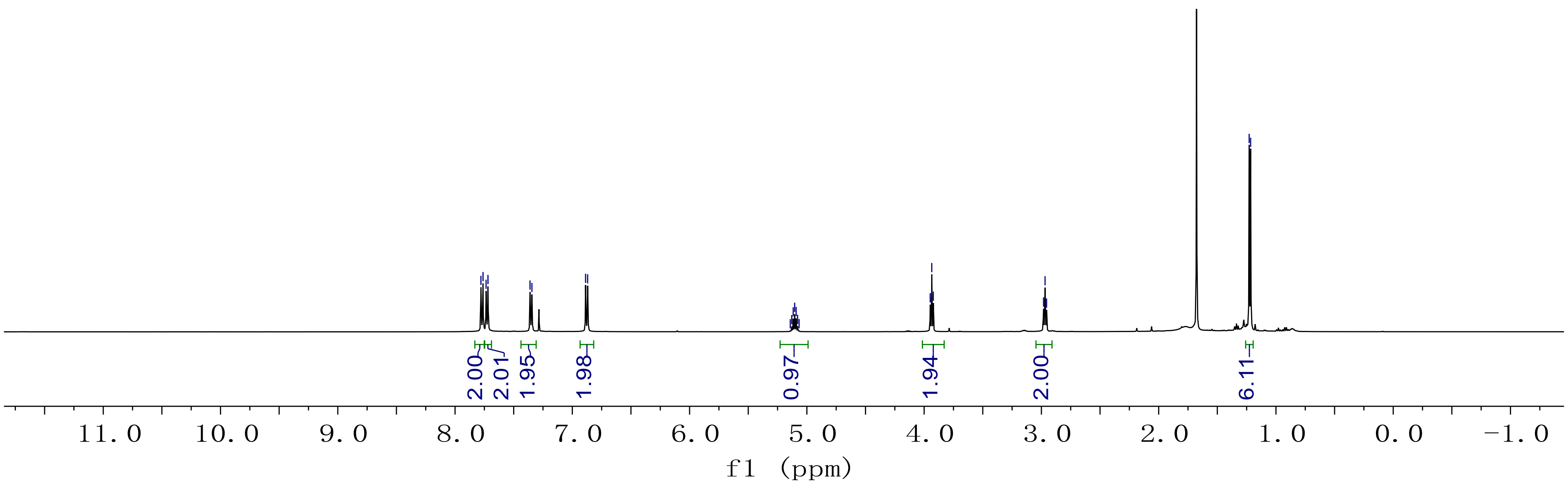

^{13}C NMR of fenofibrate-derived 2-arylethanol 15

CDCl$_3$, 23 ºC

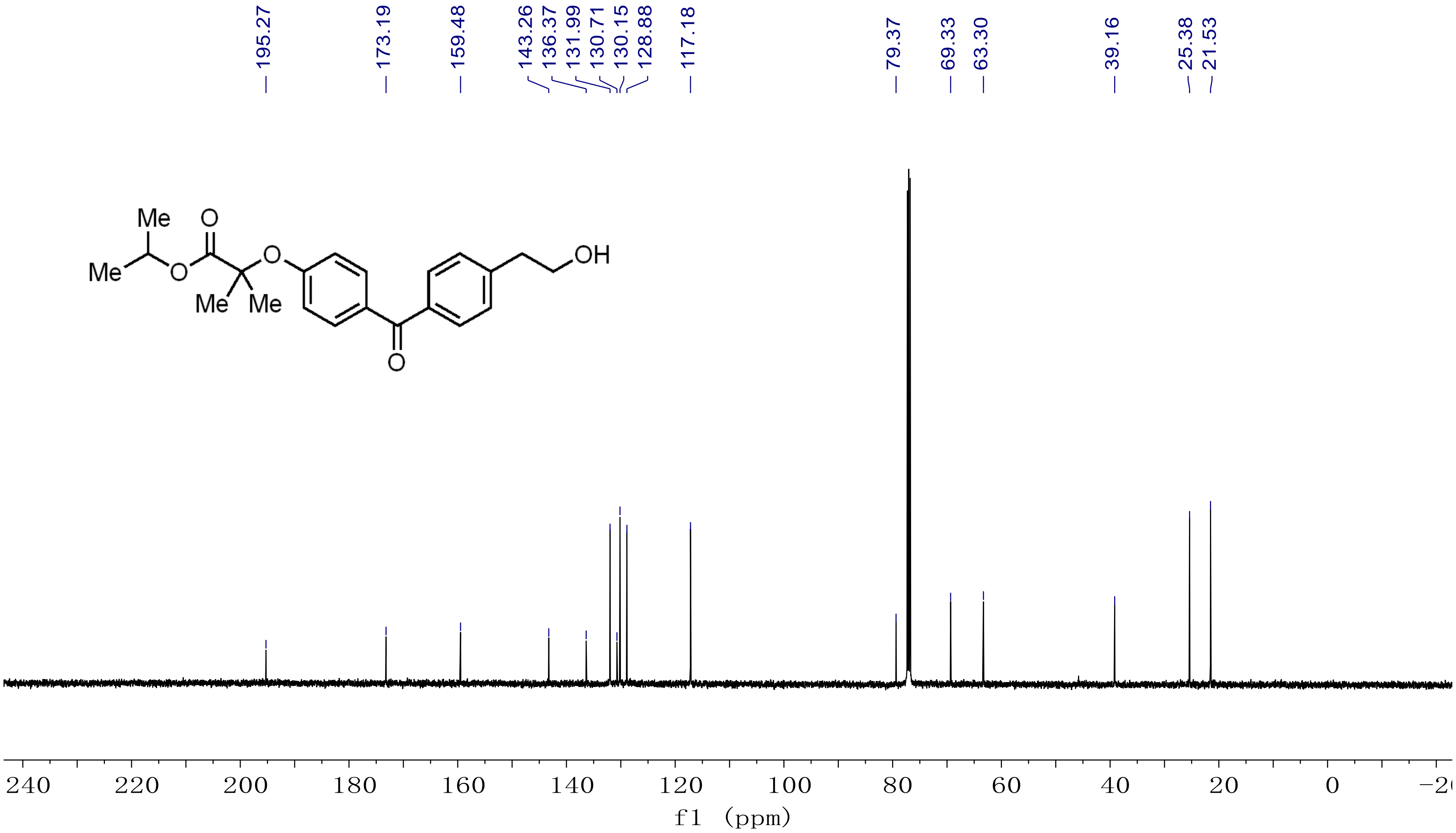

^{1}H NMR of arylethyl fluoride 16

$CDCl_3$, 23 ºC

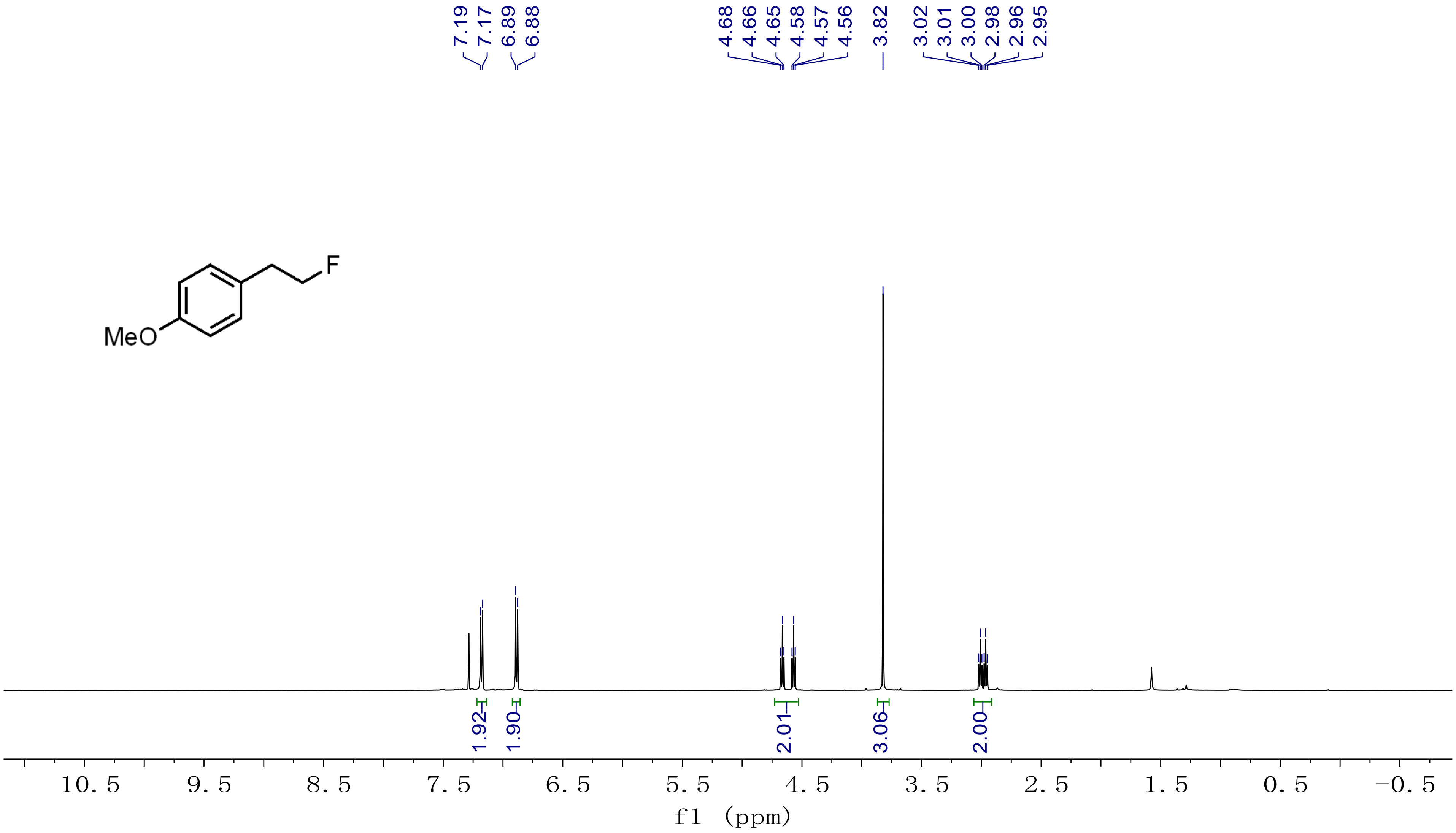

^{13}C NMR of arylethyl fluoride 16

$CDCl_3$, 23 ºC

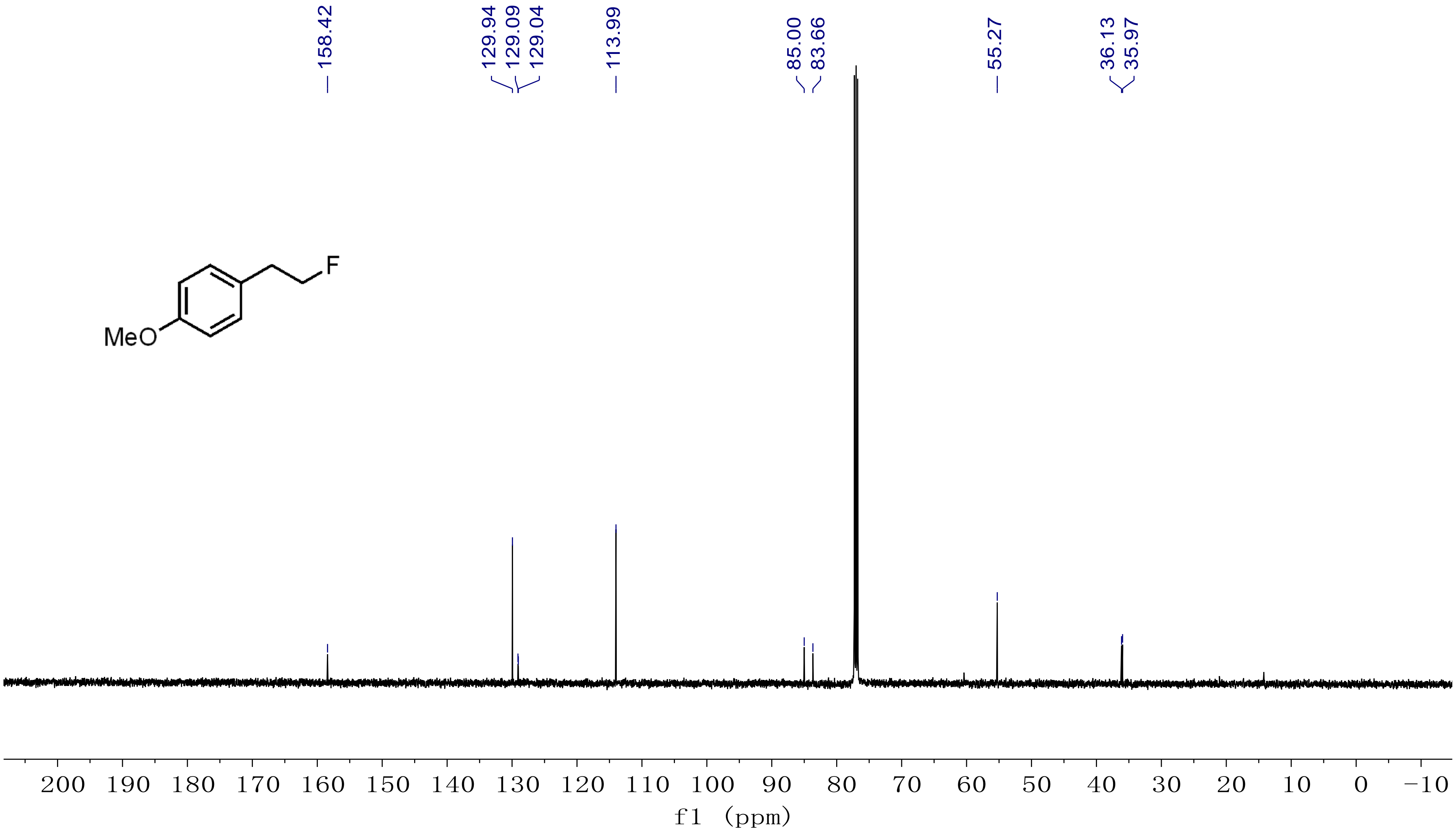

^{19}F NMR of arylethyl fluoride 16

$CDCl_3$, 23 ºC

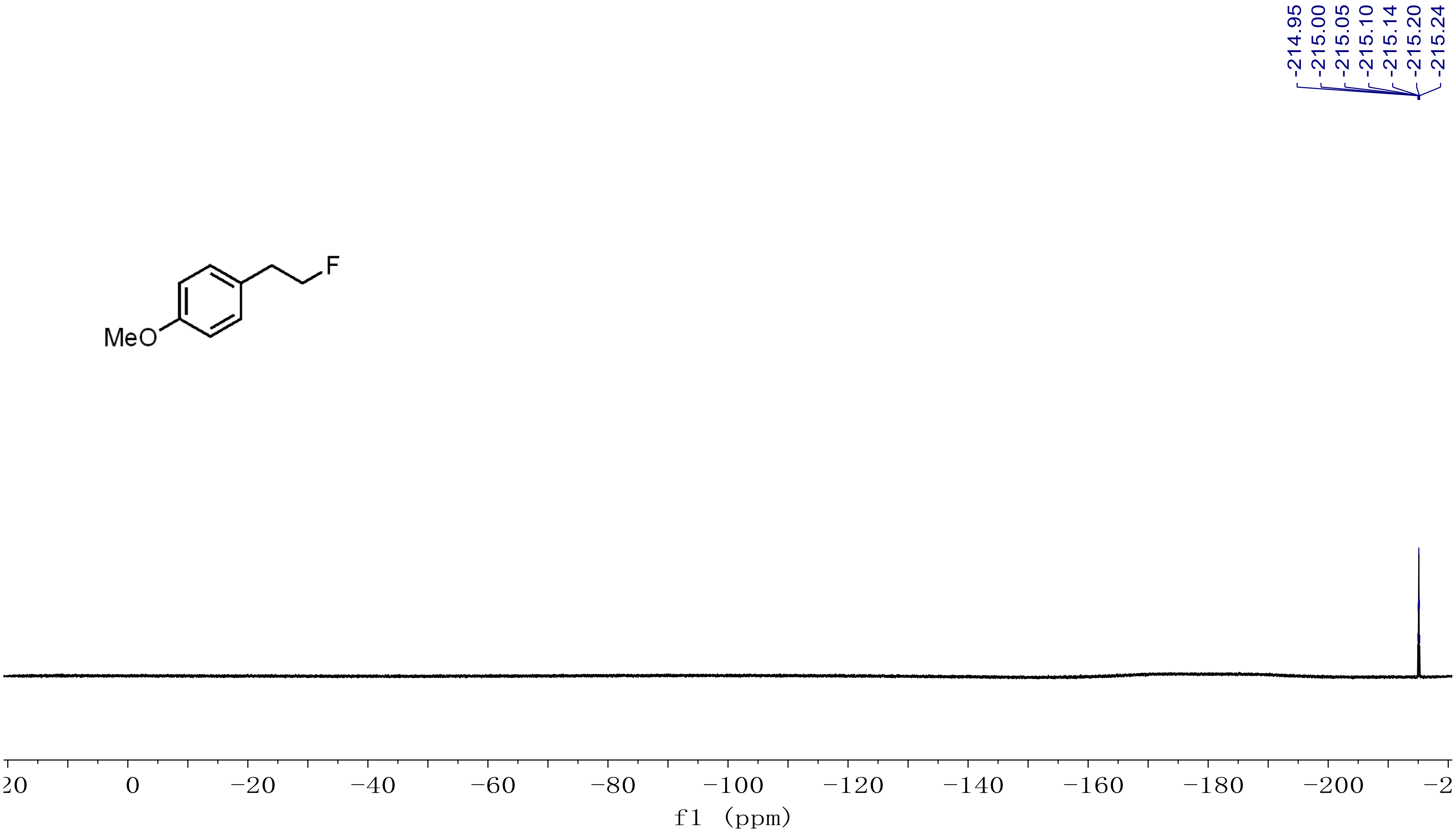

^{1}H NMR of diclofenac amide-derived arylethylamine 17

CDCl3, 23 ºC

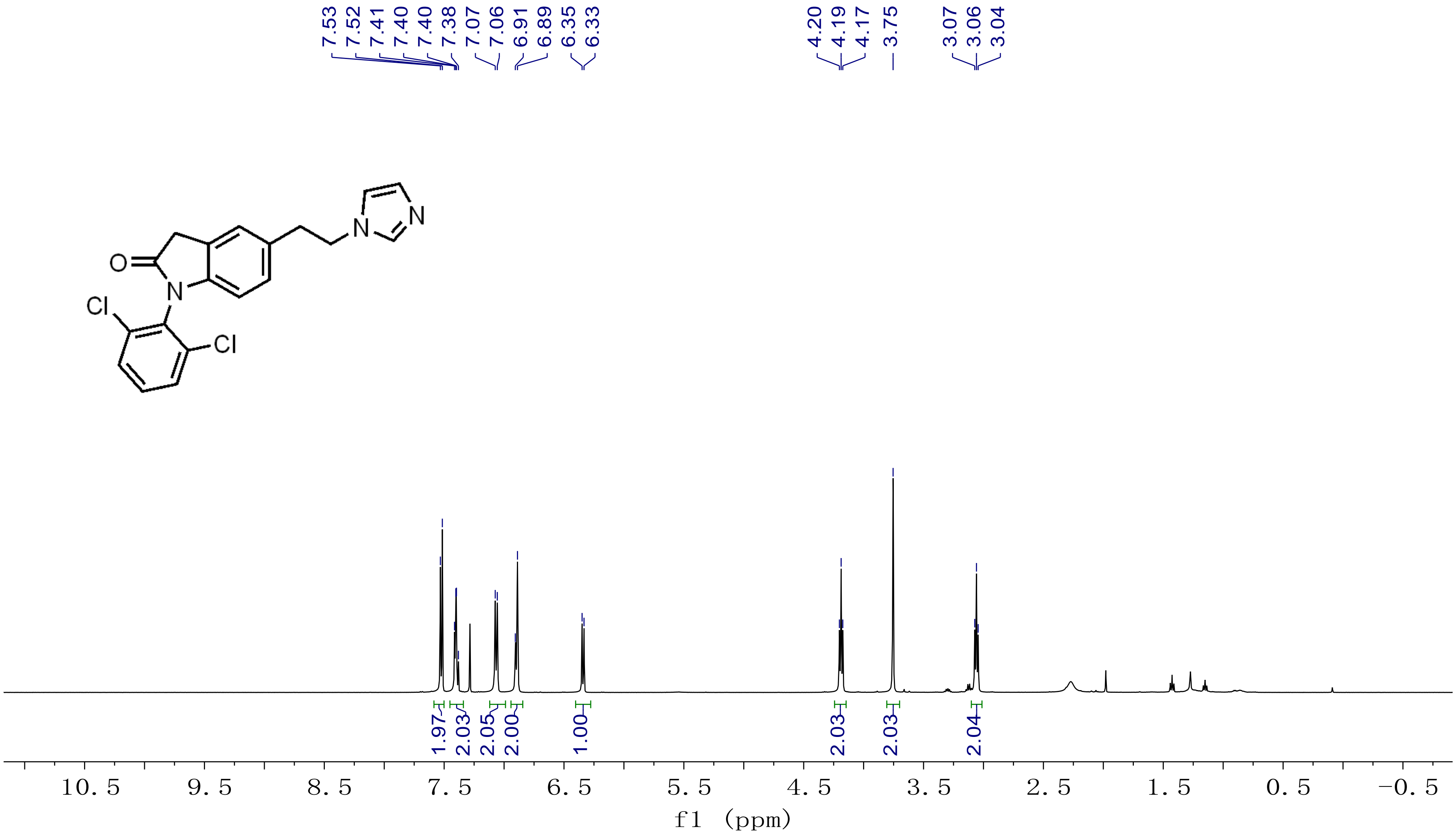

^{13}C NMR of diclofenac amide-derived arylethylamine 17

$CDCl_3$, 23 ºC

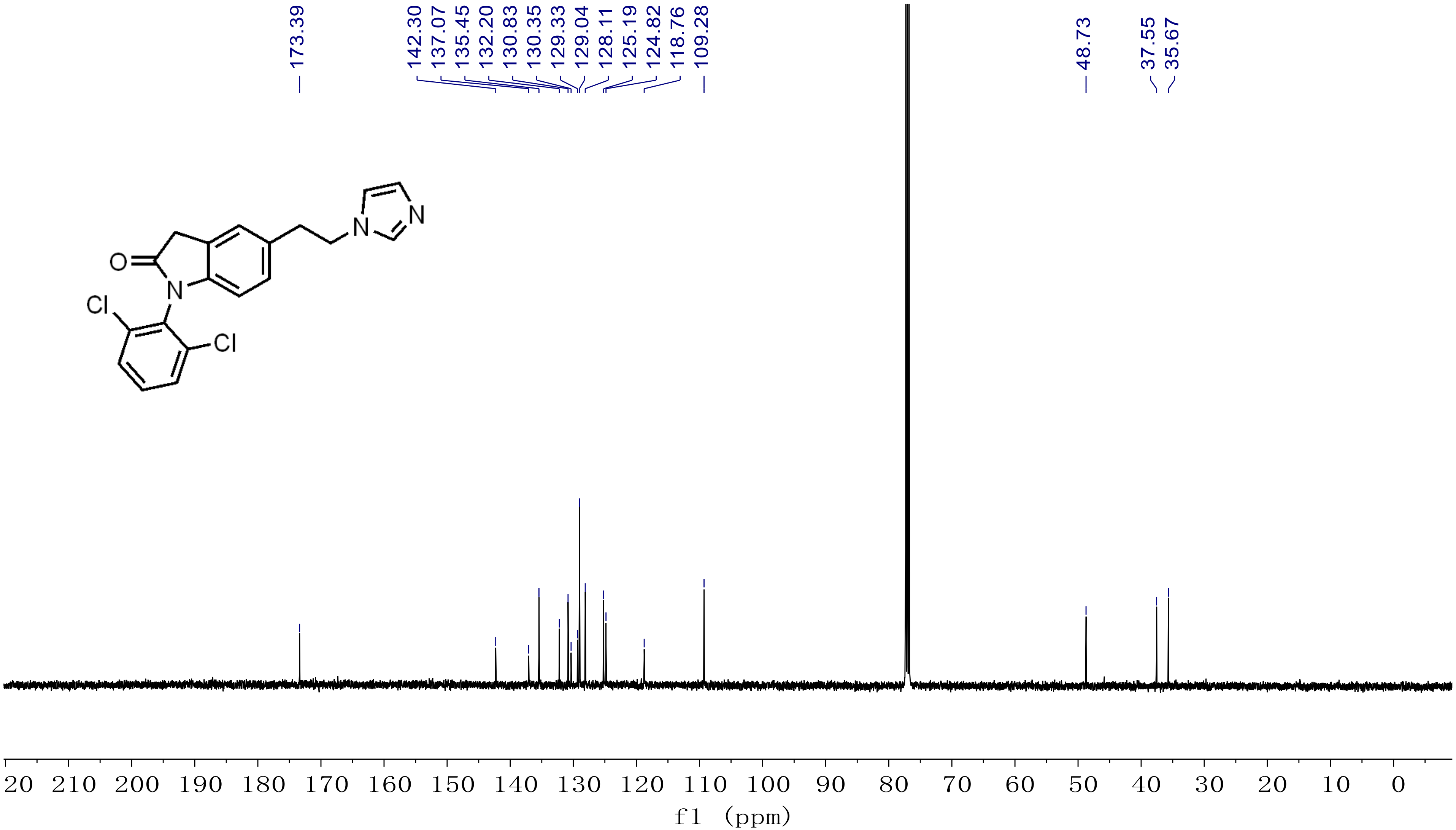

^{1}H NMR of arylethyl methyl ether 18

$CDCl_3$, 23 ºC

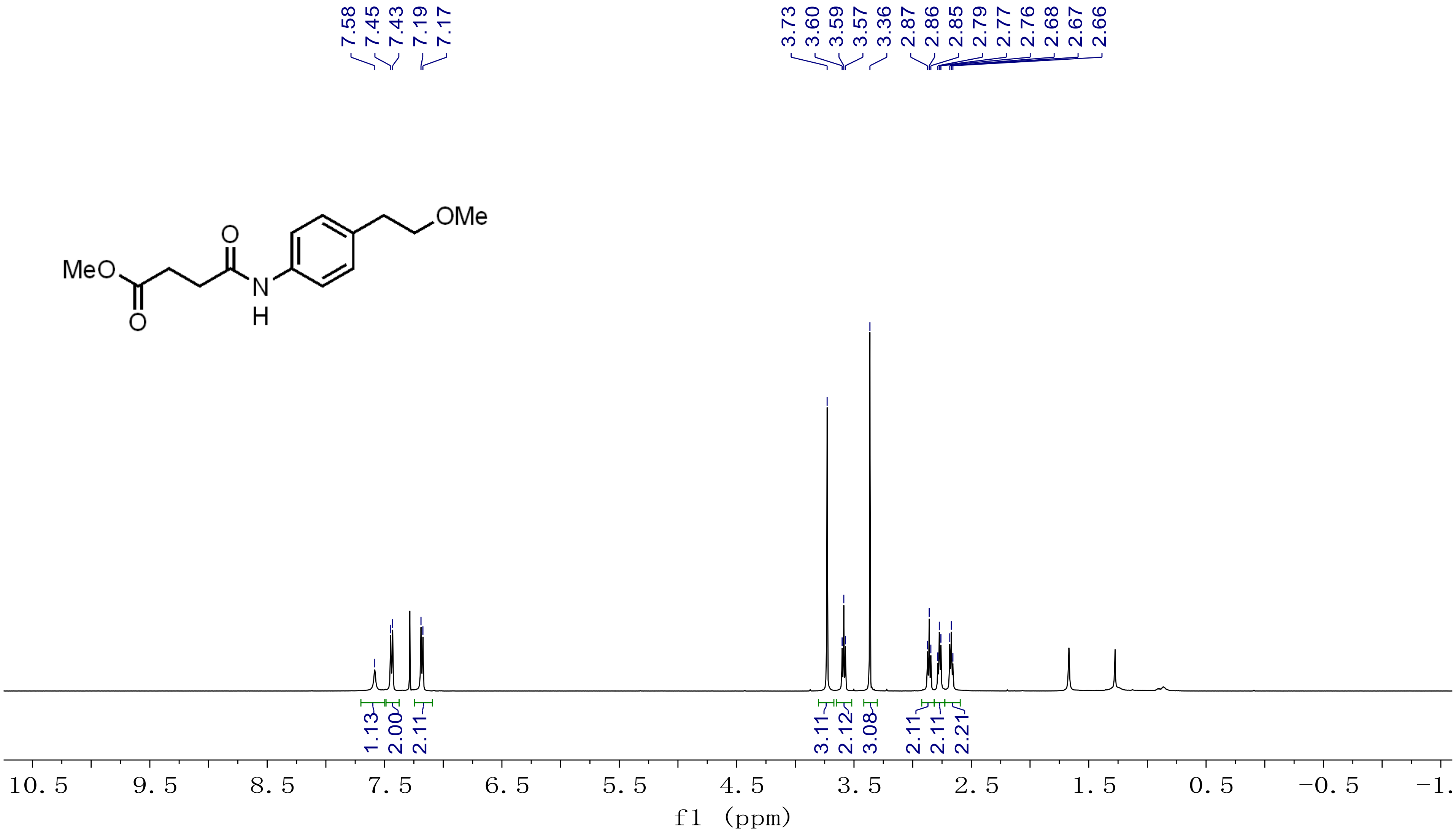

^{13}C NMR of arylethyl methyl ether 18

CDCl$_3$, 23 ºC

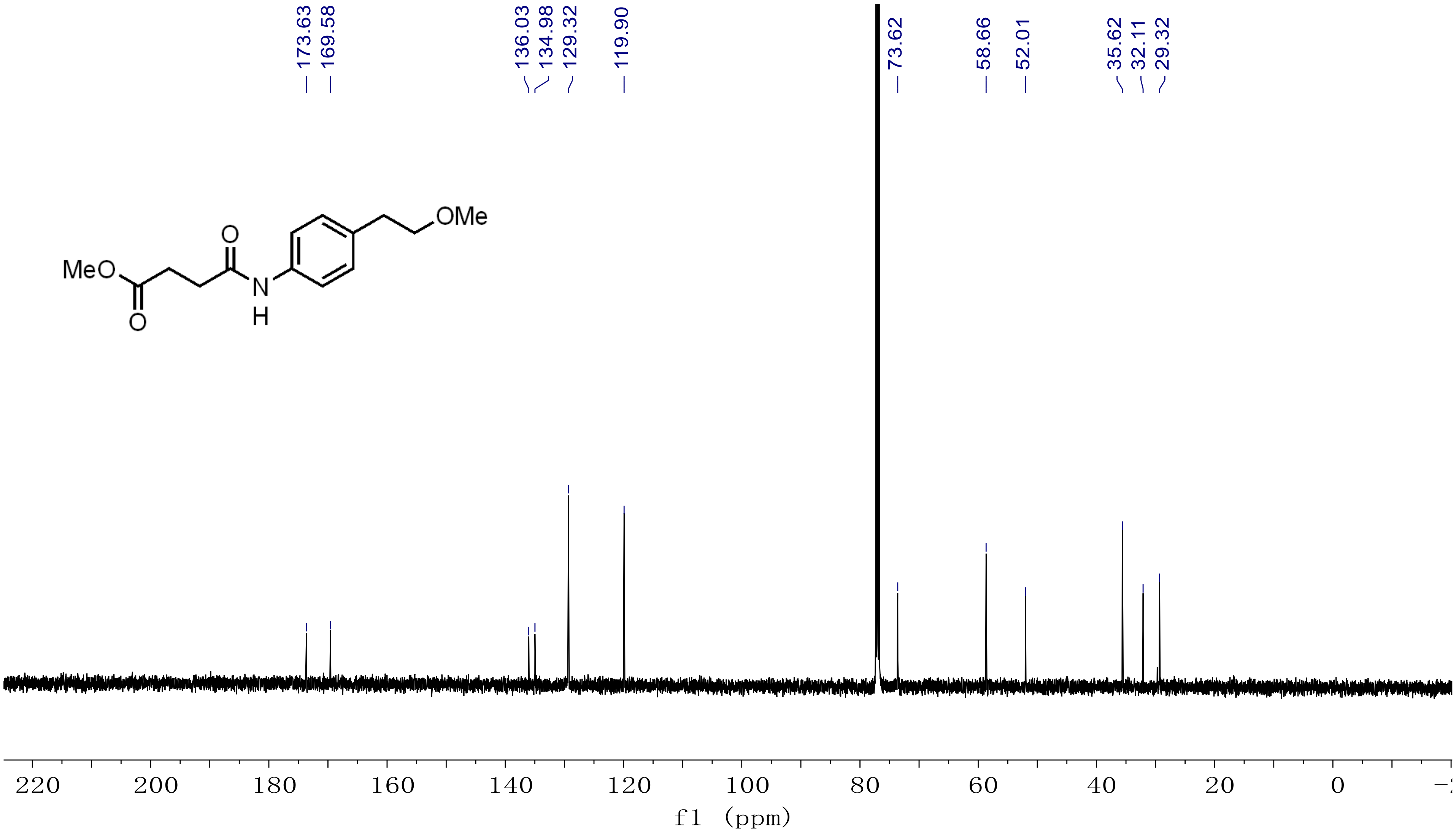

^{1}H NMR of cysteine-derived arylethyl thioether 19

$CDCl_3$, 23 ºC

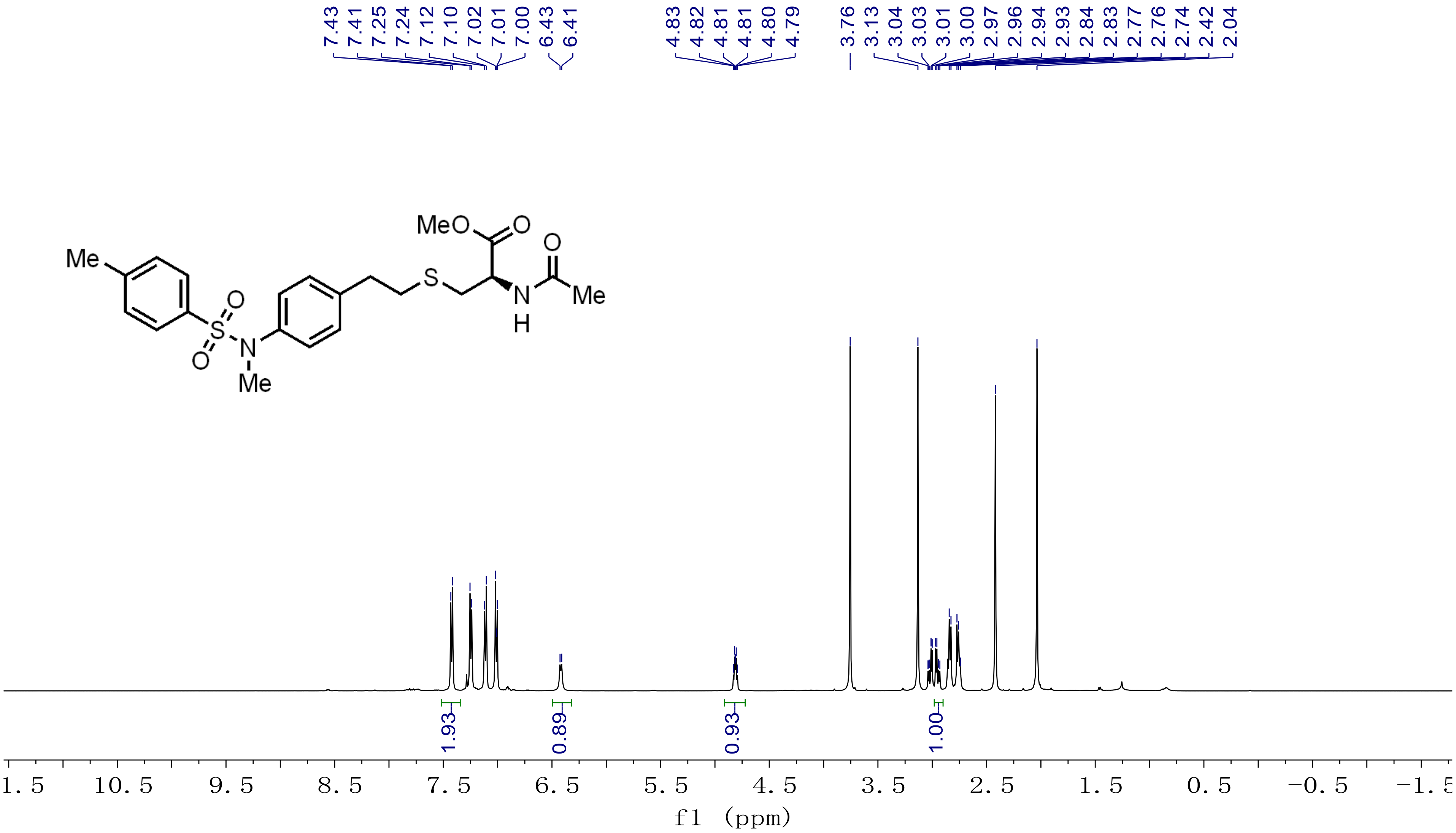

^{13}C NMR of cysteine-derived arylethyl thioether 19

$CDCl_3$, 23 ºC

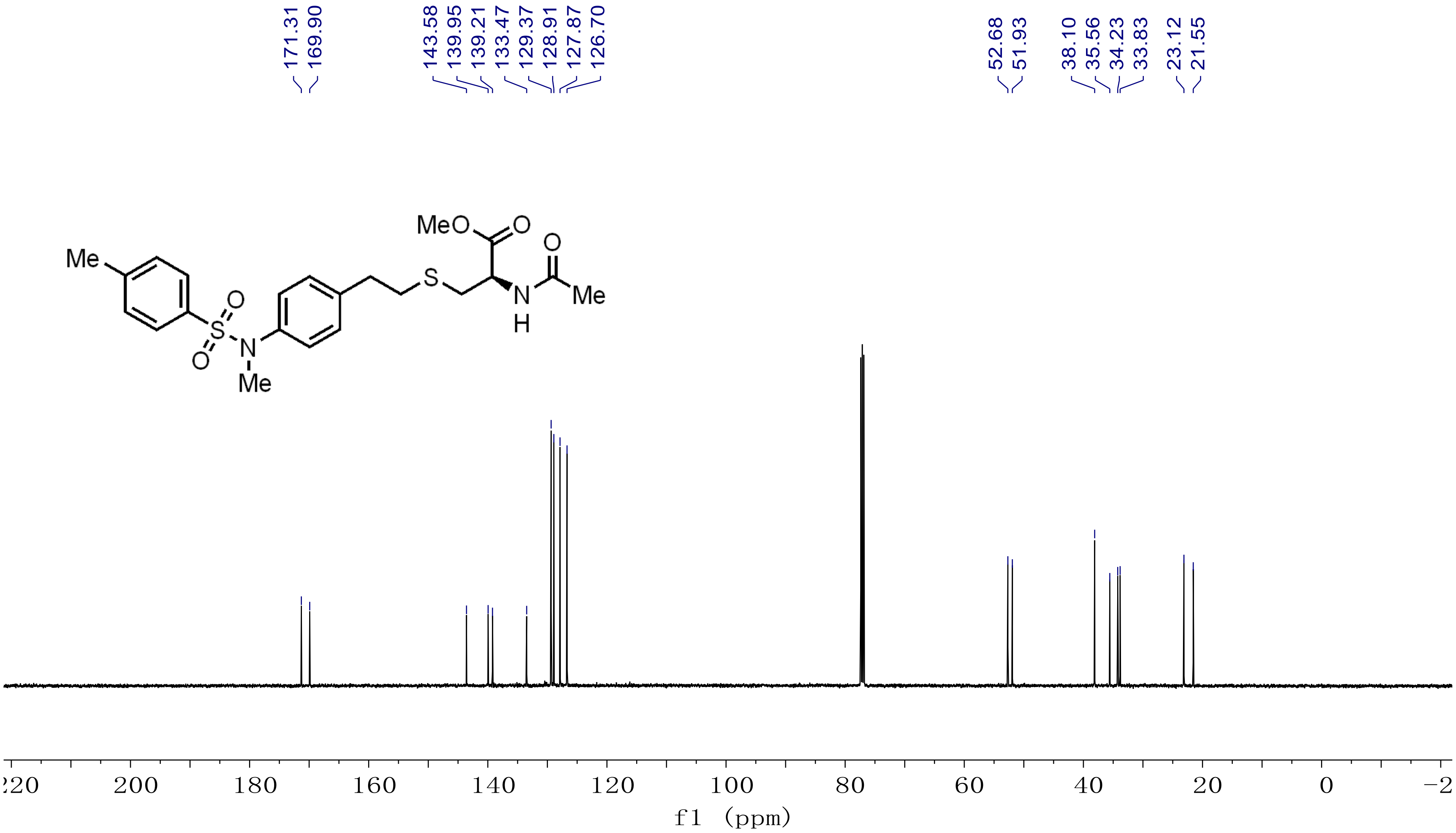

^{1}H NMR of arylpropionitrile 20

$CDCl_3$, 23 ºC

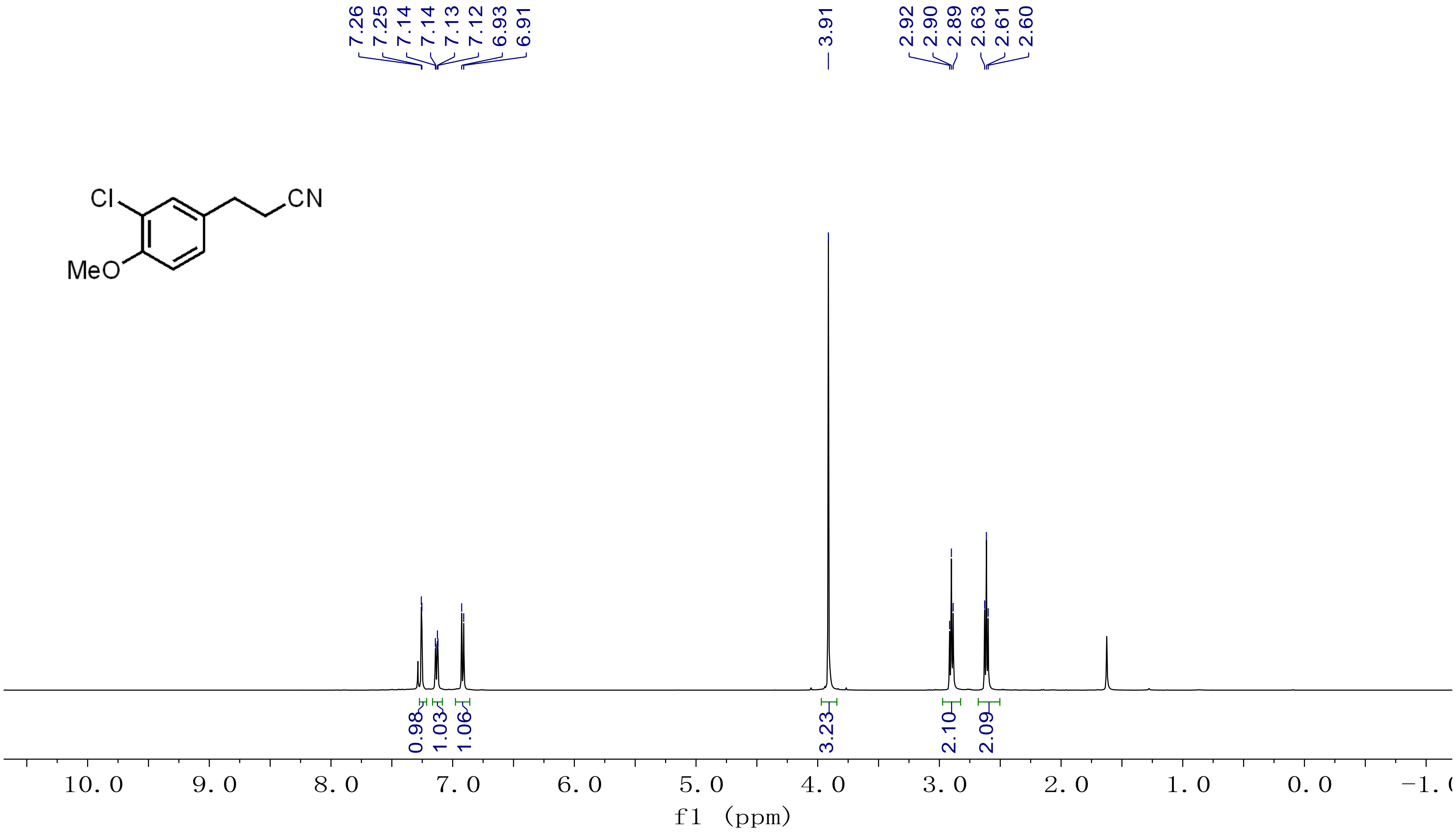

^{13}C NMR of arylpropionitrile 20

$CDCl_3$, 23 ºC

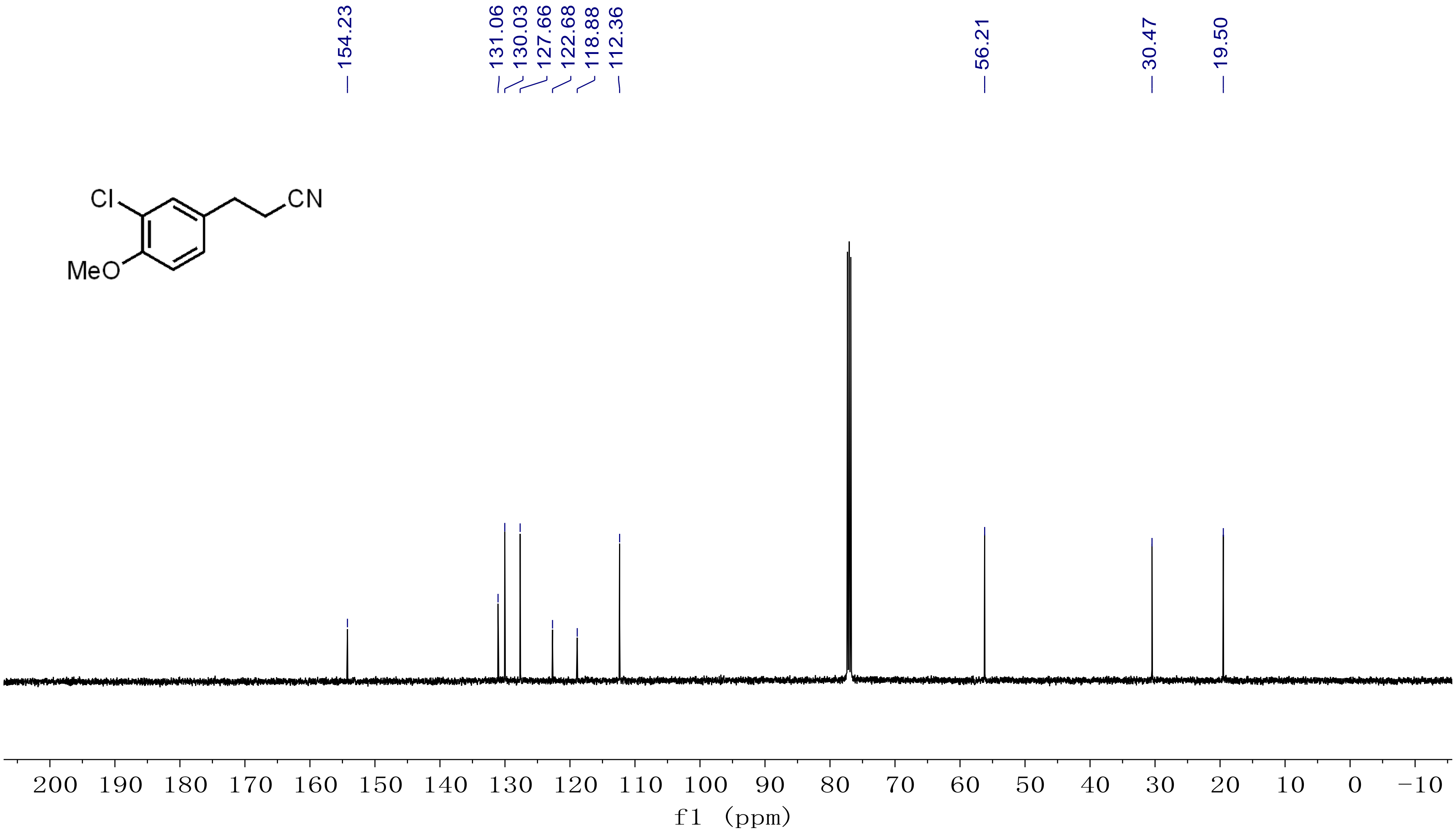

¹H NMR of calone-derived arylethylamine 21

$CDCl_3$, 23 ºC

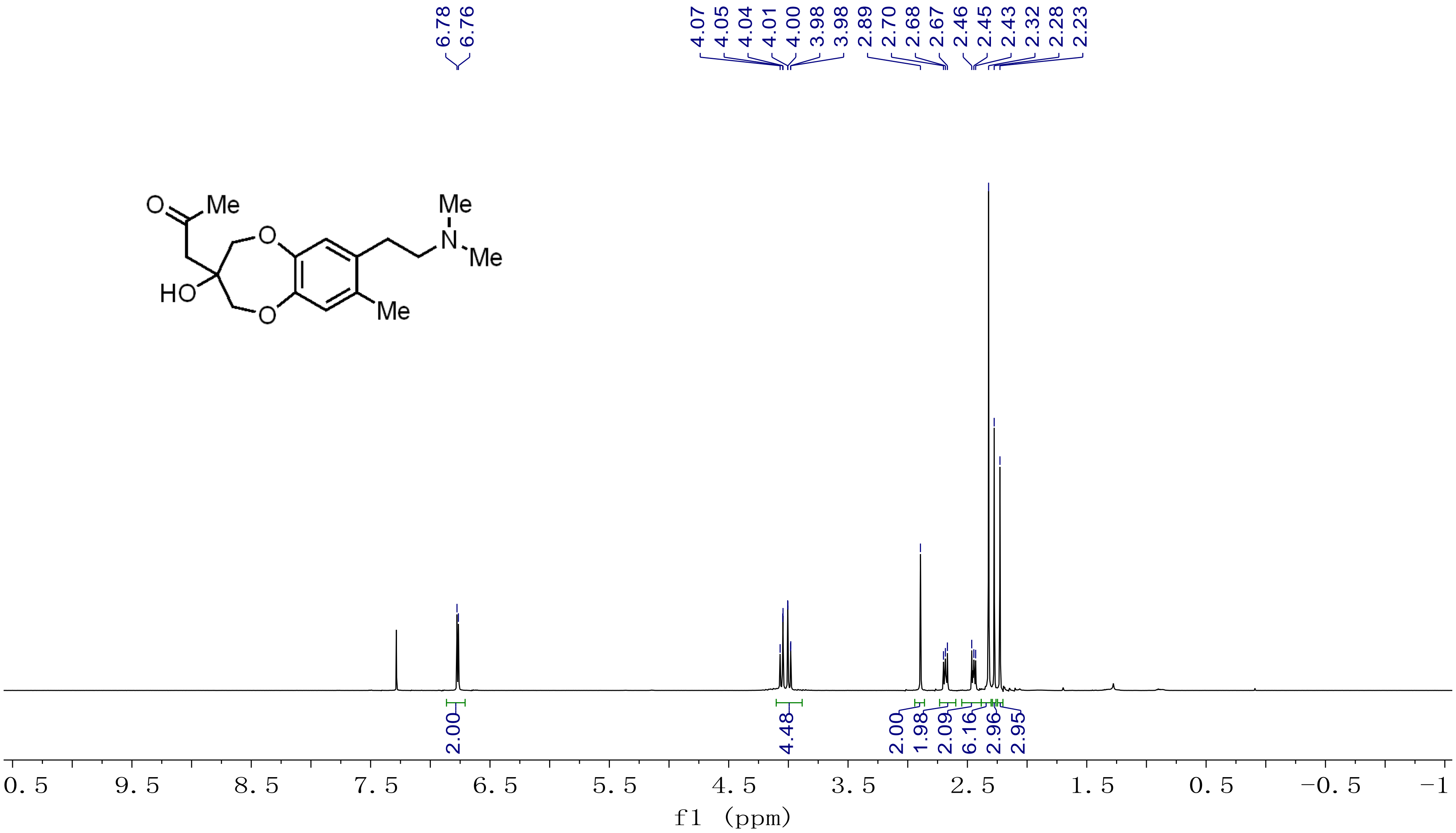

^{13}C NMR of calone-derived arylethylamine 21

$CDCl_3$, 23 ºC

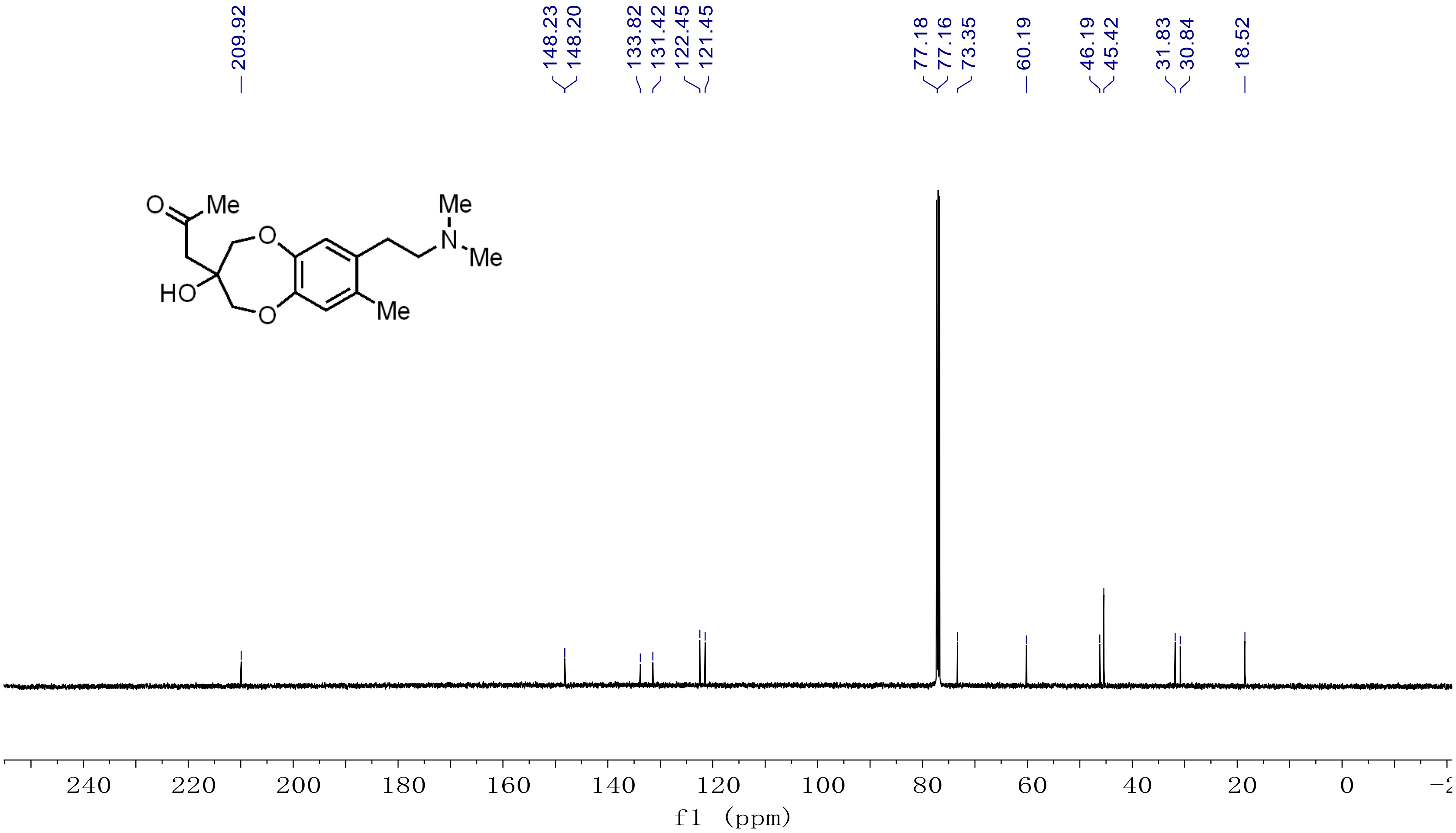

^{1}H NMR of arylethylamine 22

$CDCl_3$, 23 ºC

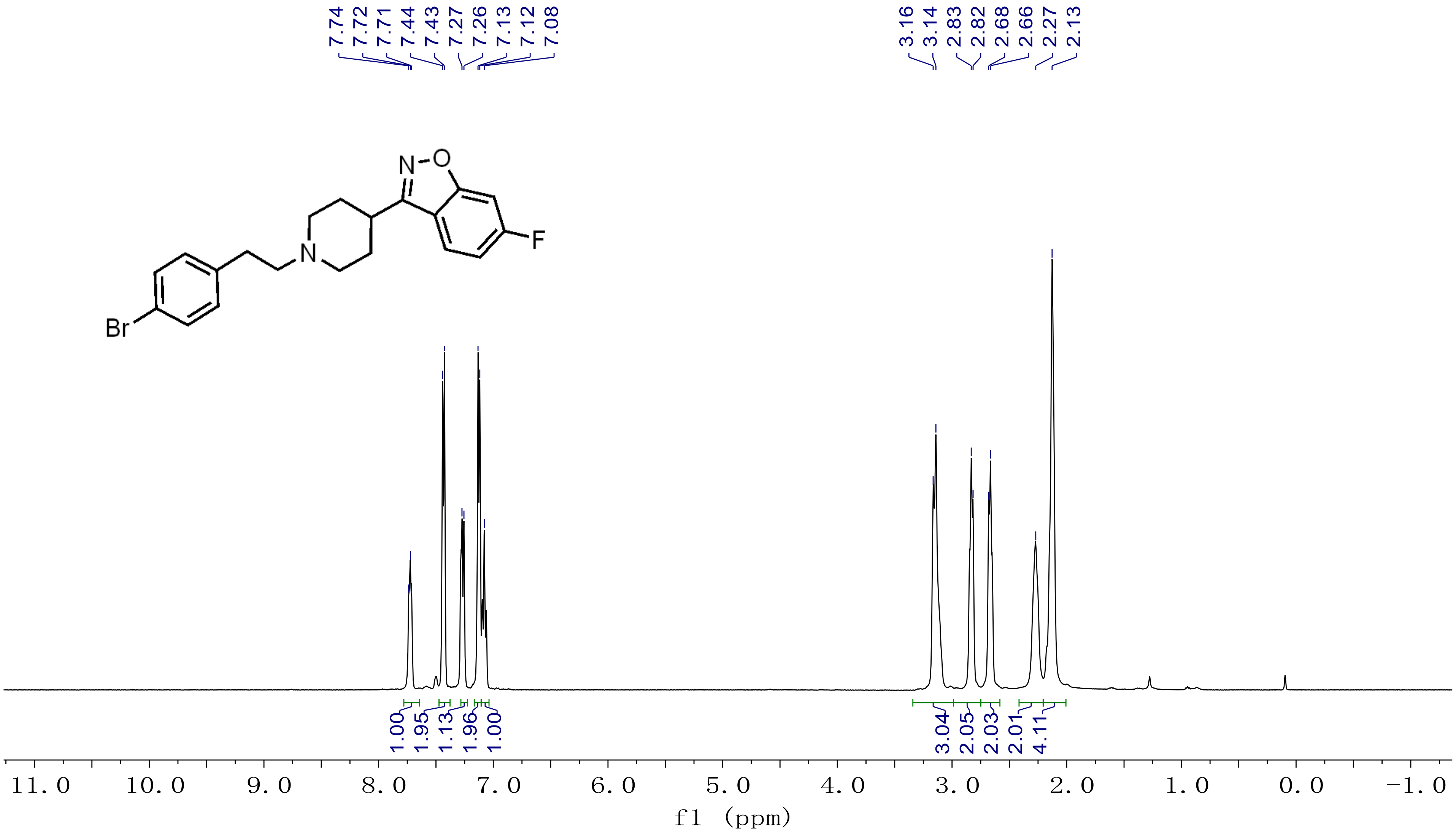

^{13}C NMR of arylethylamine 22

$CDCl_3$, 23 ºC

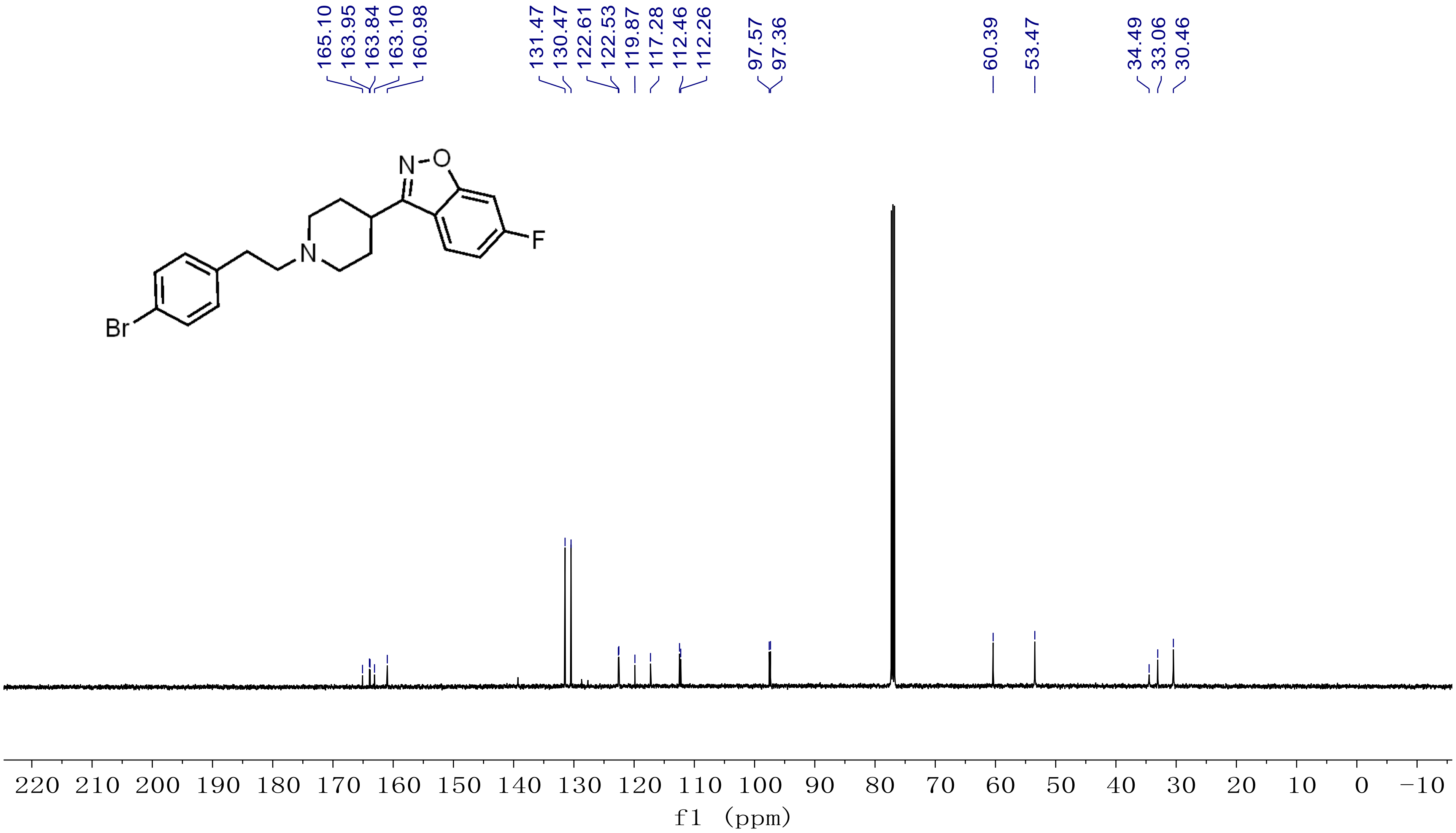

^{19}F NMR of arylethylamine 22

$CDCl_3$, 23 ºC

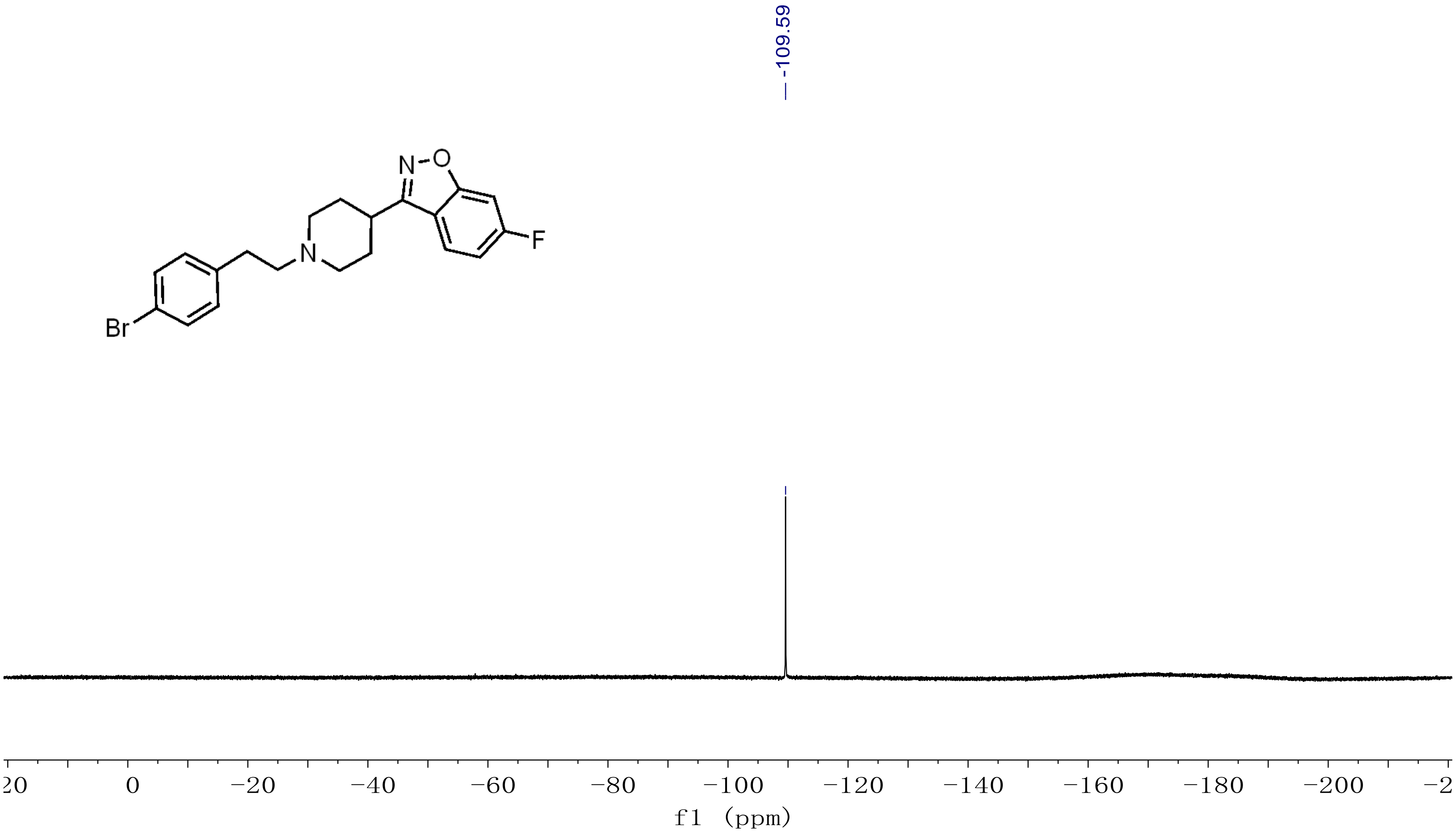

^{1}H NMR of duloxetine-derived arylethylamine 23

$CDCl_3$, 23 ºC

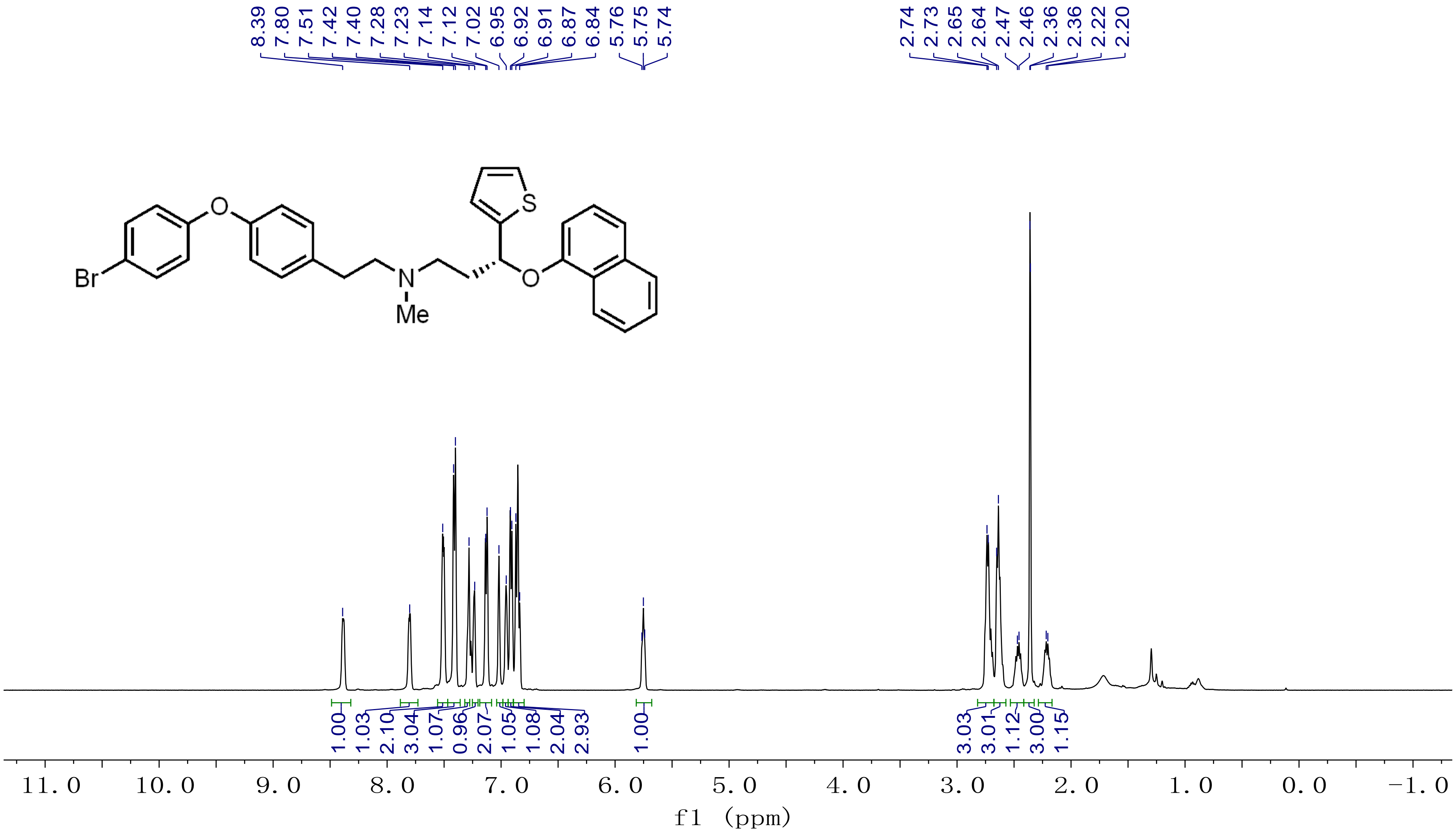

^{13}C NMR of duloxetine-derived arylethylamine 23

$CDCl_3$, 23 ºC

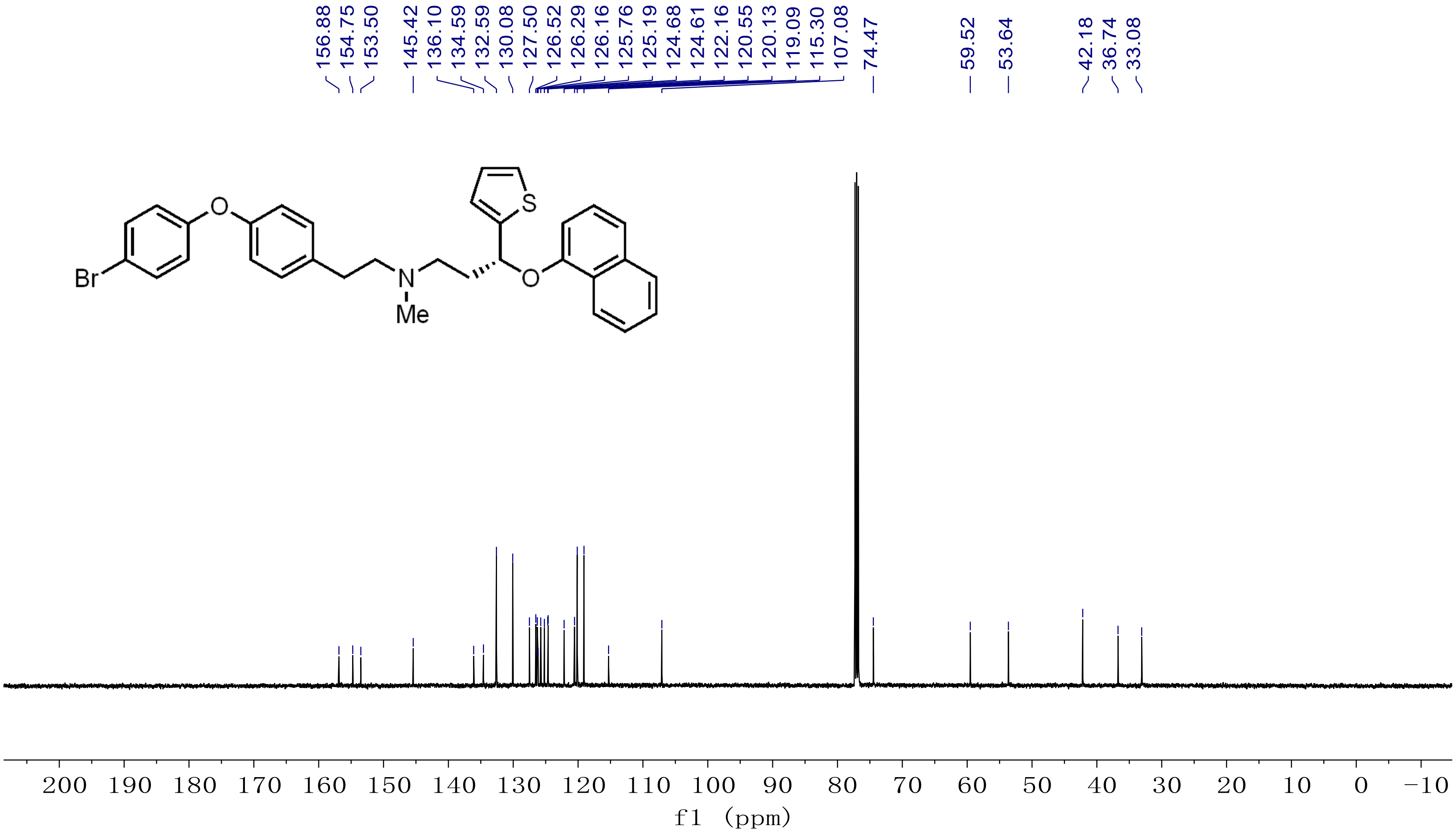

^{1}H NMR of arylethyl bromide 24

CDCl$_3$, 23 ºC

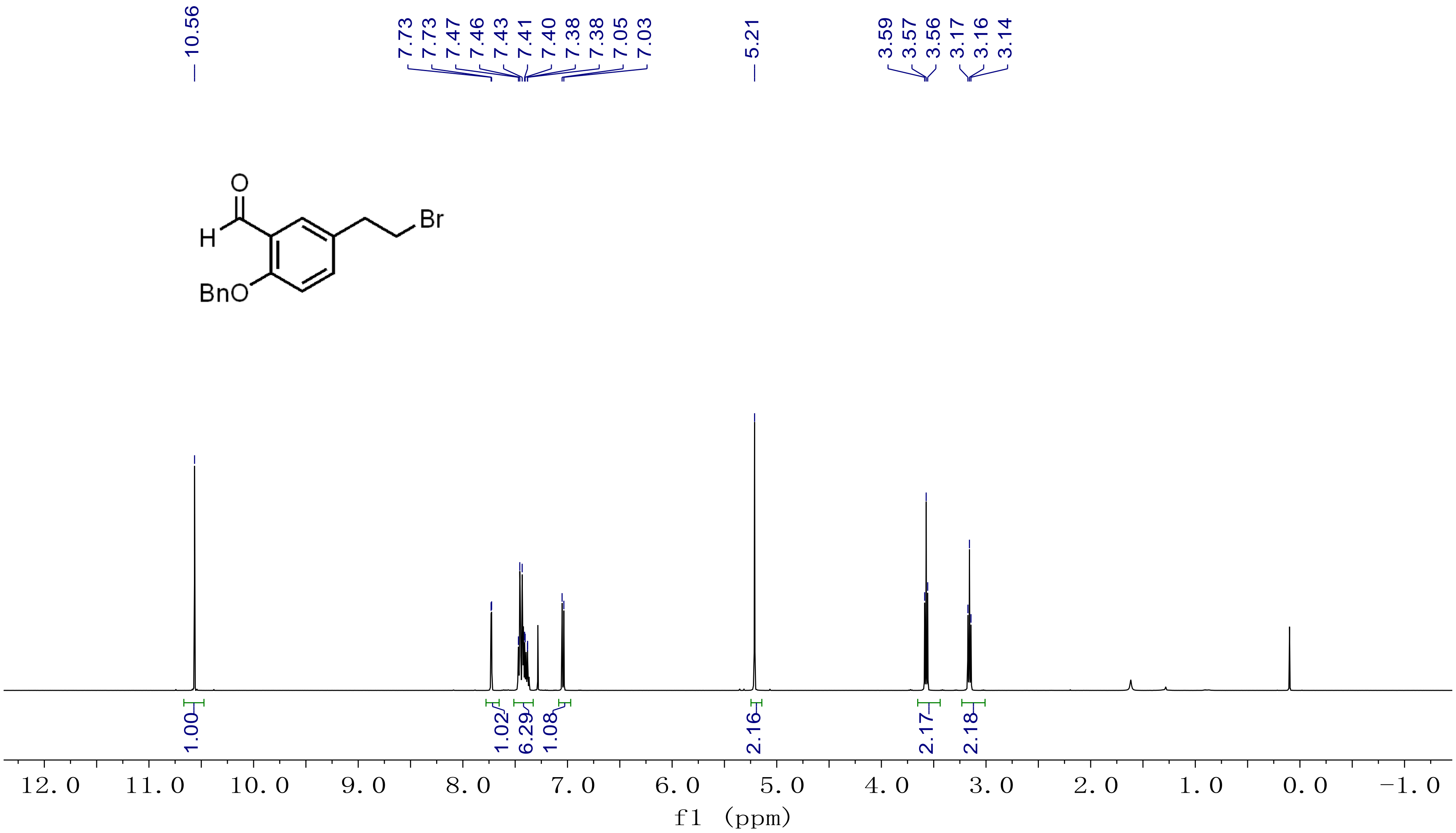

^{13}C NMR of arylethyl bromide 24

$CDCl_3$, 23 ºC

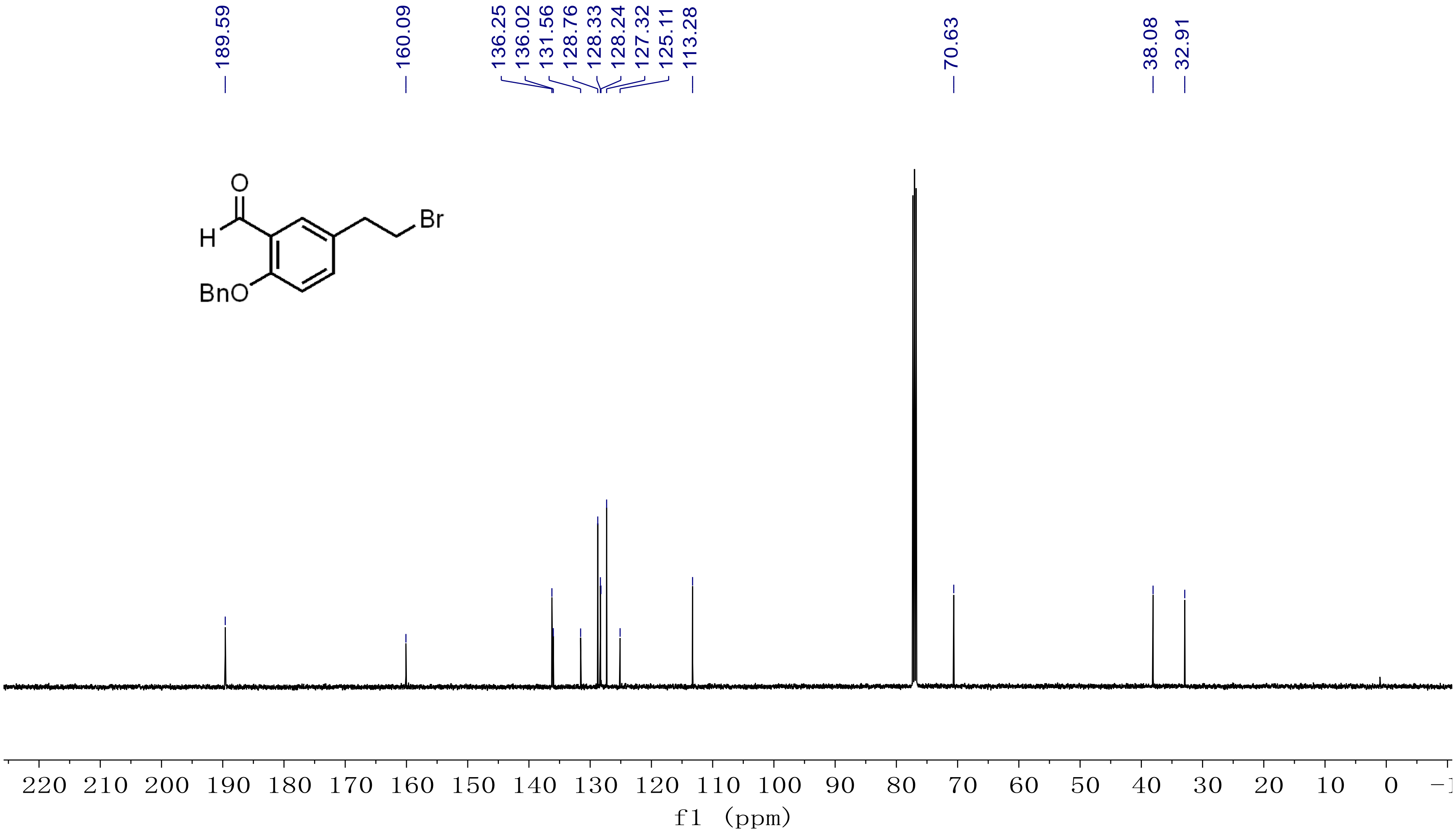

^{1}H NMR of arylethyl phosphonate 25

$CDCl_3$, 23 ºC

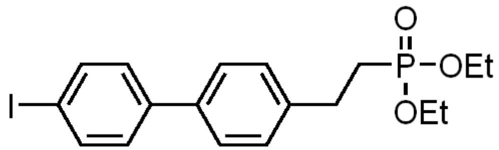

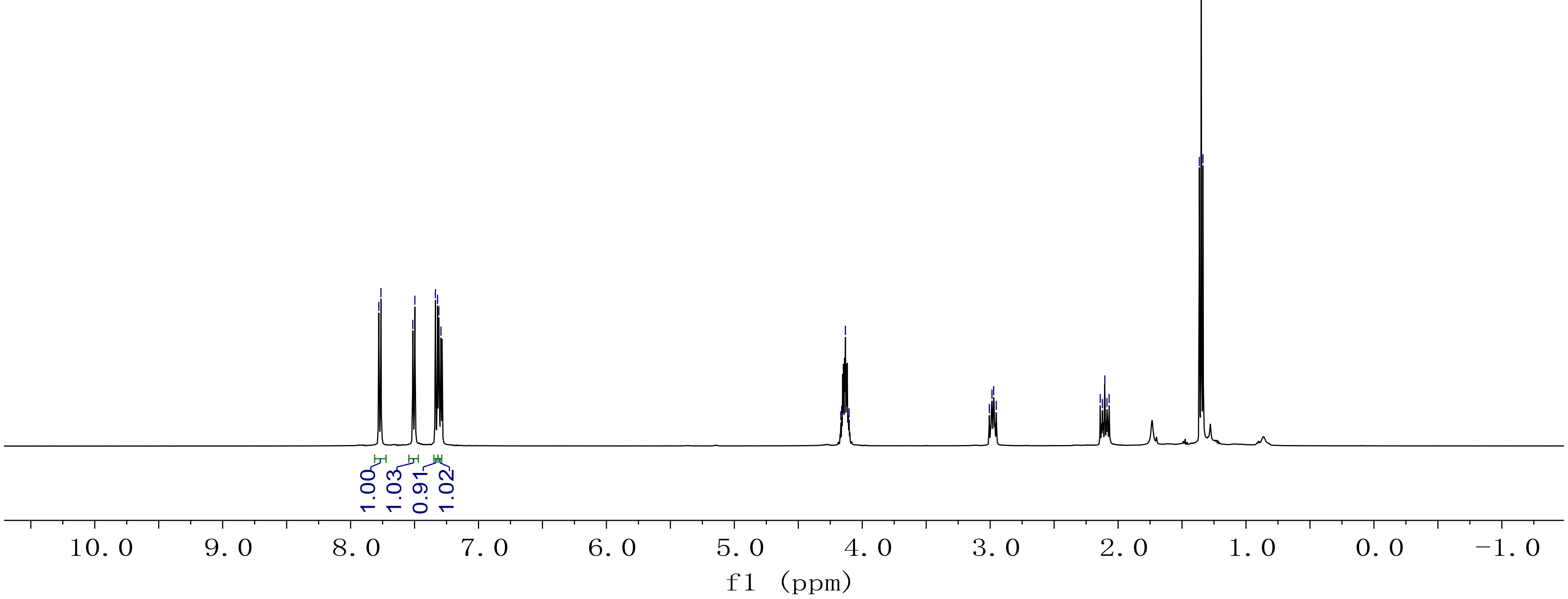

^{13}C NMR of arylethyl phosphonate 25

$CDCl_3$, 23 ºC

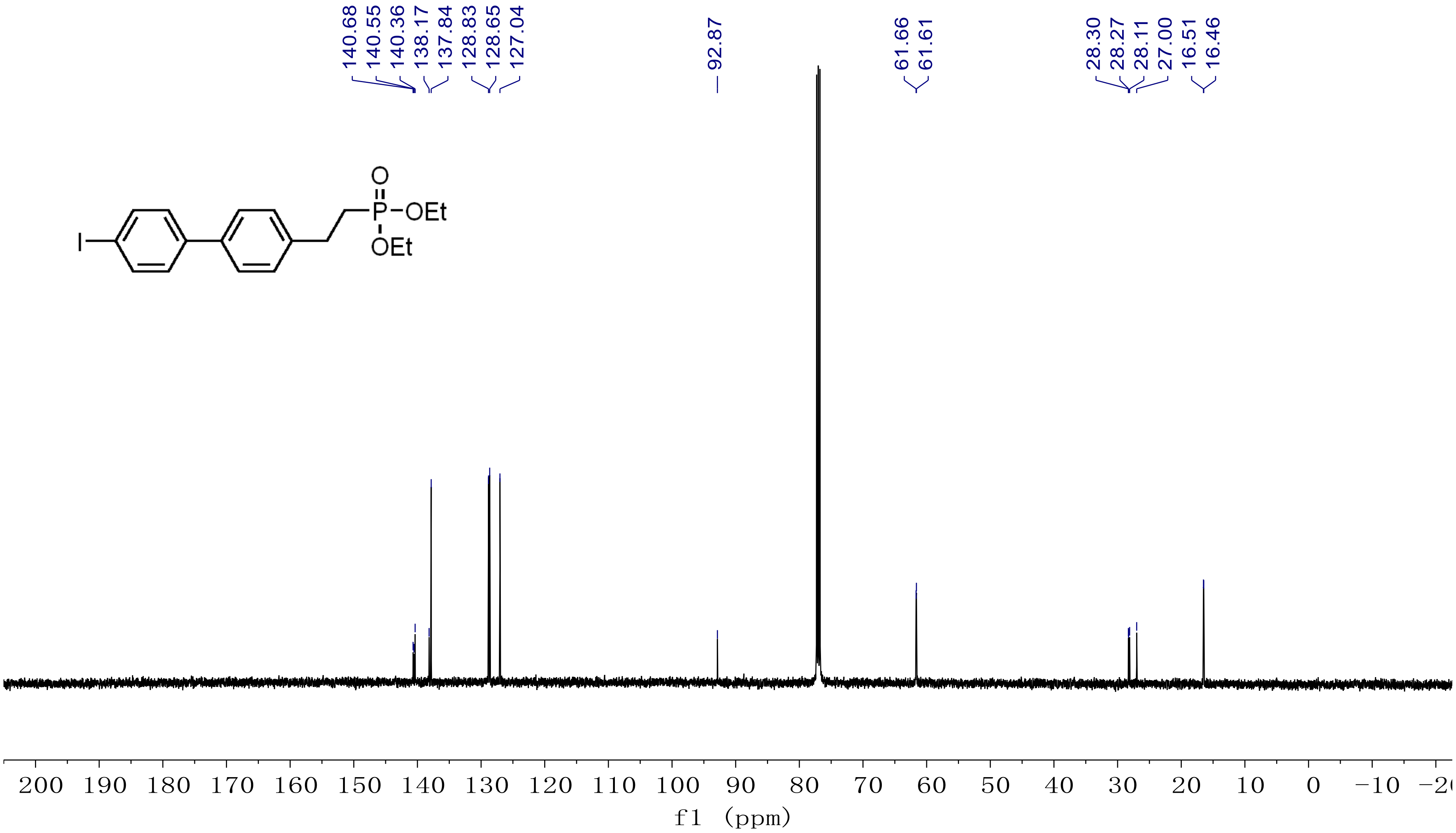

^{31}P NMR of arylethyl phosphonate 25

$CDCl_3$, 23 ºC

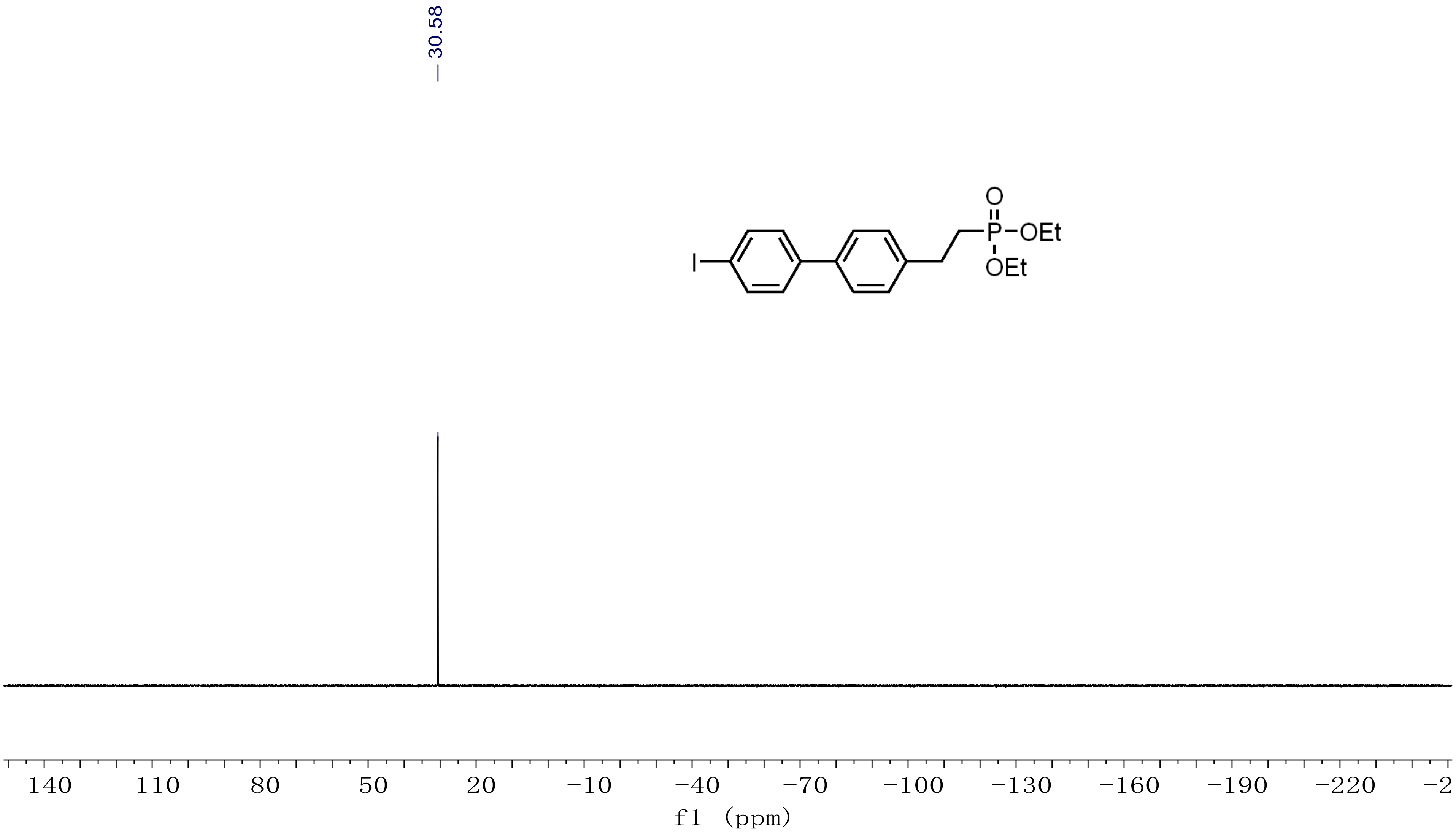

^{1}H NMR of arylethyl thiocyanate 26

$CDCl_3$, 23 ºC

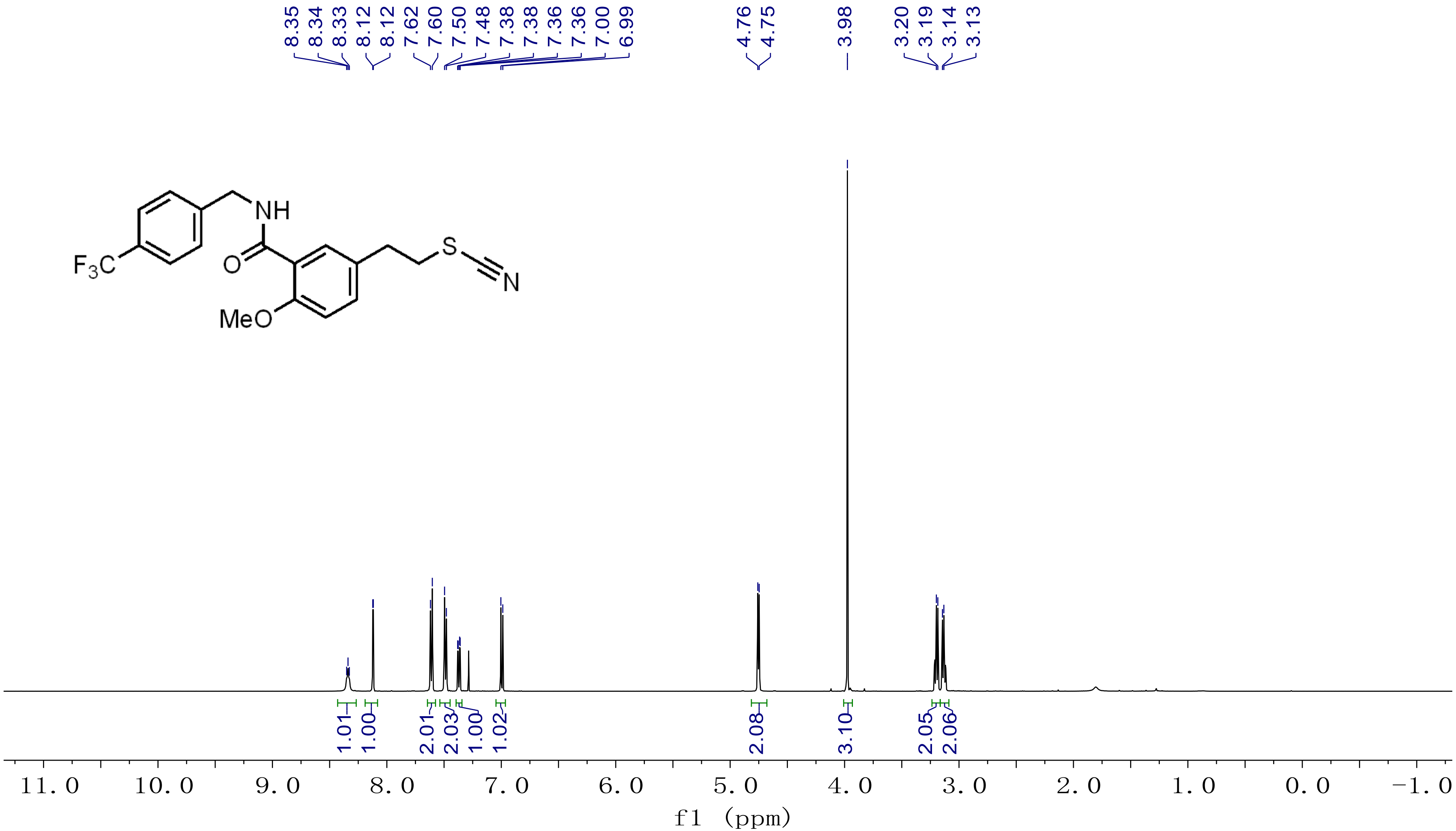

^{13}C NMR of arylethyl thiocyanate 26

CDCl$_3$, 23 ºC

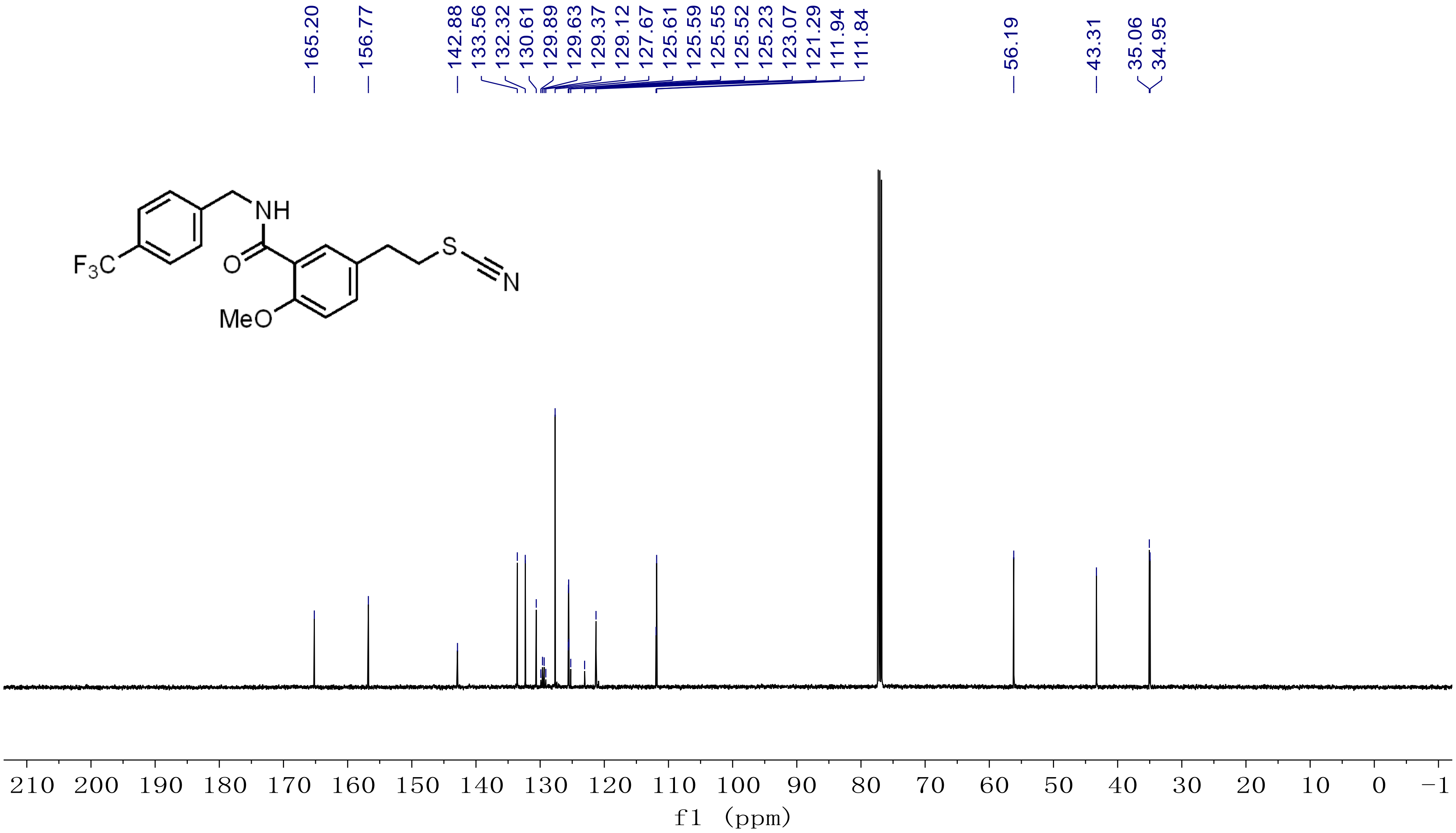

^{19}F NMR of arylethyl thiocyanate 26

$CDCl_3$, 23 ºC

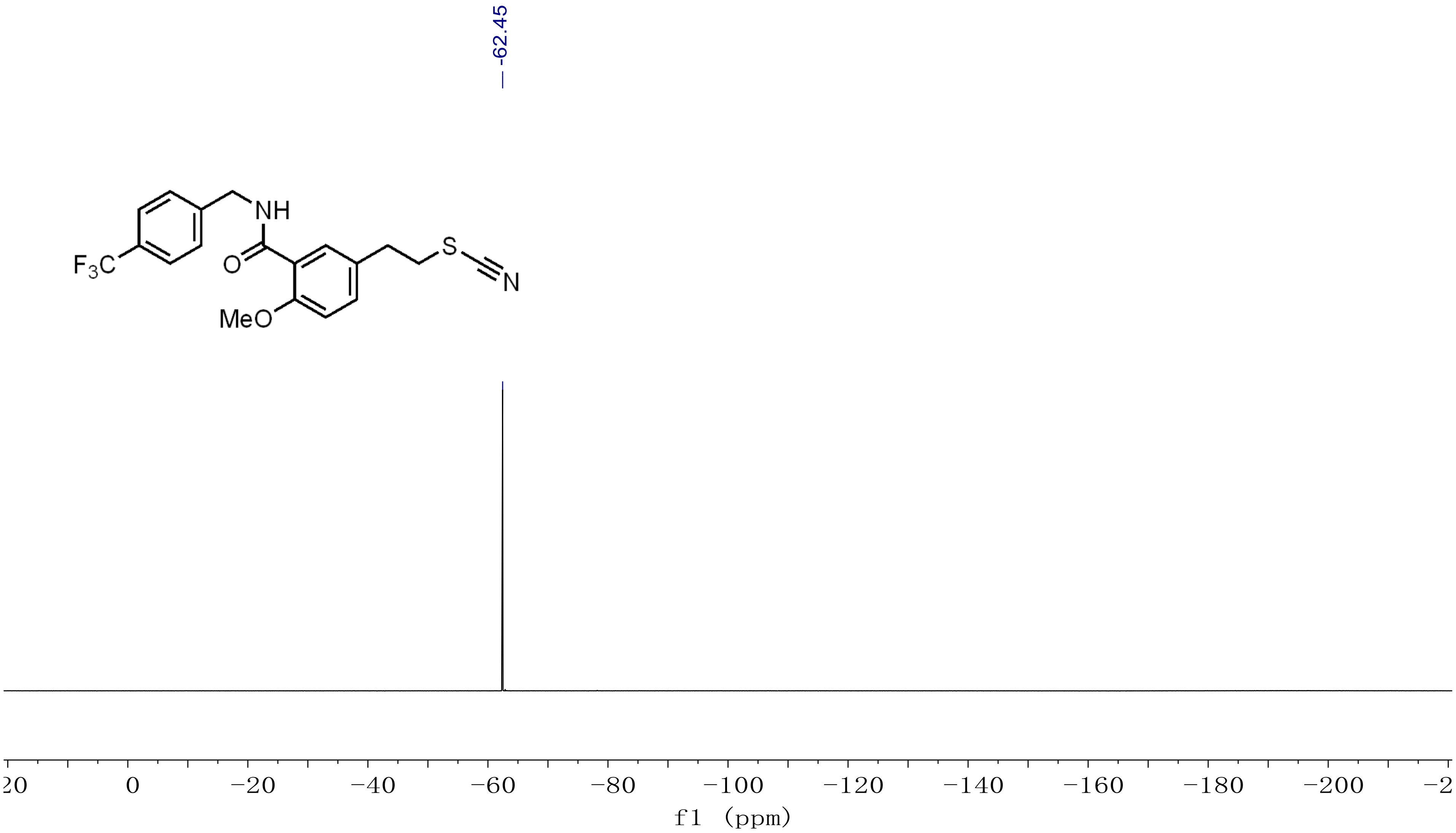

^{1}H NMR of pyriproxyfen- and cytisine-derived arylethylamine 27

CD_3CN, 23 ºC

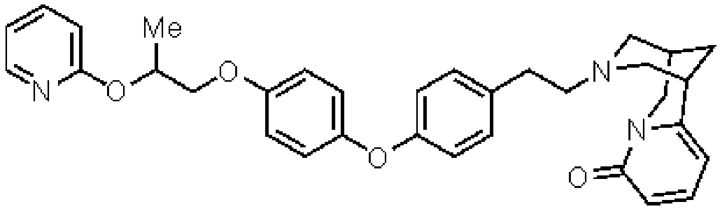

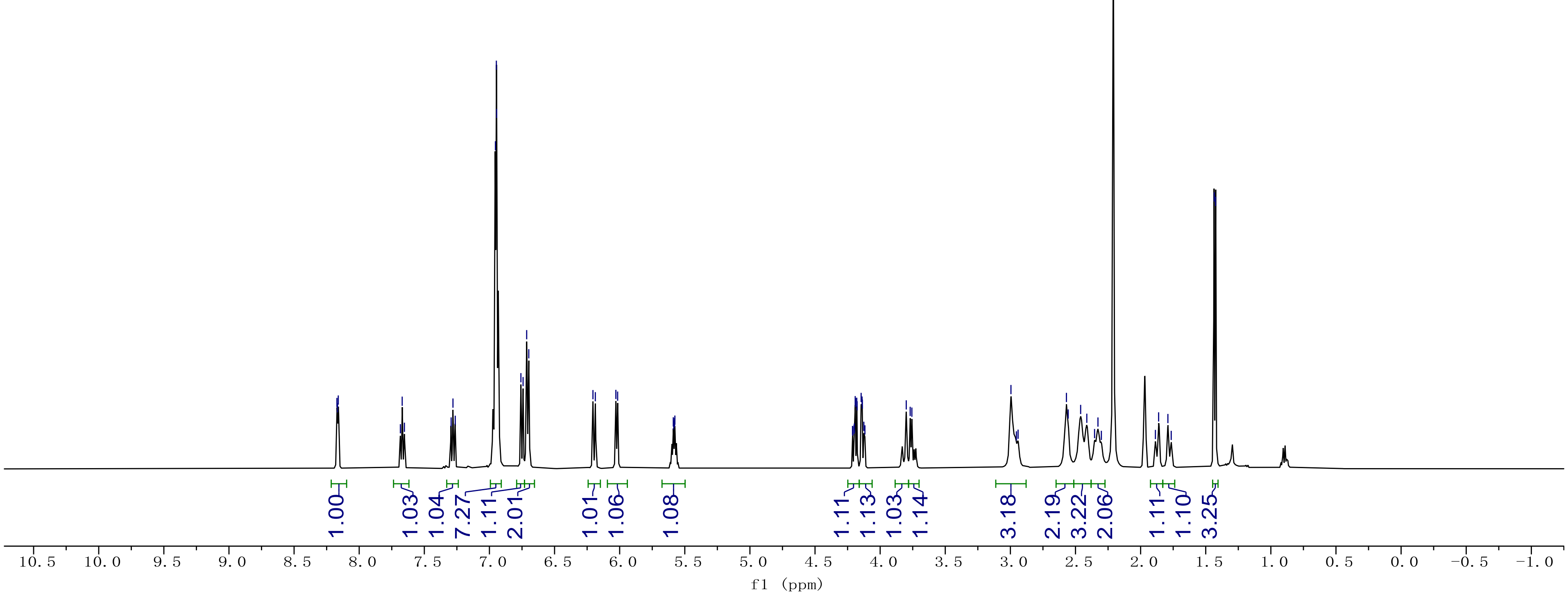

^{13}C NMR of pyriproxyfen- and cytisine-derived arylethylamine 27

CD_3CN, 23 ºC

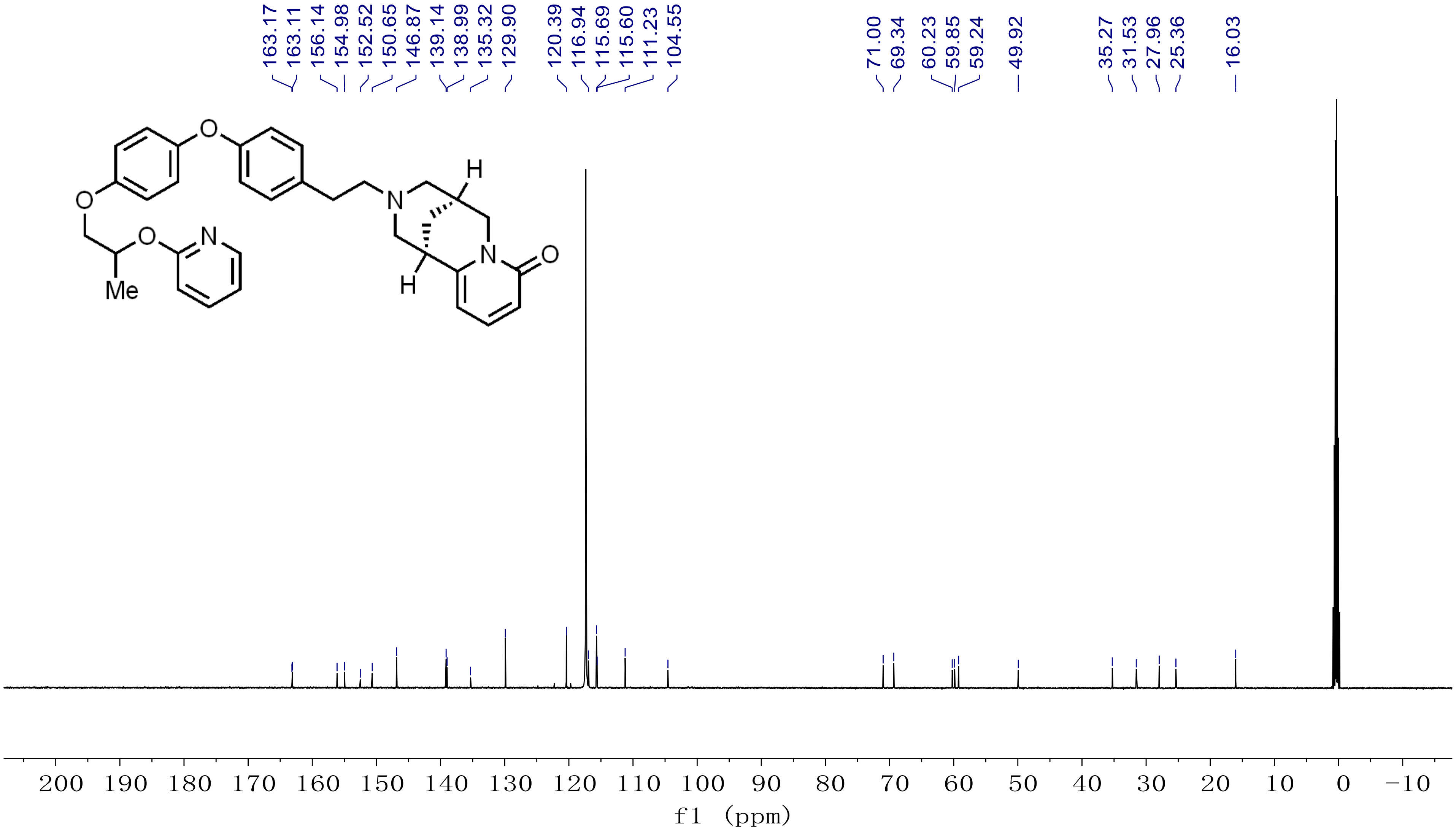

^{1}H NMR of arylethyl bromide 28

CDCl$_3$, 23 ºC

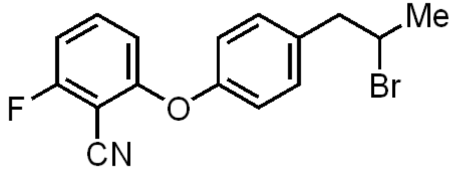

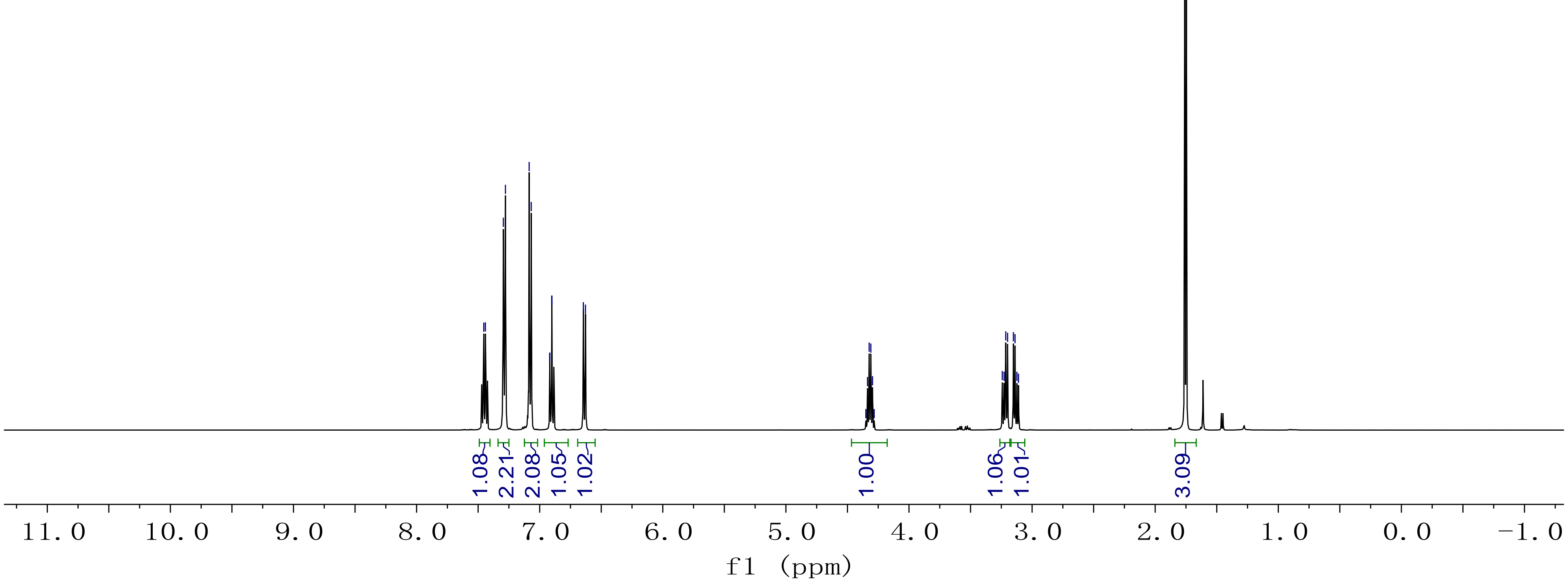

^{13}C NMR of arylethyl bromide 28

$CDCl_3$, 23 ºC

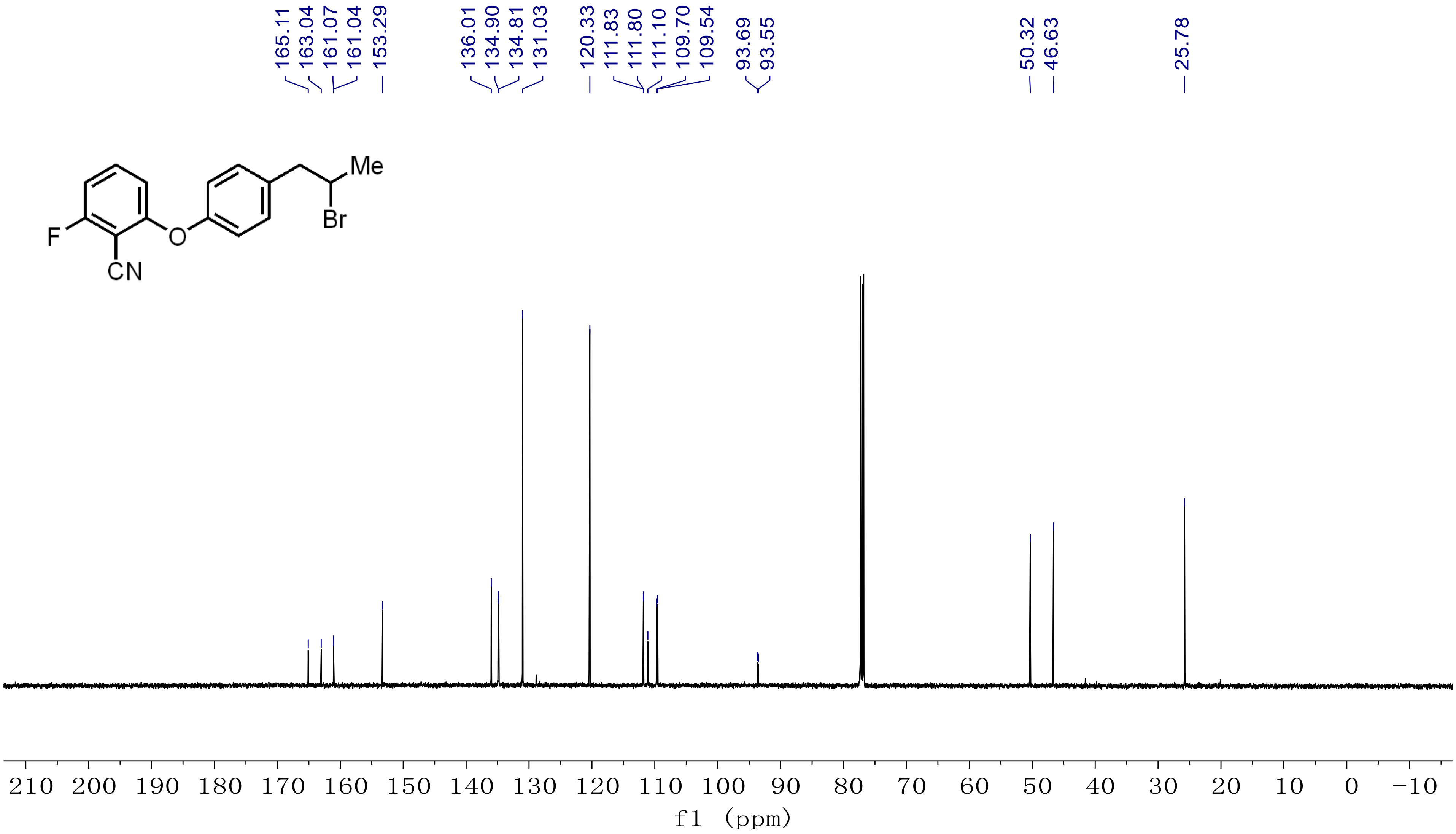

^{1}H NMR of arylethyl bromide 29

CDCl$_3$, 23 ºC

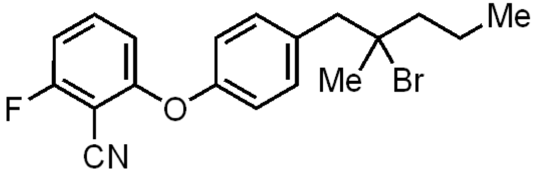

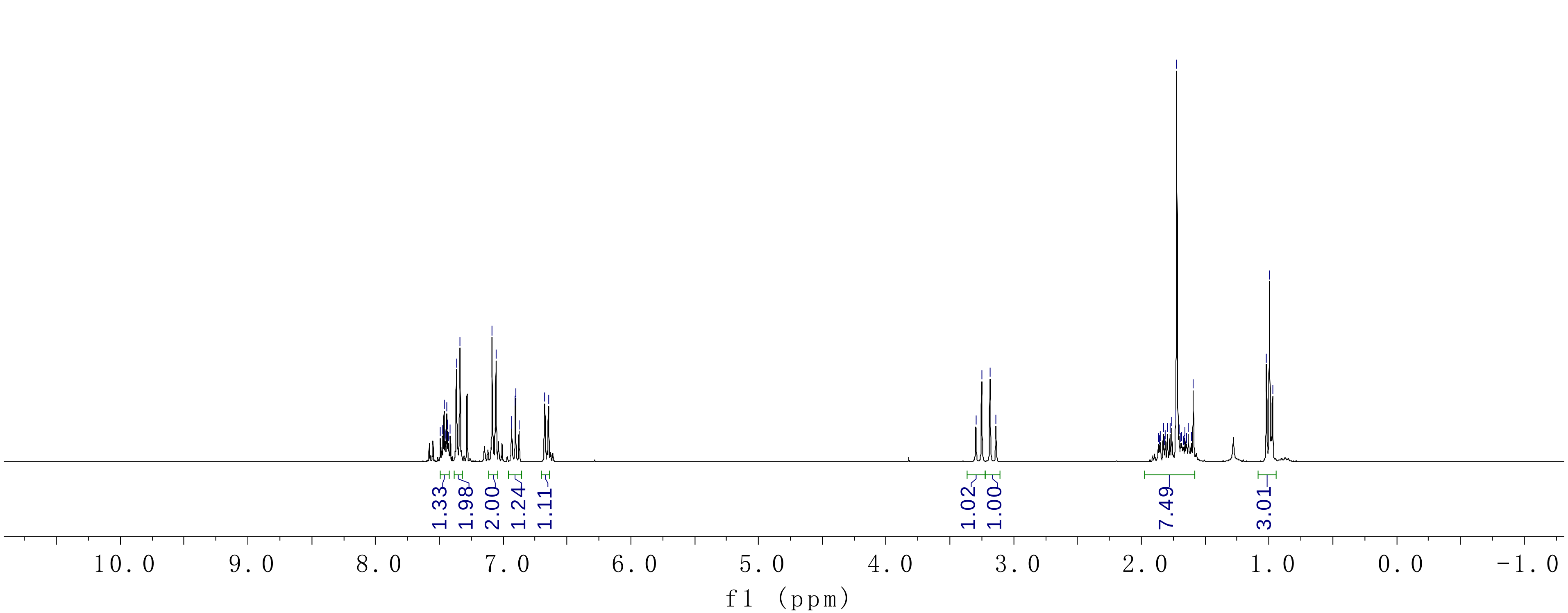

^{13}C NMR of arylethyl bromide 29

$CDCl_3$, 23 ºC

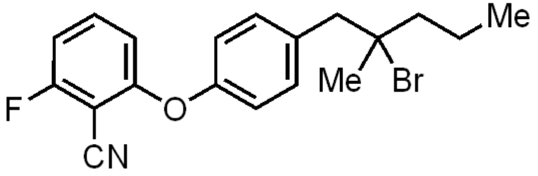

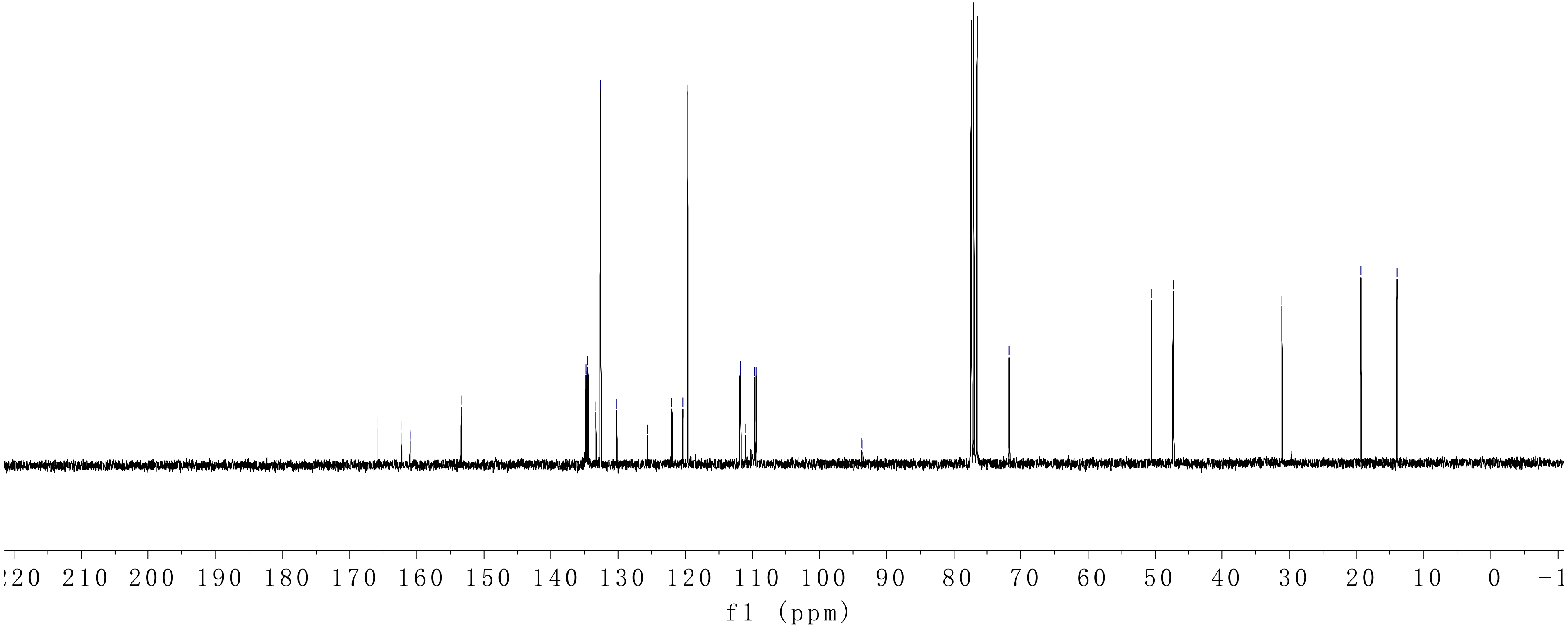

^{1}H NMR of arylethyl bromide 30

$CDCl_3$, 23 ºC

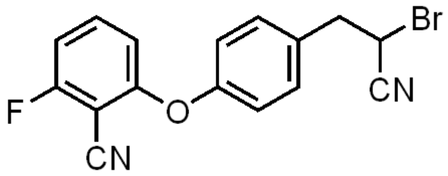

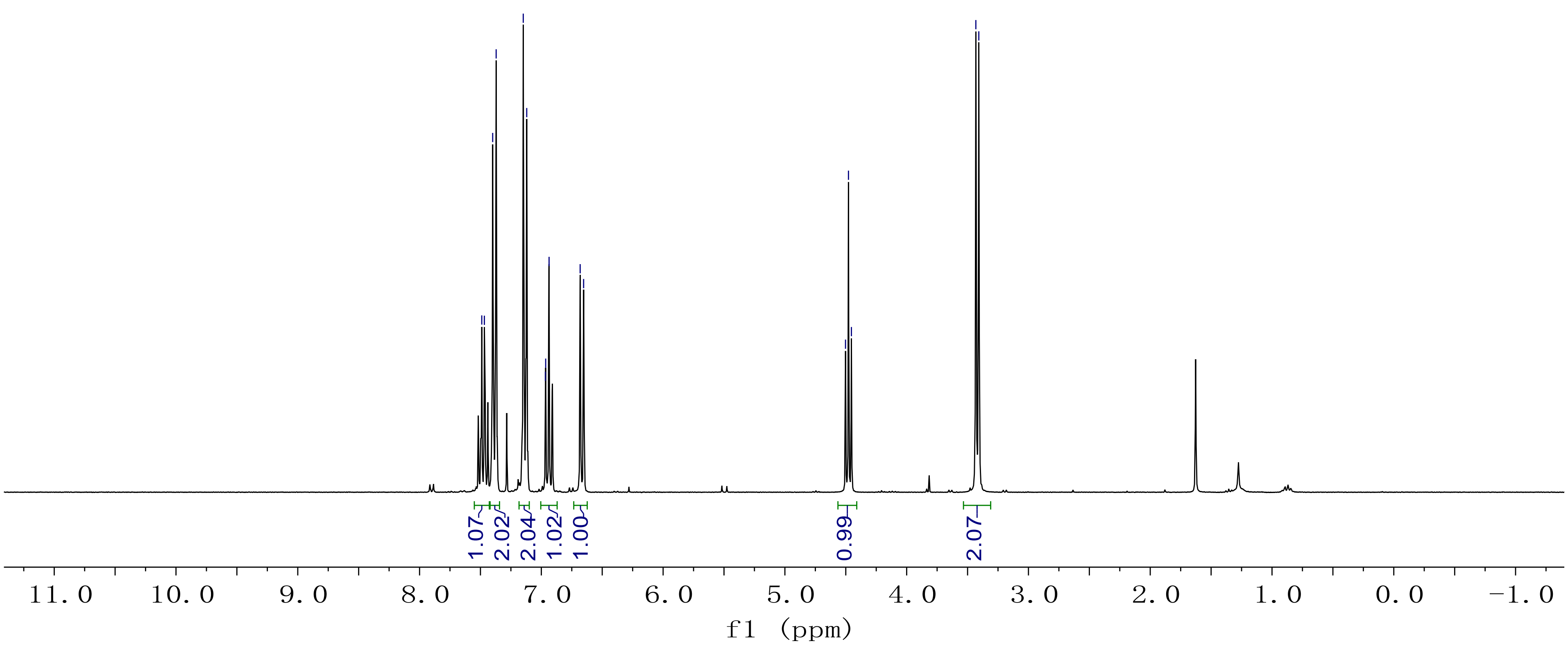

^{13}C NMR of arylethyl bromide 30

$CDCl_3$, 23 ºC

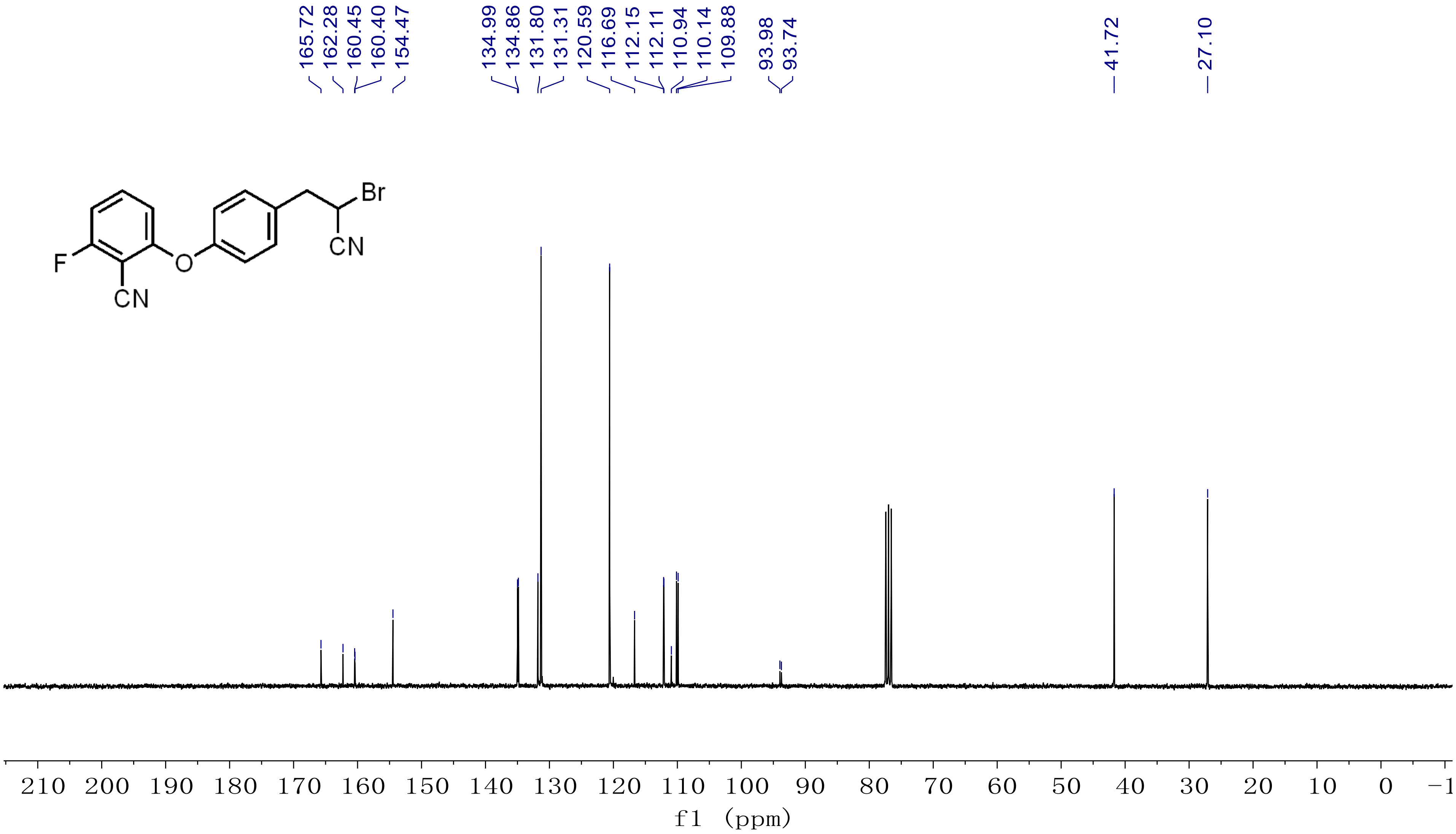

^{1}H NMR of arylethyl bromide 31

CDCl$_3$, 23 ºC

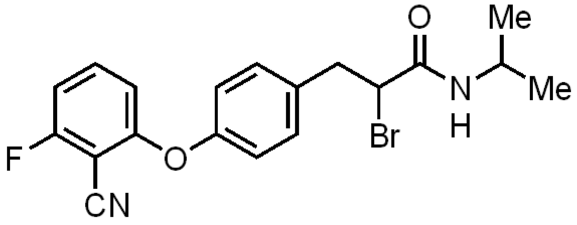

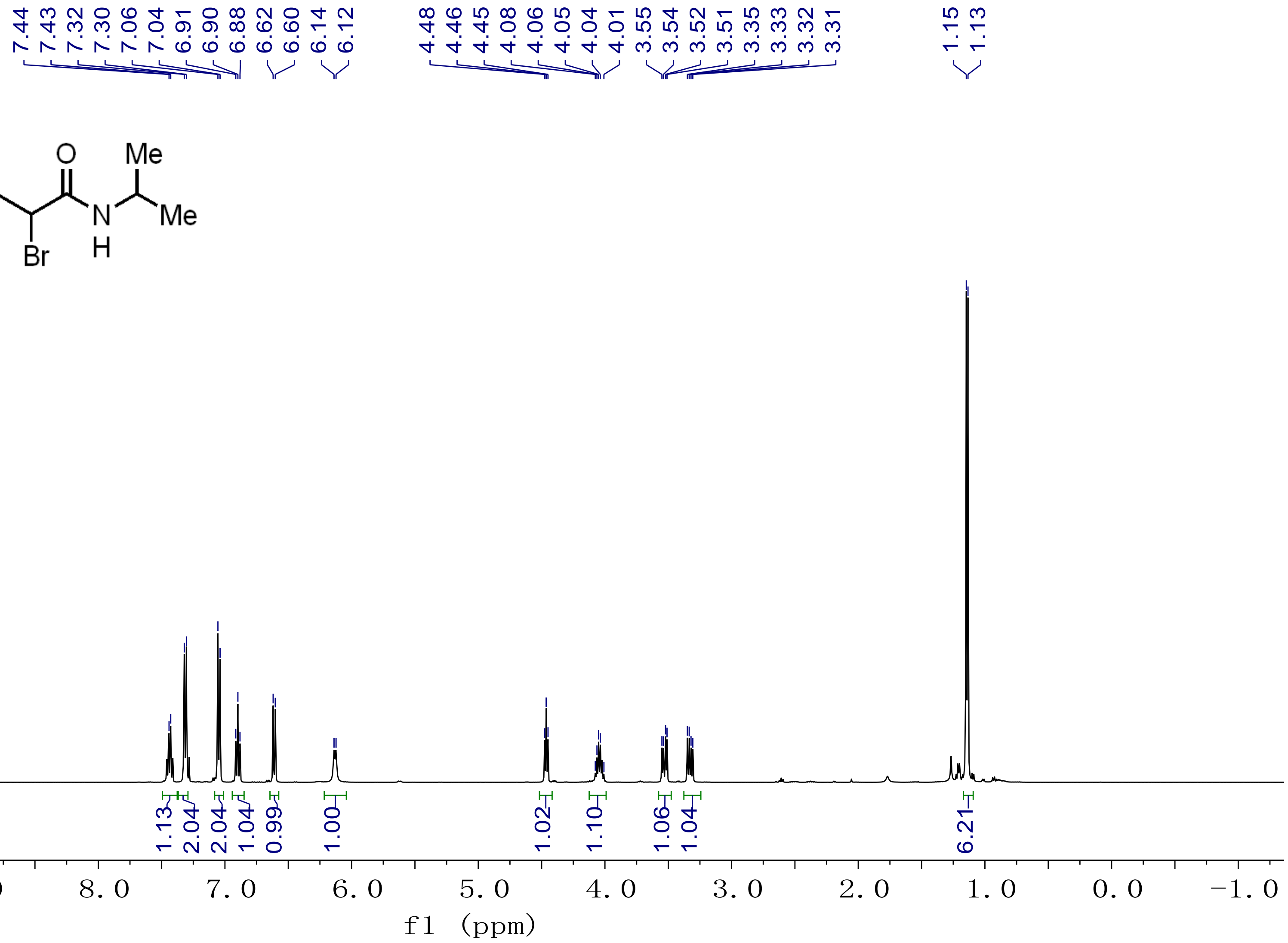

^{13}C NMR of arylethyl bromide 31

$CDCl_3$, 23 ºC

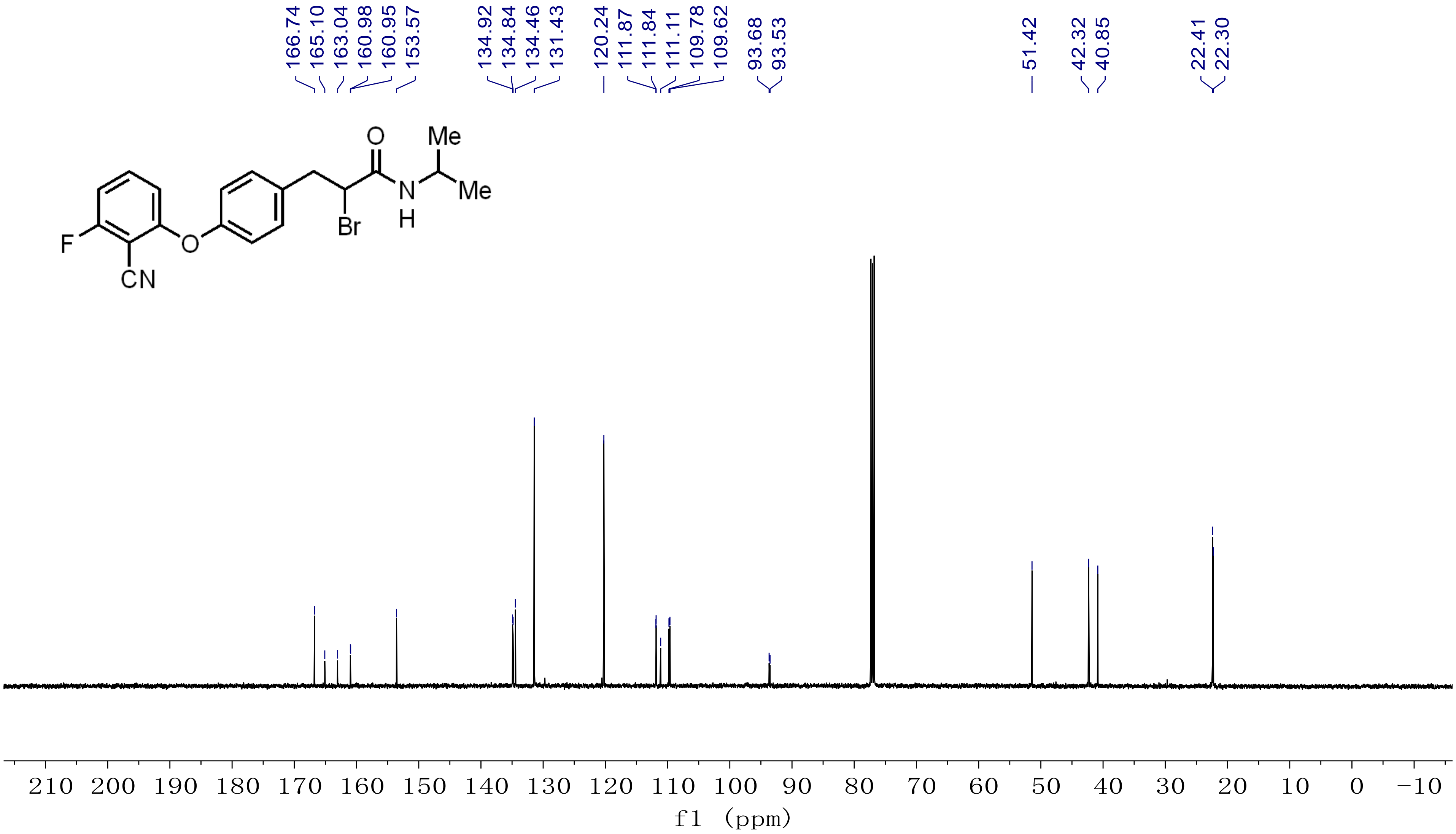

^{1}H NMR of arylethyl bromide 32

$CDCl_3$, 23 ºC

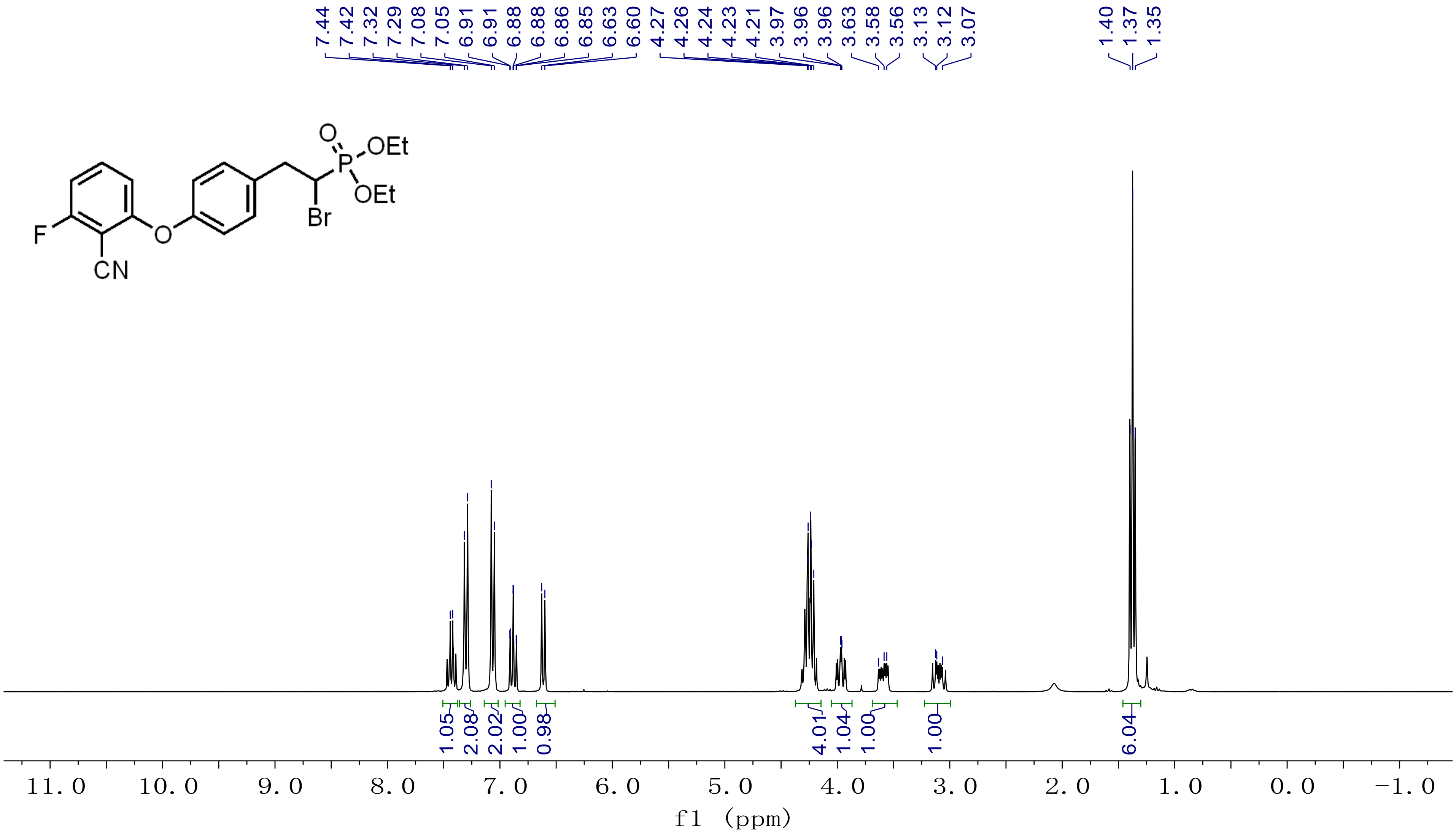

^{13}C NMR of arylethyl bromide 32

$CDCl_3$, 23 ºC

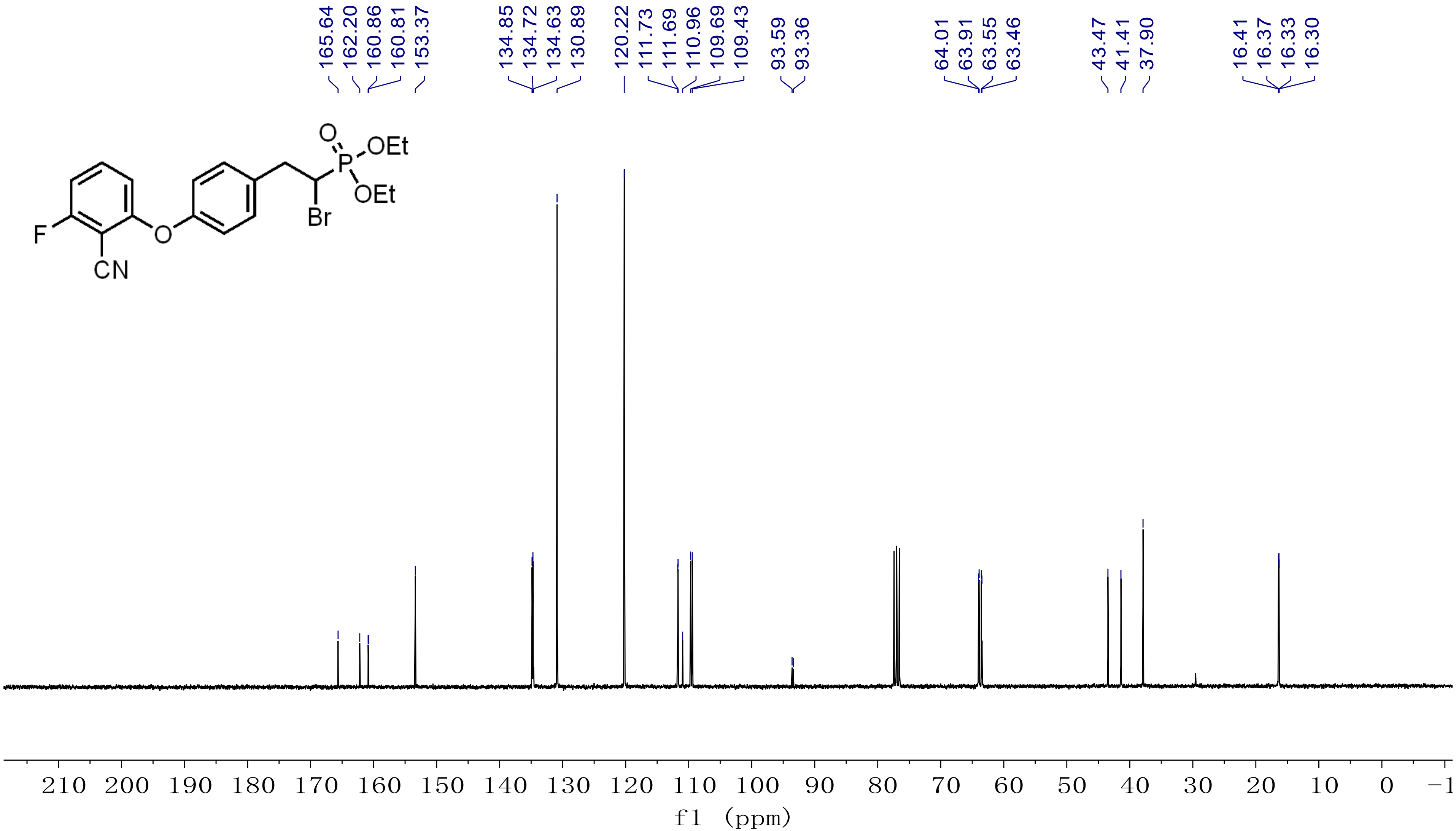

^{31}P NMR of arylethyl bromide 32

$CDCl_3$, 23 ºC

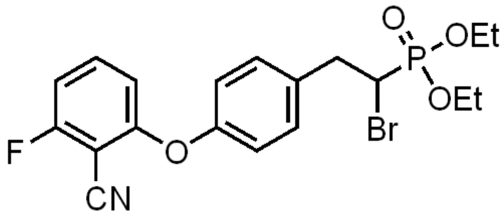

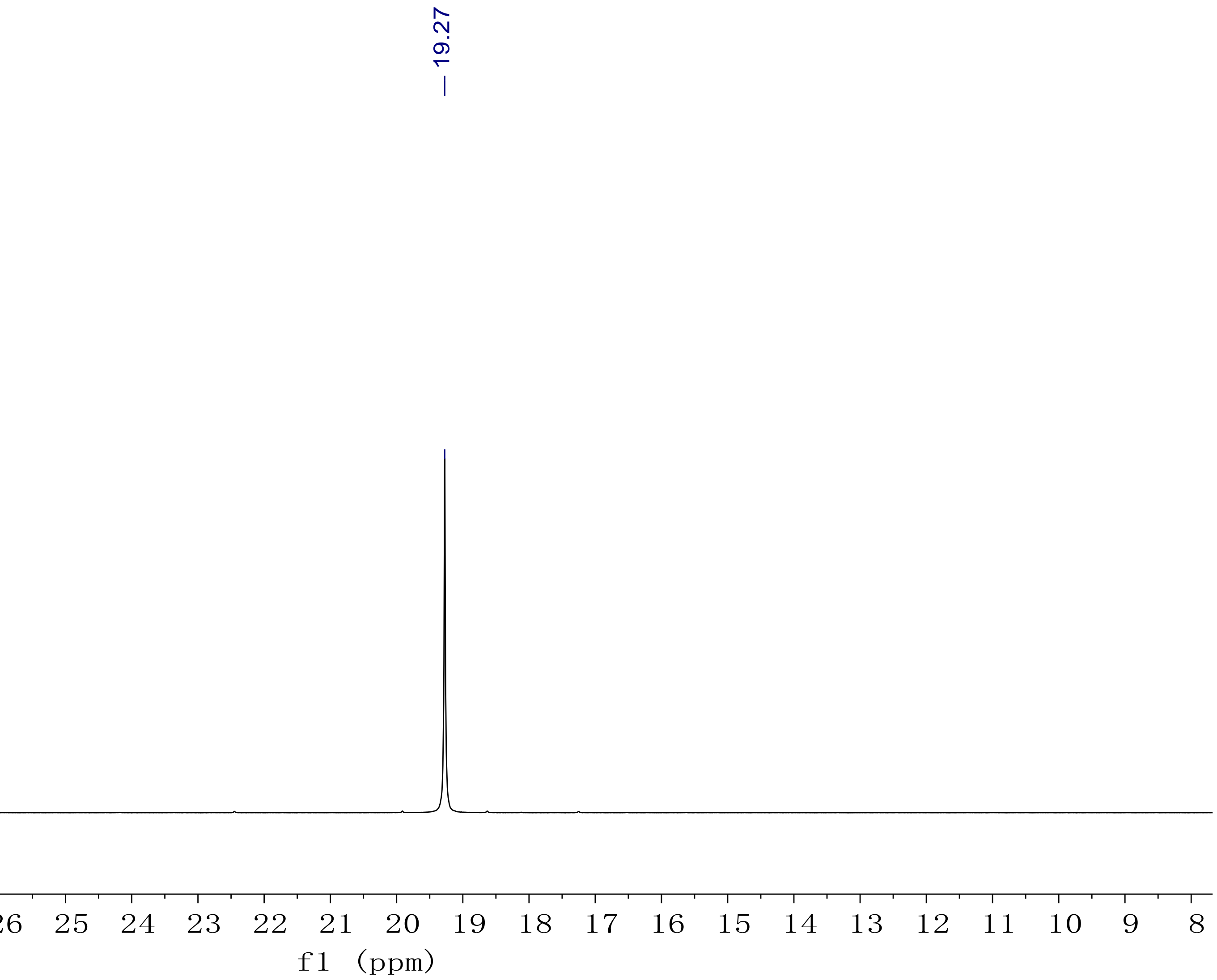

^{1}H NMR of arylethyl bromide 33

$CDCl_3$, 23 ºC

8.05 8.03 7.78 7.76 7.75 7.66 7.66 7.64 7.63 7.47 7.45 7.44 7.30 7.29 7.08 7.06 6.92 6.91 6.89 6.63 6.62 4.89 4.89 4.87 4.87 3.97 3.96 3.94 3.93 3.15 3.13 3.12 3.10

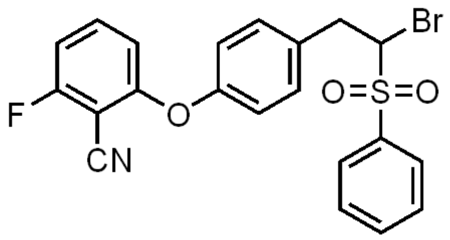

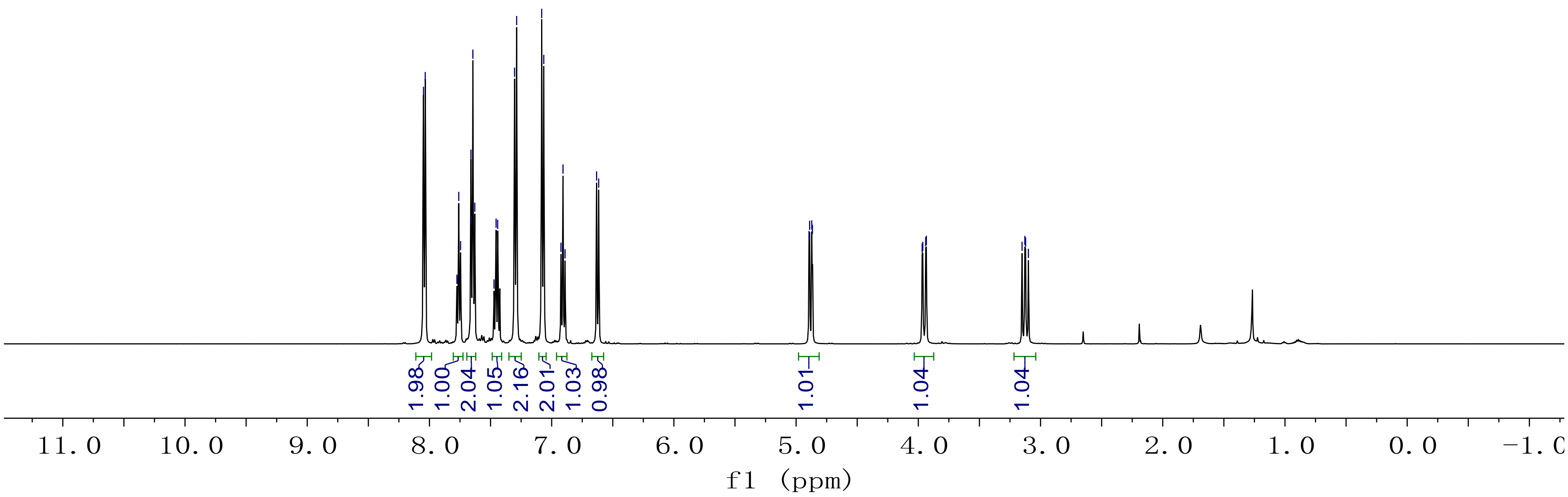

^{13}C NMR of arylethyl bromide 33

$CDCl_3$, 23 ºC

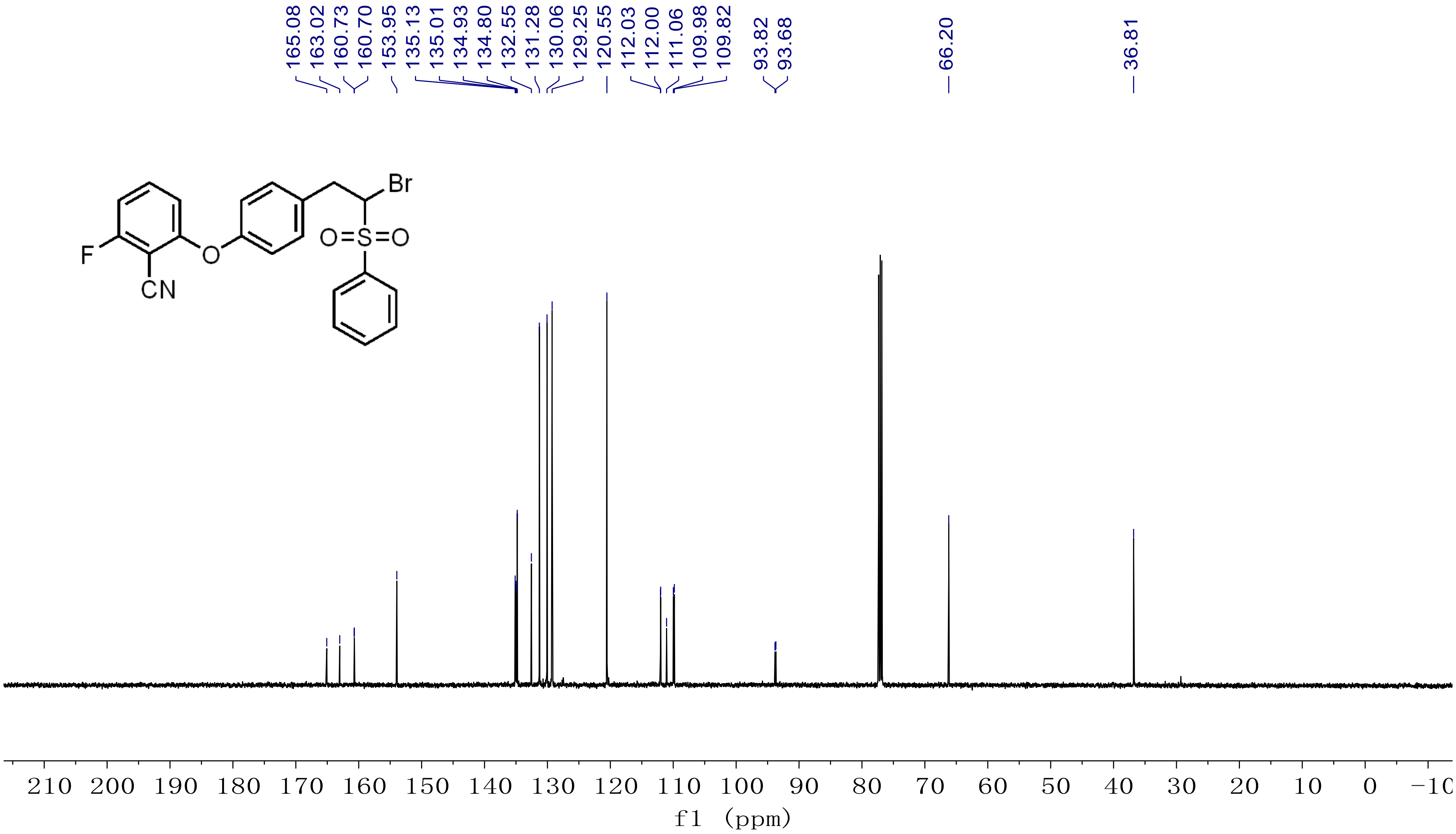

^{1}H NMR of arylethyl bromide 34

$CDCl_3$, 23 ºC

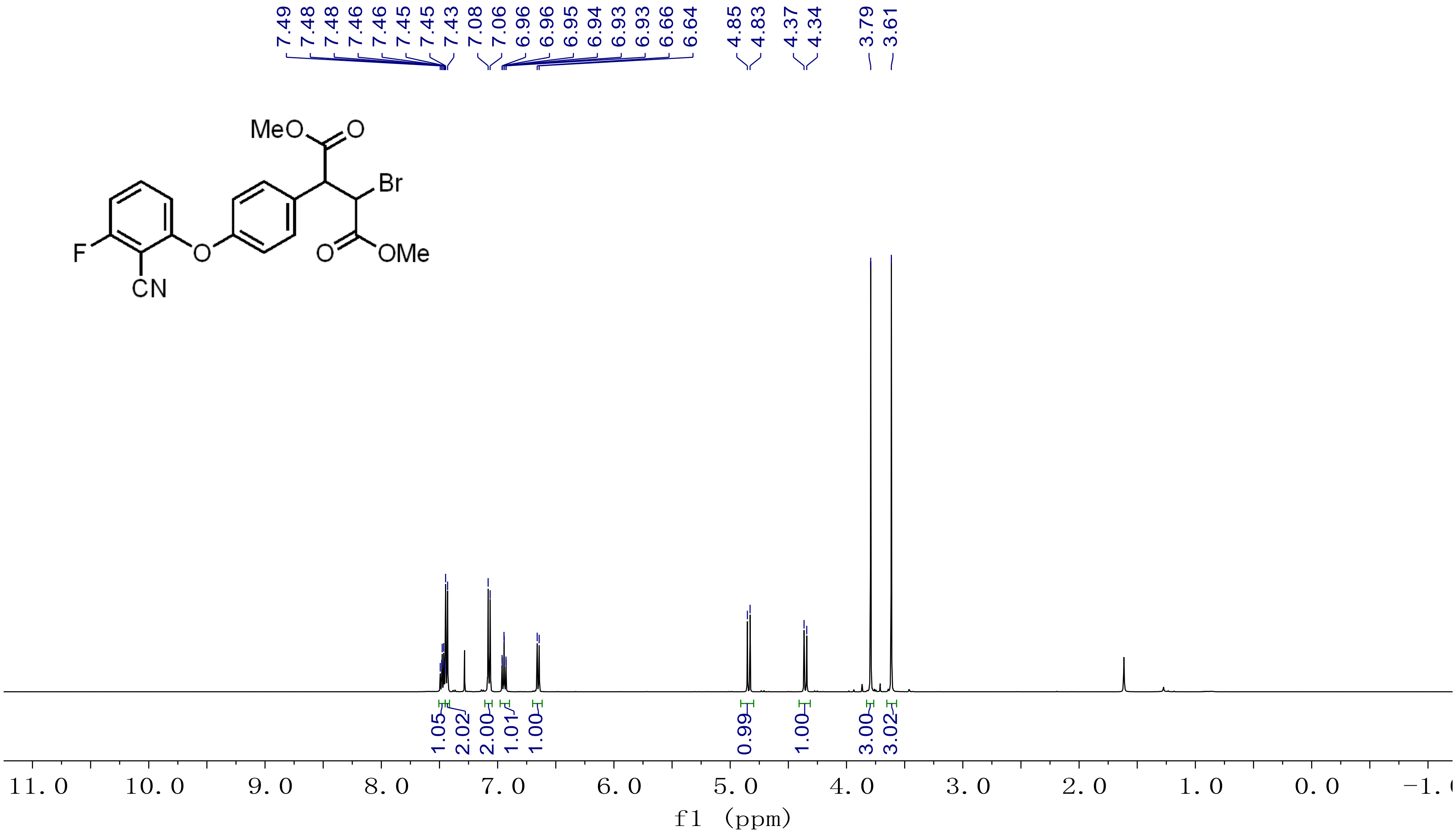

^{13}C NMR of arylethyl bromide 34

$CDCl_3$, 23 ºC

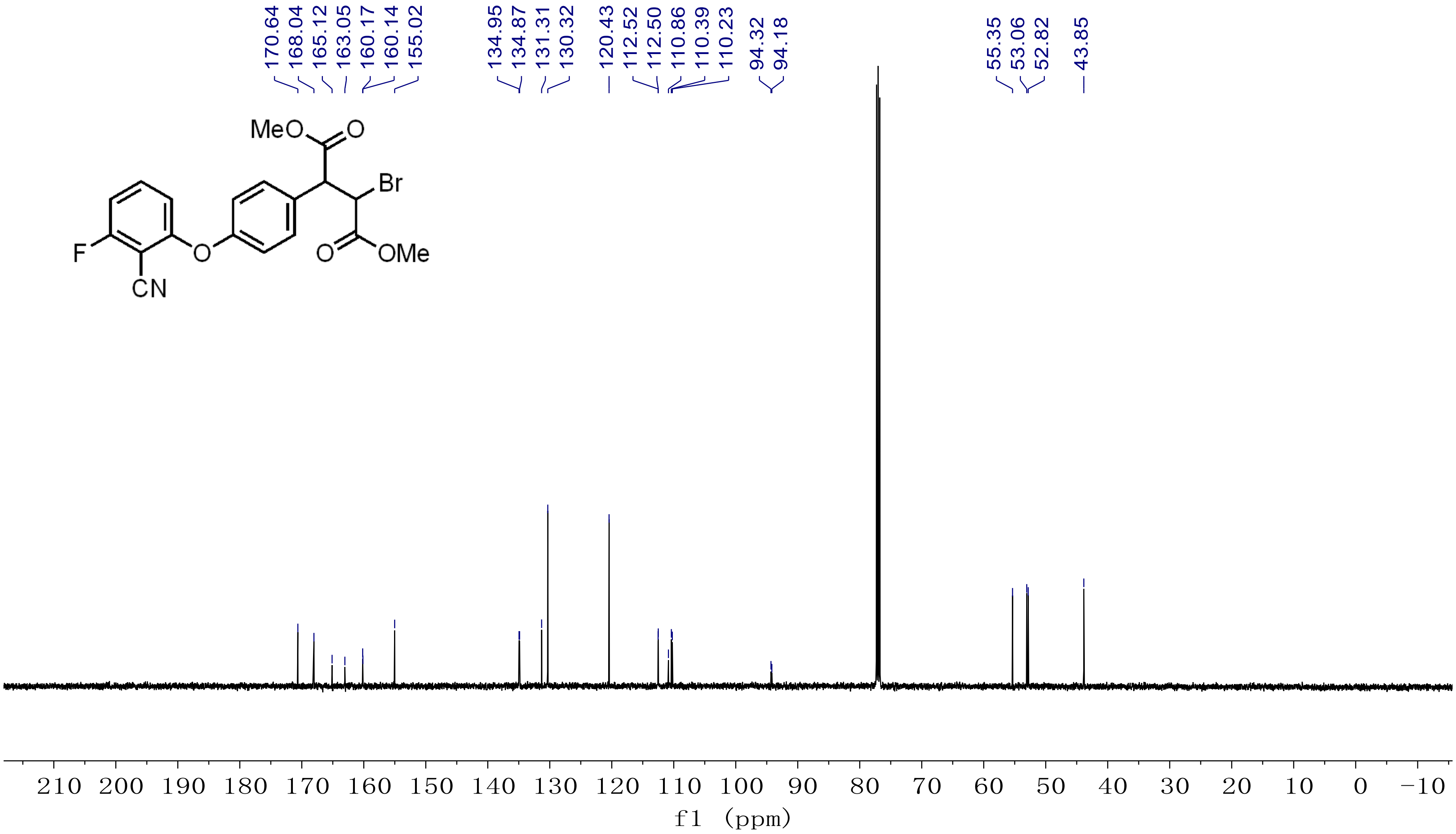

^{1}H NMR of arylethyl bromide 35

$CDCl_3$, 23 ºC

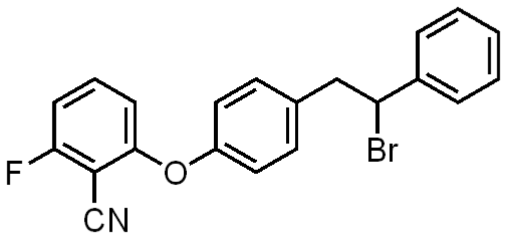

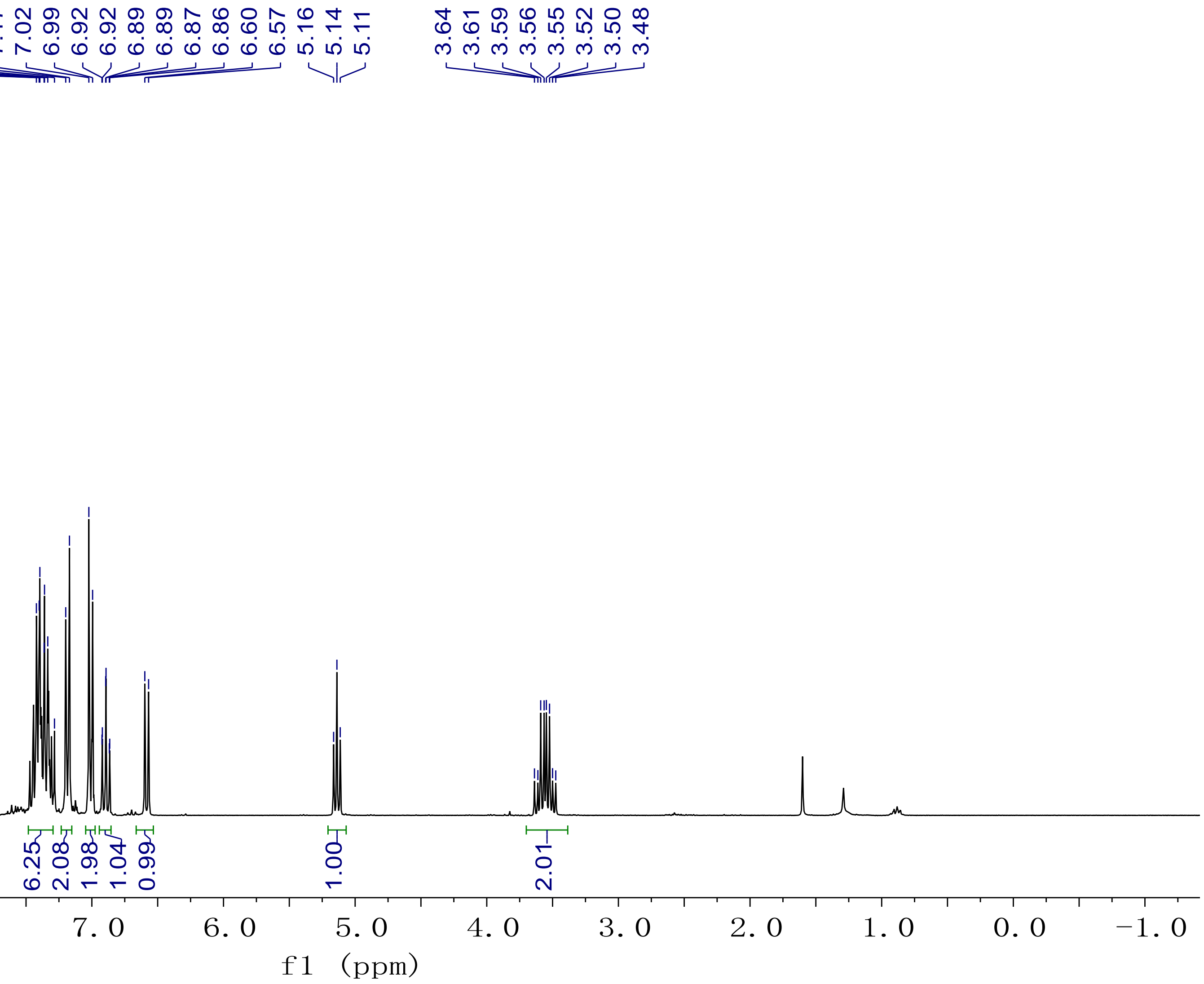

^{13}C NMR of arylethyl bromide 35

$CDCl_3$, 23 ºC

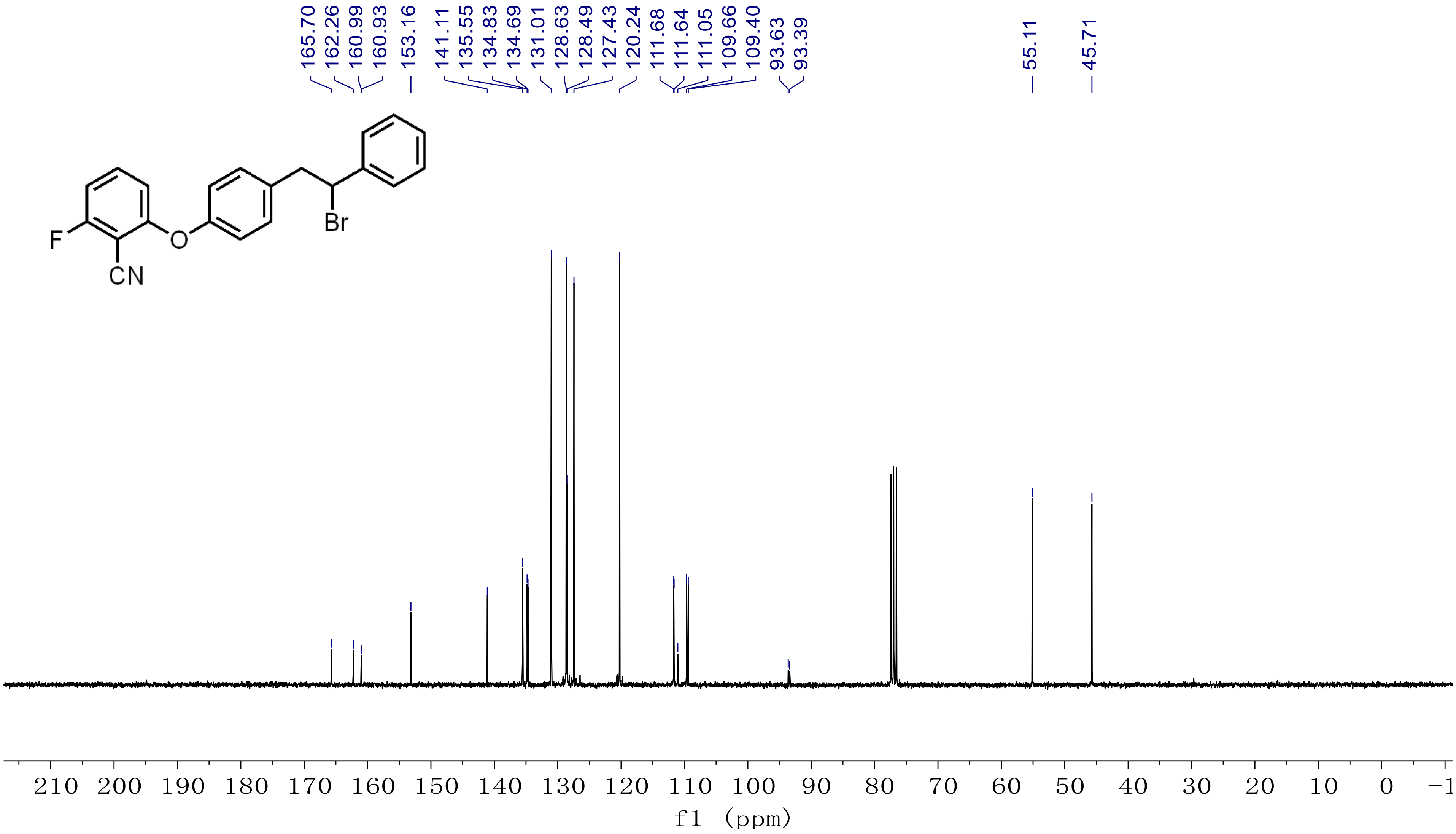

^{1}H NMR of arylethyl bromide 36

$CDCl_3$, 23 ºC

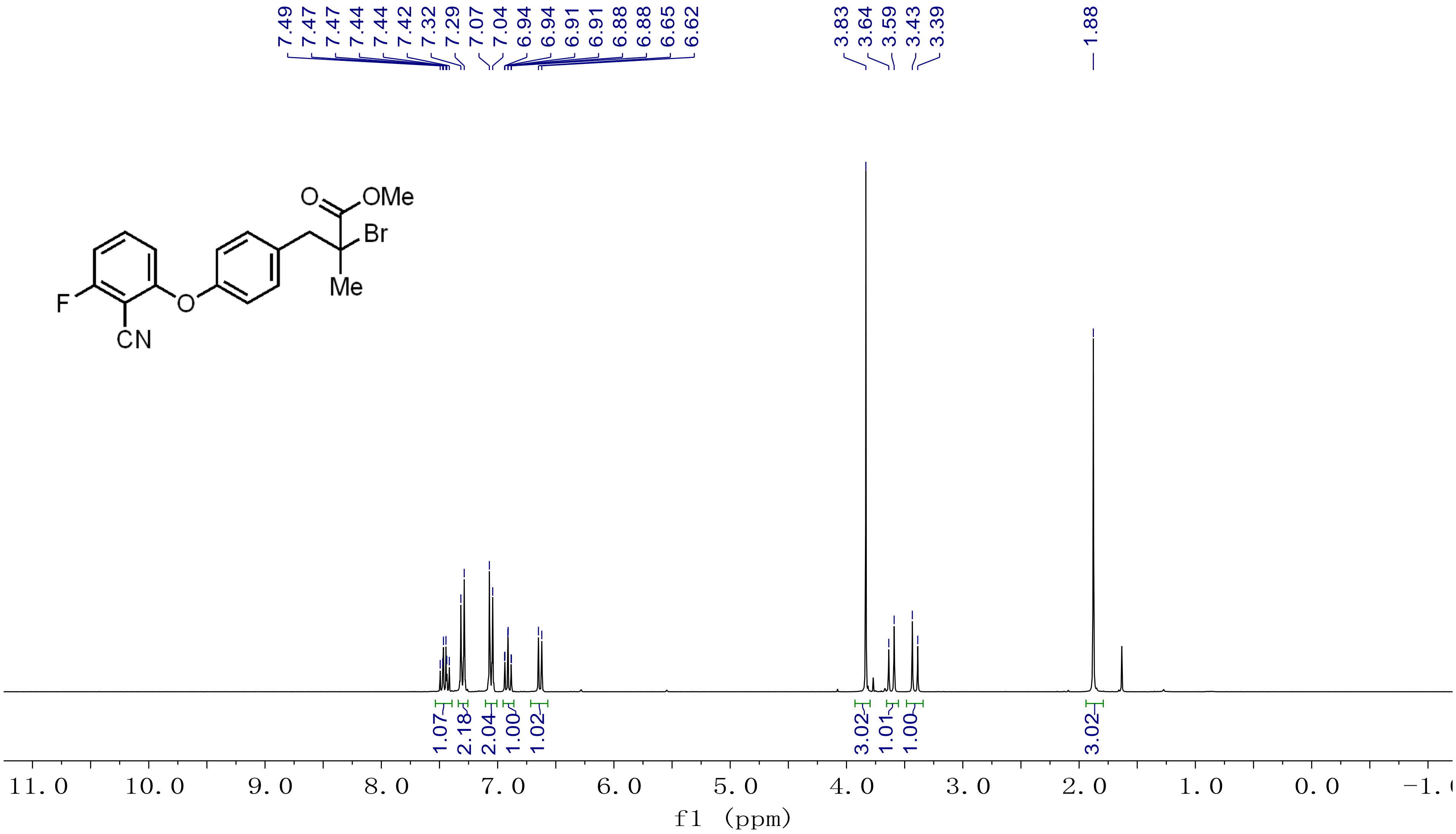

^{13}C NMR of arylethyl bromide 36

CDCl$_3$, 23 ºC

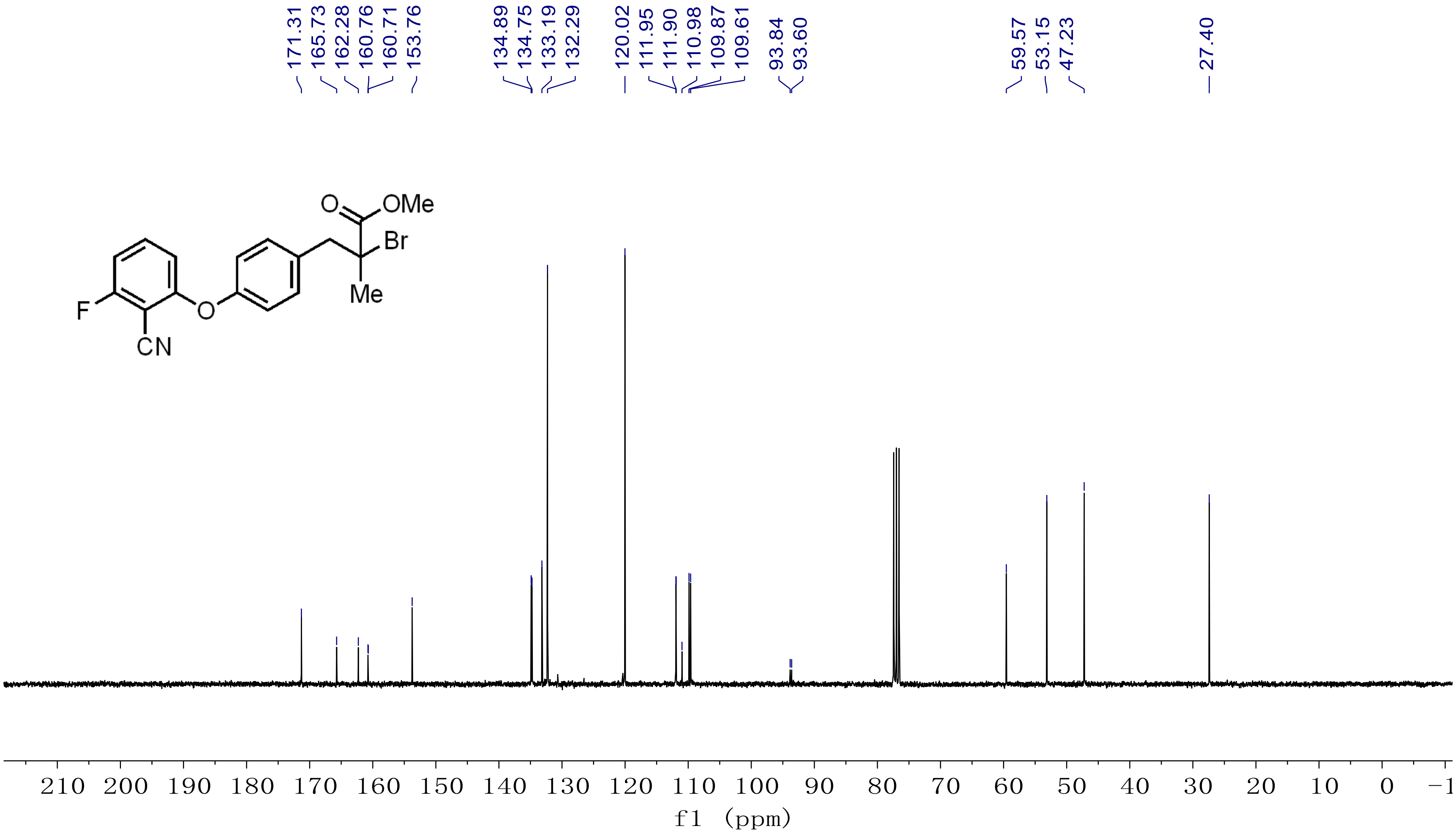

REFERENCES

1. Fulmer, G. R. et al. NMR Chemical Shifts of Trace Impurities: Common Laboratory Solvents, Organics, and Gases in Deuterated Solvents Relevant to the Organometallic Chemist. *Organometallics*, **29**, 2176–2179 (2010).
2. Berger, F. et al. Site-selective and versatile aromatic C–H functionalization by thianthrenation. *Nature* **567**, 223–228 (2019).
3. Elliott, L. D., Kayal, S., George, M. W. & Booker-Milburn, K. Rational design of triplet sensitizers for the transfer of excited state photochemistry from UV to visible. *J. Am. Chem. Soc.* **142**, 14947–14956 (2020).
4. Zähringer, T. J. B., Wienhold, M., Gilmour, R. & Kerzig, C. Direct Observation of Triplet States in the Isomerization of Alkenyl boronates by Energy Transfer Catalysis. *J. Am. Chem. Soc*. **145**, 21576−21586 (2023).
5. Xu, B. et al. Photocatalyzed diastereoselective isomerization of cinnamyl chlorides to cyclopropanes. *J. Am. Chem. Soc*. **142**, 6206–6215 (2020).
6. Luque, A. Groß, J. Zähringer, T. J. B. Kerzig, C. & Opatz, T. Vinylcyclopropane [3 + 2] Cycloaddition with Acetylenic Sulfones Based on Visible Light Photocatalysis. *Chem. –Eur. J.* **28**, No. e202104329 (2022).
7. Yang, Y.-M., Zhang, C., Yang, H. & Tang, Z.-Y. Photo-catalyzed acetoxysulfoximination of styrene with sulfoximidoyl thianthrenium salt. *Chem. Commun*. **58**, 8580–8583 (2022).
8. Thyrion, F. C. Flash Photolysis of Aromatic Sulfur Molecules. *J. Phys. Chem.* **77**, 1478–1482 (1973).
9. Shine, H. J. & Murata, Y. Kinetics and mechanism of the reaction of the thianthrene cation radical with water. *J. Am. Chem. Soc*. **91**, 1872–1874 (1969).
10. Kang, W.-J. et al. Discovery of a Thioxanthone–TfOH complex as a photoredox catalyst for hydrogenation of alkenes using p-xylene as both electron and hydrogen sources. *Angew. Chem. Int. Ed*. **61**, e202211562 (2022).
11. Yip, R. W. Szabo, A. G. & Tolg, P. K. Triplet State of Ketones in Solutions. Quenching Rate Studies of Thioxanthenone Triplets by Flash Absorption. *J. Am. Chem. Soc*. **95**, 4471−4472 (1973).
12. Carmichael, I. & Hug, G. L. Triplet−Triplet Absorption Spectra of Organic Molecules in Condensed Phases. *J. Phys. Chem. Ref. Data* **15**, 1−250 (1986).
13. Sorgo, M. de., Wasserman, B. & Szwarc, M. Aggregation of Salts of Thianthrene Radical Cations. *J. Phys. Chem*. **76**, 3468−3471 (1972).
14. Kuhn, H. J., Braslavsky, S. E. & Schmidt, R. Chemical actinometry (IUPAC technical report). *Pure Appl. Chem*. **76**, 2105–2146 (2004).
15. Cismesia, M. A. & Yoon, T. P. Characterizing chain processes in visible light photoredox catalysis. *Chem. Sci*. **6**, 5426–5434 (2015).

16. Wegner, E. E. & Adamson, A. W. Photochemistry of complex ions. III. Absolute quantum yields for the photolysis of some aqueous chromium(III) complexes. Chemical actinometry in the long wavelength visible region. *J. Am. Chem. Soc*. **88**, 394–404 (1966).

17. Neese, F. The ORCA program system. *Comput. Mol. Sci.* **2**, 73–78 (2012).

18. Lee, C., Yang, W. & Parr, R. G. Development of the Colle-Salvetti correlation-energy formula into a functional of the electron density. *Physical Review B* **37**, 785–789 (1988).

19. Grimme, S., Antony, J., Ehrlich, S. & Krieg, H. A consistent and accurate ab initio parametrization of density functional dispersion correction (DFT-D) for the 94 elements H–Pu. *J. Chem. Phys.* **132**, 154104 (2010).

20. Grimme, S., Ehrlich, S. & Goerigk, L. Effect of the damping function in dispersion corrected density functional theory. *J. Comput. Chem*. **32**, 1456–1465 (2011).

21. Weigend, F. Accurate Coulomb-fitting basis sets for H to Rn. *Physical Chemistry Chemical Physics* **8**, 1057–1065 (2006).

22. Weigend, F. & Ahlrichs, R. Balanced basis sets of split valence, triple zeta valence and quadruple zeta valence quality for H to Rn: Design and assessment of accuracy. *Physical Chemistry Chemical Physics* **7**, 3297–3305 (2005).

23. Barone, V. & Cossi, M. Quantum calculation of molecular energies and energy gradients in solution by a conductor solvent model. *The Journal of Physical Chemistry A* **102**, 1995–2001 (1998).

24. Grimme, S. & Neese, F. Double-Hybrid Density Functional Theory for Excited Electronic States of Molecules. *J. Chem. Phys.* **127**, 154116 (2007).

25. Hanwell, M. D. et al. Avogadro: an advanced semantic chemical editor, visualization, and analysis platform. *Journal of Cheminformatics* **4**, 17 (2012).

26. Zhurko, G. A. Chemcraft-graphical program for visualization of quantum chemistry computations. https://chemcraftprog.com.

27. Li, J. et al. Photoredox catalysis with aryl sulfonium salts enables site-selective late-stage fluorination. *Nat. Chem*. **12**, 56–62 (2020).

28. Morov, S. L., Carmicheal, I. & Hug, G. L. Handbook of Photochemistry (Marcel Dekker, 1993).
